# Supplementary material for: High-Throughput/High Content Imaging Screen Identifies Novel Small Molecule Inhibitors and Immunoproteasomes as Therapeutic Targets for Chordoma
Source: Pharmaceutics. 2023 Apr 18;15(4):1274. doi: 10.3390/pharmaceutics15041274 (PMC10145398; doi:10.3390/pharmaceutics15041274)
Supplement: Supplementary file 1 [file pharmaceutics-15-01274-s001.zip › pharmaceutics-2201815-supplementary.pdf]

Supplementary Table S1: List of Z’ factor for library plates

Sl. No.	Plate ID	Vendor	Replicate A	Replicate B
1	2089	Biomol 4	0.698	0.485
2	2090	Biomol 4	0.517	0.517
3	3651	Selleck Chem	0.459	0.450
4	3652	Selleck Chem	0.882	0.526
5	3653	Selleck Chem	0.417	0.641
6	3654	Selleck Chem	0.525	0.343
7	3655	Selleck Chem	0.467	0.552
8	3656	Selleck Chem	0.645	0.539
9	3657	Selleck Chem	0.387	0.578
10	3720	Cayman Chemical	0.593	0.415
11	3721	Cayman Chemical	0.492	0.431
12	3722	Medchem Express	0.516	0.438
13	3723	Medchem Express	0.636	0.639
14	3724	Medchem Express	0.656	0.642
15	3725	Medchem Express	0.561	0.699
16	3726	Medchem Express	0.778	0.682
17	3727	Medchem Express	0.682	0.565
18	3728	Medchem Express	0.684	0.676
19	3729	Medchem Express	0.648	0.668
20	3730	Medchem Express	0.620	0.615

Supplementary Table S2: List of library and cherry-picked compounds from each plate

Sl. No.	Plate ID	Vendor	Cherry pick	No. of compounds
1	2089	Biomol 4	0	
2	2090	Biomol 4	0	640
3	3651	Selleck Chem	0	
4	3652	Selleck Chem	0	
5	3653	Selleck Chem	0	
6	3654	Selleck Chem	0	
5	3655	Selleck Chem	0	
6	3656	Selleck Chem	0	
7	3657	Selleck Chem	0	1902
8	3720	Cayman Chemical	3	
9	3721	Cayman Chemical	3	
10	3722	Medchem Express	0	
11	3723	Medchem Express	4	
12	3724	Medchem Express	2	
13	3725	Medchem Express	2	
14	3726	Medchem Express	7	
15	3727	Medchem Express	7	
16	3728	Medchem Express	9	
17	3729	Medchem Express	2	
18	3730	Medchem Express	11	1600
		Total	50	4142

Supplementary Table S3: List of IC_{50} values for cherry picked compounds

**Compound name**	**IC_{50} (μM)**
Ascomycin	0.749
ONX 0914	ND
Ingenol-3-angelate	0.2787
NVP-BEP800	0.366
Radicicol	0.0000722
MI-773	0.8217
HY-10299	0.5974
MLN 8237	0.02419
MG-132	0.03263
KPT-330	ND
Palbociclib	0.1067
AZD8055	ND
Volasertib	1.192
Erlotinib	1.171
Cilengitide	1.166
OTSSP167	1.176
Flavopiridol	1.230
BAY 61-3606	1.229
Ruboxistaurin	1.214
Ispinesib	0.9359
Obatoclax	2.68
BI 2536	0.573
NVP-TAE 226	0.9528
SGI-1776	0.9178
LY2090314	0.7019
Quisinostat	0.8575
MEK162	1.212
PKC412	2.270
Amsacrine	0.6594
BNC105	0.9265
Tipifarnib	0.9954
Epoxomicin	0.5021
OSI-027	0.9978
RG7388	1.165
Duvelisib	0.6935
LEE011	2.222
PKI-587	1.211
CBL0137 HCl	1.193
AZD-7762	0.9865
MK 2206	4.17e-010
Nutlin (3a)	1.516
ARRY-520	1.086

Zotarolimus	1.051
PF-431396	1.060
TAK-285	1.103
CH5132799	1.210
AZD-5438	373.4
LY2835219	1.461
Icotinib	1.129
Tozasertib	1.189

Supplementary Table S4: List of the top hits and their SMILES

Compound name	SMILES
MLN 8237	COc1cccc(c1C1=NCc2c(c3c1cc(Cl)cc3)nc(nc2)Nc1ccc(c(c1)OC)C(=O)O)F
MG-132	O=C[C@@H](NC(=O)[C@@H](NC(=O)[C@@H](NC(=O)OCc1ccccc1)CC(C)C)CC(C)C)CC(C)C
Palbociclib	CC(=O)c1c(C)c2cnc(nc2n(c1=O)C1CCCC1)Nc1ccc(cn1)N1CCNCC1
BAY 61-3606 (dihydrochloride)	O=C(N)C1=CC=CN=C1NC2=NC(C3=CC=C(C(OC)=C3)OC)=CC4=NC=CN24.[H]Cl.[H]Cl
BNC105	COC1=CC(C(C2=C(C)OC3=C(O)C(OC)=CC=C23)=O)=CC(OC)=C1OC
Tipifarnib	O=C1N(C2=C(C(C3=CC=CC(Cl)=C3)=C1)C=C(C=C2)[C@@](N)(C4=CN=CN4C)C5=CC=C(C=C5)Cl)C
OSI-027	O=C([C@H]1CC[C@H](C2=NC(C(N3)=CC4=C3C(OC)=CC=C4)=C5C(N)=NC=NN52)CC1)O
ARRY-520	O=C1N(C2=C(C(C3=CC=CC(Cl)=C3)=C1)C=C(C=C2)[C@@](N)(C4=CN=CN4C)C5=CC=C(C=C5)Cl)C
Icotinib (Hydrochloride)	C#CC1=CC(NC2=C(C=C(OCCOCCOCCO3)C3=C4)C4=NC=N2)=CC=C1.[H]Cl
Bortezomib	B([C@H](CC(C)C)NC(=O)[C@H](CC1=CC=CC=C1)NC(=O)C2=NC=CN=C2)(O)O

Supplementary Table S5: Structures of the top hits

Compound name	Structures
MLN 8237	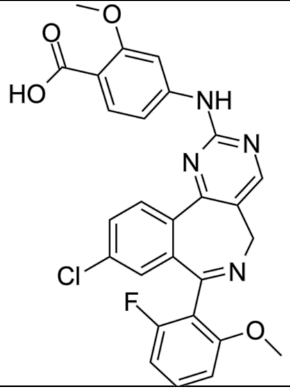
MG-132	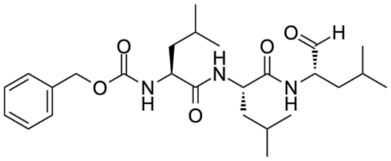
Palbociclib	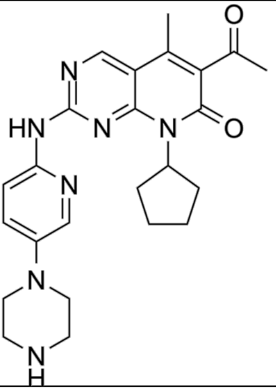
BAY 61-3606 (dihydrochloride)	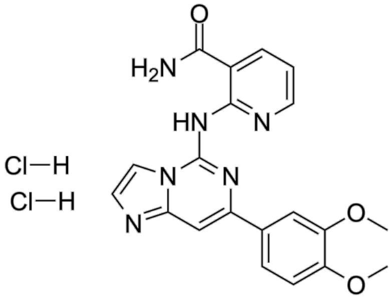

BNC105	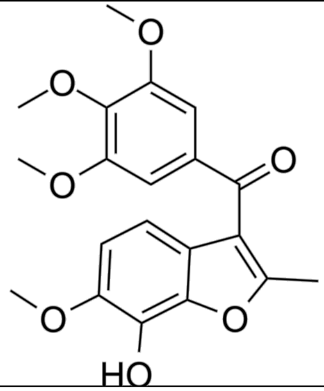
Tipifarnib	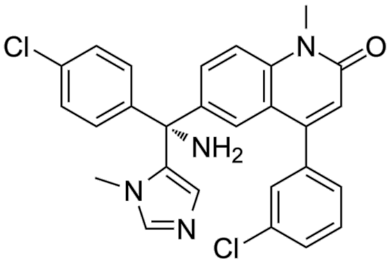
OSI-027	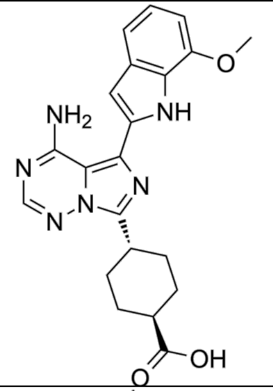
ARRY-520	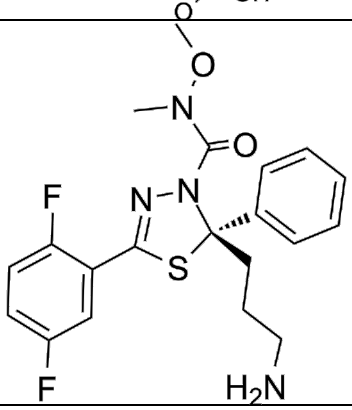

Icotinib (Hydrochloride)	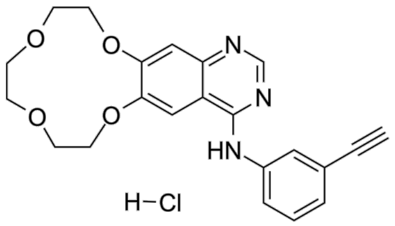
Bortezomib	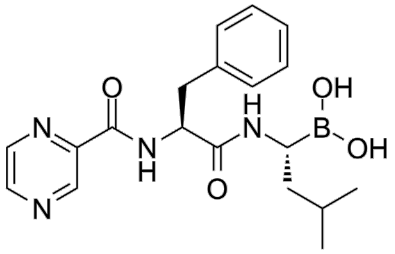

Supplementary Table S6: List of compounds screened in primary screening

Vendor	Vendor Reagent ID	SMILES	Molecular Formula	Molecula	Compound Name(s)
BIOMOL	DL-552	N1(CCOCC1)C(=N)NC(=N)N.Cl	C6H14ClN5O	207.661	Moroxydine HCl
BIOMOL	AC-554	N(CCN(C)C1)(c2c(cccc2)Cc3c4cccc3)C14	C18H20N2	264.365	Mianserin hcl
BIOMOL	A-190	[C@@H]1([C@@H]([C@H](C)Cl)NC(=O)[C@@H]2C[C@@H](CCC)CN2C)[C@H](O)[C@H](O)[C@@H](O)[C@@H](SC)O1	C18H33ClN2O5S	424.983	Clindamycin HCl
BIOMOL	AC-615	c([nH]cn1)(C(=O)N(C)C(=O)N2C)c12	C7H8N4O2	180.164	Aminophylline
BIOMOL	A-192	[C@@]1([H])([C@H](O)[C@H](O)[C@@H](OC(=O)CCCCCCCCCCCCCCC)[C@@H](SC)O1)C(C(Cl)C)NC(=O)[C@@]2([H])N(C)C[C@]([H])(CCC)C	C34H63ClN2O6S	663.392	Clindamycin PO4
BIOMOL	AC-626	c1(nc(C)[nH]2)c2C(=O)N(C)C(=O)N1Cc3occc3	C12H12N4O3	260.249	Furafylline
BIOMOL	A-195	[C@H]1([C@H](O)[C@H](C)C\C=C\C)C(=O)N[C@@H](CC)C(=O)N(C)CC(=O)N(C)[C@@H](CC(C)C)C(=O)N[C@@H](C(C)C)C(=O)N(C)[C@@H](CC(C)C)C(=O)N[C@@H](C)C(=O)N[C@H](C)C(=O)N(C)[C@@H](CC(C)C)C(=O)N(C)[C@@H](CC(C)C)C(=	C62H111N11O12	1202.611	Cyclosporin a
BIOMOL	DL-561	[C@H]1(CC[C@@H](C(C)C)CC1)C(=O)N[C@@H](C(=O)O)Cc2ccccc2	C19H27NO3	317.423	nateglinide
BIOMOL	A-239	[C@H]1([C@@H](O)[C@@H](O)[C@H](O)[C@H](O)[C@H]1O)O[C@H]2O[C@H](C)[C@@H](NC(=N)C(=O)O)C[C@@H]2N	C14H25N3O9	379.363	Kasugamycin
BIOMOL	AC-729	c1(C(O)CNC(C)C)ccc(O)c(O)c1	C11H17NO3	211.258	Isoproterenol HCl (rac)
BIOMOL	A-240	[C@H]1([C@H](O)[C@H](O)[C@@H](O)[C@@H](SC)O1)[C@@H]([C@H](O)C)NC(=O)[C@H]2N(C)C[C@H](CCC)C2	C18H34N2O6S	406.537	Lincomycin
BIOMOL	AC-734	[N+](C)(C)(C)CCOC(=O)C	C7H16NO2	146.207	Acetylcholine Cl
BIOMOL	A245	c(c(C(=O)OC)ccc1O)(nc(c2n3)c(O)cc(O)c2C=O)c13	C15H10N2O6	314.25	Lomofungin
BIOMOL	AC-735	[C@@H]1(OC(=O)C(CO)c2ccccc2)C[C@@]3([H])N(C)[C@]([H])(CC3)C1	C17H23NO3	289.369	Atropine sulfate
BIOMOL	A-249	c(COC1=O)(c(C)c(OC)c(C\C=C(/C)\CCC(=O)O)c2O)c12	C17H20O6	320.337	Mycophenolic acid
BIOMOL	AC-737	C(CCCCCCCCC[N+](C)(C)C)[N+](C)(C)C	C16H38N2	258.486	Decamethonium 2Br
BIOMOL	A-256	c(c(C)c(O[C@@H]1OC(C)(C)[C@H](OC)[C@@H](OC(=O)N)[C@@H]1O)cc2)(OC(=O)C(NC(=O)c3ccc(O)c(C\C=C(\C)/C)c3)=C4O)c24	C31H36N2O11	612.624	Novobiocin Na
BIOMOL	AC-741	C1(C)(NC)C(C)(C)[C@@]2([H])C[C@]1([H])CC2	C11H21N	167.291	Mecamylamine HCl
BIOMOL	A-275	N(CCCC1)(C(=O)C(=O)[C@]2(O)O[C@@H](CC[C@H]2C)C[C@H](OC)\C(\C)=C\C=C\C=C\[C@@H](C)C[C@@H](C)C(=O)[C@H](OC)[C@H](O)\C(\C)=C\[C@@H](C)C(=O)C[C@@H]([C@H](C)C[C@@H]3CC[C@@H](O)[C@H](OC)C3)OC4=O)[C@@H]14	C51H79NO13	914.172	Rapamycin
BIOMOL	AC-743	c1(OC(=O)N(C)C)cccc([N+](C)(C)C)c1	C12H19N2O2	223.291	Neostigmine Br
BIOMOL	AR-103	c1(nsn2)c2ccc(Cl)c1NC3=NCCN3	C9H8ClN5S	253.711	Tizanidine HCl
BIOMOL	EI-217	[C@@H]1(C[C@@H]1N)c2ccccc2	C9H11N	133.19	Tranylcypromine
BIOMOL	DL-549	[C@@H](O)([C@@H](O)CNC)[C@H](O)[C@H](O)CO	C7H17NO5	195.214	Meglumine
BIOMOL	EI-219	c(cccc1)(C(O)=C(C(=O)Nc2ncccc2)N(C)S3(=O)=O)c13	C15H13N3O4S	331.346	Piroxicam
BIOMOL	AR-107	c1(cccc2)c2cccc1OC[C@@H](O)CNC(C)C	C16H21NO2	259.343	Propranolol HCl s(-)
BIOMOL	EI-240	c1(ccccc1)CC(C)N(C)CC#C	C13H17N	187.281	Deprenyl HCl r (-)
BIOMOL	AR-112	c1(c(OCC(O)CNCCOc2ccccc2OC)ccc3)c3[nH]c4c1cccc4	C24H26N2O4	406.474	Carvedilol
BIOMOL	EI-249	[C@@]12(C(=O)[C@H](C)[C@@H](OC(=O)C)[C@@H](C)[C@@H](C)OC(=O)[C@H](C)[C@@H](O[C@@H]3O[C@@H](C)[C@H](OC(=O)C)[C@@H](OC)C3)[C@H](C)[C@@H](O[C@@H]4O[C@H](C)C[C@H](N(C)C)[C@H]4OC(=O)C)[C@@H](C)C1)OC2	C41H67NO15	813.968	Troleandomycin
BIOMOL	DL-223	c1(c2ccc(F)cc2)c(COC)c(C(C)C)nc(C(C)C)c1\C=C\[C@@H](O)C[C@@H](O)CC(O)=O	C26H34FNO5	459.55	Cerivastatin
BIOMOL	EI-265	[C@@](C)(NN)(C(=O)O)Cc1ccc(O)c(O)c1	C10H14N2O4	226.229	Carbidopa
BIOMOL	AW8655	c(cc(c1c2O)C(=O)c(c3C1=O)cccc3O)([C@@H](C(=O)OC)[C@@](O)(CC)C[C@@H]4O[C@@H]5O[C@@H](C)[C@@H](O[C@@H]6O[C@@H](C)[C@@H](O[C@H]7CCC(=O)[C@H](C)O7)[C@@H](O)C6)[C@@H](N(C)C)C5)c24	C42H53NO15	811.868	Aclarubicin
BIOMOL	EI-287	c1(Oc2ccccc2)cc([N+]([O-])=O)ccc1NS(=O)(=O)C	C13H12N2O5S	308.31	Nimesulide
BIOMOL	B-100	n1(cnc2C(=O)OCC)c2CN(C)C(=O)c3c1ccc(F)c3	C15H14FN3O3	303.288	Flumazenil
BIOMOL	EI-288	c1(C(=O)c2ccccc2)cccc(C(C)C(=O)O)c1	C16H14O3	254.281	Ketoprofen
BIOMOL	GR-103	C1(\C=C\C(\C)=C\C=C\C(\C)=C\C(=O)Nc2ccc(O)cc2)=C(C)CCCC1(C)C	C26H33NO2	391.546	Fenretinide
BIOMOL	EI-292	c(cccc1)(C(O)=C(C(=O)Nc2sc(C)cn2)N(C)S3(=O)=O)c13	C14H13N3O4S2	351.401	Meloxicam
BIOMOL	DL-269	c1(Nc2ccc(F)c(Cl)c2)ncnc3c1cc(OCCCN4CCOCC4)c(OC)c3	C22H24ClFN4O3	446.902	Gefitinib
BIOMOL	EI-318	c1(cccc2)c2cccc1CN(C)C\C=C\C#CC(C)(C)C	C21H25N	291.43	Terbinafine HCl
BIOMOL	DL-270	c1(C(=O)C(C)C)c(C(C)C)nn2c1cccc2	C14H18N2O	230.306	Ibudilast
BIOMOL	EI320	c1(ccccc1)CCCC([O-])=O.[Na+]	C10H11NaO2	186.183	Sodium phenylbutyrate

BIOMOL	A-280	[C@]([H])([C@@H](NC)[C@@H](O)[C@@H](NC)[C@@H]1O)(O[C@@]([C@]2([H])O3)(O)C(=O)C[C@@H](C)O2)[C@@]13[H]	C14H24N2O7	332.35	Spectinomycin
BIOMOL	AC-745	[C@@]1(CC2)([C@H](C[C@@]([H])(C3=C4)[C@@]([H])([C@@]5([H])OC4)[C@]1([H])N6C(=O)C5)N2C3)c7c6cccc7	C21H22N2O2	334.412	Strychnine HCl
BIOMOL	AC-104	N(Cc1ccccc1)(CC(COCC(C)C)N2CCCC2)c3ccccc3	C24H34N2O	366.54	Bepridil
BIOMOL	AC-746	c([C@@H]1[N+](C)(C)CC2)(c2cc(OC)c3O)c3Oc(ccc4C[C@H]5c(c6CCN5C)cc(c(OC)c6)Oc7cc(ccc7O)C1)cc4	C37H41N2O6	609.731	Tubocurarine Cl (+)
BIOMOL	AC-105	c1(C(=O)c2cc(I)c(OCCN(CC)CC)c(I)c2)c(CCCC)oc3c1cccc3	C25H29I2NO3	645.312	Amiodarone
BIOMOL	AC-747	c(c1ccc2)(c2CCc3c4cccc3)[C@@]4([H])CN([C@]15[H])CC[C@@](O)(C(C)(C)C)C5	C25H31NO	361.52	Butaclamol (+)
BIOMOL	AC-108	C1(C(C(=O)OC)=C(C)NC(C)=C1C(=O)OCCN(C)Cc2ccccc2)c3cccc([N+]([O-])=O)c3	C26H29N3O6	479.525	Nicardipine
BIOMOL	AC-748	c(c1CCN2C)([C@@]2([H])Cc3c4c(O)c(O)cc3)c4ccc1	C17H17NO2	267.322	Apomorphine r (-)
BIOMOL	AC-109	N1(C2CCN(CCCC(c3ccc(F)cc3)c4ccc(F)cc4)CC2)C(=O)Nc5c1cccc5	C28H29F2N3O	461.546	Pimozide
BIOMOL	AC-749	N1(CCCN(C)C)c2c(cccc2)Sc3c1cc(Cl)cc3	C17H19ClN2S	318.864	Chlorpromazine HCl
BIOMOL	AC-110	C(C(=O)N(C)C)(CCN1CCC(O)(c2ccc(Cl)cc2)CC1)(c3ccccc3)c4ccccc4	C29H33ClN2O2	477.038	Loperamide
BIOMOL	AC-751	N1(C2CCN(CCCN3C(=O)Nc4c3cccc4)CC2)C(=O)Nc5c1ccc(Cl)c5	C22H24ClN5O2	425.911	Domperidone
BIOMOL	AC-116	C12(CCN(CCCC(c3ccc(F)cc3)c4ccc(F)cc4)CC1)C(=O)NCN2c5ccccc5	C29H31F2N3O	475.573	Fluspirilene
BIOMOL	AC-753	c1(cc(C(F)(F)F)cc2)c2Sc3c(cccc3)N1CCCN4CCN(CCO)CC4	C22H26F3N3OS	437.522	Fluphenazine 2HCl
BIOMOL	AC-117	c1(ccc(C)cc1)S(=O)(=O)NC(=O)NCCCC	C12H18N2O3S	270.348	Tolbutamide
BIOMOL	AC-755	c(c1[nH]c2)(c2C[C@]3([H])[C@]4([H])C[C@@H](CSC)CN3CCC)c4ccc1	C19H26N2S	314.488	Pergolide mesylate
BIOMOL	AC-118	C(=N\C#N)(/NC(C)C(C)(C)C)\Nc1ccncc1	C13H19N5	245.323	Pinacidil
BIOMOL	AC-757	c1(C(=O)NC[C@@H]2CCCN2CC)c(O)c(Cl)cc(Cl)c1OC	C15H20Cl2N2O3	347.237	Raclopride l-tartrate s(-)
BIOMOL	AC-120	c1(S(=O)(=O)NC(=O)NC2CCCCC2)ccc(CCNC(=O)c3ncc(C)nc3)cc1	C21H27N5O4S	445.535	Glipizide
BIOMOL	AC-758	c1(C2CCN(CCC3=C(C)N=C4N(CCCC4)C3=O)CC2)noc5c1ccc(F)c5	C23H27FN4O2	410.484	Risperidone
BIOMOL	DL-271	n1c(ccnc1Nc2c(C)ccc(NC(=O)c3ccc(CN4CCN(C)CC4)cc3)c2)c5cccnc5	C29H31N7O	493.603	Imatinib
BIOMOL	FR-111	[C@H](Cc1c[nH]c(S)n1)(C([O-])=O)[N+](C)(C)C	C9H15N3O2S	229.299	Ergothioneine
BIOMOL	DL-548	c([C@@]([H])(O[C@@H]1O[C@@H](C)[C@@H](O)[C@@H](N)C1)C[C@](O)(C(=O)C)C2)(c(O)c(c3c4O)C(=O)c(c5C3=O)cccc5)c24	C26H27NO9	497.494	Idarubicin
BIOMOL	FR-112	c1(CN[C@@H]2CC[C@@H](O)CC2)cc(Br)cc(Br)c1N	C13H18Br2N2O	378.103	Ambroxol
BIOMOL	DL-273	c12c(ccc(n1)\C=C\c3cccc([C@@H](CCc4ccccc4C(C)(C)O)SCC5(CC(O)=O)CC5)c3)ccc(Cl)c2	C35H36ClNO3S	586.183	Montelukast
BIOMOL	FR114	C(OC)(C(=O)C(CCCCCCCCCCO)=C(C)C1=O)=C1OC	C19H30O5	338.439	Idebenone
BIOMOL	EI-240	c1(ccccc1)C[C@@H](C)N(C)CC#C	C13H17N	187.281	Selegiline
BIOMOL	G-233	C1(=O)C[C@H](O)C[C@@H](CC[C@H]2[C@@H](C)C=CC([C@@]23[H])=CCC[C@@H]3OC([C@@H](C)CC)=O)O1	C23H34O5	390.513	Mevastatin
BIOMOL	DL-275	n1nc(Cc2ccncc2)c(cccc3)c3c1Nc4ccc(Cl)cc4	C20H15ClN4	346.813	Vatalanib
BIOMOL	G-244	[C@]1([H])([C@@H](CC[C@H]2OC(=O)C[C@H](O)C2)[C@@H](C)C=C3)C3=C[C@H](C)C[C@@H]1OC(=O)C(C)(C)CC	C25H38O5	418.566	Simvastatin
BIOMOL	CT-100	[C@@]12(OC1)CC[C@@H](OC(=O)\C=C\C=C\C=C\C=C\C(=O)O)[C@@H](OC)[C@@]2([H])[C@@]3(C)O[C@@H]3C\C=C(/C)\C	C26H34O7	458.544	Fumagillone
BIOMOL	G-430	c(c(S([O-])(=O)=O)cc(S([O-])(=O)=O)c1)(c(NC(=O)c2ccc(C)c(NC(=O)c3cccc(NC(=O)Nc4cccc(C(=O)Nc5c(C)ccc(C(=O)Nc6ccc(S([O-])(=O)=O)c7c6c(S([O-])(=O)=O)cc(S([O-])(=O)=O)c7)c5)c4)c3)c2)ccc8S([O-])(=O)=O)c18	C51H34N6O23S6	1291.232	Suramin sodium
BIOMOL	DL-239	C1(CC)(Oc(c2C1)cccc2)C3=NCCN3	C13H16N2O	216.279	Efaroxan
BIOMOL	DL-282	c1(C[C@@H](C(=O)N[C@@H](CO)C(=O)N[C@H](C(=O)N[C@H](COC(C)(C)C)C(=O)N[C@@H](CC(C)C)C(=O)N[C@@H](CCCNC(=N)N)C(=O)N2CCC[C@H]2C(=O)NNC(=O)N)Cc3ccc(O)cc3)NC(=O)[C@@H](NC(=O)[C@H]4NC(=O)CC4)Cc5nc[nH]c5)c[nH	C59H84N18O14	1269.41	Goserelin acetate
BIOMOL	PG-007	[C@@]1([H])(C\C=C/CCCC(=O)O)C(=O)C[C@@H](O)[C@]1([H])\C=C\[C@@H](O)CCCCC	C20H32O5	352.465	Dinoprostone
BIOMOL	DL-283	c1(OC)ccccc1O	C7H8O2	124.137	Guaiacol
BIOMOL	DL-276	C(=N)(N(C)C)NC(=N)N	C4H11N5	129.164	Metformin
BIOMOL	GR-100	C1(\C=C\C(\C)=C\C=C\C(\C)=C\C(=O)O)=C(C)CCCC1(C)C	C20H28O2	300.435	Retinoic acid
BIOMOL	DL-277	c(c(Cl)c(Cl)cc1)(CN(C2=N3)CC(=O)N2)c13	C10H7Cl2N3O	256.088	Anagrelide
BIOMOL	GR-210	c(c(C)c(C)c(O)c1C)(OC(C)(COc2ccc(CC3SC(=O)NC3=O)cc2)CC4)c14	C24H27NO5S	441.54	Troglitazone
BIOMOL	AC-121	N(CC1=NCCN1)(c2ccc(C)cc2)c3cccc(O)c3	C17H19N3O	281.352	PhentolamineHCl
BIOMOL	AC-760	c1(C(=O)NC[C@@H]2CCCN2CC)cc(S(=O)(=O)N)ccc1OC	C15H23N3O4S	341.426	Sulpiride s (-)
BIOMOL	AC-122	c1(cc(OC)cc2)c2nccc1[C@@H](O)[C@]3([H])C[C@]([H])(CCN34)[C@@H](C=C)C4	C20H24N2O2	324.417	Quinine
BIOMOL	AC-763	C(OCCN(C)C)(c1ccccc1)c2ccccc2	C17H21NO	255.355	Diphenhydramine HCl

BIOMOL	AC-123	c1(Nc2cccc(C(F)(F)F)c2)ccccc1C(=O)O	C14H10F3NO2	281.23	Flufenamic acid
BIOMOL	AC-765	N1(CC(C)N(C)C)c2c(cccc2)Sc3c1cccc3	C17H20N2S	284.419	Promethazine HCl
BIOMOL	AC-124	c1(OCC(O)CNCCC)ccccc1C(=O)CCc2ccccc2	C21H27NO3	341.444	Propafenone
BIOMOL	AC-766	c1(CN(C)C)ccc(CSCCN\C(\NC)=C/[N+]([O-])=O)o1	C13H22N4O3S	314.404	Ranitidine HCl
BIOMOL	AC-125	C1(C(=O)NC(=O)N1)(c2ccccc2)c3ccccc3	C15H12N2O2	252.268	Phenytoin
BIOMOL	AC-770	[C@](CCN1CC2CC2)([C@@H]3c(c4C5)[nH]c(c46)cccc6)([C@]5(O)[C@H]1C7)c(c7ccc8O)c8O3	C26H26N2O3	414.496	Naltrindole HCl
BIOMOL	AC-127	c1(ccc(N)cc1)C(=O)NCCN(CC)CC	C13H21N3O	235.325	Procainamide
BIOMOL	AC-806	C1(COc2c(cccc2)O1)C3=NCCN3	C11H12N2O2	204.225	Idazoxan
BIOMOL	AC-128	c1(c(C)cccc1C)NC(=O)CN(CC)CC	C14H22N2O	234.337	Lidocaine
BIOMOL	AC-807	C12(CCN(CCCC(=O)c3ccc(F)cc3)CC1)C(=O)NCN2c4ccccc4	C23H26FN3O2	395.47	Spiperone
BIOMOL	AC-130	c1(C(=O)NCC2NCCCC2)cc(OCC(F)(F)F)ccc1OCC(F)(F)F	C17H20F6N2O3	414.343	Flecainide
BIOMOL	AC-808	c1([C@@H](O)CN)ccc(O)c(O)c1	C8H11NO3	169.178	Epinephrine-(+)-tartrate I (-)
BIOMOL	AC-131	c1(nc(Cl)c(N)nc1N)C(=O)NC(=N)Nc2ccccc2	C12H12ClN7O	305.723	Phenamil
BIOMOL	AC-809	c1(c(C)cccc1C)NC(SCCC2)=N2	C12H16N2S	220.334	Xylazine HCl
BIOMOL	AC-136	c1(ccccc1OCC2=NCCN2)C3CC3	C13H16N2O	216.279	Cirazoline HCl
BIOMOL	AC-810	c1(ccc(O)c(O)c1)[C@@H](O)CN.[C@H](O)(C(=O)O)[C@@H](O)C(=O)O	C12H17NO9	319.265	Norepinephrine-(+)-tartrate I (-)
BIOMOL	DL-278	c1(NS(=O)(=O)C)ccc(CCN(C)CCOc2ccc(NS(=O)(=O)C)cc2)cc1	C19H27N3O5S2	441.565	Dofetilide
BIOMOL	GR-211	c1(ccc(Cl)cc1)C(=O)NCCc2ccc(OC(C)(C)C(=O)O)cc2	C19H20ClNO4	361.819	Bezafibrate
BIOMOL	DL-279	c(c(Nc1cccc(C#C)c1)ncn2)(cc(OCCOC)c(OCCOC)c3)c23	C22H23N3O4	393.436	Erlotinib
BIOMOL	GR243	c1(C(=O)c2ccc(OCCN3CCCCC3)cc2)c4c(cc(O)cc4)sc1c5ccc(O)cc5	C28H27NO4S	473.583	Raloxifene HCl
BIOMOL	AC-227	[C@H]1(C[C@H](O)c2ccccc2)CCC[C@@H](CC(=O)c3ccccc3)N1C	C22H27NO2	337.455	Lobeline
BIOMOL	GR-301	C([C@@](O)(CC)C(=O)OC1)(C=C(N2C3=O)c4c(cc(c5n4)cccc5)C2)=C13	C20H16N2O4	348.352	Camptothecin
BIOMOL	NS-520	c(cc(OC)cc1)(c(CCNC(=O)C)c[nH]2)c12	C13H16N2O2	232.278	Melatonin
BIOMOL	GR-305	c(c(O)c(c1c2)c(O)c(C)c(O[C@@H]3O[C@H](C)[C@H](O)[C@H](O[C@@H]4O[C@H](C)[C@@H](O)[C@H](O)C4)C3)c1)(C(=O)[C@@H](O[C@@H]5O[C@H](C)[C@@H](O)[C@H](O[C@@H]6O[C@H](C)[C@@H](O)[C@H](O[C@@H]7O[C@H](C)[C@@H](O)	C52H76O24	1085.145	Plicamycin
BIOMOL	PG-008	[C@H]1(C\C=C/CCCC(=O)O)[C@@H](O)C[C@@H](O)[C@@H]1\C=C\[C@@H](O)CCCCC	C20H34O5	354.481	Dinoprost
BIOMOL	GR-306	c(C(=O)[C@@]1(C)O\C=C\[C@H](OC)[C@@H](C)[C@@H](OC(C)=O)[C@H](C)[C@H](O)[C@H](C)[C@@H](O)[C@@H](C)\C=C\C=C(\C)/C(=O)N2)(c3O1)c(O)c(\C=N\N4CCN(C)CC4)c2c5O)c5c(O)c3C	C43H58N4O12	822.94	Rifampicin
BIOMOL	EI-233	c1(ccnc2)c2cccc1S(=O)(=O)N3CCNCCC3	C14H17N3O2S	291.369	Fasudil
BIOMOL	GR-307	[C@H]1(c2cc(OC)c(O)c(OC)c2)[C@]3([H])[C@]([H])(COC3=O)[C@H](O[C@H]4[C@H](O)[C@@H](O)[C@H]5[C@@H](CO[C@@H](C)O5)O4)c6c1cc(c7c6)OCO7	C29H32O13	588.557	Etoposide
BIOMOL	C-106	c(C(O)CCC1)(c(N)c(c2n3)cccc2)c13	C13H14N2O	214.263	Hydroxytacrine maleate
BIOMOL	GR-311	[C@@](OC)([C@]1([H])[C@@]([H])(N1)C2)([C@H](COC(=O)N)C(=C34)C(=O)C(N)=C(C)C3=O)N24	C15H18N4O5	334.327	Mitomycin c
BIOMOL	C-109	N1(CC#CCN2CCCC2)CCCC1=O	C12H18N2O	206.284	Oxotremorine sesquifumarate
BIOMOL	GR-312	n1([C@@H]2O[C@H](CO)[C@@H](NC(=O)[C@@H](N)Cc3ccc(OC)cc3)[C@H]2O)cnc4c1ncnc4N(C)C	C22H29N7O5	471.51	Puromycin 2HCl
BIOMOL	C-110	c(cccc1)(nc(c2c3N)CCCC2)c13	C13H14N2	198.264	Tacrine HCl
BIOMOL	GR-314	c1(ncccc1NC(C)C)N2CCN(C(=O)c3[nH]c(c4c3)ccc(NS(=O)(=O)C)c4)CC2	C22H28N6O3S	456.561	Delavirdine mesylate
BIOMOL	C-112	c1(OCC[N+](CC)(CC)CC)c(OCC[N+](CC)(CC)CC)cccc1OCC[N+](CC)(CC)CC	C30H60N3O3	510.816	Gallamine triethiodide
BIOMOL	GR-316	[illegible] 13	C20H16N2O5	364.351	10-hydroxycamptothecin
BIOMOL	AC-138	c1(CC2=NCCN2)c(C)cc(C(C)(C)C)c(O)c1C	C16H24N2O	260.375	Oxymetazoline HCl
BIOMOL	DL-557	C1(N2CCN(CCOCCO)CC2)=Nc3c(cccc3)Sc4c1cccc4.C5(N6CCN(CCOCCO)CC6)=Nc7c(cccc7)Sc8c5cccc8.C(/C(=O)O)=C\C(=O)O	C46H54N6O8S2	883.086	Quetiapine fumarate
BIOMOL	AC-142	C1(CCN(C(C)C(O)c2ccc(O)cc2)CC1)Cc3ccccc3	C21H27NO2	325.445	Ifenprodil
BIOMOL	AC-812	c1(OCC(O)CNC(C)C)ccccc1CC=C	C15H23NO2	249.349	Alprenolol HCl
BIOMOL	AC-144	c1(cccc2)c2cccc1OCC(O)CN3CCN(c4ccccc4OC)CC3	C24H28N2O3	392.491	Naftopidil 2HCl
BIOMOL	AC-813	N1(CCCN(C)C)c2c(cccc2)CCc3c1cccc3	C19H24N2	280.407	Imipramine HCl
BIOMOL	AC-146	c(cc(OC)c(OC)c1)(c(N)nc(N2CCN(C(=O)c3occc3)CC2)n4)c14	C19H21N5O4	383.401	Prazosin HCl
BIOMOL	AC-814	C1(N2CCNCC2)=Nc3c(cccc3)Oc4c1cc(Cl)cc4	C17H16ClN3O	313.781	Amoxapine
BIOMOL	AC-152	c1(c(Cl)cccc1Cl)NC2=NCCN2	C9H9Cl2N3	230.094	Clonidine HCl

BIOMOL	AC-834	c(c1n(C)c2)(c2C[C@]3([H])[C@]4([H])C[C@H](NS(=O)(=O)N(C)C)CN3C)c4ccc1	C18H26N4O2S	362.49	Mesulergine HCl
BIOMOL	AC-153	c1(\C=N\NC(=N)N)c(Cl)cccc1Cl	C8H8Cl2N4	231.082	Guanabenz acetate
BIOMOL	DL-558	c1(cccc(Cl)c1)C(=O)C(C)NC(C)(C)C	C13H18ClNO	239.741	Amfebutamone
BIOMOL	AC-154	C(NC(OCC1)=N1)(C2CC2)C3CC3	C10H16N2O	180.247	Rilmenidine hemifumarate
BIOMOL	AC-840	c1(C(=O)NCCN(CC)CC)cc(Cl)c(N)cc1OC	C14H22ClN3O2	299.796	Metoclopramide HCl
BIOMOL	AC-160	C12(CCN(CC3COc4c(cccc4)O3)CC1)C(=O)NCN2c5ccccc5	C22H25N3O3	379.452	Spiroxatrine
BIOMOL	AC-858	[C@@]1([C@]2(O)[C@@]([H])(C3)N(CC4CC4)CC1)([C@]5([H])c(c6C2)oc(c67)cccc7)c(c38)c(c(O)cc8)O5	C26H25NO4	415.481	Naltriben mesylate
BIOMOL	AC-161	c1([nH]c(c23)cccc2)c3CCN4[C@@]1([H])C[C@@]([H])([C@]5([H])C4)[C@@H](C(=O)OC)[C@@H](O)CC5	C21H26N2O3	354.443	Yohimbine HCl
BIOMOL	DL-124	C(C(C1)(C)C)(\C=C\C(\C)=C\C=C\C(\C)=C\COC(=O)C)=C(C)CC1	C22H32O2	328.488	Vitamin a (acetate)
BIOMOL	AC-164	[C@](O)(O[C@@](C)(NC(=O)[C@H]1CN(C)[C@@]([H])(C2C1)Cc3c(c4[nH]c3)c2ccc4)C5=O)([C@@]6([H])N(CCC6)C(=O)[C@@H]7Cc8ccccc8)N57	C33H37N5O5	583.677	Dihydroergotamine mesylate
BIOMOL	AC-891	[C@@](CCCC1)(c2c(ccc(O)c2)C3)(CCN4\C=C\C)[C@]1([H])[C@@H]34	C19H25NO	283.408	Levallorphan tartrate
BIOMOL	C-115	[C@@](C=C[C@H](O)C1)([C@H]1O2)(CCN(C)C3)c(c3ccc4OC)c24	C17H21NO3	287.354	Galanthamine HBr
BIOMOL	GR-318	c12c(c(O)c3c([C@@H](O[C@H]4C[C@H](N)[C@H](O)[C@H](C)O4)C[C@](O)(C(C)=O)C3)c1O)C(=O)c5c(c(OC)ccc5)C2=O	C27H29NO10	527.52	Daunorubicin HCl
BIOMOL	CA-200	c1(nc(Cl)c(N)nc1N)C(=O)NC(=N)N	C6H8ClN7O	229.627	Amiloride
BIOMOL	GR-319	c(c(O)c1c(C(=O)c2c(cccc2OC)C1=O)c3O)(C[C@@](O)(C(=O)CO)C[C@@H]4O[C@@H]5O[C@@H](C)[C@@H](O)[C@@H](N)C5)c34	C27H29NO11	543.519	Doxorubicin HCl
BIOMOL	CA-202	C1(c2ccccc2Cl)C(C(=O)OC)=C(C)NC(COCCN)=C1C(=O)OCC	C20H25ClN2O5	408.876	Amlodipine
BIOMOL	GR336	c(c1c(C(C)(C)CCC1)cc2)(C(=O)C(=O)c3c4occ3C)c24	C19H18O3	294.344	Tanshinone iia
BIOMOL	CA-205	[C@@H]1(c2ccc(OC)cc2)Sc3c(cccc3)N(CCN(C)C)C(=O)[C@@H]1OC(=O)C	C22H26N2O4S	414.518	Diltiazem
BIOMOL	DL-284	c1(Nc2ccc(OCc3cccc(F)c3)c(Cl)c2)ncnc4c1cc(c5ccc(CNCCS(C)(=O)=O)o5)cc4	C29H26ClFN4O4S	581.058	Lapatinib
BIOMOL	CA-210	C1(C(C(=O)OC)=C(C)NC(C)=C1C(=O)OC)c2ccccc2[N+]([O-])=O	C17H18N2O6	346.335	Nifedipine
BIOMOL	DL-285	c1(C(=O)OC)ccccc1O	C8H8O3	152.147	Methyl salicylate
BIOMOL	CA-211	C1(C(C(=O)OC(C)C)=C(C)NC(C)=C1C(=O)OCCOC)c2cccc([N+]([O-])=O)c2	C21H26N2O7	418.44	Nimodipine
BIOMOL	GR-240	C1(SC(=O)NC1=O)Cc2ccc(OCCc3ncc(CC)cc3)cc2	C19H20N2O3S	356.439	Pioglitazone
BIOMOL	CA-212	C1(c2cccc([N+]([O-])=O)c2)C(C(=O)OC)=C(C)NC(C)=C1C(=O)OCC	C18H20N2O6	360.361	Nitrendipine
BIOMOL	DL-287	[illegible] 5	C27H23N5O4	481.503	Pranlukast
BIOMOL	CA-215	C(C#N)(C(C)C)(CCCN(C)CCc1ccc(OC)c(OC)c1)c2ccc(OC)c(OC)c2	C27H38N2O4	454.602	Verapamil
BIOMOL	DL-288	c1(OC(=O)N(C)CC)cccc([C@H](C)N(C)C)c1	C14H22N2O2	250.337	Rivastigmine
BIOMOL	CA-216	C1(CCN(CCCOC(=O)C2=C(C)NC(C)=C(C(=O)OC)C2c3cccc([N+]([O-])=O)c3)CC1)(c4ccccc4)c5ccccc5	C36H39N3O6	609.711	Niguldipine HCl
BIOMOL	DL-289	C(c1ccc(S(=O)(=O)C)cc1)(COC2=O)=C2c3ccccc3	C17H14O4S	314.356	Rofecoxib
BIOMOL	CA-225	C(c1ccc(F)cc1)(c2ccc(F)cc2)N3CCN(C\C=C\c4ccccc4)CC3	C26H26F2N2	404.495	Flunarizine-2HCl
BIOMOL	DL-236	[C@](O)(O[C@@](C)(NC(=O)[C@H]1CN(C)[C@](C2=C1)([H])Cc3c(c4[nH]c3)c2ccc4)C5=O)([C@@]6([H])N(CCC6)C(=O)[C@@H]7Cc8ccccc8)N57	C33H35N5O5	581.662	Ergotamine D-tartrate
BIOMOL	AC-165	[C@@]1(OC)(C[C@@H](COC(=O)c2cncc(Br)c2)CN3C)[C@@]3([H])Cc4c(c5n(C)c4)c1ccc5	C24H26BrN3O3	484.386	Nicergoline
BIOMOL	AC-893	[C@](CCN1CC2CCC2)([C@@H]3[C@@H](O)CC4)([C@]4(O)[C@H]1C5)c(c5ccc6O)c6O3	C21H27NO4	357.443	Nalbuphine HCl
BIOMOL	DL-564	[C@H]1(CS[C@H](CO)O1)N2C=C(F)C(N)=NC2=O	C8H10FN3O3S	247.247	Emtricitabine
BIOMOL	AC-911	[N+](C)(C)(C)CCOC(=O)N	C6H15N2O2	147.195	Carbamylcholine Cl
BIOMOL	AC-169	c1(OCC(O)CNC(C)C)ccc(CCOCC2CC2)cc1	C18H29NO3	307.428	Betaxolol HCl
BIOMOL	AC-912	C(C(C)OC(=O)N)[N+](C)(C)C	C7H17N2O2	161.222	Carbamyl-beta-methylcholine Cl
BIOMOL	AC-171	c1(OCC(O)CNC(C)C)ccc(NC(=O)C)cc1	C14H22N2O3	266.336	Practolol
BIOMOL	AC-915	C(COC(=O)CCC)[N+](C)(C)C	C9H20NO2	174.261	Butyrylcholine Cl
BIOMOL	AC-172	c1(N2CCOCC2)nsnc1OC[C@@H](O)CNC(C)(C)C	C13H24N4O3S	316.42	Timolol maleate (s)
BIOMOL	AC-925	c1(\N=C(\N)/N)scc(CSCCC(=N)NS(=O)(=O)N)n1	C8H15N7O2S3	337.445	Famotidine
BIOMOL	AC-173	N1(CCOCC1)C(=O)NCCNCC(O)COc2ccc(O)cc2	C16H25N3O5	339.387	Xamoterol hemifumarate
BIOMOL	AC-928	C(O)(c1ccccc1)(c2ccccc2)C3CCN(CCCC(O)c4ccc(C(C)(C)C)cc4)CC3	C32H41NO2	471.673	Terfenadine
BIOMOL	AC-174	C(C)(C)(C)NCC(O)c1cc(Cl)c(N)c(Cl)c1	C12H18Cl2N2O	277.19	Clenbuterol
BIOMOL	AC-986	c1(ccncc1)C(=O)NN	C6H7N3O	137.139	Isoniazid
BIOMOL	AC-175	c1(C=CC(=O)N2)c2c(O)ccc1[C@@H](O)[C@H](CC)NC(C)C	C16H22N2O3	290.357	Procaterol hcl

BIOMOL	AC-993	c(ccs1)(CN(Cc2ccccc2Cl)CC3)c13	C14H14ClNS	263.786	Ticlopidine HCl
BIOMOL	AC-176	c1(C(O)CNC(C)(C)C)ccc(O)c(CO)c1	C13H21NO3	239.311	Salbutamol sulfate
BIOMOL	DL-246	c1(CC(C)CN2C[C@@H](C)O[C@@H](C)C2)ccc(C(C)(C)CC)cc1	C21H35NO	317.509	Amorolfine
BIOMOL	AC-181	c(c(OCC(O)CNC(C)C)ccc1)(cc[nH]2)c12	C14H20N2O2	248.321	Pindolol
BIOMOL	DL-247	[C@@](C)(OCC[C@H]1CCCN1C)(c2ccccc2)c3ccc(Cl)cc3	C21H26ClNO	343.89	Clemastine fumarate
BIOMOL	CA-234	C1(CN)(CCCCC1)CC(O)=O	C9H17NO2	171.237	Gabapentin
BIOMOL	DL-291	c(ccc(F)c1)(\C(=C\c2ccc(S(=O)C)cc2)\C(C)=C3CC(=O)O)c13	C20H17FO3S	356.411	Sulindac
BIOMOL	CA-236	C1(C(C(=O)OC)=C(C)NC(C)=C1C(=O)OCC)c2cccc(Cl)c2Cl	C18H19Cl2NO4	384.254	Felodipine
BIOMOL	DL-292	C(C(=O)O)(CCC)CCC	C8H16O2	144.211	Valproic acid
BIOMOL	CA-237	C1(c2cccc([N+]([O-])=O)c2)C(C(=O)OCCOC)=C(C)NC(C)=C1C(=O)OC\C=C\c3ccccc3	C27H28N2O7	492.52	Cilnidipine
BIOMOL	DL-547	[C@@]([H])(\C(=C\C=C(\C[C@@H](O)C[C@@H]1O)/C1=C)\CCC2)(CC[C@@H]3[C@H](C)\C=C\[C@@H](O)C4CC4)[C@]23C	C27H40O3	412.605	Calcipotriene
BIOMOL	CA-305	c1(ccccc1)CN(CCCl)C(C)COc2ccccc2	C18H22ClNO	303.826	Phenoxybenzamine HCl
BIOMOL	DL-294	c1(cc(NC(=O)OC2CCCC2)cc3)c3n(C)cc1Cc4ccc(C(=O)NS(=O)(=O)c5ccccc5C)cc4OC	C31H33N3O6S	575.675	Zafirlukast
BIOMOL	CA-310	c1(cc(C(F)(F)F)cc2)c2Sc3c(cccc3)N1CCCN4CCN(C)CC4	C21H24F3N3S	407.496	Trifluoperazine
BIOMOL	DL-295	c1(sc(c2c1)cccc2)C(C)N(O)C(=O)N	C11H12N2O2S	236.29	Zileuton
BIOMOL	DL-218	c(n(CC(=O)C)cn1)(C(=O)N(CCCC)C(=O)N2CCCC)c12	C16H24N4O3	320.387	Denbufylline
BIOMOL	PR-118	c1(C(=N)N)ccc(OCCCCCOc2ccc(C(=N)N)cc2)cc1	C19H24N4O2	340.419	Pentamidine
BIOMOL	DL-131	O(CCCCCCCCCCCCCCCC)P([O-])(=O)OCC[N+](C)(C)C	C21H46NO4P	407.568	Miltefosine
BIOMOL	DL-297	C(=O)([C@H](Cc1ccccc1)NC(=O)c2cnccn2)N[C@H](CC(C)C)B(O)O	C19H25BN4O4	384.237	Bortezomib
BIOMOL	DL-281	[C@H]1(C\C=C/CCCC(=O)OC(C)C)[C@@H](O)C[C@@H](O)[C@@H]1CC[C@@H](O)CCc2ccccc2	C26H40O5	432.593	Latanoprost
BIOMOL	DL-161	C(C)(C)(C)NCC(O)c1cc(OC(=O)N(C)C)cc(OC(=O)N(C)C)c1	C18H29N3O5	367.44	Bambuterol
BIOMOL	CM-109	[C@@]1(CO)([C@H](O)C[C@H](O[C@@H]2O[C@@H](C)[C@H](O)[C@@H](O)[C@H]2O)C3)[C@]3(O)CC[C@]4([H])[C@@]1([C@H](O)C[C@@]5(C)[C@]4(O)CC[C@@H]5C6=CC(=O)OC6)[H]	C29H44O12	584.652	Ouabain
BIOMOL	DL-186	c1(c2ccc(Cl)cc2)nc(c3ccccc3)sc1CC(=O)O	C17H12ClNO2S	329.801	Fentiazac
BIOMOL	CM-112	c1(C(=O)NN2[C@H](C)CCC[C@@H]2C)ccc(Cl)c(S(=O)(=O)N)c1	C14H20ClN3O3S	345.845	Clopamide
BIOMOL	J120	C1(CC)(NC(=O)N(C)C1=O)c2ccccc2	C12H14N2O2	218.252	Mephenytoin
BIOMOL	AC-183	c1(C(O)CNC(C)C)ccc(N)c(C#N)c1	C12H17N3O	219.283	Cimaterol
BIOMOL	DL-248	[illegible] O	C23H32N6O4S	488.603	Vardenafil
BIOMOL	AC-185	c1(CCNC(C)CCc2ccc(O)cc2)ccc(O)c(O)c1	C18H23NO3	301.38	Dobutamine HCl
BIOMOL	DL-249	N1(C(=O)O[C@@H](CNC(=O)C)C1)c2ccc(N3CCOCC3)c(F)c2	C16H20FN3O4	337.346	Linezolid
BIOMOL	AC-189	c(cccc1)(cc(C(O)CNC(C)C)cc2)c12	C15H19NO	229.317	Pronethalol HCl
BIOMOL	DL-250	[C@]1(OC(=O)C)(CO2)[C@@]2([H])C[C@H](O)[C@](C)([C@@]13[H])C(=O)[C@H](O)C(=C4C)C(C)(C)[C@@](O)(C[C@@H]4OC(=O)[C@H](O)[C@@H](NC(=O)OC(C)(C)C)c5ccccc5)[C@H]3OC(=O)c6ccccc6	C43H53NO14	807.879	Docetaxil
BIOMOL	AC-192	c1(ccc(NS(=O)(=O)C)cc1)C(O)CNC(C)C	C12H20N2O3S	272.364	Sotalol HCl
BIOMOL	DL-251	c(cc(CC(=O)O)cc1)(c1OC2)\C(=C/CCN(C)C)\c(cccc3)c23	C21H23NO3	337.412	Olopatadine
BIOMOL	AR-111	[C@@](CCCNC)(CC1)(c2c3cccc2)c(cccc4)c4[C@@]13[H]	C20H23N	277.403	Maprotiline HCl
BIOMOL	DL-258	C1(c2cccc([N+]([O-])=O)c2)C(C(=O)OC)=C(C)NC(C)=C1C(=O)OCCN3CCN(C(c4ccccc4)c5ccccc5)CC3	C35H38N4O6	610.699	Manidipine
BIOMOL	AC-194	C(CCNC)(Oc1ccccc1OC)c2ccccc2	C17H21NO2	271.354	Nisoxetine HCl
BIOMOL	DL-252	c1(cc(O)c(O)c([N+]([O-])=O)c1)C(=O)c2ccc(C)cc2	C14H11NO5	273.241	Tolcapone
BIOMOL	DL-553	C1(\C=C\C(\C)=C\C=C\C(\C)=C\C=C\C=C(/C)\C=C\C=C(/C)\C=C\C2=C(C)C(=O)CCC2(C)C)=C(C)C(=O)CCC1(C)C	C40H52O2	564.84	Canthaxanthin
BIOMOL	DL-253	[C@](CC)([C@](O)(C#C)CC1)(C=CC(=C(CCC2=O)C3=C2)[C@@]4([H])CC3)[C@@]14[H]	C21H24O2	308.414	Gestrinone
BIOMOL	AC-214	[C@H]1(Cc2cncn2C)COC(=O)[C@H]1CC	C11H16N2O2	208.257	Pilocarpine HCl
BIOMOL	DL-254	c1(C(=O)OCC(OC(=O)O2)=C2C)c(C(C)(C)O)nc(CCC)n1Cc3ccc(c4ccccc4c5nnn[nH]5)cc3	C29H30N6O6	558.585	Olmesartan
BIOMOL	AC-218	[C@@]([H])(CC1)(C[C@H](OC(=O)C(CO)c2ccccc2)C3)[N+](C)(C(C)C)[C@@]13[H]	C20H30NO3	332.457	Ipratropium Br
BIOMOL	DL-255	C1(C(C(=O)OC)=C(C)NC(C)=C1C(=O)OCC(C)C)c2ccccc2[N+]([O-])=O	C20H24N2O6	388.414	Nisoldipine
BIOMOL	AC-220	N1(C(=O)CN2CCN(C)CC2)c3c(cccc3)C(=O)Nc4c1nccc4	C19H21N5O2	351.402	Pirenzepine 2HCl
BIOMOL	DL-256	C1(C(C(=O)OCC)=C(C)NC(C)=C1C(=O)OCC)c2ccccc2\C=C\C(=O)OC(C)(C)C	C26H33NO6	455.543	Lacidipine
BIOMOL	CN-244	[N+]([N-]1)(=C\C(\O1)=N\C(=O)OCC)N2CCOCC2	C9H14N4O4	242.232	Molsidomine

BIOMOL	KC-115	c(ccc(Cl)c1)(N=C(C)NS2(=O)=O)c12	C8H7ClN2O2S	230.671	Diazoxide
BIOMOL	CR110	c1(cccc2)c2n(CCN3CCOCC3)c(C)c1C(=O)c4ccc(OC)cc4	C23H26N2O3	378.464	Pravadoline
BIOMOL	KC-120	c1(C(=O)NCCc2ccc(S(=O)(=O)NC(=O)NC3CCCCC3)cc2)cc(Cl)ccc1OC	C23H28ClN3O5S	494.004	Glyburide
BIOMOL	CT110	c1(NC(=O)\C=C\c2ccc(OC)c(OC)c2)ccccc1C(=O)O	C18H17NO5	327.331	Tranilast
BIOMOL	KC-125	C1(=NC(=N)N(O)C(N)=C1)N2CCCCC2	C9H15N5O	209.248	Minoxidil
BIOMOL	CT115	c(c(O)ccc1O)(C(=O)C([C@H](O)C\C=C(\C)/C)=CC2=O)c12	C16H16O5	288.295	Shikonin
BIOMOL	KC-135	S(=O)(=O)(NC(=O)NN1CCCCCC1)c2ccc(C)cc2	C14H21N3O3S	311.4	Tolazamide
BIOMOL	D-102	N(C(=O)[C@](C(C)C)(NC(=O)[C@H]1CN(C)C(C2=C1)Cc3c(c4[nH]c3Br)c2ccc4)O5)([C@@H](CC(C)C)C(=O)N6[C@@]7([H])CCC6)[C@@]57O	C32H40BrN5O5	654.595	Bromocriptine mesylate
BIOMOL	KC-152	c1(cnccc1)C(=O)NCCO[N+]([O-])=O	C8H9N3O4	211.175	Nicorandil
BIOMOL	D-107	C1(N2CCN(C)CC2)=Nc3c(ccc(Cl)c3)Nc4c1cccc4	C18H19ClN4	326.823	Clozapine
BIOMOL	DL-298	c(cc(C(=C)c1ccc(C(=O)O)cc1)c(C)c2)(C(C)(C)CCC3(C)C)c23	C24H28O2	348.478	Bexarotene
BIOMOL	D-108	C1(N2CCN(C)CC2)=Nc3c(cccc3)Sc4c1cc(Cl)cc4	C18H18ClN3S	343.874	Clothiapine
BIOMOL	DL-221	c1(ccc(OC)cc1)C(=O)\C(\Br)=C/C(=O)O	C11H9BrO4	285.091	Bromebric acid
BIOMOL	D-109	[C@@]([H])(N(CCC)CCC1)(Cc(c2C3)c[nH]n2)[C@]13[H]	C13H21N3	219.326	Quinpirole HCl (-)-
BIOMOL	DL-299	n1(c2ccc(S(=O)(=O)N)cc2)nc(C(F)(F)F)cc1c3ccc(C)cc3	C17H14F3N3O2S	381.372	Celecoxib
BIOMOL	D-111	c1(C(=O)NC[C@@H]2CCCN2CC)c(OC)ccc(Br)c1OC	C16H23BrN2O3	371.269	Remoxipride
BIOMOL	DL-172	N1(CCCC1=O)[C@@H](CC)C(=O)N	C8H14N2O2	170.209	Levetiracetam
BIOMOL	DM100	[C@](C)([C@@H]([C@H](C)CCCC(O)(C)C)CC1)(CC2)[C@]1([H])\C(\C2)=C\C=C(\C[C@@H](O)CC3)/C3=C	C27H44O2	400.637	Calcifediol
BIOMOL	DL-300	C(n1ncnc1)(c2ccc(C#N)cc2)c3ccc(C#N)cc3	C17H11N5	285.303	Letrozole
BIOMOL	AC-221	N1(C(=O)CN2CCN(C)CC2)c3c(cccc3)NC(=O)c4c1c(C)sc4	C19H22N4O2S	370.469	Telenzepine 2HCl
BIOMOL	DL-257	C1(N2CCN(C)CC2)=Nc3c(cccc3)Nc4c1cc(C)s4	C17H20N4S	312.432	Olanzapine
BIOMOL	AC-222	C(CO)(C(=O)N(CC)Cc1ccncc1)c2ccccc2	C17H20N2O2	284.353	Tropicamide
BIOMOL	G-226	[C@]1([H])([C@@H](CC[C@H]2OC(=O)C[C@H](O)C2)[C@@H](C)C=C3)C3=C[C@H](C)C[C@@H]1OC(=O)[C@@H](C)CC	C24H36O5	404.54	Lovastatin
BIOMOL	AC-232	[C@]1([H])(C[C@H]([N+]2(C)CCCCC2)[C@@H]3OC(=O)C)[C@]3(C)CC[C@@]4([H])[C@@]1([H])CC[C@]5([H])[C@]4(C)C[C@H]([N+]6(C)CCCC6)[C@@H](OC(=O)C)C5	C35H60N2O4	572.862	Pancuronium Br
BIOMOL	NA-137	c1(nnc(N)nc1N)c2cccc(Cl)c2Cl	C9H7Cl2N5	256.091	Lamotrigine
BIOMOL	AC-238	[C@@](O)([C@@H]1C(=O)O[C@H]2C[C@H](O[C@@]3(CC[C@H](C)[C@@]([H])([C@@H](C)CC)O3)C2)C\C=C(/C)\[C@H]4O[C@@H]5O[C@@H](C)[C@H](O[C@@H]6O[C@@H](C)[C@H](O)[C@@H](OC)C6)[C@@H](OC)C5)(\C(=C\C=C\[C@@H]4C)\CO7)[	C48H74O14	875.093	Ivermectin
BIOMOL	DL-260	c1(ccc(O)cc1)C(O)CNC(C)CCc2ccc(O)cc2	C18H23NO3	301.38	Ractopamine
BIOMOL	AC-241	[C@]1(C)(CCN2C)[C@@]2([H])N(C)c3c1cc(OC(=O)NC)cc3	C15H21N3O2	275.346	Physostigmine sulfate
BIOMOL	DL-261	c1(cc(S(=O)(=O)N2CCN(C)CC2)ccc1OCC)C(NC(=O)c3c4c(CCC)nn3C)=N4	C22H30N6O4S	474.576	Sildenafil
BIOMOL	DL-554	c1(ccncc1)C(=O)NNCCC(=O)NCc2ccccc2	C16H18N4O2	298.34	Nialamide
BIOMOL	DL-262	[C@H]1(CC[C@@H](c2ccc(Cl)cc2)CC1)C3=C(O)C(=O)c4c(cccc4)C3=O	C22H19ClO3	366.837	Atovaquone
BIOMOL	AC-250	c1(ccc(Cl)cc1)C2(O)CCN(CCCC(=O)c3ccc(F)cc3)CC2	C21H23ClFNO2	375.864	Haloperidol HCl
BIOMOL	DL-208	c(cccc1Cl)(c(COC(Cn2cncc2)c3ccc(Cl)cc3Cl)cs4)c14	C20H15Cl3N2OS	437.77	Sertaconazole
BIOMOL	AC-259	c(OCO1)(cc(CN2CCN(c3ncccn3)CC2)cc4)c14	C16H18N4O2	298.34	Piribedil HCl
BIOMOL	DL-191	N(C(C([O-])=O)=C(C[N+]1(C)CCCC1)CS2)(C(=O)[C@H]3NC(=O)\C(=N/OC)\c4nc(N)sc4)[C@H]23	C19H24N6O5S2	480.561	Cefepime
BIOMOL	AC-277	N(CCN(C)C)(Cc1ccc(OC)cc1)c2ncccc2	C17H23N3O	285.384	Mepyramine maleate
BIOMOL	DL-178	c(NC(=O)CC1)(cc(OCCCCN2CCN(c3cccc(Cl)c3Cl)CC2)cc4)c14	C23H27Cl2N3O2	448.385	Aripiprazole
BIOMOL	AC-279	C(=C\CN1CCCC1)(/c2ncccc2)\c3ccc(C)cc3	C19H22N2	278.391	Trans-triprolidine HCl
BIOMOL	DL-264	c1(c(C(=O)O)ccc2)c2nc(OCC)n1Cc3ccc(c4ccccc4c5nnn[nH]5)cc3	C24H20N6O3	440.454	Candesartan
BIOMOL	DM200	[C@@]1([H])(\C(\C2)=C\C=C(\C[C@@H](O)C[C@@H]3O)/C3=C)[C@@](C)([C@@H]([C@H](C)CCCC(C)(C)O)CC1)CC2	C27H44O3	416.636	Calcitriol
BIOMOL	DL-301	[C@H]1([C@H](C[C@H](O)O[C@H]1C)OC)O.C([C@]2(O3)C[C@@]([H])(OC(=O)[C@]([H])(C=C(C)[C@H]4O)[C@](O)(\C(\CO5)=C\C=C\[C@H](C)[C@@H](\C(=C\C6)\C)O[C@@H](O[C@@H]7C)C[C@H]([C@@H]7C)OC)[C@@]45[H])C[C@]36[H])=C	C47H71O14R	860.058	Abamectin
BIOMOL	EI-107	[C@]1(Cn2cncc2)(OC[C@H](COc3ccc(N4CCN(C(=O)C)CC4)cc3)O1)c5ccc(Cl)cc5Cl	C26H28Cl2N4O4	531.431	Ketoconazole
BIOMOL	DL-222	[C@](C)([C@@H]([C@H](C)CCCC(C)C)CC1)(CC2)[C@]1([H])\C(\C2)=C/C=C(\C[C@@H](O)C[C@@H]3O)/C3=C	C27H44O2	400.637	Alfacalcidol
BIOMOL	EI-114	C(c1ccccc1)(c2ccccc2)N3CCN(CCCN4C(=O)Nc5c4cccc5)CC3	C27H30N4O	426.553	Oxatomide

BIOMOL	DL-151	C1(=CC(=S)SS1)c2ccc(OC)cc2	C10H8OS3	240.365	Anethole-trithione (anetholtrithion)
BIOMOL	EI-121	c(c(OCC(O)COc1cccc2c1C(=O)C=C(C([O-])=O)O2)ccc3)(C(=O)C=C(C([O-])=O)O4)c34.[Na+].[Na+]	C23H14Na2O11	512.33	Disodium cromoglycate
BIOMOL	DL-144	c1(C(C)(C)C#N)cc(Cn2ncnc2)cc(C(C)(C)C#N)c1	C17H19N5	293.366	Anastrozole
BIOMOL	EI-125	c1(CNC(=O)CCCC\C=C\C(C)C)ccc(O)c(OC)c1	C18H27NO3	305.412	Capsaicin
BIOMOL	DL-162	c1(C(F)(F)F)cc(NC(=O)C(O)(C)CS(=O)(=O)c2ccc(F)cc2)ccc1C#N	C18H14F4N2O4S	430.373	Bicalutamide
BIOMOL	EI-126	C(C(=O)C=C([C@@]12C)CC[C@]3([H])[C@]1(F)[C@@H](O)C[C@]([C@]34[H])(C)[C@@](O)(C(CO)=O)[C@H](C)C4)=C2	C22H29FO5	392.461	Dexamethasone
BIOMOL	DL-180	C(Cl)(Cl)(P(=O)(O)O)P(=O)(O)O	CH4Cl2O6P2	244.892	Clodronate disodium
BIOMOL	EI-127	c(c(N1CCCCC1)nc(N(CCO)CCO)n2)(nc(N(CCO)CCO)nc3N4CCCCC4)c23	C24H40N8O4	504.626	Dipyridamole
BIOMOL	DL-102	[C@H]([C@H](C)Cl)(NC(=O)[C@@H]1C[C@@H](CCC)CN1C)[C@@H]2[C@H](O)[C@H](O)[C@@H](O)[C@@H](SC)O2	C18H33ClN2O5S	424.983	Clindamycin palmitate
BIOMOL	EI-128	c1(C(=O)C(=C)CC)ccc(OCC(=O)O)c(Cl)c1Cl	C13H12Cl2O4	303.138	Ethacrynic acid
BIOMOL	DL-164	c1(cc(Cl)c(C(C#N)c2ccc(Cl)cc2)c(Cl)c1)N3N=CC(=O)NC3=O	C17H9Cl3N4O2	407.638	Diclazuril
BIOMOL	EI-131	c(ccc(OC)c1)(n(C(=O)c2ccc(Cl)cc2)c(C)c3CC(=O)O)c13	C19H16ClNO4	357.788	Indomethacin
BIOMOL	DL-302	n1([C@H]2CC[C@@H](CO)O2)cnc3c1N=CNC3=O	C10H12N4O3	236.227	Didanosine
BIOMOL	EI-133	c(cc([C@H](C)C(=O)O)cc1)(ccc(OC)c2)c12	C14H14O3	230.259	Naproxen
BIOMOL	DL-199	[C@@]([H])(C[C@@]([H])(OC(=O)c1c[nH]c2c1cccc2)C3)(C4)N(CC5=O)[C@]3([H])C[C@]45[H]	C19H20N2O3	324.374	Dolasetron
BIOMOL	AC-280	c1(CSCC\N=C(\NC)/NC#N)nc[nH]c1C	C10H16N6S	252.339	Cimetidine
BIOMOL	DL-101	c1(cccc2)c2cccc1CN(C)Cc3ccc(C(C)(C)C)cc3	C23H27N	317.467	Butenafine
BIOMOL	AC-282	c1(CSCCN\C(\NC)=N\C#N)csc(NC(=N)N)n1	C10H16N8S2	312.418	Tiotidine
BIOMOL	DL-200	c(sc(S(=O)(=O)N)c1)(S(=O)(=O)[C@@H](C)C[C@@H]2NCC)c12	C10H16N2O4S3	324.44	Dorzolamide
BIOMOL	DL-555	c1c2c(ccc1)c(CS(=O)(=O)N)no2	C8H8N2O3S	212.226	Zonisamide
BIOMOL	DL-212	[C@]1(OCc(c12)cc(C#N)cc2)(CCCN(C)C)c3ccc(F)cc3	C20H21FN2O	324.392	Escitalopram
BIOMOL	DL-556	C(O)(Cn1cncc1)(P(=O)(O)O)P(=O)(O)O	C5H10N2O7P2	272.09	Zoledronic acid
BIOMOL	DL-209	n1(Cc2ccc(C(=O)O)cc2)c(CCCC)ncc1\C=C(\C(=O)O)/Cc3sccc3	C23H24N2O4S	424.513	Eprosartan
BIOMOL	AC-322	[C@](CCN1CC2CC2)([C@@H]3C(=O)CC4)([C@]4(O)[C@H]1C5)c(c5ccc6O)c6O3	C20H23NO4	341.401	Naltrexone HCl
BIOMOL	DL-119	c1(CC([O-])=O)ccccc1.[Na+]	C8H7NaO2	158.13	Sodium phenylacetate
BIOMOL	DL-560	[nH]1c(cc2)c(cc2C[C@@H]3NC(=C)OC3)c(CCN(C)C)c1.O	C17H25N3O2	303.399	zolmitriptan
BIOMOL	DL-551	c1(ccc(\C=C\C(=O)O)cc1)Cn2cncc2	C13H12N2O2	228.247	Ozagrel
BIOMOL	AC-408	[C@]1(C)(C[C@]([H])(C[C@]2(C)C1)C[C@]3(N)C2)C3	C12H21N	179.302	Memantine HCl
BIOMOL	DL-217	c1(\C=C(/C#N)\C(=O)N(CC)CC)cc(O)c(O)c([N+]([O-])=O)c1	C14H15N3O5	305.286	Entacapone
BIOMOL	AC-439	N1(CCCC1=O)C(=O)c2ccc(OC)cc2	C12H13NO3	219.237	Aniracetam
BIOMOL	AP-302	[C@@H](c1nc[nH]c1)(O[C@@H]2O[C@@H](CO)[C@@H](O)[C@H](O)[C@@H]2O[C@@H]3O[C@H](CO)[C@@H](O)[C@@H](OC(=O)N)[C@@H]3O)[C@H](C(=O)N[C@H](C)[C@@H](O)[C@H](C)C(=O)N[C@H]([C@H](O)C)C(=O)NCCc4scc(c5ncc(C(=O)NCC	C55H84N17O21S3	1415.552	Bleomycin sulfate
BIOMOL	AC-501	C(F)(F)(F)Oc1ccc2c(sc(N)n2)c1	C8H5F3N2OS	234.198	Riluzole HCl
BIOMOL	AR-100	c(c(OCC(CNC(C)(C)C)OC(=O)c1ccccc1)ccc2)(cc(C)[nH]3)c23	C23H28N2O3	380.48	Bopindolol malonate
BIOMOL	AC-508	c1(C(C)C)cccc(C(C)C)c1O	C12H18O	178.271	Propofol
BIOMOL	AR-102	c1(c(Cl)cccc1Cl)CC(=O)NC(=N)N.Cl	C9H10Cl3N3O	282.554	Guanfacine HCl
BIOMOL	EI-160	c1(ccc(Cl)cc1)OC(C)(C)C(=O)OCC	C12H15ClO3	242.699	Clofibrate
BIOMOL	DL-192	N1(C(=O)[C@H](C)N[C@H](C(O)=O)CCc2ccccc2)CCC[C@H]1C(=O)O	C18H24N2O5	348.394	Enalaprilat
BIOMOL	EI-164	c1(ccc(CC(C)C)cc1)C(C)C(=O)O	C13H18O2	206.281	Ibuprofen
BIOMOL	DL-108	c1(c2ccc(F)cc2)c(\C=C\[C@@H](O)C[C@@H](O)CC(O)=O)n(C(C)C)c3c1cccc3	C24H26FNO4	411.466	Fluvastatin Na
BIOMOL	EI-165	c1(CNNC(=O)C(N)CO)ccc(O)c(O)c1O	C10H15N3O5	257.243	Benserazide HCl
BIOMOL	DL-155	[C@@H]1(C2CCCCC2)C[C@@H](C(=O)O)N(C(=O)CP(=O)(CCCCc3ccccc3)OC(C(C)C)OC(=O)CC)C1	C30H46NO7P	563.663	Fosinopril
BIOMOL	EI-166	c(cccc1)(CN(C(=N)N)CC2)c12	C10H13N3	175.23	Debrisoquin sulfate
BIOMOL	DL-215	[C@H]1(O[C@H](CO)[C@@H](O)C1(F)F)N2C=CC(N)=NC2=O	C9H11F2N3O4	263.198	Gemcitabine HCl
BIOMOL	EI-167	C1(N2CCN(C)CC2)Cc3c(cccc3)Sc4c1cc(SC)cc4	C20H24N2S2	356.548	Methiothepin maleate
BIOMOL	DL-243	[C@@]12(O3)[C@]([C@@H]4OC1=O)([C@@]([C@@H](O)C(=O)O5)([C@]35[H])[C@H](C(C)(C)C)C4)C[C@@]6([H])[C@@]2([C@H](C)C(=O)O6)	C20H24O9	408.399	Ginkgolide a
BIOMOL	EI-168	c1(Oc2ccccc2)c(S(=O)(=O)N)cc(C(=O)O)cc1NCCCC	C17H20N2O5S	364.416	Bumetanide
BIOMOL	DL-166	c1(cccc2)c2n(C)nc1C(=O)N[C@H]3C[C@]4([H])N(C)[C@@]([H])(CCC4)C3	C18H24N4O	312.409	Granisetron

BIOMOL	EI-180	[C@@H]1(O[C@@H]2[C@H](N)[C@@H](O)[C@H](O)[C@@H](CN)O2)[C@@H](N)C[C@@H](N)[C@H](O)[C@H]1O[C@@H]3O[C@H](CO)[C@@H](O[C@@H]4[C@H](N)[C@@H](O)[C@H](O)[C@H](CN)O4)[C@H]3	C23H46N6O13	614.644	Neomycin sulfate
BIOMOL	DL-105	C1(=O)O[Pt]OC1.N.N	C2H8N2O3Pt	303.175	Nedaplatin
BIOMOL	EI-206	[C@H]1(OC(=O)C)[C@H](OC(=O)C)[C@@H](COC(=O)C)O[C@@H](S[Au]=P(CC)(CC)CC)[C@@H]1OC(=O)C	C20H34AuO9PS	678.484	Auranofin
BIOMOL	DL-303	C1(=O)O[Pt]OC1=O.[C@]2([H])(N)CCCC[C@@]2([H])N	C8H14N2O4Pt	397.286	Oxaliplatin
BIOMOL	EI-213	N1(C(=O)[C@H](C)CS)CCC[C@H]1C(=O)O	C9H15NO3S	217.285	Captopril
BIOMOL	DL-174	c1(\C=C\C)ccc(OC)cc1	C10H12O	148.202	Anethole, trans-
BIOMOL	EI-216	C(CCCCC#CCCCC#CCO)(C(=O)C(C)=C(C)C1=O)=C1C	C21H26O3	326.429	Docebenone
BIOMOL	DL-546	[C@@H](C(C)(C)C)(NC(OC)=O)C(=O)NN(Cc1ccc(c2ccccn2)cc1)C[C@H](O)[C@H](Cc3ccccc3)NC(=O)[C@H](C(C)(C)C)NC(OC)=O	C38H52N6O7	704.855	Atazanavir
BIOMOL	DL-545	c(COC1=O)(c(C)c(OC)c(C\C=C(/C)\CCC(=O)OCCN2CCOCC2)c3O)c13	C23H31NO7	433.495	Mycophenolate mofetil
BIOMOL	DL-363	[C@]1(C(=O)CCl)(OC(=O)CC)[C@@H](C)C[C@]2([H])[C@]1(C)C[C@H](O)[C@@]3(F)[C@@]2([H])CCC(=C4)[C@]3(C)C=CC4=O	C25H32ClFO5	466.97	Clobetasol propionate
BIOMOL	DL-544	c1(ccc(c2cc(Cl)cnc2c3ccc(C)nc3)cc1)S(C)(=O)=O	C18H15ClN2O2S	358.842	Etoricoxib
BIOMOL	DL-364	c1(cc(C)c(NC(=O)c2cc(I)cc(I)c2O)cc1Cl)C(C#N)c3ccc(Cl)cc3	C22H14Cl2I2N2O2	663.074	Closantel
BIOMOL	DL-103	c1(nc(Cl)nc2N)c2ncn1[C@H]3[C@@H](F)[C@H](O)[C@@H](CO)O3	C10H11ClFN5O3	303.677	Clofarabine
BIOMOL	DL-365	[C@]([H])([C@]1(C)C(=CC(=O)CC1)CC2)([C@@H](O)C[C@@]3(C)[C@@]4([H])CC[C@@H]3C(=O)CO)[C@@]24[H]	C21H30O4	346.461	Corticosterone
BIOMOL	DL-219	[C@@]1([H])(C[C@@H](C(=O)N(CCCN(C)C)C(=O)NCC)CN2CC=C)[C@@]2([H])Cc3c(c4[nH]c3)c1ccc4	C26H37N5O2	451.604	Cabergoline
BIOMOL	DL-366	c1(ccccc1C)N(CC)C(=O)\C=C\C	C13H17NO	203.28	Crotamiton
BIOMOL	DL-207	c1(C(=O)OCCCN2CCCN(CCCOC(=O)c3cc(OC)c(OC)c(OC)c3)CC2)cc(OC)c(OC)c(OC)c1	C31H44N2O10	604.688	Dilazep
BIOMOL	DL-367	P1(=O)(OCCCN1)N(CCCl)CCCl	C7H15Cl2N2O2P	261.086	Cyclophosphamide monohydrate
BIOMOL	DL-213	C(O)(P(=O)(O)O)(P(=O)(O)O)CCN(C)CCCCC	C9H23NO7P2	319.229	Ibandronate
BIOMOL	DL-368	C1(=C2)[C@](C)([C@@H]3[C@@H](C3)C2=O)[C@]4([H])[C@]([H])([C@]([C@]5(C)CC4)([H])CC[C@@]5(C(C)=O)OC(C)=O)C=C1Cl	C24H29ClO4	416.938	Cyproterone acetate
BIOMOL	DL-202	N(C(C(=O)O)=C(SCCNC=N)C1)(C(=O)[C@]2([H])[C@]([H])(O)C)[C@]12[H]	C12H17N3O4S	299.346	Imipenem
BIOMOL	DL-369	[C@H](O[C@H](CO)[C@H]1O)(N2C(=NC(=N)C=C2)O3)[C@H]13	C9H11N3O4	225.201	Cyclocytidine HCl
BIOMOL	DL-156	C1(CCCCC1)NC(=O)N(N=O)CCCl	C9H16ClN3O2	233.695	Lomustine
BIOMOL	DL-370	[C@@H]1([C@@H](O)[C@H](O)[C@@H](CO)O1)N2C=CC(N)=NC2=O	C9H13N3O5	243.217	Cytarabine
BIOMOL	DL-543	[C@]1([H])([C@]2([H])C[C@@H](O)[C@@H]1\C=C\[C@@H](O)C(C)CC#CC)C\C(=C\CCCC(=O)O)\C2	C22H32O4	360.487	Iloprost
BIOMOL	DL-371	c1(N=NN(C)C)[nH]cnc1C(=O)N	C6H10N6O	182.183	Dacarbazine
BIOMOL	DL-244	C(c1ccc(F)cc1)(c2ccc(F)cc2)N3CCN(Cc4ccc(OC)c(OC)c4OC)CC3	C27H30F2N2O3	468.536	Lomerizine HCl
BIOMOL	DL--372	[C@]([H])([C@]1(C)C(=Cc(c2C1)onc2)CC3)(CC[C@@]4(C)[C@@]5([H])CC[C@@]4(O)C#C)[C@@]35[H]	C22H27NO2	337.455	Danazol
BIOMOL	DL-439	c(cc(C(=O)c1ccccc1)cc2)(nc(NC(=O)OC)[nH]3)c23	C16H13N3O3	295.293	Mebendazol
BIOMOL	DL-514	[C@@]([H])([C@H](N(C)C)C(O)=C(C(=O)N)C1=O)(C[C@@]([H])(C2=C3O)[C@@](O)(C)c(c4C2=O)cccc4O)[C@@]13O	C22H24N2O8	444.435	Tetracycline
BIOMOL	DL-440	c1(C(=O)N)cnccc1	C6H6N2O	122.125	Medroxyprogesterone 17-acetate
BIOMOL	DL-169	c(nc(OC)cc1)(nc(S(=O)Cc2ncc(C)c(OC)c2C)[nH]3)c13	C16H18N4O3S	346.404	Tenatoprazole
BIOMOL	DL-441	c1(Nc2cccc(C)c2C)ccccc1C(=O)O	C15H15NO2	241.285	Mefenamic acid
BIOMOL	DL-190	c(c(C(=O)N)nc1)(N=NN(C)C2=O)n12	C6H6N6O2	194.151	Temozolomide
BIOMOL	DL-442	c1(ccc(C[C@H](N)C(=O)O)cc1)N(CCCl)CCCl	C13H18Cl2N2O2	305.2	Melphalan
BIOMOL	DL-206	[C@]1([H])([C@H](C)CC2=C3CCC(=O)C2)[C@]3([H])CC[C@@]4(C)[C@@]1([H])CC[C@@]4(O)C#C	C21H28O2	312.446	Tibolone
BIOMOL	DL-443	[C@@](N)(C)(Cc1ccc(O)c(O)c1)C(=O)O	C10H13NO4	211.215	Methyldopa
BIOMOL	DL-515	C(OCc1ccsc1Cl)(Cn2cncc2)c3ccc(Cl)cc3Cl	C16H13Cl3N2OS	387.711	Tioconazole
BIOMOL	DL-444	[C@]1([H])(C[C@H](C)C(=C2)[C@]3(C)C=CC2=O)[C@]3([H])[C@@H](O)C[C@@]4(C)[C@@]1([H])CC[C@]4(O)C(=O)CO	C22H30O5	374.471	Methylprednisolone
BIOMOL	DL-516	S(=O)(=O)(CC)CCn1c(C)ncc1[N+]([O-])=O	C8H13N3O4S	247.272	Tinidazole
BIOMOL	DL-445	c1(OCC(O)CNC(C)C)ccc(CCOC)cc1	C15H25NO3	267.364	Metoprolol tartrate
BIOMOL	DL-517	[C@H]1(O[C@@H]2[C@H](O)[C@@H](N)[C@H](O)[C@@H](CO)O2)[C@H](N)C[C@H](N)[C@@H](O[C@@H]3[C@H](N)C[C@H](O)[C@@H](CN)O3)[C@@H]1O	C18H37N5O9	467.514	Tobramycin (free base)
BIOMOL	DL-446	C1(=S)NC=CN1C	C4H6N2S	114.169	Methimazole

BIOMOL	DL-188	c1(nc(N2CCC(N)C2)c(F)c3)c3C(=O)C(C(=O)O)=CN1c4ccc(F)cc4F	C19H15F3N4O3	404.343	Tosufloxacin
BIOMOL	DL-447	n1(CCO)c(C)ncc1[N+]([O-])=O	C6H9N3O3	171.154	Metronidazole
BIOMOL	DL-518	C([C@@](O)(CC)C(=O)OC1)(C=C(N2C3=O)c4c(cc(c5n4)c(CN(C)C)c(O)cc5)C2)=C13	C23H23N3O5	421.446	Topotecan
BIOMOL	DL-448	c1(ccc(Cl)cc1Cl)C(OCc2ccc(Cl)cc2Cl)Cn3cncc3	C18H14Cl4N2O	416.129	Miconazole
BIOMOL	DL-519	C(/c1ccccc1)(\c2ccc(OCCN(C)C)cc2)=C(/CCCl)\c3ccccc3	C26H28ClNO	405.96	Toremifene
BIOMOL	DL-138	c1(c(Cl)cccc1Cl)OC(C)C2=NCCN2	C11H12Cl2N2O	259.132	Lofexidine
BIOMOL	DL-373	[C@@]([H])([C@@]1([H])[C@@](C)(C(=O)CC1)CC2)(CC=C3[C@]4(C)CC[C@H](O)C3)[C@]24[H]	C19H28O2	288.424	Dehydroepiandrosterone
BIOMOL	DL-167	N(C(C(=O)O)=C(S[C@@H]1CN[C@H](C(=O)N(C)C)C1)[C@@H]2C)(C(=O)[C@]3([H])[C@H](O)C)[C@]23[H]	C17H25N3O5S	383.463	Meropenem
BIOMOL	DL-154	C(=C(\CCNC1)/C1)(/c2c(cccn2)CC3)\c(ccc(Cl)c4)c34	C19H19ClN2	310.821	Desloratadine
BIOMOL	DL-205	C1(NCCN(CCO)CCCc2ccc([N+]([O-])=O)cc2)=CC(=O)N(C)C(=O)N1C	C19H27N5O5	405.448	Nifekalant HCl
BIOMOL	DL-374	[C@]1(CCN2C)(CCCC3)[C@@]3([H])[C@@H]2Cc4c1cc(OC)cc4	C18H25NO	271.397	Dextromethorphan HBr
BIOMOL	DL-309	c1(C[C@@H]2C(=O)N[C@@H](CCCCN)C(=O)N[C@@H]([C@H](O)C)C(=O)N[C@H](C(=O)N[C@H](CO)[C@H](O)C)CSSC[C@H](NC(=O)[C@H](N)Cc3ccccc3)C(=O)N[C@@H](Cc4ccccc4)C(=O)N2)c[nH]c5c1cccc5	C49H66N10O10S2	1019.239	Octreotide
BIOMOL	DL-375	c1(Nc2c(Cl)cccc2Cl)ccccc1CC(O)=O	C14H11Cl2NO2	296.149	Diclofenac, Na
BIOMOL	DL-194	[C@@H]1(OC(CC)CC)C=C(C(=O)OCC)C[C@H](N)[C@H]1NC(=O)C	C16H28N2O4	312.405	Oseltamivir
BIOMOL	DL-376	[C@@H]1(CC[C@@H](CO)O1)N2C=CC(N)=NC2=O	C9H13N3O3	211.218	2',3' - dideoxycytidine
BIOMOL	DL-196	C(O)(CCN)(P(=O)(O)O)P(=O)(O)O	C3H11NO7P2	235.069	Pamidronic acid
BIOMOL	DL-377	C(/CC)(\c1ccc(O)cc1)=C(\CC)/c2ccc(O)cc2	C18H20O2	268.35	Diethylstilbestrol
BIOMOL	DL-197	c(nc(N)s1)(CC[C@H](NCCC)C2)c12	C10H17N3S	211.327	Pramipexole
BIOMOL	DL-378	c1(ccc(F)cc1F)c2ccc(O)c(C(=O)O)c2	C13H8F2O3	250.198	Diflunisal
BIOMOL	DL-310	c1(cccc2)c2[nH]cc1C[C@H](C(=O)N[C@@H](CC(C)C)C(=O)N[C@@H](CCCNC(=N)N)C(=O)N3CCC[C@H]3C(=O)NCC(=O)N)NC(=O)[C@@H](NC(=O)[C@H](CO)NC(=O)[C@@H](NC(=O)[C@@H](NC(=O)[C@@H]4CCC(=O)N4)Cc5nc[nH]c5)Cc6c[nH]c7c6	C64H82N18O13	1311.449	Triptorelin
BIOMOL	DL-379	N(CC)(CC)C(=S)SSC(=S)N(CC)CC	C10H20N2S4	296.539	Disulfiram
BIOMOL	NP-526	c(c1O2)(C(=O)[C@@]2(C)O\C=C\[C@H](OC)[C@H](C)[C@H](C(=O)OC)[C@@H](C)[C@@H](O)[C@H](C)[C@H](O)[C@H](C)\C=C\C=C(\C)/C(=O)N3)c(c([O-])cc3c4O)c4c(O)c1C	C37H46NO12	696.761	Rifamycin sv
BIOMOL	DL-204	c(nc(N1CCN(C(=O)C2COc3c(cccc3)O2)CC1)nc4N)(cc(OC)c(OC)c5)c45	C23H25N5O5	451.475	Doxazosin mesylate
BIOMOL	DL-198	C(O)(P(=O)(O)O)(P(=O)(O)O)Cc1cnccc1	C7H11NO7P2	283.112	Risedronic acid
BIOMOL	DL-380	[C@H]1(O[C@H](C)[C@@H](O)[C@H]1O)N2C=C(F)C(=O)NC2=O	C9H11FN2O5	246.192	Doxifluridine
BIOMOL	DL-449	[C@@]([H])([C@H](N(C)C)C(O)=C(C(=O)N)C1=O)(C[C@@]([H])(C2=C3O)Cc(c4C2=O)c(N(C)C)ccc4O)[C@@]13O	C23H27N3O7	457.476	Minocycline HCl
BIOMOL	DL-520	c1(Nc2cccc(Cl)c2C)ccccc1C(=O)O	C14H12ClNO2	261.704	Tolfenamic acid
BIOMOL	DL-450	c(C(=O)c(c1C2=O)c(O)ccc1O)(c(NCCNCCO)ccc3NCCNCCO)c23	C22H28N4O6	444.481	Mitoxantrone 2HCl
BIOMOL	DL-521	c1(ccc(CC(O)=O)n1C)C(=O)c2ccc(C)cc2	C15H15NO3	257.285	Tolmetin Na
BIOMOL	T-104	[C@@]1([C@H](OC(=O)c2ccccc2)[C@@]3(O)C(C)(C)C(=C(C)[C@@H](OC(=O)[C@H](O)[C@@H](NC(=O)c4ccccc4)c5ccccc5)C3)[C@@H](OC(=O)C)C6=O)([C@@]6(C)[C@@H](O)C[C@@]([C@]17OC(C)=O)([H])OC	C47H51NO14	853.906	Taxol
BIOMOL	DL-522	N1(C(=O)NC(=O)N(C)C1=O)c2ccc(Oc3ccc(SC(F)(F)F)cc3)c(C)c2	C18H14F3N3O4S	425.382	Toltrazuril
BIOMOL	DL-452	C(C#N)(CCCC)(Cn1ncnc1)c2ccc(Cl)cc2	C15H17ClN4	288.775	Myclobutanil
BIOMOL	DL-523	[C@]1(O)(CCCC[C@@H]1CN(C)C)c2cccc(OC)c2	C16H25NO2	263.375	Tramadol HCl
BIOMOL	DL-453	c(N1C=C(C(=O)O)C2=O)(c2cc(F)c3N4CCC(O)CC4)c3CCC1C	C19H21FN2O4	360.379	Nadifloxacin
BIOMOL	DL-524	[C@](F)([C@]1(C)C(=CC(=O)C=C1)CC2)([C@@H](O)C[C@@]3(C)[C@@]4([H])C[C@@H](O)[C@]3(O)C(=O)CO)[C@@]24[H]	C21H27FO6	394.434	Triamcinolone
BIOMOL	DL-454	c(cc(CCC(=O)C)cc1)(ccc(OC)c2)c12	C15H16O2	228.286	Nabumetone
BIOMOL	DL-525	c1(cc(OC)c(OC)c(OC)c1)Cc2cnc(N)nc2N	C14H18N4O3	290.318	Trimethoprim
BIOMOL	DL-455	c1(cccc2)c2cccc1CC3=NCCN3	C14H14N2	210.274	Naphazoline HCl
BIOMOL	DL-158	[illegible] 3	C17H20N2O2	284.353	Tropisetron HCl
BIOMOL	DL-456	N1(CCOc2ccccc2)C(=O)N(CCCN3CCN(c4cccc(Cl)c4)CC3)N=C1CC	C25H32ClN5O2	470.007	Nefazodone
BIOMOL	DL-526	[C@@H]1(O[C@@H]2O[C@@H](C)[C@H](O)[C@@](O)(C)C2)[C@@H](C)O[C@@H](O[C@@H]3[C@H](C)[C@@H](O)CC(=O)O[C@@H](CC)[C@@H](CO[C@H]4O[C@@H](C)[C@H](O)[C@H](OC)[C@@H]4OC)\C=C(\C)/C=C/C(=O)[C@H](C)C[C@@H]3C=O)[C@	C45H75NO17	902.074	Tylosin tartrate
BIOMOL	DL-457	c1(Nc2cccc(C(F)(F)F)c2)ncccc1C(=O)O	C13H9F3N2O2	282.218	Niflumic acid
BIOMOL	DL-527	c(N=C(N)NC1=O)(n(COCCOC(=O)[C@@H](N)C(C)C)cn2)c12	C13H20N6O4	324.336	Valaciclovir

BIOMOL	DL-458	[C@@]([H])([C@@]1([H])[C@@]([H])([C@]2([H])C(=CC(=O)CC2)CC1)CC3)(CC[C@@]4(O)C#C)[C@]34C	C20H26O2	298.419	Norethindrone
BIOMOL	DL-528	[C@](C)([C@@H](OC(=O)C)[C@@H]([N+]1(C)CCCCC1)C2)(CC[C@@]3([H])[C@@]4([H])CC[C@]5([H])[C@]3(C)C[C@H](N6CCCCC6)[C@@H](OC(=O)C)C5)[C@@]24[H]	C34H57N2O4	557.827	Vecuronium Br
BIOMOL	DL-187	C1CCC[N+]1(CC=C)[C@H]2C[C@]([H])([C@@]3(C)[C@H]2OC(=O)C)[C@@]4([H])[C@@]([H])([C@]5(C)[C@]([H])(C[C@H](O)[C@@H](N6CCOCC6)C5)CC4)CC3	C32H53N2O4	529.774	Rocuronium bromide
BIOMOL	DL-181	c(n(CC1OCCO1)cn2)(C(=O)N(C)C(=O)N3C)c23	C11H14N4O4	266.253	Doxofylline
BIOMOL	DL-148	c(cc(S(=O)CCC)cc1)(nc(NC(=O)OC)[nH]2)c12	C12H15N3O3S	281.331	Ricobendazole
BIOMOL	DL-381	[C@@]([H])([C@H](N(C)C)C(O)=C(C(=O)N)C1=O)([C@@H](O)[C@@]([H])(C2=C3O)[C@@H](C)c(c4C2=O)cccc4O)[C@@]13O	C22H24N2O8	444.435	Doxycycline HCl
BIOMOL	DL-176	C1[C@]2([H])N([C@@H](C(O)=O)C(C)(C)S2(=O)=O)C1=O	C8H11NO5S	233.242	Sulbactam
BIOMOL	PI-152	N1(C(=O)[C@H](C)N[C@H](C(=O)OCC)CCc2ccccc2)CCC[C@H]1C(=O)O	C20H28N2O5	376.447	Enalapril
BIOMOL	DL-143	[illegible] O	C14H18Cl2N2O6S	413.274	Thiamphenicol glycinate
BIOMOL	DL-383	c(nc(N1CCNCC1)c(F)c2)(N(CC)C=C(C(=O)O)C3=O)c23	C15H17FN4O3	320.319	Enoxacin
BIOMOL	DL-175	c1(ccccc1Cl)C(O)CNC(C)(C)C	C12H18ClNO	227.73	Tulobuterol
BIOMOL	DL-384	c1(cc(N2CCN(CC)CC2)c(F)c3)c3C(=O)C(C(=O)O)=CN1C4CC4	C19H22FN3O3	359.395	Enrofloxacin
BIOMOL	NP-461	[C@@](CC1)([C@@H]2[C@@](O)(C(=O)OC)[C@@H]3OC(=O)C)(c4c(cc(OC)c([C@@]5(C(OC)=O)C[C@@]([H])(CN6Cc7c5[nH]c(c78)cccc8)C=C(CC)C6)c4)N2C)[C@@](N1CC=C9)([C@]39CC)[H]	C45H54N4O8	778.932	Vinorelbine
BIOMOL	DL-385	[C@]1([H])(CC[C@@]2(O)C#C)[C@]2(C)CC[C@@]3([H])[C@@]1([H])CCC(=C4)[C@]3(C)CCC4=O	C21H28O2	312.446	Ethisterone
BIOMOL	DL-220	[C@](CC1)(c2c(cc(OC)c([C@@]3(C(=O)OC)C[C@]4([H])CN(C[C@](O)(CC)C4)CCc5c3[nH]c(c56)cccc6)c2)N7C)([C@]7([H])[C@@](O)(C(=O)N)[C@@H]8O)[C@@]([H])(N1CC=C9)[C@]89CC	C43H55N5O7	753.926	Vindesine
BIOMOL	DL-386	c(cc(OC)cc1)(nc(S(=O)Cc2ncc(C)c(OC)c2C)[nH]3)c13	C17H19N3O3S	345.416	Esomeprazole potassium
BIOMOL	DL-210	c1(C(O)CNCCCCCCOCCCCc2ccccc2)ccc(O)c(CO)c1	C25H37NO4	415.566	Salmeterol
BIOMOL	BL-093	[C@]1([H])(CC[C@@H]2O)[C@]2(C)CC[C@@]3([H])[C@@]1([H])CCc4c3ccc(O)c4	C18H24O2	272.382	Estradiol
BIOMOL	T-117	c1([nH]c(c23)cccc2)c3CCN(C[C@@](CC)(O)C4)C[C@]4(C[C@]1(C(=O)OC)c5c(OC)cc6c([C@@]7([C@@]8([H])N6C=O)[C@](N9CC7)([H])[C@](C=CC9)(CC)[C@@H](OC(C)=O)[C@]8(O)C(OC)=O)c5)[H]	C46H56N4O10	824.958	Vincristine sulfate
BIOMOL	DL-388	[C@]1([H])(C[C@@H](O)[C@@H]2O)[C@]2(C)CC[C@@]3([H])[C@@]1([H])CCc4c3ccc(O)c4	C18H24O3	288.381	Estriol
BIOMOL	DL-312	c(ccc(OC)c1)(n(C(=O)c2ccc(Cl)cc2)c(C)c3CC(=O)OCC(=O)O)c13	C21H18ClNO6	415.824	Acemetacin
BIOMOL	BL-090	[C@]1([H])(CCC2=O)[C@]2(C)CC[C@@]3([H])[C@@]1([H])CCc4c3ccc(O)c4	C18H22O2	270.366	Estrone
BIOMOL	DL-459	c(cc(N1CCNCC1)c(F)c2)(N(CC)C=C(C(=O)O)C3=O)c23	C16H18FN3O3	319.331	Norfloxacin
BIOMOL	DL-529	C1(O)(CCCCC1)C(CN(C)C)c2ccc(OC)cc2	C17H27NO2	277.402	Venlafaxine HCl
BIOMOL	DL-460	[C@H]1(O[C@H](C)[C@@H](O)[C@H](N)[C@@H]1O)OC2\C=C\C=C\C=C\C=C\CC\C=C\C=C\C(C)C(O)C(C)C(C)OC(=O)CC(O)CC(O)CC(O)CCC(O)C(O)CC(=O)CC(O)C(C(=O)O)C(O)C2	C47H75NO17	926.095	Nystatin
BIOMOL	DL-530	n1([C@H]2[C@@H](O)[C@H](O)[C@@H](CO)O2)cnc3c1ncnc3N	C10H13N5O4	267.241	Vidarabine
BIOMOL	DL-461	c(N1C=C(C(=O)O)C2=O)(c2cc(F)c3N4CCN(C)CC4)c3OCC1C	C18H20FN3O4	361.368	Ofloxacin
BIOMOL	DL-531	c1(ccncc1)C(=O)NNC(C)C	C9H13N3O	179.219	Iproniazid
BIOMOL	DL-106	C(SSC1=S)(=C1C)c2nccnc2	C8H6N2S3	226.342	Oltipraz
BIOMOL	NA-103	c1(C(=O)\N=C(/N)\NCc2ccccc2)nc(Cl)c(N)nc1N	C13H14ClN7O	319.75	Benzamil
BIOMOL	DL-462	c(cc(OC)cc1)(nc(S(=O)Cc2ncc(C)c(OC)c2C)[nH]3)c13	C17H19N3O3S	345.416	Omeprazole
BIOMOL	NA-139	C1(C(=O)Nc2c(C)cccc2C)CCCCN1CCCC	C18H28N2O	288.428	Bupivacaine HCl
BIOMOL	DL-463	c1(cccc2)c2C(=O)Cc3c(cccc3)N1C(=O)N	C15H12N2O2	252.268	Oxcarbazepine
BIOMOL	NH-101	n1(Cc2ccc(F)cc2)c(NC3CCN(CCc4ccc(OC)cc4)CC3)nc5c1cccc5	C28H31FN4O	458.57	Astemizole
BIOMOL	DL-464	C(=N/OCc1ccc(Cl)cc1Cl)(/Cn2cncc2)\c3ccc(Cl)cc3Cl	C18H13Cl4N3O	429.127	Oxiconazole nitrate
BIOMOL	NH-106	C(=C(\CCN(C)C1)/C1)(/c2c(cccc2)CC3=O)\c(ccs4)c34	C19H19NOS	309.425	Ketotifen fumarate
BIOMOL	DL-465	N([C@@H](C([O-])=O)C(C)(C)S1)(C(=O)[C@H]2NC(=O)c3c(C)onc3c4ccccc4)[C@H]12	C19H18N3O5S	400.428	Oxacillin sodium monohydrate
BIOMOL	NH-107	[C@@]1(C(=O)O)(CCN([C@@H]2CC[C@@](C#N)(c3ccc(F)cc3)CC2)C[C@@H]1C)c4ccccc4	C26H29FN2O2	420.519	Levocabastine HCl
BIOMOL	DL-466	c(cc(S(=O)c1ccccc1)cc2)(nc(NC(=O)OC)[nH]3)c23	C15H13N3O3S	315.347	Oxfendazole
BIOMOL	NO-101	[C@](CCN1CC=C)([C@@H]2C(=O)CC3)([C@]3(O)[C@H]1C4)c(c4ccc5O)c5O2	C19H21NO4	327.374	Naloxone HCl
BIOMOL	DL-467	c(ccc(OCCC)c1)([nH]c(NC(=O)OC)n2)c12	C12H15N3O3	249.266	Oxibendazole

BIOMOL	NO-102	[C@](CCN1CC=C)([C@@H]2\C(=N\N=C(/CC3)\[C@H](O4)[C@@](CCN5CC=C)([C@]3(O)[C@H]5C6)c(c4c(O)cc7)c67)\CC8)([C@]8(O)[C@H]1C9)c(c9ccc%10O)c%10O2	C38H42N4O6	650.763	Naloxonazine 2HCl
BIOMOL	DL-313	c1(C(=O)O)ccccc1OC(=O)C	C9H8O4	180.157	Acetylsalicylic acid
BIOMOL	DL-390	C(O)(C)(P([O-])(=O)O)P([O-])(=O)O	C2H6O7P2	204.012	Etidronate 2Na
BIOMOL	DL-314	c1(C(=O)O)ncc(C)[n+]([O-])c1	C6H6N2O3	154.123	Acipimox
BIOMOL	DL-170	c1(\C=C\C(\C)=C\C=C\C(\C)=C\C(=O)OCC)c(C)cc(OC)c(C)c1C	C23H30O3	354.482	Etretinate
BIOMOL	DL-315	c1(Nc2c(Cl)cccc2Cl)ccccc1CC(=O)OCC(=O)O	C16H13Cl2NO4	354.185	Aceclofenac
BIOMOL	DL-563	c(cc(S(=O)(=O)N)c(Cl)c1)(S(=O)(=O)NC(C(Cl)Cl)N2)c12	C8H8Cl3N3O4S2	380.656	Trichloromethiazide
BIOMOL	DL-316	c(N=C(N)NC1=O)(n(COCCO)cn2)c12	C8H11N5O3	225.205	Acycloguanosine
BIOMOL	DL-392	c1(nc(N)nc2)c2ncn1CCC(COC(=O)C)COC(=O)C	C14H19N5O4	321.332	Famciclovir
BIOMOL	DL-317	[C@H]1(O[C@H](CO)[C@@H](N=[N+]=[N-])C1)N2C=C(C)C(=O)NC2=O	C10H13N5O4	267.241	3'-azido-3'-deoxythymidine
BIOMOL	DL-393	c(cc(Sc1ccccc1)cc2)(nc(NC(=O)OC)[nH]3)c23	C15H13N3O2S	299.348	Fenbendazole
BIOMOL	DL-318	n1c(O)c2c(N=NC2)nc1	C5H4N4O	136.111	Allopurinol
BIOMOL	DL-394	c1(ccc(C(=O)CCC(=O)O)cc1)c2ccccc2	C16H14O3	254.281	Fenbufen
BIOMOL	PR-123	C(O)(CCCN)(P([O-])(=O)O)P(=O)(O)O	C4H12NO7P2	248.088	Alendronate
BIOMOL	DL-395	c12c(C(c3ccc(O)cc3)CNCC1)cc(O)c(O)c2Cl	C16H16ClNO3	305.756	Fenoldopam mesylate
BIOMOL	DL-320	c1(N(C)C)nc(N(C)C)nc(N(C)C)n1	C9H18N6	210.279	Altretamine
BIOMOL	DL-396	c1(Oc2ccccc2)cccc(C(C)C(=O)O)c1	C15H14O3	242.27	Fenoprofen
BIOMOL	DL321	c(ccc(SCCC)c1)([nH]c(NC(=O)OC)n2)c12	C12H15N3O2S	265.331	Albendazole
BIOMOL	DL-397	c1(ccc(OC(C)(C)C(=O)OC(C)C)cc1)C(=O)c2ccc(Cl)cc2	C20H21ClO4	360.831	Fenofibrate
BIOMOL	DL-565	C(C(=O)O)CC(=O)O.c(cc(CS(=O)(=O)NC)cc1)(c(CCN(C)C)c[nH]2)c12	C18H27N3O6S	413.488	Sumatriptan Succinate
BIOMOL	DL-398	[C@]1([H])(CC[C@@H]2C(=O)NC(C)(C)C)[C@]2(C)CC[C@@]3([H])[C@@]1([H])CC[C@]4([H])[C@]3(C)C=CC(=O)N4	C23H36N2O2	372.544	Finasteride
BIOMOL	DL-468	c(ccc(OC(F)F)c1)([nH]c(S(=O)Cc2nccc(OC)c2OC)n3)c13	C16H15F2N3O4S	383.37	Pantoprazole
BIOMOL	AC-1053	c1(ccnc2C)c2[nH]c3c1ccc(OC)c3	C13H12N2O	212.247	Harmine
BIOMOL	NS-710	[C@@H]1(c2ccc(F)cc2)CCNC[C@H]1COc3ccc4c(OCO4)c3	C19H20FNO3	329.365	Paroxetine HCl
BIOMOL	NS-102	c1(SCCCN(C)C)ccccc1NC(=O)\C=C\c2ccccc2	C20H24N2OS	340.482	Cinanserin
BIOMOL	DL-470	c(N1C=C(C(=O)O)C2=O)(c2cc(F)c3C4(N)CC4)c3OC[C@@H]1C	C16H15FN2O4	318.3	Pazufloxacin
BIOMOL	NS-107	c(cccc1)(N(CCN(C)C)C(=O)c(c2N3C)cccc2)c13	C18H21N3O	295.379	Dibenzepine HCl
BIOMOL	DL-471	C(N(CC)c1c(cc(F)c(N2CCN(C)CC2)c1)C3=O)=C3C(=O)O	C17H20FN3O3	333.357	Pefloxacine mesylate
BIOMOL	NS-108	[C@](O)(O[C@@](C(C)C)(NC(=O)[C@H]1CN(C)[C@@]([H])([C@]2([H])C1)Cc3c(c4[nH]c3)c2ccc4)C5=O)([C@@]6([H])N(CCC6)C(=O)[C@@H]7Cc8ccccc8)N57	C35H41N5O5	611.731	Dihydroergocristine mesylate
BIOMOL	DL-472	c(N=C(N)NC1=O)(n(CCC(CO)CO)cn2)c12	C10H15N5O3	253.258	Penciclovir
BIOMOL	NS-109	C1(N2CCN(C)CC2)=Nc3c(ccc(F)c3)Cc4c1cccc4	C19H20FN3	309.381	Fluperlapine
BIOMOL	DL-473	c(n(C)cn1)(C(=O)N(CCCCC(=O)C)C(=O)N2C)c12	C13H18N4O3	278.307	Pentoxifylline
BIOMOL	NS-140	C(CCNC)(Oc1ccc(C(F)(F)F)cc1)c2ccccc2	C17H18F3NO	309.326	Fluoxetine HCl
BIOMOL	DL-474	N([C@@H](C([O-])=O)C(C)(C)S1)(C(=O)[C@H]2NC(=O)COc3ccccc3)[C@H]12	C16H17N2O5S	349.382	Pencillin v potassium
BIOMOL	NS-145	c1(C(=O)C(Cn2ccnc2C)CC3)c3n(C)c4c1cccc4	C18H19N3O	293.363	Ondansetron
BIOMOL	DL-475	N1(c2ccccc2)C(=O)C(CCCC)C(=O)N1c3ccccc3	C19H20N2O2	308.374	Phenylbutazone
BIOMOL	NS-515	c(cccc1)(NC(=O)N(CCN2CCC(C(=O)c3ccc(F)cc3)CC2)C4=O)c14	C22H22FN3O3	395.427	Ketanserin tartrate
BIOMOL	DL-476	[C@H]([C@H](NC(=O)[C@H](NC(=O)N1CCN(CC)C(=O)C1=O)c2ccccc2)C3=O)(SC(C)(C)[C@@H]4C(O)=O)N34	C23H27N5O7S	517.555	Piperacillin
BIOMOL	DL-562	C(O)(C(=O)O[C@@H]1C[C@@]([H])([N+](C)(C)[C@@]2([H])C1)[C@H]([C@H]23)O3)(c4sccc4)c5sccc5.[Br-]	C19H22BrNO4S2	472.416	Tiotropium Br
BIOMOL	DL-477	[C@]12([H])C(C=C[C@H](C)[C@@H]1CC[C@@H]3C[C@@H](O)CC(=O)O3)=C[C@@H](O)C[C@@H]2OC([C@@H](C)CC)=O	C23H34O6	406.512	Pravastatin lactone
BIOMOL	NS-531	c1(cc(S(=O)C)cc2)c2Sc3c(cccc3)N1CCC4CCCCN4C	C21H26N2OS2	386.574	Mesoridazine besylate
BIOMOL	GR-341	S(P(=O)(O)O)CCNCCCN	C5H15N2O3PS	214.223	Amifostine
BIOMOL	DL-399	C1(=O)NC(=O)NC=C1F	C4H3FN2O2	130.077	5-fluorouracil
BIOMOL	DL-324	C1(CC)(CCC(=O)NC1=O)c2ccc(N)cc2	C13H16N2O2	232.278	Dl-aminoglutethimide
BIOMOL	DL-400	c1(ccc(C(C)C(=O)O)cc1F)c2ccccc2	C15H13FO2	244.261	Flurbiprofen
BIOMOL	DL-325	c1(C(=O)O)ccc(N)cc1O	C7H7NO3	153.135	4-aminosalicylic acid
BIOMOL	DL-401	c(c(F)c(N1CCN(C)CC1)c(F)c2)(N(CCF)C=C(C(=O)O)C3=O)c23	C17H18F3N3O3	369.338	Fleroxacin
BIOMOL	DL-326	c1(C(=O)O)cc(N)ccc1O	C7H7NO3	153.135	5-aminosalicylic acid
BIOMOL	DL-402	c1(ccc([C@@H](O)[C@@H](CF)NC(=O)C(Cl)Cl)cc1)S(C)(=O)=O	C12H14Cl2FNO4S	358.213	Florfenicol

BIOMOL	DL-327	N([C@@H](C(=O)O)C(C)(C)S1)(C(=O)[C@H]2NC(=O)[C@H](N)c3ccccc3)[C@H]12	C16H19N3O4S	349.405	Ampicillin trihydrate
BIOMOL	DL-403	[C@@H]1(C[C@H](O)[C@@H](CO)O1)N2C=C(F)C(=O)NC2=O	C9H11FN2O5	246.192	Floxuridine
BIOMOL	DL-328	C1(OC(C)OC(=O)OCC)=C(C(=O)Nc2ncccc2)N(C)S(=O)(=O)c3c1cccc3	C20H21N3O7S	447.462	Ampiroxicam
BIOMOL	DL-404	[C@]1(C(=O)CO)(OC(C)(C)O2)[C@H]2C[C@]3([H])[C@]1(C)C[C@H](O)[C@@]4(F)[C@@]3([H])C[C@H](F)C(=C5)[C@]4(C)C=CC5=O	C24H30F2O6	452.488	Fluocinolone acetonide
BIOMOL	DL-329	[C@@]([H])(C[C@@H](N)[C@@H](O[C@@H]1[C@@H](N)C[C@@H](N)[C@H](O)[C@H]1O)O2)(O[C@H](O[C@@H]3[C@H](O)[C@@H](O)[C@H](N)[C@@H](CO)O3)[C@@H](NC)[C@H]4O)[C@]24[H]	C21H41N5O11	539.577	Apramycin
BIOMOL	DL-405	c(cc(C(=O)c1ccc(F)cc1)cc2)(nc(NC(=O)OC)[nH]3)c23	C16H12FN3O3	313.283	Flubendazole
BIOMOL	NP-016	[C@@]([C@@]1([H])CC[C@H]2C)([C@@]2([H])CC3)(OO[C@@]3(C)O4)[C@]4([H])OC(=O)[C@@H]1C	C15H22O5	282.332	Artemisinin
BIOMOL	DL-406	c1(C(F)(F)F)cc(NC(=O)C(C)C)ccc1[N+]([O-])=O	C11H11F3N2O3	276.212	Flutamide
BIOMOL	AC-166	c1(OCC(O)CNC(C)C)ccc(CC(=O)N)cc1	C14H22N2O3	266.336	Atenolol
BIOMOL	DL-407	c1(ccc(F)cc1F)C(O)(Cn2ncnc2)Cn3ncnc3	C13H12F2N6O	306.271	Fluconazole
BIOMOL	DL-332	c1(cc(OC)c(OC)c2)c2CC[N+](C)(CCC(=O)OCCCCCOC(=O)CC[N+]3(C)CCc4c(cc(OC)c(OC)c4)C3Cc5ccc(OC)c(OC)c5)C1Cc6ccc(OC)c(OC)c6	C53H72N2O12	929.145	Atracurium besylate
BIOMOL	DL-408	[C@]([H])([C@]1(C)C(=C(O)C(=O)CC1)CC2)(CC[C@@]3(C)[C@@]4([H])CCC3=O)[C@@]24[H]	C19H26O3	302.408	Formestane
BIOMOL	DL-478	c(cc(C(C)C(=O)O)cc1)(Cc(c2O3)cccn2)c13	C15H13NO3	255.269	Pranoprofen
BIOMOL	NS-835	N1(CCC2CCCCN2C)c3c(cccc3)Sc4c1cc(SC)cc4	C21H26N2S2	370.575	Thioridazine HCl
BIOMOL	DL-479	[C@@]1([C@]2(C)C[C@H](O)[C@@]3([H])[C@]1(CCC(=C4)[C@]3(C)C=CC4=O)[H])(CC[C@@]2(C(CO)=O)O)[H]	C21H28O5	360.444	Prednisolone
BIOMOL	PD-125	c(C=CC(=O)N1)(cc(OCCCC(=O)N(C)C2CCCCC2)cc3)c13	C20H26N2O3	342.432	Cilostamide
BIOMOL	DL-480	[C@]([H])([C@]1(C)C(=CC(=O)CC1)CC2)(CC[C@@]3(C)[C@@]4([H])CC[C@@H]3C(=O)C)[C@@]24[H]	C21H30O2	314.462	Progesterone
BIOMOL	PD-130	c1(cnn2CC)c2ncc(C(=O)OCC)c1N\N=C(/C)\C	C14H19N5O2	289.333	Etazolate
BIOMOL	DL-481	c1(ccc(CNNC)cc1)C(=O)NC(C)C	C12H19N3O	221.299	Procarbazine HCl
BIOMOL	PD-140	c1(nc[nH]2)c2C(=O)N(C)C(=O)N1CC(C)C	C10H14N4O2	222.244	Isobutylmethylxanthine
BIOMOL	DL-482	c1(CCC)nccc(C(=S)N)c1	C9H12N2S	180.27	Prothionamide
BIOMOL	PD-141	c1(nc(N)nc(N)n1)c2cc(Cl)ccc2Cl	C9H7Cl2N5	256.091	Irsogladine maleate
BIOMOL	DL-483	[C@@]1([C@]2(C)CC(=O)[C@@]3([H])[C@]1(CCC(=C4)[C@]3(C)C=CC4=O)[H])(CC[C@@]2(C(=O)CO)O)[H]	C21H26O5	358.428	Prednisone
BIOMOL	PD-152	C1(=C(C)NC(=O)C(C#N)=C1)c2ccncc2	C12H9N3O	211.219	Milrinone
BIOMOL	DL-484	[C@]([H])([C@]1(C)C(C[C@@H](O)CC1)=CC2)(CC[C@@]3(C)[C@@]4([H])CC[C@@H]3C(C)=O)[C@@]24[H]	C21H32O2	316.478	Pregnenolone
BIOMOL	PD-175	c1(OC2CCCC2)cc(C3CC(=O)NC3)ccc1OC	C16H21NO3	275.343	Rolipram
BIOMOL	DL-485	c1(NC(C)CCCN)cc(OC)cc2c1nccc2	C15H21N3O	259.347	Primaquine phosphate
BIOMOL	PD-179	c1(ccc(\N=C(/NC)\NC#N)cc1)C2=NNC(=O)CC2C	C14H16N6O	284.316	Siguazodan
BIOMOL	DL-486	C1(CN(C(=O)C2CCCCC2)CC3=O)N3CCc4c1cccc4	C19H24N2O2	312.406	Praziquantel
BIOMOL	PD180	C1(=C\C(=N/c2c(C)cc(C)cc2C)\N(C)C3=O)N3CCc4c1cc(OC)c(OC)c4	C24H27N3O3	405.489	Trequinsin
BIOMOL	DL-487	c1(c(O)c(C(=O)O)cc(cc2)c1cc2)Cc3c(O)c(C(=O)O)cc4c3cccc4.C5(=NCCCN5C)\C=C\c6sccc6	C34H30N2O6S	594.677	Pyrantel pamoate
BIOMOL	PD-185	[C@@]([H])([C@@]1(CC)CCC2)(N2CC3)c(c3c(c45)cccc4)n5C(C(=O)OCC)=C1	C22H26N2O2	350.454	Vinpocetine
BIOMOL	DL-333	N1(CCN(CCCC(=O)c2ccc(F)cc2)CC1)c3ncccc3	C19H22FN3O	327.396	Azaperone
BIOMOL	DL-409	N1(C=C(F)C(=O)NC1=O)C2OCCC2	C8H9FN2O3	200.167	Ftorafur
BIOMOL	T-116	C1[C@@]([H])(C[C@](CC)(O)C2)CN2CCc(c(c3[nH]4)cccc3)c4[C@@]1(C(OC)=O)c5cc6c(N(C)[C@@]([C@@]67[C@](N8CC7)([H])[C@@]9(CC)C=CC8)([H])[C@@](O)(C(OC)=O)[C@@H]9OC(C)=O)cc5OC	C46H58N4O9	810.974	Vinblastine sulfate
BIOMOL	DL-410	c1(C(=O)O)cc(S(=O)(=O)N)c(Cl)cc1NCc2occc2	C12H11ClN2O5S	330.744	Furosemide
BIOMOL	DL-335	[C@H]1(O[C@@H]2O[C@H](C)C[C@H](N(C)C)[C@H]2O)[C@@](O)(C)C[C@@H](C)CN(C)[C@H](C)[C@@H](O)[C@@](O)(C)[C@@H](CC)OC(=O)[C@H](C)[C@@H](O[C@@H]3O[C@@H](C)[C@H](O)[C@](C)(OC)C3)[C@@H]1C	C38H72N2O12	748.984	Azithromycin
BIOMOL	DL-411	c(N=C(N)NC1=O)(n(COC(CO)CO)cn2)c12	C9H13N5O4	255.231	Ganciclovir
BIOMOL	DL-336	[C@@H]1(NC(=O)\C(=N/OC(C)(C)C(=O)O)\c2nc(N)sc2)C(=O)N(S(=O)(=O)O)[C@H]1C	C13H17N5O8S2	435.433	Aztreonam
BIOMOL	DL-412	c1(c(OC)c(N2CCNC(C)C2)c(F)c3)c3C(=O)C(C(=O)O)=CN1C4CC4	C19H22FN3O4	375.394	Gatifloxacin
BIOMOL	DL-337	[C@@]1(OC(=O)CC)(C(=O)COC(=O)CC)[C@@H](C)C[C@]2([H])[C@]1(C)C[C@H](O)C3(F)[C@@]2([H])CCC(=C4)[C@]3(C)C=CC4=O	C28H37FO7	504.588	Betamethasone

BIOMOL	DL-413	[C@H]1(O[C@H]2OC[C@@](O)(C)[C@H](NC)[C@H]2O)[C@H](N)C[C@H](N)[C@@H](O[C@@H]3[C@H](N)CC[C@@H]([C@@H](C)NC)O3)[C@@H]1O	C21H43N5O7	477.595	Gentamycin sulfate
BIOMOL	DL-338	C(c1ccccc1)(n2cncc2)c3ccc(c4ccccc4)cc3	C22H18N2	310.392	Bifonazole
BIOMOL	DL-414	c1(OCCCC(C)(C)C(=O)O)cc(C)ccc1C	C15H22O3	250.333	Gemfibrozil
BIOMOL	DL-339	C(c1ncccc1)(c2ccc(OC(=O)C)cc2)c3ccc(OC(=O)C)cc3	C22H19NO4	361.391	Bisacodyl
BIOMOL	DL-415	C(CCC1)(CN(NC(=O)NS(=O)(=O)c2ccc(C)cc2)C3)C13	C15H21N3O3S	323.411	Gliclazide
BIOMOL	DL-340	c1(CN(C)C2CCCCC2)cc(Br)cc(Br)c1N	C14H20Br2N2	376.13	Bromhexine HCl
BIOMOL	DL-416	N1(C(=O)NCCc2ccc(S(=O)(=O)NC(=O)N[C@@H]3CC[C@@H](C)CC3)cc2)CC(C)=C(CC)C1=O	C24H34N4O5S	490.616	Glimepiride
BIOMOL	DL-341	C12(CCCC1)CC(=O)N(CCCCN3CCN(c4ncccn4)CC3)C(=O)C2	C21H31N5O2	385.503	Buspirone HCl
BIOMOL	DL-417	c1(OCC(O)CO)ccccc1OC	C10H14O4	198.216	Guaifenesin
BIOMOL	DL-342	[C@@]12(CCC(=O)O1)CC[C@]3([H])[C@]2(C)CC[C@@]4([H])[C@@]3([H])C=CC(=C5)[C@]4(C)CCC5=O	C22H28O3	340.456	Canrenone
BIOMOL	DL-418	[C@@H](CC)(c1ccc(O)cc1)[C@@H](CC)c2ccc(O)cc2	C18H22O2	270.366	Hexestrol
BIOMOL	DL-488	c1(NC(C)CCCN(CC)CC)c2c(cc(Cl)cc2)nc3c1cc(OC)cc3	C23H30ClN3O	399.957	Quinacrine 2HCl dihydrate
BIOMOL	PD-190	c1(ccccc1OCCC)C2=NC(=O)c3c(nn[nH]3)N2	C13H13N5O2	271.275	Zaprinast
BIOMOL	DL-489	N1(C(=O)[C@H](C)N[C@H](C(=O)OCC)CCc2ccccc2)Cc(c3C[C@H]1C(=O)O)cccc3	C25H30N2O5	438.516	Quinapril HCl
BIOMOL	PD-195	c1(ccc(OC(F)F)c(OC)c1)C2=NNC(=O)C=C2	C12H10F2N2O3	268.216	Zardaverine
BIOMOL	DL-159	C(CSC(=O)C)(C(=O)NCC(=O)OCc1ccccc1)Cc2ccccc2	C21H23NO4S	385.477	Racecadotril
BIOMOL	PG-006 PGE1	[C@@]1([H])(CCCCCCC(=O)O)C(=O)C[C@@H](O)[C@]1([H])\C=C\[C@@H](O)CCCCC	C20H34O5	354.481	Alprostadil
BIOMOL	DL-490	c1(OCC(O)CN2CCN(CC(=O)Nc3c(C)cccc3C)CC2)ccccc1OC	C24H33N3O4	427.537	Ranolazine 2HCl
BIOMOL	PG-051	[C@H]1(\C=C\CC(O)(C)CCCC)[C@H](O)CC(=O)[C@@H]1CCCCCCC(=O)OC	C22H38O5	382.534	Misoprostol
BIOMOL	DL-491	N1(C(=O)[C@H](C)N[C@H](C(=O)OCC)CCc2ccccc2)[C@H](C(=O)O)C[C@@]3([H])[C@]1([H])CCC3	C23H32N2O5	416.511	Ramipril
BIOMOL	PI-146	c1(NCC(C)C2)c2cccc1S(=O)(=O)N[C@@H](CCCNC(=N)N)C(=O)N3CC[C@@H](C)C[C@@H]3C(=O)O	C23H36N6O5S	508.634	Argatroban
BIOMOL	DL-147	c1(cccc2)c2NC(=O)C=C1CC(C(=O)O)NC(=O)c3ccc(Cl)cc3	C19H15ClN2O4	370.786	Rebamipide
BIOMOL	PI-153	[C@@H]1(C(=O)N\C(\C(=O)O)=C/CCCCSC[C@@H](N)C(=O)O)CC1(C)C	C16H26N2O5S	358.453	Cilastatin
BIOMOL	DL-492	[C@@H]1([C@H](O)[C@H](O)[C@@H](CO)O1)n2cnc(C(=O)N)n2	C8H12N4O5	244.205	Ribavirin
BIOMOL	PR-117	[C@@]([C@]1([H])[C@@]([H])(C)[C@@]([H])(OC(=O)CCC(O)=O)O2)([C@]3([H])[C@]([H])(C)CC1)(OO4)[C@]2(O[C@@]4(C)CC3)[H]	C19H28O8	384.421	Artesunate
BIOMOL	DL-566	[C@@H](N(C[C@@H]1[C@H]2CCCC1)C[C@@H](O)[C@H](CSc3ccccc3)NC(=O)c4cccc(O)c4C)(C(=O)NC(C)(C)C)C2.S(=O)(=O)(O)C	C33H49N3O7S2	663.888	Nelfinavir Mesylate
BIOMOL	RA-104	c1(C(=O)NCc2cnccc2)cc(C(=O)NCc3cnccc3)ccc1OC	C21H20N4O3	376.409	Picotamide
BIOMOL	DL-494	[C@@]1(C[C@@]([H])(C[C@@]2([H])C1)C[C@@]3([H])C2)(C3)C(N)C	C12H21N	179.302	Rimantadine HCl
BIOMOL	S104	[C@@H](O1)([C@H](O)[C@@H](O)[C@H](O)[C@H]2O)[C@@H]12	C6H10O5	162.141	Conduritol b epoxide
BIOMOL	ST-405	c1(cccc2)c2cccc1OCC(O)CNC(C)C	C16H21NO2	259.343	Propranolol
BIOMOL	S-510	C(=C1C(=CC(=O)CC1)CC2)([C@@H](c3ccc(N(C)C)cc3)C[C@@]4(C)[C@@]5([H])CC[C@@]4(O)C#CC)[C@@]25[H]	C29H35NO2	429.594	Mifepristone
BIOMOL	DL-343	c(cccc1)([n+]([O-])c(\C=N\NC(=O)OC)c[n+]2[O-])c12	C11H10N4O4	262.222	Carbadox
BIOMOL	DL-419	[C@@]1([C@]2(C)C[C@H](O)[C@]([C@]13[H])([H])[C@]4(C)C(=CC(=O)CC4)CC3)(CC[C@@]2(C(CO)=O)O)[H]	C21H30O5	362.46	Hydrocortisone
BIOMOL	DL-344	C12(CCC1)C(=O)O[Pt]OC2=O	C6H6O4Pt	337.187	Carboplatin
BIOMOL	DL-420	[C@](C)([C@](C(=O)COC(=O)C)(O)CC1)(C[C@H](O)[C@@]2([H])[C@]3(CCC(=C4)[C@]2(C)CCC4=O)[H])[C@@]13[H]	C23H32O6	404.497	Hydrocortisone 21-acetate
BIOMOL	DL-345	N1(C(=O)N)c2c(cccc2)C=Cc3c1cccc3	C15H12N2O	236.269	Carbamazepine
BIOMOL	DL-421	[C@]1([H])(CCC(=C2)[C@]3(C)CCC2=O)[C@]3([H])CC[C@@]4(C)[C@@]1([H])CC[C@]4(O)C(=O)C	C21H30O3	330.461	17- hydroxyprogesterone
BIOMOL	DL-346	C1([C@@H](NC([C@H](NC(=O)N2CCN(CC)C(=O)C2=O)c3ccc(O)cc3)=O)[C@]4([H])N1C(C(O)=O)=C(CSc5nnnn5C)CS4)=O	C25H27N9O8S2	645.667	Cefoperazone acid
BIOMOL	DL-422	[C@@H]1(C[C@H](O)[C@@H](CO)O1)N2C=C(I)C(=O)NC2=O	C9H11IN2O5	354.099	Idoxuridine
BIOMOL	DL-347	N1([C@]2([H])SCC(COC(=O)C)=C1C(=O)O)C(=O)[C@H]2NC(=O)\C(=N/OC)\c3csc(N)n3	C16H17N5O7S2	455.465	Cefotaxime acid
BIOMOL	DL-423	N1(CCCl)CCCOP1(=O)NCCCl	C7H15Cl2N2O2P	261.086	Ifosfamide
BIOMOL	DL-348	N(C(C([O-])=O)=C(C[n+]1ccccc1)CS2)(C(=O)[C@H]3NC(=O)\C(=N/OC(C)(C)C(=O)O)\c4nc(N)sc4)[C@H]23	C22H22N6O7S2	546.576	Ceftazidime
BIOMOL	DL-424	c1(n(CC(C)C)cn2)c2c(N)nc3c1cccc3	C14H16N4	240.304	Imiquimod

BIOMOL	DL-349	c1(ccc([N+]([O-])=O)cc1)[C@@H](O)[C@@H](CO)NC(=O)C(Cl)Cl	C11H12Cl2N2O5	323.129	Chloramphenicol
BIOMOL	DL-425	c1(cccc2)c2CC(C)N1NC(=O)c3ccc(Cl)c(S(=O)(=O)N)c3	C16H16ClN3O3S	365.835	Indapamide
BIOMOL	DL-350	[C@]([C@@]([C@]12[H])(C)CC[C@@]3([H])[C@@]1([H])C=C(Cl)C(=C4)[C@]3(C)CCC4OC(=O)C)(C(=O)C)(OC(=O)C)CC2	C25H33ClO5	448.98	Chlormadinone acetate
BIOMOL	DL-426	c1(C(=O)NCc2ccc(OCCN(C)C)cc2)ccc(OC)c(OC)c1	C20H26N2O4	358.431	Itopride HCl
BIOMOL	DL-351	c1(ccc(CCCC(=O)O)cc1)N(CCCl)CCCl	C14H19Cl2NO2	304.212	Chlorambucil
BIOMOL	DL-427	C1(Cn2ncnc2)(OCC(COc3ccc(N4CCN(c5ccc(N6C=NN(C(C)CC)C6=O)cc5)CC4)cc3)O1)c7ccc(Cl)cc7Cl	C35H38Cl2N8O4	705.633	Itraconazole
BIOMOL	DL-352	C(CCN(C)C)(c1ncccc1)c2ccc(Cl)cc2	C16H19ClN2	274.788	Chlorpheniramine maleate
BIOMOL	EI-288	c1(C(=O)c2ccccc2)cccc([C@H](C)C(=O)O)c1	C16H14O3	254.281	Ketoprofen (s)
BIOMOL	DL-496	c1(CN2CCCCC2)cccc(OCCCNC(=O)COC(=O)C)c1	C19H28N2O4	348.437	Roxatidine acetate HCl
BIOMOL	S-515	[C@]1(C(=O)C)(OC(=O)C)CC[C@]2([H])[C@]1(C)CC[C@@]3([H])[C@@]2([H])C=C(C)C(=C4)[C@]3(C)CCC4=O	C24H32O4	384.508	Megestrol acetate
BIOMOL	DL-497	[C@@H]1(O[C@@H]2O[C@@H](C)[C@H](O)[C@](C)(OC)C2)[C@@H](C)C(=O)O[C@H](CC)[C@](O)(C)[C@H](O)[C@@H](C)\C(=N/OCOCCOC)\[C@H](C)C[C@](O)(C)[C@H](O[C@@H]3O[C@H](C)C[C@H](N(C)C)[C@H]3O)[C@H]1C	C41H76N2O15	837.047	Roxithromycin
BIOMOL	S-520	[C@]1(C(=O)C)(OC(=O)C)C(=C)C[C@]2([H])[C@]1(C)CC[C@@]3([H])[C@@]2([H])C=C(C)C(=C4)[C@]3(C)CCC4=O	C25H32O4	396.519	Melengestrol acetate
BIOMOL	DL-498	c(N1C=C(C(=O)O)C2=O)(c2cc(F)c3N4CCN(C)CC4)c3SCC1	C17H18FN3O3S	363.407	Rufloxacin
BIOMOL	S-650	C(/c1ccccc1)(\c2ccc(OCCN(C)C)cc2)=C(/CC)\c3ccccc3	C26H29NO	371.515	Tamoxifen citrate
BIOMOL	DL-499	c1(cc(N2CCNCC2)c(F)c3)c3C(=O)C(C(=O)O)=CN1c4ccc(F)cc4	C20H17F2N3O3	385.364	Sarafloxacin HCl
BIOMOL	DL-532	N(CC(C)C)(C[C@@H](O)[C@@H](NC(=O)O[C@@H]1COCC1)Cc2ccccc2)S(=O)(=O)c3ccc(N)cc3	C25H35N3O6S	505.627	Amprenavir
BIOMOL	DL-500	[C@@]([H])(O1)([C@]([H])([N+]([C@@]23[H])(C)CCCC)C[C@@H](OC(=O)[C@H](CO)c4ccccc4)C2)[C@@]13[H]	C21H30NO4	360.467	Scopolamine n-butylbromide
BIOMOL	DL-533	C1(CN2CCO[C@H](O[C@H](C)c3cc(C(F)(F)F)cc(C(F)(F)F)c3)[C@@H]2c4ccc(F)cc4)=NC(=O)NN1	C23H21F7N4O3	534.427	Aprepitant
BIOMOL	DL-501	[C@@]([H])(O1)([C@]([H])(N([C@@]23[H])C)C[C@@H](OC(=O)[C@H](CO)c4ccccc4)C2)[C@@]13[H]	C17H21NO4	303.353	Scopolamine HBr
BIOMOL	DL-534	c1(Oc2ccccc2OC)c(OCCO)nc(c3ncccn3)nc1NS(=O)(=O)c4ccc(C(C)(C)C)cc4	C27H29N5O6S	551.614	Bosentan
BIOMOL	DL-502	n1(CC(O)C)c(C)ncc1[N+]([O-])=O	C7H11N3O3	185.181	Secnidazole
BIOMOL	DL-535	[C@@]1(C#CC2CC2)(c3c(ccc(Cl)c3)NC(=O)O1)C(F)(F)F	C14H9ClF3NO2	315.675	Efavirenz
BIOMOL	DL-503	C1(CCC1)(C(N(C)C)CC(C)C)c2ccc(Cl)cc2	C17H26ClN	279.848	Sibutramine HCl
BIOMOL	DL-536	[C@]1([H])([C@H](O)C[C@@]2([H])[C@]3(C)CC[C@@H](O)C2)[C@]3([H])C[C@H](O)[C@@]4(C)[C@@]1([H])CC[C@@H]4[C@H](C)CCC(=O)NCCS(=O)(=O)O	C26H45NO7S	515.703	Taurocholic acid, sodium salt hydrate
BIOMOL	DL-149	c(c(F)c(N1C[C@@H](C)N[C@@H](C)C1)c(F)c2N)(N(C3CC3)C=C(C(=O)O)C4=O)c24	C19H22F2N4O3	392.4	Sparfloxacin
BIOMOL	SL-230	N1(CCCC)C[C@H](O)[C@@H](O)[C@H](O)[C@H]1CO	C10H21NO4	219.278	Miglustat
BIOMOL	DL-504	[C@@]12(CCC(=O)O1)CC[C@]3([H])[C@]2(C)CC[C@@]4([H])[C@@]3([H])[C@H](SC(=O)C)CC(=C5)[C@]4(C)CCC5=O	C24H32O4S	416.573	Spironolactone
BIOMOL	DL-104	[C@@]([H])([C@@]1([H])[C@@](C)([C@@H](O)CC1)CC2)([C@H](CCCCCCCCS(=O)CCCC(F)(F)C(F)(F)F)Cc3c4ccc(O)c3)[C@]24[H]	C32H47F5O3S	606.771	Fulvestrant
BIOMOL	DL-353	c(ccc(Cl)c1)(c(NC(C)CCCN(CC)CC)ccn2)c12	C18H26ClN3	319.872	Chloroquine phosphate
BIOMOL	DL-429	[C@H]1(N=C(N2C1)SCC2)c3ccccc3	C11H12N2S	204.291	Levamisole HCl
BIOMOL	T-115	N1(C(=O)c(c2C1=O)cccc2)C3CCC(=O)NC3=O	C13H10N2O4	258.229	Thalidomide
BIOMOL	DL-430	[C@](CC)([C@](O)(C#C)CC1)(CC[C@@]2([H])[C@@]3([H])CCC(=C4)[C@]2([H])CCC4=O)[C@@]13[H]	C21H28O2	312.446	Levonorgestrel
BIOMOL	DL-355	c1(cc(N2CCNCC2)c(F)c3)c3C(=O)C(C(=O)O)=CN1C4CC4	C17H18FN3O3	331.342	Ciprofloxacin
BIOMOL	DL-431	c1(O)cc(C[C@H](N)C(=O)O)ccc1O	C9H11NO4	197.188	Levodopa
BIOMOL	AR-116	C1(CCCN(C)C)(c2ccc(F)cc2)OCc3c1ccc(C#N)c3	C20H21FN2O	324.392	Citalopram
BIOMOL	DL-432	c(N1C=C(C(=O)O)C2=O)(c2cc(F)c3N4CCN(C)CC4)c3OC[C@@H]1C	C18H20FN3O4	361.368	Levofloxacin HCl
BIOMOL	DL-357	[C@@H]1(O[C@@H]2O[C@H](C)C[C@H](N(C)C)[C@H]2O)[C@@H](C)[C@H](O[C@@H]3O[C@@H](C)[C@H](O)[C@](C)(OC)C3)[C@@H](C)C(=O)O[C@H](CC)[C@](O)(C)[C@H](O)[C@@H](C)C(=O)[C@H](C)C[C@@]1(C)OC	C38H69NO13	747.953	Clarithromycin
BIOMOL	DL-433	c1(cnoc1C)C(=O)Nc2ccc(C(F)(F)F)cc2	C12H9F3N2O2	270.207	Leflunomide
BIOMOL	DL-358	C(C(=O)C(C)(C)C)(Oc1ccc(Cl)cc1)n2cncc2	C15H17ClN2O2	292.761	Climbazole
BIOMOL	DL-434	N1(C(=O)[C@H](CCCCN)N[C@H](C(=O)O)CCc2ccccc2)CCC[C@H]1C(=O)O	C21H31N3O5	405.488	Lisinopril

BIOMOL	DL-359	c1(c(Cl)c(N2CCC(N)C2)c(F)c3)c3C(=O)C(C(=O)O)=CN1C4CC4	C17H17ClFN3O3	365.787	Clinafloxacin HCl
BIOMOL	DL-435	c(c(F)c(N1CCNC(C)C1)c(F)c2)(N(CC)C=C(C(=O)O)C3=O)c23	C17H19F2N3O3	351.348	Lomefloxacin HCl
BIOMOL	DL-360	C(=C(/Cl)\c1ccccc1)(/c2ccccc2)\c3ccc(OCCN(CC)CC)cc3	C26H28ClNO	405.96	Clomiphene citrate
BIOMOL	DL-436	C(=C(\CCN(C(=O)OCC)C1)/C1)(/c2c(cccn2)CC3)\c(ccc(Cl)c4)c34	C22H23ClN2O2	382.883	Loratadine
BIOMOL	DL-361	c1(O)c(Cl)c(C)nc(C)c1Cl	C7H7Cl2NO	192.043	Clopidol
BIOMOL	DL-437	C(CCC(=O)O)(NC(=O)c1ccc(Cl)c(Cl)c1)C(=O)N(CCCCC)CCCCC	C22H32Cl2N2O4	459.406	Lorglumide
BIOMOL	AC-1290	N1(CCc2c(ccs2)C1)[C@H](C(=O)OC)c3ccccc3Cl	C16H16ClNO2S	321.822	Clopidogrel sulfate
BIOMOL	DL-438	c1(c2nnnn2[K])ccccc1c3ccc(Cn4c(CO)c(Cl)nc4CCCC)cc3	C22H22ClKN6O	461.001	Losartan potassium
BIOMOL	DL-505	[C@]([H])([C@]1(C)[C@]([H])(Cc(c2C1)[nH]nc2)CC3)(CC[C@@]4(C)[C@@]5([H])CC[C@@]4(O)C)[C@@]35[H]	C21H32N2O	328.492	Stanozolol
BIOMOL	DL-538	c(c1n(C)c2)(c2C[C@]3([H])C4=C[C@@H](C(=O)N[C@H](CO)CC)CN3C)c4ccc1	C21H27N3O2	353.458	Methysergide
BIOMOL	DL-506	[C@@H]1(O[C@@H]2O[C@@H](CO)[C@H](O)[C@@H](O)[C@@H]2NC)[C@@](O)(C=O)[C@H](C)O[C@H]1O[C@@H]3[C@@H](O)[C@H](O)[C@@H](NC(=N)N)[C@H](O)[C@H]3NC(=N)N	C21H39N7O12	581.574	Streptomycin sulfate
BIOMOL	DL-107	c1(OCC(O)CNC(C)C)ccc(CCC(=O)OC)cc1	C16H25NO4	295.374	Esmolol
BIOMOL	DL-507	c1(NS(=O)(=O)c2ccc(N)cc2)ncnc(OC)c1OC	C12H14N4O4S	310.329	Sulfadoxine
BIOMOL	DL-123	[C@H](O)(C(=O)NCCC(=O)O)C(C)(C)CO	C9H17NO5	219.235	Pantothenic acid
BIOMOL	DL-508	c1(ccc(N)cc1)S(=O)(=O)Nc2ncccn2	C10H10N4O2S	250.277	Sulfadiazine
BIOMOL	DL-146	c1(C(=O)CCc2ccc(O)cc2)c(O)cc(O)cc1O[C@H]3[C@H](O)[C@@H](O)[C@H](O)[C@@H](CO)O3	C21H24O10	436.409	Phloridzin
BIOMOL	DL-509	c1(nc(OC)nc(OC)c1)NS(=O)(=O)c2ccc(N)cc2	C12H14N4O4S	310.329	Sulfadimethoxine
BIOMOL	DL-225	c1(ccccc1)[C@@H](O)[C@@H](N)C	C9H13NO	151.206	Phenylpropanolamine
BIOMOL	DL-510	c1(ccc(N=Nc2ccc(O)c(C(=O)O)c2)cc1)S(=O)(=O)Nc3ncccc3	C18H14N4O5S	398.393	Sulfasalazine
BIOMOL	DL-234	[N+](C)(C)(C)CCOC(=O)CCC(=O)OCC[N+](C)(C)C	C14H30N2O4	290.399	Succinylcholine
BIOMOL	DL-160	c1(S(=O)(=O)N)cc(C[C@@H](C)NCCOc2ccccc2OCC)ccc1OC	C20H28N2O5S	408.512	Tamsulosin HCl
BIOMOL	DL-195	c1(cccc(C(F)(F)F)c1)C2(O)CCN(CCCC(=O)c3ccc(F)cc3)CC2	C22H23F4NO2	409.417	Trifluperidol 2HCl
BIOMOL	DL-511	c1(cc(c2nc(c3n2C)cccc3)cc4C)c4nc(CCC)n1Cc5ccc(c6ccccc6C(=O)O)cc5	C33H30N4O2	514.617	Telmisartan
BIOMOL	DL-540	c1(cc(I)c(O)c(I)c1)Oc2c(I)cc(C[C@H](N)C(=O)O)cc2I	C15H11I4NO4	776.87	L-thyroxine [(3-[4-(4-hydroxy-3,5-diiodophenoxy)-3,5-diiodophenyl]-l-alanine]
BIOMOL	DL-512	c(scc1)(C(O)=C(C(=O)Nc2ncccc2)N(C)S3(=O)=O)c13	C13H11N3O4S2	337.374	Tenoxicam
BIOMOL	DL-136	C(=C(\CCN(C)C1)/C1)(/c2c(cccc2)C=C3)\c(cccc4)c34	C21H21N	287.398	Cyproheptadine
BIOMOL	DL-513	c(cc(OC)c(OC)c1)c(c(N)nc(N2CCN(C(=O)C3OCCC3)CC2)n4)c14	C19H25N5O4	387.433	Terazosin HCl
BIOMOL	DL-559	c(cccc1)(n(Cc2ccccc2)nc3OCCCN(C)C)c13	C19H23N3O	309.405	Benzydamine
Selleck Chemicals	S1011	c1(F)ccc(Nc2c3c(cc(O[C@@H]4COCC4)c(NC(=O)C=CCN(C)C)c3)ncn2)cc1Cl	C24H25ClFN5O3	485.938	Afatinib (BIBW2992)
Selleck Chemicals	S1888	C1=C[C@@]2(C)C(CC[C@]3([H])[C@@]2([H])[C@H](O)C[C@@]4(C)[C@@]3([H])C[C@]5([H])[C@]4(C(=O)COC(=O)C)N=C(C)O5)=CC1=O	C25H31NO6	441.517	Deflazacort
Selleck Chemicals	S1198	N1(C2CCN(C(Oc3ccc(c4c3)nc5c(CN(C5=C6)C(=O)C7=C6[C@@](CC)(O)C(=O)OC7)c4CC)=O)CC2)CCCCC1	C33H38N4O6	586.678	Irinotecan
Selleck Chemicals	S1941	c1cc(CC[C@@H](C(=O)OCC)N[C@@H](C)C(=O)N2CCC[C@H]2C(=O)O)ccc1.C(=O)(C=CC(O)=O)O	C24H32N2O9	492.519	Enalapril Maleate
Selleck Chemicals	S1274	N1(C(Nc2cccc(Nc3ncc(I)c(NCCCNC(=O)c4sccc4)n3)c2)=O)CCCC1	C23H26IN7O2S	591.468	BX-795
Selleck Chemicals	S2013	N1c(c2CCC1=O)ccc(Nc3ncc(C(F)(F)F)c(NCc4cccc(S(C)(=O)=O)c4)n3)c2	C22H20F3N5O3S	491.486	PF-573228
Selleck Chemicals	S1343	C1([C@@H]2C[C@H]3C(C)(C)C)(C34[C@@]([H])(OC(=O)[C@H]4O)O5)[C@@]5(C(=O)O2)[C@]6(O)[C@@]([H])(OC(=O)[C@@H]6C)[C@H]1O	C20H24O10	424.399	Ginkgolide B
Selleck Chemicals	S2054	c1(ccccc1)C(c2c(C)cccc2)OCCN(C)C.OC(=O)CC(O)(C(O)=O)CC(=O)O	C24H31NO8	461.505	Orphenadrine Citrate
Selleck Chemicals	S1389	c1cc2c(nc(S(Cc3ncc(C)c(OC)c3C)=O)[nH]2)cc1OC	C17H19N3O3S	345.416	Omeprazole
Selleck Chemicals	S2096	c1(CS(=O)(=O)N2CCCC2)cc3c([nH]cc3CCN(C)C)cc1.C(O)(=O)C(O)CC(=O)O	C21H31N3O7S	469.552	Almotriptan Malate
Selleck Chemicals	S1544	c1ccc2c(c(C(=O)c3c(I)ccc([N+]([O-])=O)c3)cn2CC4CCCCN4C)c1	C22H22IN3O3	503.333	AM1241
Selleck Chemicals	S2153	c(ncn1[C@H]2[C@@H](O)[C@H](O)[C@@H](C(=O)NCC)O2)(c(N)nc(NCCc3ccc(CCC(=O)O)cc3)n4)c14.Cl	C23H30ClN7O6	535.981	CGS 21680 HCl
Selleck Chemicals	S1638	c1cc(C(C)C(=O)O)ccc1CC(C)C	C13H18O2	206.281	Ibuprofen

Selleck Chemicals	S2217	C1CCN(C2CCN(C(=O)Oc3cc4c(nc5c(CN6C5=CC7=C(COC(=O)[C@]7(O)CC)C6=O)c4CC)cc3)CC2)CC1.Cl.O.O.O	C33H45ClN4O9	677.185	Irinotecan HCl Trihydrate
Selleck Chemicals	S1696	C1C[C@@]2(C)C(CC[C@]3([H])[C@@]2([H])[C@H](O)C[C@@]4(C)[C@@]3([H])CC[C@@]4(O)C(CO)=O)=CC1=O	C21H30O5	362.46	Hydrocortisone
Selleck Chemicals	S2293	OC(=O)CC(C[N+](C)(C)C)O.[Cl-]	C7H16ClNO3	197.66	DL-Carnitine HCl
Selleck Chemicals	S1763	OC(=O)C=CC(=O)O.c1cc2c(Sc3c(cccc3)C(N4CCN(CCOCCO)CC4)=N2)cc1.c5cc6c(Sc7c(cccc7)C(N8CCN(CCOCCO)CC8)=N6)cc5	C46H54N6O8S2	883.086	Quetiapine Fumarate
Selleck Chemicals	S2327	c1(O)cc2c(C(=O)C(O[C@H]3[C@@H](O)[C@H](O)[C@@H](O)[C@H](C)O3)=C(c4cc(O)c(O)c(O)c4)O2)c(O)c1	C21H20O12	464.376	Myricitrin
Selleck Chemicals	S1829	c(C(=O)C=C(c1[nH]nnn1)O2)(ccc(NC(=O)c3ccc(OCCCCc4ccccc4)cc3)c5)c25	C27H23N5O4	481.503	Pranlukast
Selleck Chemicals	S2363	c1(OC)c(OC)c2c(C(=O)C=C(c3ccc(OC)cc3)O2)c(OC)c1OC	C20H20O7	372.369	Tangeretin
Selleck Chemicals	S2393	OC[C@@H](O)[C@@H](O)[C@H](O)[C@@H](O)CO	C6H14O6	182.172	Sorbitol
Selleck Chemicals	S2872	c1c(I)ccc2c1C(=Cc3cc(Br)c(O)c(Br)c3)C(=O)N2	C15H8Br2INO2	520.942	GW5074
Selleck Chemicals	S2450	C1Oc2c(ccc(O)c2)C[C@H]1c3ccc(O)cc3	C15H14O3	242.27	Equol
Selleck Chemicals	S2886	c1(cc(NC(=O)CN(C)C)cc2)c2NC(=O)c3c1cccc3	C17H17N3O2	295.336	PJ34
Selleck Chemicals	S2532	O1C(=O)C(O)=C(O)[C@@]1([H])[C@@H](O)COC(=O)CCCCCCCCCCCCCCC	C22H38O7	414.533	L-Ascorbyl 6-palmitate
Selleck Chemicals	S2900	c1(nc(C(C)C)sc1)CN(C)C(=O)N[C@@H](CCN2CCOCC2)C(=O)N[C@@H](Cc3ccccc3)CC[C@H](Cc4ccccc4)NC(=O)OCc5scnc5	C40H53N7O5S2	776.023	Cobicistat (GS-9350)
Selleck Chemicals	S2585	c1(ccc(Br)cc1)C(c2ncccc2)CCN(C)C.OC(=O)C=CC(=O)O	C20H23BrN2O4	435.312	Brompheniramine hydrogen maleate
Selleck Chemicals	S2910	C1(C)(C)CC(O)CC(C)(C)N1[O]	C9H18NO2	172.245	Tempol
Selleck Chemicals	S2691	c1cc(N2CCN(CCN3C(=O)CC4(CCCC4)CC3=O)CC2)c(OC)cc1.Cl.Cl	C22H33Cl2N3O3	458.422	BMY 7378
Selleck Chemicals	S2919	C(O)(=O)CNC(C1=C(O)c(c2N(Cc3ccccc3)C1=O)cccc2)=O	C19H16N2O5	352.341	IOX2
Selleck Chemicals	S2804	C(NC(c1ccccc1)C)(=O)c2ccccc2N=Cc3c(c4ccc3O)cccc4	C26H22N2O2	394.465	Sirtinol
Selleck Chemicals	S2930	c1cc(C#CS(=O)(=O)N)ccc1	C8H7NO2S	181.212	Pifithrin-?
Selleck Chemicals	S2819	n1(c2ccc(Cl)cc2Cl)c(c3ccc(I)cc3)c(C)c(C(NN4CCCCC4)=O)n1	C22H21Cl2IN4O	555.239	AM251
Selleck Chemicals	S3038	c1([C@@H]2N(CC(NC(=O)N3P(O)(=O)O)=N3)CCO[C@@H]2O[C@H](C)c4cc(C(F)(F)F)cc(C(F)(F)F)c4)ccc(F)cc1.CNC[C@H](O)[C@@H](O)[C@H](O)[C@H](O)CO.CNC[C@H](O)[C@@H](O)[C@H](O)[C@H](O)CO	C37H56F7N6O16P	1004.834	Fosaprepitant dimeglumine salt
Selleck Chemicals	S2842	c(ccc(C#C[C@@](COC)(C)O)n1)(C(=O)C(C(=O)NC)=C(N)N2CC)c12	C18H22N4O4	358.392	SAR131675
Selleck Chemicals	S3052	c(C(=C1CCN(Cc2cc(C)cnc2)CC1)c3c(cc(Cl)cc3)CC4)(nccc5)c45.C(O)(=O)C=CC(O)=O	C30H30ClN3O4	532.03	Rupatadine Fumarate
Selleck Chemicals	S2855	C(NCCCCNC(=S)Nc1cccc(N=C=S)c1)(=S)Nc2cccc(N=C=S)c2	C20H20N6S4	472.673	MRS 2578
Selleck Chemicals	S3067	C(Nc1ccc(Cl)cc1)(=N)NC(=N)NCCCCCCNC(=N)NC(Nc2ccc(Cl)cc2)=N.Cl.Cl	C22H32Cl4N10	578.368	Chlorhexidine HCl
Selleck Chemicals	S2863	c1cc2c(nc(N)n2S(=O)(c3ccc(C)cc3)=O)cc1	C14H13N3O2S	287.337	ML130 (Nodinitib-1)
Selleck Chemicals	S3081	[C@H]1(c2ccc(N(C)C)cc2)C[C@@]3(C)[C@@]([H])(CC[C@@]3(OC(=O)C)C(C)=O)[C@@]4([H])C1=C5C(=CC(=O)CC5)CC4	C30H37NO4	475.619	Ulipristal
Selleck Chemicals	S1014	n1c(c2c(Nc3cc(OC)c(Cl)cc3Cl)c(C#N)c1)cc(OCCCN4CCN(C)CC4)c(OC)c2	C26H29Cl2N5O3	530.446	Bosutinib (SKI-606)
Selleck Chemicals	S1890	s1cc(CSCCNC(NC)=C[N+]([O-])=O)nc1CN(C)C	C12H21N5O2S2	331.457	Nizatidine
Selleck Chemicals	S1214	S([O-])(O)(=O)=O.[C@@H](NC(=O)c1c(C)c(N)nc([C@H](CC(=O)N)NC[C@@H](N)C(=O)N)n1)(C(=O)N[C@@H](C)[C@H](O)[C@@H](C)C(=O)N[C@@H](C(=O)NCCc2scc(c3scc(C(=O)NCCC[S+](C)C)n3)n2)[C@H](O)C)C(O[C@H]4O[C@H](CO)[C@H](O)[C@@H](O)[C@@]4([H])O[C@H]5O[C@H](CO)[C@@H](O)[C@H](OC(=O)N)[C@@H]5O)c6c[nH]cn6	C55H85N17O25S4	1512.622	Bleomycin Sulfate
Selleck Chemicals	S1958	C1(=O)N2[C@]([H])(S(=O)(=O)C(C)(C)[C@]2(C(=O)O)[H])C1	C8H11NO5S	233.242	Sulbactam

Selleck Chemicals	S1284	N1(CC(=O)O)c2c(cccc2)CC[C@H](N[C@@H](CCc3ccccc3)C(=O)OCC)C1=O.Cl	C24H29ClN2O5	460.95	Benazepril HCl
Selleck Chemicals	S2017	c1(cccc([N+]([O-])=O)c1)[C@@H]2C(C(=O)OC)=C(C)NC(C)=C2C(=O)O[C@H]3CN(Cc4ccccc4)CCC3.Cl	C28H32ClN3O6	542.023	Benidipine HCl
Selleck Chemicals	S1351	[C@](O)(C1=CC=C[C@H](C)[C@H](O[C@]2([H])O[C@@H](C)[C@H](O[C@]3([H])O[C@@H](C)[C@H](O)[C@@H](OC)C3)[C@@H](OC)C2)C(C)=CC[C@]([H])(C[C@@H]4OC5=O)O[C@@]6(CC[C@H](C)[C@@]([H])([C@H](CC)C)O6)C4)([C@]5([H])C=C(C)[C@H]7O)[C@]7([H])OC1	C48H74O14	875.093	Ivermectin
Selleck Chemicals	S2069	NC(=N)NCCC[C@]([H])(NS(=O)(=O)c1c2c(CC(C)CN2)ccc1)C(=O)N3[C@H](C(O)=O)C[C@H](C)CC3	C23H36N6O5S	508.634	Argatroban
Selleck Chemicals	S1394	C1(=C2CCN(C)CC2)c3c(cccc3)CCc4c1ccs4.C(O)(=O)C(O)CC(O)=O	C23H27NO5S	429.529	Pizotifen Malate
Selleck Chemicals	S2099	c1cc(CC[C@@H](C(=O)OCC)N[C@H]2CS[C@H](c3cccs3)CN(CC(=O)O)C2=O)ccc1.Cl	C23H29ClN2O5S2	513.07	Temocapril HCl
Selleck Chemicals	S1578	c1(Cn2c(OCC)nc3c2c(C(=O)O)ccc3)ccc(c4c(c5n[nH]nn5)cccc4)cc1	C24H20N6O3	440.454	Candesartan
Selleck Chemicals	S2160	c1cccc([C@H](N2[C@@H](CCc3ccc(C(F)(F)F)cc3)c4c(cc(OC)c(OC)c4)CC2)C(=O)NC)c1.Cl	C29H32ClF3N2O3	549.024	Almorexant HCl
Selleck Chemicals	S1640	c1(SCCC)cc2c(nc(NC(=O)OC)[nH]2)cc1	C12H15N3O2S	265.331	Albendazole
Selleck Chemicals	S2228	c1(C(=O)N[C@@H](C(C)(C)C)C(N2CCC[C@H]2C(=O)N[C@@H]3[C@@H](OCC)OC(=O)C3)=O)ccc(N)c(Cl)c1	C24H33ClN4O6	508.995	VX-765
Selleck Chemicals	S1703	C(CCC)(CCC)C(=O)[O-].[Na+].C(O)(=O)C(CCC)CCC	C16H31NaO4	310.405	Divalproex Sodium
Selleck Chemicals	S2303	c1(C=O)c2c(cc(C)c(c3c(C)cc4c(c(C=O)c(O)c(O)c4C(C)C)c3O)c2O)c(C(C)C)c(O)c1O	C30H30O8	518.554	Gossypol
Selleck Chemicals	S1771	c1cc2c(C(=CCCN(C)C)c3c(ccc(Cl)c3)S2)cc1	C18H18ClNS	315.86	Chlorprothixene
Selleck Chemicals	S2329	c1c(O)c2c(O[C@H](c3ccc(O)cc3)CC2=O)cc1O[C@H]4[C@@H](O[C@H]5[C@@H](O)[C@H](O)[C@@H](O)[C@H](C)O5)[C@@H](O)[C@H](O)[C@@H](CO)O4	C27H32O14	580.535	Naringin
Selleck Chemicals	S1832	c1(OC)cc2c(C(Cc3cc(OC)c(OC)cc3)[N+](C)(CCC(=O)OCCCCCOC(=O)CC[N+]4(C)CCc(c5C4Cc6cc(OC)c(OC)cc6)cc(OC)c(OC)c5)CC2)cc1OC.c7c(S(=O)(=O)[O-])cccc7.c8c(S(=O)(=O)[O-])cccc8	C65H82N2O18S2	1243.479	Atracurium Besylate
Selleck Chemicals	S2369	c1c(O)c2c(OC(c3ccc(OCCO)c(OCCO)c3)=C(O[C@H]4[C@H](O)[C@@H](O)[C@H](O)[C@H](CO[C@@H]5O[C@@H](C)[C@H](O)[C@@H](O)[C@H]5O)O4)C2=O)cc1OCCO	C33H42O19	742.675	Troxerutin
Selleck Chemicals	S2397	c1cc2c(c[n+]3c(c4c(cc(OC)c(OC)c4)CC3)c2)c(OC)c1OC.[Cl-]	C21H22ClNO4	387.857	Palmatine chloride
Selleck Chemicals	S2874	c1(ccc(NC(=N)N)cc1)C(=O)Oc2ccc(CC(=O)OCC(=O)N(C)C)cc2.S(=O)(=O)(O)C	C21H26N4O8S	494.518	Camostat Mesilate
Selleck Chemicals	S2454	c1c(C)c(NC(=O)C2CCCCN2CCCC)c(C)cc1.Cl	C18H29ClN2O	324.889	Bupivacaine HCl
Selleck Chemicals	S2890	c1ccc(CNc2nc(Nc3cc4c(NC(=O)C4)cc3)ncc2C(F)(F)F)c(N(S(C)(=O)=O)C)n1	C21H20F3N7O3S	507.489	PF-562271
Selleck Chemicals	S2538	CC(Nc1ccccc1)=O	C8H9NO	135.163	Acetanilide
Selleck Chemicals	S2901	c1(F)ccc(CN2C(=O)SN(c3ccc(C)cc3)C2=O)cc1	C16H13FN2O2S	316.35	CCG 50014
Selleck Chemicals	S2590	c1(CC)cnc(CCOc2ccc(CC3SC(=O)NC3=O)cc2)cc1	C19H20N2O3S	356.439	Pioglitazone
Selleck Chemicals	S2911	c1(OC)cc2c(n(CCCN(C)C)cc2C3=C(c4c[nH]c5c4cccc5)C(=O)NC3=O)cc1	C26H26N4O3	442.51	Go 6983
Selleck Chemicals	S2693	c1cc(CN(C)C)ccc1S(=O)(=O)n2cc(C=CC(=O)NO)cc2	C16H19N3O4S	349.405	Resminostat
Selleck Chemicals	S2921	c1(cnn(C)c1c2ccc(C)cc2)c3nn(C)c4c3c(N5CC[C@H](N6CCCCC6)C5)ncn4	C26H32N8	456.586	PF-4981517
Selleck Chemicals	S2806	c1(N2CCN(C)CC2)cc(Nc3nn4c(c(c5ccc(S(=O)(C)=O)cc5)ccc4)n3)ccc1	C24H26N6O2S	462.567	CEP-33779
Selleck Chemicals	S3002	c1cc(N2CCOCC2=O)ccc1N3CC(CNC(=O)c4ccc(Cl)s4)OC3=O	C19H18ClN3O5S	435.881	Rivaroxaban
Selleck Chemicals	S2821	[C@@]([H])(C(=O)O)(Cc1c[nH]c2c1cccc2)N3C(=O)c(c4C3=O)cccc4	C19H14N2O4	334.325	RG108
Selleck Chemicals	S3042	c(n(C1CCCCC1)cn2)(nc(Oc3cccc4c3cccc4)nc5Nc6ccc(N7CCOCC7)cc6)c25	C31H32N6O2	520.625	Purmorphamine
Selleck Chemicals	S2843	c1(O)c(F)cc(Nc2ncc3c(N(CCC(C)C)C(C)C(=O)N3C)n2)cc1F	C19H23F2N5O2	391.415	BI-D1870

Selleck Chemicals	S3053	C(N)(NC(C)=C(C(OC(C)C)=O)C1c2cccc([N+]([O-])=O)c2)=C1C(OC3CN(C(c4ccccc4)c5ccccc5)C3)=O	C33H34N4O6	582.646	Azelnidipine
Selleck Chemicals	S2856	c(ccc(Cl)c1)(sc(S(Nc2ccc(OC)c(N3CCNCC3)c2)(=O)=O)c4C)c14	C20H22ClN3O3S2	451.99	SB271046
Selleck Chemicals	S3071	C(=O)c1ccc(O)c(OC)c1	C8H8O3	152.147	Vanillin
Selleck Chemicals	S2864	c1c(O)c(C(=O)Nc2cc(C(F)(F)F)cc(C(F)(F)F)c2)cc(Cl)c1	C15H8ClF6NO2	383.673	IMD 0354
Selleck Chemicals	S3083	N1c(c2C=CC1=O)c(O)ccc2[C@@H](O)CNC3Cc(c4C3)cc(CC)c(CC)c4.C(=CC(O)=O)C(O)=O	C28H32N2O7	508.563	Indacaterol Maleate
Selleck Chemicals	S1018	c1c2c(C(N)=C(c3nc4c(cc(N5CCN(C)CC5)cc4)[nH]3)C(=O)N2)c(F)cc1	C21H21FN6O	392.429	Dovitinib (TKI-258, CHIR-258)
Selleck Chemicals	S1894	c1(CN(C(CCCC)=O)[C@]([H])(C(O)=O)C(C)C)ccc(c2c(c3n[nH]nn3)cccc2)cc1	C24H29N5O3	435.519	Valsartan
Selleck Chemicals	S1216	c1(NS(c2ccccc2OC)(=O)=O)cc3c(NC(=O)N(C)C3)cc1	C16H17N3O4S	347.389	PFI-1 (PF-6405761)
Selleck Chemicals	S1959	c1(ccccc1C(=O)O)Nc2c(C)c(Cl)ccc2	C14H12ClNO2	261.704	Tolfenamic Acid
Selleck Chemicals	S1293	c1c(C2C(C(=O)OCCOC)=C(C)NC(C)=C2C(=O)OCC=Cc3ccccc3)cc([N+]([O-])=O)cc1	C27H28N2O7	492.52	Cilnidipine
Selleck Chemicals	S2020	OC(=O)C=CC(=O)O.c1cc(C[C@H](C)NC[C@@H](O)c2cc(NC=O)c(O)cc2)ccc1OC.c3cc(C[C@H](C)NC[C@@H](O)c4cc(NC=O)c(O)cc4)ccc3OC	C42H52N4O12	804.882	Formoterol Hemifumarate
Selleck Chemicals	S1353	c1(N2CCN(C(C)=O)CC2)ccc(OC[C@H]3CO[C@@](c4c(Cl)cc(Cl)cc4)(Cn5cncc5)O3)cc1	C26H28Cl2N4O4	531.431	Ketoconazole
Selleck Chemicals	S2079	c1(OC)cc2c(CN(C([C@@H](N[C@@H](CCc3ccccc3)C(OCC)=O)C)=O)[C@H](C(=O)O)C2)cc1OC.Cl	C27H35ClN2O7	535.029	Moexipril HCl
Selleck Chemicals	S1432	c1(CS(=O)(=O)NC)cc2c([nH]cc2CCN(C)C)cc1.OC(=O)CCC(=O)O	C18H27N3O6S	413.488	Sumatriptan Succinate
Selleck Chemicals	S2102	c1cc2c(CC[C@@H]2NCC#C)cc1.O=S(O)(C)=O	C13H17NO3S	267.344	Rasagiline Mesylate
Selleck Chemicals	S1604	n1(c(C(=O)OCC2=C(C)OC(=O)O2)c(C(C)(C)O)nc1CCC)Cc3ccc(c4c(c5nn[nH]n5)cccc4)cc3	C29H30N6O6	558.585	Olmesartan Medoxomil
Selleck Chemicals	S2163	[nH]1c2c(cc(C(F)(F)F)cc2)nc1CN3CCN(c4c(CC)cncn4)CC3	C19H21F3N6	390.405	PF-4708671
Selleck Chemicals	S1649	c1c2c([nH]cc2CCN(C)C)ccc1C[C@H]3COC(=O)N3	C16H21N3O2	287.357	Zolmitriptan
Selleck Chemicals	S2233	c1(OC)cc2c([n-]c(S(=O)Cc3c(C)c(OC)c(C)cn3)n2)cc1.[Na+]	C17H18N3NaO3S	367.398	Esomeprazole Sodium
Selleck Chemicals	S1716	C1CC(NC(=O)NS(=O)(=O)c2ccc(CCNC(=O)c3cc(Cl)ccc3OC)cc2)CCC1	C23H28ClN3O5S	494.004	Glyburide
Selleck Chemicals	S2304	c(c(CN(C)C)c[nH]1)(cccc2)c12	C11H14N2	174.242	Gramine
Selleck Chemicals	S1776	c1cc(C(=C(CCCl)c2ccccc2)c3ccccc3)ccc1OCCN(C)C.OC(=O)CC(O)(C(O)=O)CC(O)=O	C32H36ClNO8	598.083	Toremifene Citrate
Selleck Chemicals	S2332	c1c(O)c2c(O[C@H](c3cc(O)cc(OC)c3)CC2=O)cc1O[C@H]4[C@@H](O[C@H]5[C@@H](O)[C@H](O)[C@@H](O)[C@H](C)O5)[C@@H](O)[C@H](O)[C@@H](CO)O4	C28H34O15	610.561	Neohesperidin
Selleck Chemicals	S1845	c1(C)[nH]cnc1CSCCNC(NC)=NC#N	C10H16N6S	252.339	Cimetidine
Selleck Chemicals	S2371	c1cc(CCC(=O)C)cc(OC)c1O	C11H14O3	194.227	Vanillylacetone
Selleck Chemicals	S2411	[C@H]1(O)[C@@H](O)[C@@H](O)[C@H](O[C@H]2[C@]3([H])[C@]([H])(CC=C3CO)C(C(OC)=O)=CO2)O[C@@H]1CO	C17H24O10	388.366	Geniposide
Selleck Chemicals	S2875	c1(C(=O)NC2CCN(CCCOC)CC2)cc(Cl)c(N)c3c1OCC3	C18H26ClN3O3	367.87	Prucalopride
Selleck Chemicals	S2491	c1cc([N+](=O)[O-])cc(C2C(C(OC)=O)=C(C)NC(C)=C2C(OCC)=O)c1	C18H20N2O6	360.361	Nitrendipine
Selleck Chemicals	S2892	c1(C(Nc2ccc(Cl)c(OC)c2)=O)ncccc1	C13H11ClN2O2	262.692	VU 0361737
Selleck Chemicals	S2550	c1cc(O)c([C@H](CCN(C(C)C)C(C)C)c2ccccc2)cc1C.O=C([C@H](O)[C@@H](O)C(=O)O)O	C26H37NO7	475.574	Tolterodine tartrate
Selleck Chemicals	S2902	n1cnc(c2cnn([C@@H](CC#N)C3CCCC3)c2)c4c1[nH]cc4	C17H18N6	306.365	S-Ruxolitinib (INCB018424)
Selleck Chemicals	S2610	c1cc2c(c(C(=O)O)nn2Cc3ccc(Cl)cc3Cl)cc1	C15H10Cl2N2O2	321.158	Lonidamine
Selleck Chemicals	S2912	c1(CNc2ncccc2)ccc(CNc3ncccc3)cc1	C18H18N4	290.362	WZ811

Selleck Chemicals	S2694	c1cc2c([nH]c3c2C(C)(C)CN(C(=O)c4cc(F)c(F)cc4)C=C3C(OC(C)C)=O)cc1	C25H24F2N2O3	438.466	XL335
Selleck Chemicals	S2922	c1c(Nc2c3c(cc4c(OCCOCCOCCO4)c3)ncn2)cccc1C#C	C22H21N3O4	391.42	Icotinib
Selleck Chemicals	S2809	c1(C)nc(C#Cc2ccccc2)ccc1	C14H11N	193.244	MPEP
Selleck Chemicals	S3015	[C@H]1(NC([C@H](N)c2ccc(O)cc2)=O)[C@@]3([H])N([C@@H](C(O)=O)C(C)(C)S3)C1=O	C16H19N3O5S	365.404	Amoxicillin
Selleck Chemicals	S2822	c1(F)cc2c(n(CC(=O)O)c(C)c2Cc3nc4c(cccc4)cc3)cc1	C21H17FN2O2	348.37	OC000459
Selleck Chemicals	S3043	c1cc(C(COC2=O)=C2c3ccccc3)ccc1S(=O)(=O)C	C17H14O4S	314.356	Rofecoxib
Selleck Chemicals	S2845	c1cc2c(NC(=O)C2=Cc3c(C)cc(C)[nH]3)cc1	C15H14N2O	238.284	Semaxanib (SU5416)
Selleck Chemicals	S3054	c1(CCCN(CC)CCCc2ccccc2)ccccc1.C(C(O)=O)C(O)(C(O)=O)CC(O)=O	C26H35NO7	473.559	Alverine Citrate
Selleck Chemicals	S2857	c(cccc1)(C[C@@H](N[C@]23C)c4c2cccc4)c13	C16H15N	221.297	MK-801 (Dizocilpine)
Selleck Chemicals	S3073	[C@@H]1(O)C[C@]2([H])N(C(=O)[C@]([H])([C@H](O)C)NC(=O)[C@@H](NC(=O)CCCCCCCC[C@H](C)C[C@H](C)CC)C[C@@H](O)[C@@H](NCCN)NC(=O)[C@@]3([H])N(CC[C@@H]3O)C(=O)[C@@]([H])([C@@H](O)CCN)NC(=O)[C@]([H])([C@@H]([C@H](c4ccc(O)cc4)O)O)NC2=O)C1.CC(O)=O.CC(O)=O	C56H96N10O19	1213.417	Caspofungin Acetate
Selleck Chemicals	S2865	c1cc2c(nc(Cl)c(N3CCN(C)CC3)n2)cc1	C13H15ClN4	262.738	VUF 10166
Selleck Chemicals	S3100	N1C=CC(=O)NC1=S	C4H4N2OS	128.152	2-Thiouracil
Selleck Chemicals	S1061	N1(C(N2CC(=O)NCC2)=O)C(c3ccc(OC)cc3OC(C)C)=NC(c4ccc(Cl)cc4)C1c5ccc(Cl)cc5	C30H30Cl2N4O4	581.49	Nutlin-3
Selleck Chemicals	S1897	[K+].[I-]	IK	166.003	Potassium Iodide
Selleck Chemicals	S1223	c1c(OC)c2c(C(=O)c3c(c(O)c4c(C[C@@](O)(C(=O)CO)C[C@H]4O[C@]5([H])O[C@@H](C)[C@H](O)[C@@H](N)C5)c3O)C2=O)cc1.Cl	C27H30ClNO11	579.98	Epirubicin HCl
Selleck Chemicals	S1960	c1cc2c(Oc3c(cc(C(C)C(=O)O)cc3)C2)nc1	C15H13NO3	255.269	Pranoprofen
Selleck Chemicals	S1305	N(C(=S)c1c(nc[nH]1)N2)=C2	C5H4N4S	152.177	Mercaptopurine (6-MP)
Selleck Chemicals	S2025	N1(C)C(=O)C=C(NCCCN2CCN(c3c(OC)cccc3)CC2)N(C)C1=O.Cl	C20H30ClN5O3	423.937	Urapidil HCl
Selleck Chemicals	S1357	c1c(C)c(NC(=O)CN(CC)CC)c(C)cc1	C14H22N2O	234.337	Lidocaine
Selleck Chemicals	S2080	CCCC(=O)OCOC(=O)C(C(c1c(Cl)c(Cl)ccc1)C(C(=O)OC)=C(C)N2)=C2C	C21H23Cl2NO6	456.316	Clevidipine Butyrate
Selleck Chemicals	S1436	c(cccc1)(N(C)S(=O)(=O)c2c(ccc(Cl)c2)C3NCCCCCCC([O-])=O)c13.[Na+]	C21H24ClN2NaO4S	458.934	Tianeptine sodium
Selleck Chemicals	S2104	c1c(S(=O)(N)=O)cc(C(NC[C@@H]2CCCN2CC)=O)c(OC)c1	C15H23N3O4S	341.426	Levosulpiride
Selleck Chemicals	S1608	c1[n+](C)cccc1OC(=O)N(C)C.[Br-]	C9H13BrN2O2	261.116	Pyridostigmine Bromide
Selleck Chemicals	S2168	c1(O)cc2c(OC[C@@]3([H])[C@]2([H])OCCN3CCC)cc1	C14H19NO3	249.306	PD128907 HCl
Selleck Chemicals	S1654	N1(c2ccccc2)C(=O)C(CCCC)C(=O)N1c3ccccc3	C19H20N2O2	308.374	Phenylbutazone
Selleck Chemicals	S2240	c1ccccc1[C@H](c2cc(CO)ccc2OC(=O)C(C)C)CCN(C(C)C)C(C)C.C(C(=O)O)=CC(=O)O	C30H41NO7	527.649	Fesoterodine Fumarate
Selleck Chemicals	S1727	C1C[C@@]2([H])C(CC[C@]3([H])[C@@]2([H])CC[C@@]4(CC)[C@@]3([H])CC[C@@]4(C#C)O)=CC1=O	C21H28O2	312.446	Levonorgestrel
Selleck Chemicals	S2306	C1C[C@@]2(C)[C@@]([H])(CC[C@]3(C)[C@@]2([H])C[C@@H](O)[C@@]4([H])[C@@]3(C)CC[C@H]4[C@@](CCC=C(C)C)(C)O[C@H]5[C@@H](O)[C@@H](O)[C@H](O)[C@@H](CO[C@H]6[C@@H](O)[C@@H](O)[C@@H](O)CO6)O5)C(C)(C)[C@H]1O[C@H]7[C@@H](O)[C@@H](O)[C@H](O)[C@@H](CO)O7	C47H80O17	917.128	Gynostemma Extract
Selleck Chemicals	S1782	[C@H]1(O)[C@H](O)[C@H](N2C=NC(N)=NC2=O)O[C@@H]1CO	C8H12N4O5	244.205	Azacitidine
Selleck Chemicals	S2333	c1(OC)c(OC)c2c(C(=O)C=C(c3ccc(OC)c(OC)c3)O2)c(OC)c1OC	C21H22O8	402.395	Nobiletin
Selleck Chemicals	S1854	c1cc(C(n2ccnc2)c3ccc(c4ccccc4)cc3)ccc1	C22H18N2	310.392	Bifonazole

Selleck Chemicals	S2372	c1cc2c(Oc3c(cccc3)C2=O)cc1	C13H8O2	196.201	Xanthone
Selleck Chemicals	S2425	c1(OC)c(O)ccc(C(=O)C)c1	C9H10O3	166.174	Apocynin
Selleck Chemicals	S2876	[C@]1(C)(N[C@]2([H])c3c1cccc3)c4c(cccc4)C2.C(C(=O)O)=CC(=O)O	C20H19NO4	337.369	(-)-MK 801 Maleate
Selleck Chemicals	S2516	c1c(C)c(NC2=NCCCS2)c(C)cc1.Cl	C12H17ClN2S	256.795	Xylazine HCl
Selleck Chemicals	S2894	c1c(S(=O)(=O)c2cc(c3nc2)cccc3N4CCNCC4)cccc1	C19H19N3O2S	353.438	SB742457
Selleck Chemicals	S2551	C1(=O)N2[C@]([H])(S(=O)(=O)C(C)(C)[C@]2(C(=O)[O-])[H])C1.[Na+]	C8H10NNaO5S	255.223	Sulbactam sodium
Selleck Chemicals	S2903	c1c(Cl)c(Nc2c(CC(=O)O)cc(C)cc2)c(F)cc1	C15H13ClFNO2	293.721	Lumiracoxib
Selleck Chemicals	S2625	c1(OC)cc(Nc2nc(Nc3nc4c(OC(C)(C)C(=O)N4COP(=O)(O)O)cc3)c(F)cn2)cc(OC)c1OC	C23H26FN6O9P	580.46	Fostamatinib (R788)
Selleck Chemicals	S2913	c1(ccc(C)cc1)S(=O)(=O)C=CC#N	C10H9NO2S	207.249	BAY 11-7082
Selleck Chemicals	S2698	c1c(c2nc(N)nc(C(C)C)c2)c3c(cccc3)c(F)c1	C17H16FN3	281.327	RS-127445
Selleck Chemicals	S2923	c1(nccc2)c2cccc1NC(=S)NC(C(Cl)(Cl)Cl)NC(=O)C=Cc3ccccc3	C21H17Cl3N4OS	479.81	Salubrinal
Selleck Chemicals	S2812	c1(O)c(c2cc(C)c1c3c(C)cc(c4c3O)c(C(C)C)c(O)c(O)c4C=O)c(C=O)c(O)c(O)c2C(C)C.C(O)(=O)C	C32H34O10	578.606	AT101
Selleck Chemicals	S3033	C1CC[C@@H](C#N)N1C(=O)CNC2(CC(O)(CC3C2)CC4C3)C4	C17H25N3O2	303.399	Vildagliptin (LAF-237)
Selleck Chemicals	S2824	c1(NC(N)=O)sc(c2ccc(F)cc2)cc1C(N)=O	C12H10FN3O2S	279.29	TPCA-1
Selleck Chemicals	S3045	N1(CC(N2CCCC2)=O)CCN(C(=O)C=Cc3cc(OC)c(OC)c(OC)c3)CC1.C(=O)(O)C=CC(=O)O	C26H35N3O9	533.571	Cinepazide maleate
Selleck Chemicals	S2847	n1(C)c(C(N[C@@H](Cc2cccc(Cl)c2)C(NCC#N)=O)=O)cc(C(C)(C)C)n1	C20H24ClN5O2	401.89	Cathepsin Inhibitor 1
Selleck Chemicals	S3057	c1(OCC)n(Cc2ccc(c3ccccc3C4=NC(=O)ON4)cc2)c5c(cccc5C(OCC6=C(C)OC(=O)O6)=O)n1	C30H24N4O8	568.534	Azilsartan Medoxomil
Selleck Chemicals	S2858	c1(O)ccc(CCNc2c(c3nc(c4csc5c4cccc5)n2)ncn3C(C)C)cc1	C24H23N5OS	429.537	StemRegenin 1 (SR1)
Selleck Chemicals	S3075	c1ccc(C)c(C)c1[C@H](C)c2cnc[nH]2	C13H16N2	200.28	Dexmedetomidine
Selleck Chemicals	S2866	N(c1ccc(F)cc1)C(=O)Nc2ccc(S(=O)(=O)N)cc2	C13H12FN3O3S	309.316	U-104
Selleck Chemicals	S3104	c1cccc(OC)c1OCC2SCCN2C(CC(=O)OCC)=O	C16H21NO5S	339.407	Moguisteine
Selleck Chemicals	S1121	c1c(C(C)(C)C)c(S(=O)(=O)c2ccc(NC(=O)c3cc(Cc4c(C(C)C)cccc4)c(O)c(O)c3O)cc2)ccc1	C33H35NO6S	573.699	TW-37
Selleck Chemicals	S1905	N1C(C)=C(C(OC)=O)C(c2ccccc2Cl)C(C(=O)OCC)=C1COCCN	C20H25ClN2O5	408.876	Amlodipine
Selleck Chemicals	S1228	c1cc2c(C(=O)c3c(c(O)c4c(C[C@@](O)(C(=O)C)C[C@H]4O[C@H]5C[C@H](N)[C@H](O)[C@H](C)O5)c3O)C2=O)cc1.Cl	C26H28ClNO9	533.955	Idarubicin HCl
Selleck Chemicals	S1972	c1(OCCN(C)C)ccc(C(c2ccccc2)=C(c3ccccc3)CC)cc1.C(O)(=O)CC(O)(C(O)=O)CC(O)=O	C32H37NO8	563.638	Tamoxifen Citrate
Selleck Chemicals	S1319	C1C=C(C)CC[C@@H]2[C@H](OC(=O)C2=C)C=C(C)C1	C15H20O2	232.318	Costunolide
Selleck Chemicals	S2037	C1CC(OC(=O)OC(C)OC(c2c3c(nc(OCC)n3Cc4ccc(c5ccccc5c6[nH]nnn6)cc4)ccc2)=O)CCC1	C33H34N6O6	610.66	Candesartan Cilexetil
Selleck Chemicals	S1359	c1c(c2ccccc2c3nnn[nH]3)ccc(Cn4c(CCCC)nc(Cl)c4CO)c1.[K]	C22H23ClKN6O	462.009	Losartan Potassium (DuP 753)
Selleck Chemicals	S2081	c1ccc(CC[C@@H](C(OCC)=O)N[C@@H]2C(=O)N3N(CCC[C@@H]3C(=O)O)CCC2)cc1.O	C22H33N3O6	435.514	Cilazapril Monohydrate
Selleck Chemicals	S1468	C1C(=CC=C2CCC[C@@]3(C)[C@@]2([H])CC[C@@H]3[C@H](C)CCCC(C)C)C(=C)[C@@H](O)C[C@@H]1O	C27H44O2	400.637	Alfacalcidol
Selleck Chemicals	S2108	c1(Nc2cccc(C(F)(F)F)c2C)ncccc1C(O)=O.N(C)C[C@@H]([C@H]([C@@H]([C@@H](CO)O)O)O)O	C21H28F3N3O7	491.458	Flunixin Meglumin
Selleck Chemicals	S1609	N1C(=S)N(C)C=C1	C4H6N2S	114.169	Methimazole
Selleck Chemicals	S2169	[Ca+2].n1c(C(C)C)c(C=C[C@H](C[C@@H](O)CC(=O)[O-])O)c(c2ccc(F)cc2)nc1N(C)S(=O)(=O)C.n3c(C(C)C)c(C=C[C@H](C[C@@H](O)CC(=O)[O-])O)c(c4ccc(F)cc4)nc3N(C)S(=O)(=O)C	C44H54CaF2N6O12S	1001.137	Rosuvastatin Calcium

Selleck Chemicals	S1657	c1cc(CC[C@H](N[C@@H](C)C(=O)N2CCC[C@H]2C(O)=O)C(=O)O)ccc1.O.O	C18H28N2O7	384.424	Enalaprilat Dihydrate
Selleck Chemicals	S2250	c1(O)cc(C(=O)O[C@@H]2Cc3c(cc(O)cc3O)O[C@@H]2c4cc(O)c(O)c(O)c4)cc(O)c1O	C22H18O11	458.372	(-)-Epigallocatechin Gallate
Selleck Chemicals	S1740	c1ccc(OCC(O)CO)c(OC)c1	C10H14O4	198.216	Guaifenesin
Selleck Chemicals	S2313	c1c2c([nH]cc2CO)ccc1	C9H9NO	147.174	Indole-3-carbinol
Selleck Chemicals	S1801	N(C)(C)Cc1oc(CSCCNC(NC)=C[N+]([O-])=O)cc1.Cl	C13H23ClN4O3S	350.865	Ranitidine
Selleck Chemicals	S2336	C(C(=O)NC(=O)N1)=C1C(=O)O	C5H4N2O4	156.096	Orotic acid (6-Carboxyuracil)
Selleck Chemicals	S1856	[C@@H](O)([C@@H](O)C(=O)O)C(=O)O.c1cc(OCC(O)CNC(C)C)ccc1CCOC.c2cc(OCC(O)CNC(C)C)ccc2CCOC	C34H56N2O12	684.815	Metoprolol Tartrate
Selleck Chemicals	S2376	[C@@]1(C(=O)O)(C)C[C@]2([H])[C@@](C)(CC[C@]3(C)C2=CC(=O)[C@@]4([H])[C@@]3(C)CC[C@]5([H])[C@@]4(C)CC[C@H](O[C@]6([H])[C@@H](O[C@]7([H])O[C@H](C(O)=O)[C@@H](O)[C@H](O)[C@H]7O)[C@@H](O)[C@H](O)[C@@H](C(=O)O)O6)C5(C)C)CC1.N	C42H65NO16	839.963	Ammonium Glycyrrhizinate
Selleck Chemicals	S2439	c1(N=C(N)NC2=O)c2ncn1[C@H]3[C@H](O)[C@H](O)[C@H](CO)O3	C10H13N5O5	283.241	Guanosine
Selleck Chemicals	S2882	c1(C(N2CCC(N3CCCC3)CC2)=O)ccc(Nc4nccc(c5sc6c(cccc6)c5)n4)cc1	C28H29N5OS	483.628	IKK-16 (IKK Inhibitor VII)
Selleck Chemicals	S2519	c1(cccc2)c2c(CC(NCC3)=N3)ccc1.Cl	C14H15ClN2	246.735	Naphazoline HCl
Selleck Chemicals	S2895	c1(C(C)(C)C)cc(C=C(C#N)C#N)cc(C(C)(C)C)c1O	C18H22N2O	282.38	Tyrphostin 9
Selleck Chemicals	S2553	NCC(=O)CCC(=O)O.Cl	C5H10ClNO3	167.591	5-Aminolevulinic acid HCl
Selleck Chemicals	S2904	c1c2c(c3C=NNC2=O)c([nH]c3c4cn(C)nc4)cc1NC(=O)[C@H](N)C5CCCCC5	C22H25N7O2	419.48	PF-477736
Selleck Chemicals	S2637	c1ccc(COc2cc3c([C@H](CC(O)=O)CO3)cc2)cc1c4c(C)cc(OCCCS(=O)(C)=O)cc4C.O.c5ccc(COc6cc7c([C@H](CC(O)=O)CO7)cc6)cc5c8c(C)cc(OCCCS(=O)(C)=O)cc8C	C58H66O15S2	1067.266	TAK-875
Selleck Chemicals	S2914	n1c(Nc2c(C)cc(C)cc2C)ccnc1Nc3ccc(C#N)cc3	C20H19N5	329.398	Dapivirine (TMC120)
Selleck Chemicals	S2721	C1(C)=C(C(OC(C)C)=O)C(c2cc([N+]([O-])=O)ccc2)C(C(=O)OC)=C(C#N)N1	C19H19N3O6	385.371	Nilvadipine
Selleck Chemicals	S2925	c1(C(F)(F)F)cc(CN(c2nn(C)nn2)[C@]3([H])c4c(c(C)cc(C)c4)N(C[C@]5([H])CC[C@@]([H])(C(=O)O)CC5)CCC3)cc(C(F)(F)F)c1	C31H36F6N6O2	638.647	Evacetrapib (LY2484595)
Selleck Chemicals	S2813	C(C1CC1)(c2ccc(OCCCc3c[nH]cn3)cc2)=O.C(O)(=O)C=CC(O)=O	C20H22N2O6	386.398	Ciproxifan
Selleck Chemicals	S3034	c1(C(Cc2c(C)cc(c3c2)OCO3)=O)sccc1S([N-]c4onc(C)c4Cl)(=O)=O.[Na+]	C18H14ClN2NaO6S2	476.886	Sitaxentan sodium
Selleck Chemicals	S2825	c1ccc2c(cccc2CNCc3ccc(OC)cc3)c1.Cl	C19H20ClNO	313.821	ML133 HCl
Selleck Chemicals	S3046	c1(OCC)n(Cc2ccc(c3ccccc3C4=NC(=O)ON4)cc2)c5c(cccc5C(O)=O)n1	C25H20N4O5	456.45	Azilsartan
Selleck Chemicals	S2849	N1(S(c2cccc(O)c2)(=O)=O)CCC[C@@H]1CCN3CCC(C)CC3.Cl	C18H29ClN2O3S	388.952	SB269970 HCl
Selleck Chemicals	S3060	c1(C(C)c2c[nH]cn2)cccc(C)c1C.Cl	C13H17ClN2	236.74	Medetomidine HCl
Selleck Chemicals	S2859	c1c(F)c(NC(=O)C2(CC2)C(=O)Nc3ccc(F)cc3)ccc1Oc4ccnc(NC(=O)N5CCC(N6CCN(C)CC6)CC5)c4	C33H37F2N7O4	633.688	Golvatinib (E7050)
Selleck Chemicals	S3076	P(=O)([O-])([O-])C([O-])=O.[Na+].[Na+].[Na+]	CNa3O5P	191.951	Foscarnet Sodium
Selleck Chemicals	S2867	n1c(c2c(Nc3ccc(O)c(Br)c3)nc1)cc(OC)c(OC)c2	C16H14BrN3O3	376.205	WHI-P154
Selleck Chemicals	S3105	c1(cc(F)c2N3CCC(O)CC3)c(N4C=C(C(O)=O)C1=O)c2CCC4C	C19H21FN2O4	360.379	Nadifloxacin
Selleck Chemicals	S1138	c([nH]c(C)c1)(ccc(Oc2c3n(cc(OC[C@H](OC(=O)[C@H](C)N)C)c3C)ncn2)c4F)c14	C22H24FN5O4	441.455	Brivanib Alaninate (BMS-582664)
Selleck Chemicals	S1913	c1cc(C(C(N(Cc2ccncc2)CC)=O)CO)ccc1	C17H20N2O2	284.353	Tropicamide
Selleck Chemicals	S1235	c1c(C(c2ccc(C#N)cc2)n3cncn3)ccc(C#N)c1	C17H11N5	285.303	Letrozole
Selleck Chemicals	S1973	C(N1C(O[C@H]2[C@H]1O[C@H](CO)[C@@H]2O)=NC3=N)=C3.Cl	C9H12ClN3O4	261.662	Cyclocytidine HCl

Selleck Chemicals	S1322	C1=C[C@@]2(C)C(CC[C@]3([H])[C@@]2(F)[C@H](O)C[C@@]4(C)[C@@]3([H])C[C@@H](C)[C@@]4(O)C(CO)=O)=CC1=O	C22H29FO5	392.461	Dexamethasone (DHAP)
Selleck Chemicals	S2041	c1cc(C(=O)CCN2CCCCC2)ccc1OCCCC.Cl	C18H28ClNO2	325.873	Dyclonine HCl
Selleck Chemicals	S1362	c1(C=CS(=O)(=O)Cc2cc(NCC(=O)[O-])c(OC)cc2)c(OC)cc(OC)cc1OC.[Na+]	C21H24NNaO8S	473.472	Rigosertib (ON-01910)
Selleck Chemicals	S2082	C(=O)(OCCN(CC)CC)C(c1ccccc1)c2ccccc2.Cl	C20H26ClNO2	347.879	Adiphenine HCl
Selleck Chemicals	S1469	C1C(=CC=C2CCC[C@@]3(C)[C@@]2([H])CC[C@@]3([H])[C@H](C)CCCC(O)(C)C)C(=C)CC[C@@H]1O	C27H44O2	400.637	Calcifediol
Selleck Chemicals	S2109	CCOC([C@H](CCc1ccccc1)N[C@@H](C)C(=O)N2[C@@H](C(O)=O)CN(C)C2=O)=O	C20H27N3O6	405.445	Imidapril HCl
Selleck Chemicals	S1611	C1(CSc2n(C)nnn2)=C(C(O)=O)N3[C@@]([H])([C@H](NC([C@H](NC(=O)N4CCN(CC)C(=O)C4=O)c5ccc(O)cc5)=O)C3=O)SC1	C25H27N9O8S2	645.667	Cefoperazone
Selleck Chemicals	S2173	c1cc(c2nc(N)nc(O[C@@H](C(F)(F)F)c3c(n4ccc(C)n4)cc(Cl)cc3)c2)ccc1C[C@H](N)C(=O)OCC.c5cc(C(=O)NCC(=O)O)ccc5	C36H35ClF3N7O6	754.155	Telotristat Etiprate (LX 1606 Hippurate)
Selleck Chemicals	S1675	C1(=O)[C@@H](CCCCCCC(=O)O)[C@@H](CCC(=O)C(F)(F)CCCC)[C@H](O)C1	C20H32F2O5	390.462	Lubiprostone
Selleck Chemicals	S2258	c1(O)cc2c(C=CC(=O)O2)cc1O[C@H]3[C@@H](O)[C@@H](O)[C@H](O)[C@@H](CO)O3	C15H16O9	340.282	Esculin
Selleck Chemicals	S1748	C(C)(NC(C)=C(C(=O)OC)C1c2c([N+]([O-])=O)cccc2)=C1C(=O)OCC(C)C	C20H24N2O6	388.414	Nisoldipine
Selleck Chemicals	S2316	c(nc[nH]1)(ncnc2NCc3ccco3)c12	C10H9N5O	215.211	Kinetin
Selleck Chemicals	S1805	CC(=O)OCC[N+](C)(C)C.[Cl-]	C7H16ClNO2	181.66	Acetylcholine Chloride
Selleck Chemicals	S2350	C(c1ccc(O)c(O)c1)(Oc2c(c(O)cc(O)c2)C3=O)=C3O[C@H]4[C@@H](O)[C@@H](O)[C@H](O)[C@@H](CO[C@H]5[C@@H](O)[C@H](O)[C@@H](O)[C@H](C)O5)O4	C27H30O16	610.518	Rutin
Selleck Chemicals	S1865	c1(cccc2)c2S[C@@H](c3ccc(OC)cc3)[C@@H](OC(=O)C)C(=O)N1CCN(C)C.Cl	C22H27ClN2O4S	450.979	Diltiazem HCl
Selleck Chemicals	S2383	[C@@H]1(O)[C@H](CO)O[C@@H](Oc2ccc(CO)cc2)[C@H](O)[C@H]1O	C13H18O7	286.278	Gastrodin
Selleck Chemicals	S2442	O1[C@H](n2cnc3c2N=CNC3=O)[C@H](O)[C@H](O)[C@H]1CO	C10H12N4O5	268.226	Inosine
Selleck Chemicals	S2883	C(O)(=O)CNC(c1ccc(N)cc1)=O	C9H10N2O3	194.187	4-Aminohippuric Acid
Selleck Chemicals	S2521	c1(O)cc([C@H](CNC)O)ccc1O.C(O)(=O)[C@@H]([C@H](C(O)=O)O)O	C13H19NO9	333.291	Epinephrine Bitartrate
Selleck Chemicals	S2896	c1(O)cc(Nc2c(c3ncn2)ccc(OCc4ccccc4)c3)c(F)cc1C.Cl	C22H19ClFN3O2	411.857	ZM 323881 HCl
Selleck Chemicals	S2573	c1cc(C(=O)OCCN(C)C)ccc1NCCCC.Cl	C15H25ClN2O2	300.824	Tetracaine HCl
Selleck Chemicals	S2905	c1(Cl)cc2c([nH]c(C(=O)N3CCN(C)CC3)c2)cc1	C14H16ClN3O	277.749	JNJ-7777120
Selleck Chemicals	S2662	c1ccc(CNC(=O)N2CCC(=O)N3[C@]2([H])CN(Cc4c5c(cccc5)ccc4)C(=O)[C@H]3Cc6ccc(O)cc6)cc1	C33H32N4O4	548.632	ICG-001
Selleck Chemicals	S2915	c1(C(=O)Nc2ccccc2)c(Cl)ccc([N+]([O-])=O)c1	C13H9ClN2O3	276.675	GW9662
Selleck Chemicals	S2769	N1c(c2C(N)=C(c3[nH]c4c(ccc(N5CCN(C)CC5)c4)n3)C1=O)cccc2F.CC(O)C(=O)O.CC(O)C(=O)O	C27H33FN6O7	572.585	Dovitinib (TKI-258) Dilactic Acid
Selleck Chemicals	S2927	c(C(=O)C(=O)N1Cc2ccc(Cl)c(Cl)c2)(cccc3)c13	C15H9Cl2NO2	306.143	Apoptosis Activator 2
Selleck Chemicals	S2816	C(=S)(N)C(C#N)=Cc1cc(C(C)(C)C)c(O)c(C(C)(C)C)c1	C18H24N2OS	316.461	Tyrphostin AG 879
Selleck Chemicals	S3035	c1c(OC)c2c(C(=O)c3c(c(O)c4c(C[C@](C(=O)C)(O)C[C@H]4O[C@]5([H])O[C@@H](C)[C@@H](O)[C@@H](N)C5)c3O)C2=O)cc1.Cl	C27H30ClNO10	563.981	Daunorubicin HCl
Selleck Chemicals	S2828	N1(c2snc(c3ccccc3)n2)CCN(C(Nc4ccccc4)=O)CC1	C19H19N5OS	365.452	JNJ-1661010
Selleck Chemicals	S3047	C([N+](CC)(CC)C)COC(=O)c1ccc(NC(=O)c2ccccc2OCCCCCCCC)cc1.[Br-]	C29H43BrN2O4	563.567	Otilonium Bromide
Selleck Chemicals	S2851	CCS(N1CC(n2ncc(c3ncnc(c34)[nH]cc4)c2)(CC#N)C1)(=O)=O	C16H17N7O2S	371.417	Baricitinib (LY3009104, INCB028050)
Selleck Chemicals	S3063	c1c(Cl)c(Nc2c(CC(=O)O)cccc2)c(Cl)cc1.N(CC)CC	C18H22Cl2N2O2	369.285	Diclofenac Diethylamine
Selleck Chemicals	S2860	C(N)CCCCNCC1(CC(CC2C1)CC3C2)C3.Br.Br	C16H32Br2N2	412.247	IEM 1754 dihydrobroMide

Selleck Chemicals	S3078	C1=C[C@@]2(C)C(CC[C@]3([H])[C@@]2(Cl)[C@H](O)C[C@@]4(C)[C@@]3([H])C[C@H](C)[C@@]4(OC(CC)=O)C(=O)COC(CC)=O)=CC1=O	C28H37ClO7	521.042	Beclomethasone dipropionate
Selleck Chemicals	S2868	C(#N)c1ccccc1CN2C(=O)N(C)C(=O)C=C2N3C[C@H](N)CCC3	C18H21N5O2	339.392	Alogliptin
Selleck Chemicals	S3106	S1C[C@@H](C(O)=O)N(C([C@@H]2CCC(=O)N2)=O)C1	C9H12N2O4S	244.268	Pidotimod
Selleck Chemicals	S1148	c1c(C(O[C@@H]2C([C@@]3(C)C(=O)[C@H](O)C(C([C@@]24O)(C)C)=C(C)[C@H](OC(=O)[C@H](O)[C@@H](NC(=O)OC(C)(C)C)c5ccccc5)C4)[C@]6(OC(=O)C)[C@H](OC6)C[C@@H]3O)=O)cccc1	C43H53NO14	807.879	Docetaxel
Selleck Chemicals	S1921	c1cc2c(C(=O)C(c3ccccc3)C2=O)cc1	C15H10O2	222.239	Phenindione
Selleck Chemicals	S1252	N(C(=O)c1c(n([C@@H]2C(=C)[C@H](CO)[C@@H](O)C2)cn1)N3)=C3N.O	C12H17N5O4	295.294	Entecavir Hydrate
Selleck Chemicals	S1983	c([nH]cn1)(ncnc2N)c12.Cl	C5H6ClN5	171.588	Adenine HCl
Selleck Chemicals	S1328	c1cc(CC)c2c(c3c([C@](CC)(CC(O)=O)OCC3)[nH]2)c1	C17H21NO3	287.354	Etodolac
Selleck Chemicals	S2046	c1(CC)cnc(CCOc2ccc(CC3SC(=O)NC3=O)cc2)cc1.Cl	C19H21ClN2O3S	392.9	Pioglitazone HCl
Selleck Chemicals	S1374	C1(=O)N2[C@@]([H])([C@H](C)C(S[C@H]3C[C@@H](CNS(=O)(=O)N)NC3)=C2C(O)=O)[C@]1([H])[C@@H](O)C.O	C15H26N4O7S2	438.52	Doripenem Hydrate
Selleck Chemicals	S2085	c1(OC)cc(C(=O)OCC(N(C)C)(CC)c2ccccc2)cc(OC)c1OC	C22H29NO5	387.469	Trimebutine
Selleck Chemicals	S1480	[C@H]1(N(C([C@H]2CC[C@H](C)CC2)=O)c3cc(C#CC(C)(C)C)sc3C(=O)O)CC[C@@H](O)CC1	C25H35NO4S	445.615	VX-222 (VCH-222, Lomibuvir)
Selleck Chemicals	S2123	OC[C@@H](O)[C@@H](O)[C@H](O)[C@@H](O)C=O	C6H12O6	180.156	Dextrose
Selleck Chemicals	S1619	c1c(C)c(NC(=O)C(C)NCCC)ccc1	C13H20N2O	220.311	Prilocaine
Selleck Chemicals	S2194	c1(OC)c(OC)cc(Nc2ncc(F)c(Nc3ccc4c(NC(=O)C(C)(C)O4)n3)n2)cc1OC.c5cc(S(=O)(=O)O)ccc5	C28H29FN6O8S	628.629	R406
Selleck Chemicals	S1681	c1c(N)ccc(O)c1C(=O)O	C7H7NO3	153.135	Mesalamine
Selleck Chemicals	S2280	C1[C@](O)(C(O)=O)C[C@@H](OC(=O)C=Cc2ccc(O)c(O)c2)[C@H](O)[C@@H]1O	C16H18O9	354.309	Chlorogenic Acid
Selleck Chemicals	S1759	[Ca+2].c1cc2c(nc(C3CC3)c(C=C[C@H](C[C@@H](O)CC(=O)[O-])O)c2c4ccc(F)cc4)cc1.c5cc6c(nc(C7CC7)c(C=C[C@H](C[C@@H](O)CC(=O)[O-])O)c6c8ccc(F)cc8)cc5	C50H46CaF2N2O8	880.984	Pitavastatin Calcium
Selleck Chemicals	S2317	[C@H]1(O)[C@@H](C)OC(O)[C@H](O)[C@@H]1O.O	C6H14O6	182.172	L-(+)-Rhamnose Monohydrate
Selleck Chemicals	S1808	c1ccc([N+](=O)[O-])c(C2C(C(OC)=O)=C(C)NC(C)=C2C(=O)OC)c1	C17H18N2O6	346.335	Nifedipine
Selleck Chemicals	S2354	C1CC2[C@](C)(CCCC2(C)C)[C@@H](CCC(O)(C=C)C)[C@@]1(O)C	C20H36O2	308.499	Sclareol
Selleck Chemicals	S1880	C(N1CCCCC1)c2cccc(OCCCNC(=O)COC(=O)C)c2.Cl	C19H29ClN2O4	384.898	Roxatidine Acetate HCl
Selleck Chemicals	S2387	C1[C@@H](OC)[C@]2(C3[C@@H]4[C@]5(O)[C@@](O)([C@@H](OC)[C@@]6([H])[C@@H](OC)C5)[C@]2([H])C6)[C@]([H])(C4)[C@](OC(=O)c7ccccc7NC(C)=O)(CN3CC)C1.Br	C32H45BrN2O8	665.612	Lappaconite HBr
Selleck Chemicals	S2443	c1(S(NC(NCCCC)=O)(=O)=O)ccc(C)cc1	C12H18N2O3S	270.348	Tolbutamide
Selleck Chemicals	S2884	O1C(C)=CC(=O)NS1(=O)=O.[K]	C4H5KNO4S	202.25	Acesulfame Potassium
Selleck Chemicals	S2524	N1C([O-])=NC(=O)C1(c2ccccc2)c3ccccc3.[Na+]	C15H11N2NaO2	274.25	Phenytoin sodium
Selleck Chemicals	S2897	c(c(Nc1ccc(Cl)cc1F)ncn2)(cc(OC)c(OC)c3)c23	C16H13ClFN3O2	333.745	ZM 306416
Selleck Chemicals	S2577	c1cc(NC(=O)C)ccc1OCC	C10H13NO2	179.216	Phenacetin
Selleck Chemicals	S2906	c1c(c2onc(C)c2NC(=O)OC(C)c3ccccc3Cl)ccc(CSCCC(=O)OC)c1	C24H25ClN2O5S	488.984	Ki16198
Selleck Chemicals	S2672	c1ccc(CNc2nc(Nc3ccc4c(NC(=O)C4)cc3)ncc2C(F)(F)F)c(N(S(=O)(C)=O)C)n1.c5cc(S(=O)(=O)O)ccc5	C27H26F3N7O6S2	665.664	PF-00562271
Selleck Chemicals	S2916	c1cc(C(=O)Nc2cccc(NC(=O)CCC)c2)c(Br)cc1	C17H17BrN2O2	361.233	ML161
Selleck Chemicals	S2774	c(ncc(c1cnn(C)c1)c2)(C=Cc3c(cc(NS(=O)(=O)N(C)C[C@@H]4COCCO4)c c3)C5=O)c25	C24H25N5O5S	495.551	MK-2461

Selleck Chemicals	S2928	C(Nc1nccc(c2sc(CC)nc2c3cccc(C)c3)c1)(=O)c4ccccc4	C24H21N3OS	399.508	TAK-715
Selleck Chemicals	S2817	N1(c2cccc(C(F)(F)F)c2)c(c3C=CC1=O)c4c(ccc(c5ccc(N)nc5)c4)nc3	C24H15F3N4O	432.397	Torin 2
Selleck Chemicals	S3036	C1[C@@H](OC(=O)[C@H](CC)C)[C@@]2([H])C(C=C[C@H](C)[C@H]2CC[C@@H](O)C[C@@H](O)CC([O-])=O)=C[C@H]1O.[Na+]	C23H35NaO7	446.51	Pravastatin sodium
Selleck Chemicals	S2830	[C@H]1(O)[C@H](SC)O[C@H]([C@@H](NC([C@@H]2C[C@@H](CCC)CN2C)=O)C(Cl)C)[C@H](O)[C@@H]1O	C18H33ClN2O5S	424.983	Clindamycin
Selleck Chemicals	S3048	[C@H]1(c2ccccc2)c(c3CCN1C(O[C@]4([H])C(CCN5C4)CC5)=O)cccc3.C(O)(=O)CCC(O)=O	C27H32N2O6	480.553	Solifenacin succinate
Selleck Chemicals	S2852	[nH]1c(c2c(C3CCN(C)CC3)c1)ccc(O)c2	C14H18N2O	230.306	BRL-54443
Selleck Chemicals	S3064	[C@@H]1(O)CC[C@@H](NCc2cc(Br)cc(Br)c2N)CC1.Cl	C13H19Br2ClN2O	414.564	Ambroxol HCl
Selleck Chemicals	S2861	c1(C#Cc2c(C)n(c3ccc(OC(F)(F)F)cc3)c(C)n2)cc(Cl)ncc1	C19H13ClF3N3O	391.774	CTEP (RO4956371)
Selleck Chemicals	S3079	C1(=C(O)C(=O)c2c(cccc2)C1=O)[C@H]3CC[C@H](c4ccc(Cl)cc4)CC3	C22H19ClO3	366.837	Atovaquone
Selleck Chemicals	S2870	c1(O)cccc(c2cnc(c3n2)nc(N)nc3N)c1	C12H10N6O	254.247	TG100713
Selleck Chemicals	S3113	c1nc(C)c(O)c(CO)c1CO.Cl	C8H12ClNO3	205.639	Pyridoxine HCl
Selleck Chemicals	S1195	c1cc2c(ccc(C3(O)CCn4c3cnc4)c2)cc1C(=O)NC	C18H17N3O2	307.346	TAK-700 (Orteronel)
Selleck Chemicals	S1933	C1=C[C@@]2(C)C(CC[C@]3([H])[C@@]2(F)[C@H](O)C[C@@]4(C)[C@@]3([H])C[C@@H](O)[C@@]4(O)C(=O)CO)=CC1=O	C21H27FO6	394.434	Triamcinolone
Selleck Chemicals	S1256	c1c(F)c(Cn2cc(C(N)=O)nn2)c(F)cc1	C10H8F2N4O	238.194	Rufinamide
Selleck Chemicals	S1994	c1c(C2C(C(=O)OCC)=C(C)NC(C)=C2C(=O)OCC)c(C=CC(OC(C)(C)C)=O)ccc1	C26H33NO6	455.543	Lacidipine
Selleck Chemicals	S1334	c1(F)ccc(CNc2ccc(NC(OCC)=O)c(N)n2)cc1.OC(=O)C=CC(=O)O	C19H21FN4O6	420.392	Flupirtine maleate
Selleck Chemicals	S2051	C(O)(=O)[C@H]1CCCN1C(=O)[C@H](C)CS	C9H15NO3S	217.285	Captopril
Selleck Chemicals	S1385	c1(Cl)cc(C(=O)NCC2CN(Cc3ccc(F)cc3)CCO2)c(OCC)cc1N.OC(=O)CC(O)(C(O)=O)CC(=O)O	C27H33ClFN3O10	614.016	Mosapride Citrate
Selleck Chemicals	S2092	c1(Cc2c[nH]cn2)c(C)c(C)ccc1.Cl	C12H15ClN2	222.714	Detomidine HCl
Selleck Chemicals	S1534	c1(Cl)cc2c([nH]c(C(NCCc3ccc(N4CCCCC4)cc3)=O)c2CC)cc1	C24H28ClN3O	409.952	Org 27569
Selleck Chemicals	S2124	OC[C@@H](O)[C@H](O)[C@@H](O)C=O	C5H10O5	150.13	Xylose
Selleck Chemicals	S1630	N(C(=O)C(=C1)C(NN1)=N2)=C2	C5H4N4O	136.111	Allopurinol
Selleck Chemicals	S2199	C(=O)(O)C=CC(=O)O.c1(OC)ccc(C[C@H](C(C)C)C[C@H](N)[C@@H](O)C[C@@H](C(C)C)C(=O)NCC(C)(C)C(=O)N)cc1OCCCOC.c2(OC)ccc(C[C@H](C(C)C)C[C@H](N)[C@@H](O)C[C@@H](C(C)C)C(=O)NCC(C)(C)C(=O)N)cc2OCCCOC	C64H110N6O16	1219.589	Aliskiren Hemifumarate
Selleck Chemicals	S1683	[Br-].C1(CC(OC(=O)C(CO)c2ccccc2)C3)[N+](C(C)C)(C)C3CC1	C20H30BrNO3	412.361	Ipratropium Bromide
Selleck Chemicals	S2286	N1(C)[C@H](CC(C)C)C(=O)N(C)[C@H](C(C)C)C(=O)N(C)[C@H]([C@@H]([C@H](C)CC=CC)O)C(=O)N[C@@H](CC)C(=O)N(C)CC(=O)N(C)[C@@H](CC(C)C)C(=O)N[C@@H](C(C)C)C(=O)N(C)[C@@H](CC(C)C)C(=O)N[C@@H](C)C(=O)N[C@H](C)C(=O)N(C)[C@@H](CC(C)C)C1=O	C62H111N11O12	1202.611	Cyclosporin A
Selleck Chemicals	S1761	c1(C(=O)c2cccs2)ccc(C(C)C(=O)O)cc1	C14H12O3S	260.308	Suprofen
Selleck Chemicals	S2321	c1c(CC=C)cc(c2c(O)ccc(CC=C)c2)c(O)c1	C18H18O2	266.334	Magnolol
Selleck Chemicals	S1823	[Ca+2].O(c1ccccc1)c2cc(C(C(=O)[O-])C)ccc2.O(c3ccccc3)c4cc(C(C(=O)[O-])C)ccc4	C30H26CaO6	522.602	Fenoprofen Calcium
Selleck Chemicals	S2358	c1(OC)c(O)ccc(C2C(CO)Oc3c(ccc(C4Oc5c(c(O)cc(O)c5)C(=O)C4O)c3)O2)c1	C25H22O10	482.436	Silymarin
Selleck Chemicals	S1885	C(C(=O)OCC)(C(c1c(Cl)c(Cl)ccc1)C(C(=O)OC)=C(C)N2)=C2C	C18H19Cl2NO4	384.254	Felodipine
Selleck Chemicals	S2389	c1(O[C@H]2[C@@H](O[C@]3([H])O[C@@H](C)[C@H](O)[C@@H](O)[C@H]3O)[C@@H](O)[C@H](O)[C@@H](CO)O2)cc(O)c(C(=O)CCc4ccc(O)cc4)c(O)c1	C27H34O14	582.55	Naringin Dihydrochalcone

Selleck Chemicals	S2449	C1[C@@H](O)[C@@]2(C)[C@@]([H])([C@H](O)[C@H](OC(=O)C)[C@]3(C)[C@@]2(O)C(=O)C[C@](C)(C=C)O3)C(C)(C)C1	C22H34O7	410.501	Forskolin
Selleck Chemicals	S2885	c(sc(C(N)=O)c1)(cncc2Sc3ccc(C)cc3)c12	C15H12N2OS2	300.399	A-205804
Selleck Chemicals	S2526	c1cc2c(C(=O)c3c(c(O)c(O)cc3)C2=O)cc1	C14H8O4	240.211	Alizarin
Selleck Chemicals	S2899	c1cc(C(N)=O)cc(c2cc(Nc3ccc(OC(F)(F)F)cc3)ncn2)c1	C18H13F3N4O2	374.317	GNF-2
Selleck Chemicals	S2581	c1cc(CC[C@@H](C(=O)OCC)N[C@@H](C)C(=O)N2Cc3c(cccc3)C[C@H]2C(=O)O)ccc1.Cl	C25H31ClN2O5	474.977	Quinapril HCl
Selleck Chemicals	S2907	N1(C=C(C)C=CC1=O)c2ccccc2	C12H11NO	185.222	Pirfenidone
Selleck Chemicals	S2679	c1c(O)c2c(OC(c3ccccc3Cl)=CC2=O)c([C@@]4([H])CCN(C)C[C@H]4O)c1O.Cl	C21H21Cl2NO5	438.301	Flavopiridol HCl
Selleck Chemicals	S2918	c1(c2ncn1CC(=O)Nc3ccc(C(C)C)cc3)C(=O)N(C)C(=O)N2C	C18H21N5O3	355.391	HC-030031
Selleck Chemicals	S2782	c1c(C(O)=O)cc(C=Cc2ccc(OCc3c(C(C)C)onc3c4c(Cl)cccc4Cl)cc2Cl)cc1	C28H22Cl3NO4	542.838	GW4064
Selleck Chemicals	S2929	C1CC2=C(SC(=N)N2CC(=O)c3ccc(C)cc3)CC1.Br	C16H19BrN2OS	367.304	Pifithrin-? (PFT?)
Selleck Chemicals	S2818	c1(NC(=O)C)ccc(C(Nc2ccccc2N)=O)cc1	C15H15N3O2	269.298	CI994 (Tacedinaline)
Selleck Chemicals	S3037	N1(CCCC(O)=O)CCC(O[C@@H](c2ccc(Cl)cc2)c3ncccc3)CC1.c4cccc(S(=O)(=O)O)c4	C27H31ClN2O6S	547.063	Bepotastine Besilate
Selleck Chemicals	S2832	C1C[C@@]2(C)[C@@]([H])(CC[C@]3([H])[C@@]2([H])CC[C@@]4(C)[C@@]3([H])CCC4=O)C[C@H]1O	C19H30O2	290.44	Epiandrosterone
Selleck Chemicals	S3051	c1(S(Nc2c(Oc3ccccc3OC)c(OCCO)nc(c4ncccn4)n2)(=O)=O)ccc(C(C)(C)C)cc1.O	C27H31N5O7S	569.629	Bosentan Hydrate
Selleck Chemicals	S2854	C(=O)(N1CCOCC1)Cc2c(c3n(C(=O)c4ccc(Cl)cc4)c2C)cc(OC)cc3	C23H23ClN2O4	426.893	BML-190
Selleck Chemicals	S3066	c(c1c(O)cc2)(c2C3)[C@@](CCN4CC=C)([C@@]5([H])O1)[C@@](O)(CCC5=O)[C@@H]34.Cl	C19H22ClNO4	363.835	Naloxone HCl
Selleck Chemicals	S2862	c1(C(Nc2cccc(Cl)c2)=O)ncccc1	C12H9ClN2O	232.666	VU 0364770
Selleck Chemicals	S3080	c1(Oc2c(C)cc(C#N)cc2C)c(Br)c(N)nc(Nc3ccc(C#N)cc3)n1	C20H15BrN6O	435.277	Etravirine (TMC125)
Selleck Chemicals	S2871	C(Nc1ccncc1)(=O)c2cc([N+]([O-])=O)ccc2Cl	C12H8ClN3O3	277.663	T0070907
Selleck Chemicals	S3114	OC1=C(O)C(=O)O[C@@H]1[C@H](CO)O	C6H8O6	176.124	Vitamin C
Selleck Chemicals	S3116	c1(S(Nc2nccs2)(=O)=O)ccc(N)cc1	C9H9N3O2S2	255.317	Sulfathiazole
Selleck Chemicals	S4045	c1cc(C(c2ccccn2)CCN(C)C)ccc1.C(=O)(O)C=CC(=O)O	C20H24N2O4	356.416	Pheniramine Maleate
Selleck Chemicals	S3139	C1N(CN(CN2C3)CN13)C2	C6H12N4	140.186	Methenamine
Selleck Chemicals	S4054	C1C[C@@]2(C)C(C[C@@H](SC(=O)C)[C@]3([H])[C@@]2([H])CC[C@@]4(C)[C@@]3([H])CC[C@]45CCC(=O)O5)=CC1=O	C24H32O4S	416.573	Spironolactone
Selleck Chemicals	S3154	c1(CN(Cc2ccc(C(C)(C)C)cc2)C)c(c3ccc1)cccc3.Cl	C23H28ClN	353.928	Butenafine HCl
Selleck Chemicals	S4068	c1c([N+]([O-])=O)n(CCS(=O)(=O)CC)c(C)n1	C8H13N3O4S	247.272	Tinidazole
Selleck Chemicals	S3172	c1cc2c(CN3C(=NC(=O)C3)N2)c(Cl)c1Cl.Cl	C10H8Cl3N3O	292.549	Anagrelide HCl
Selleck Chemicals	S4078	C(O)(=O)c1ccccc1Nc2cccc(C)c2C	C15H15NO2	241.285	Mefenamic Acid
Selleck Chemicals	S3186	c(C(=C1CCN(C)CC1)c2c(cccc2)CC3)(nccc4)c34.C(O)(=O)C=CC(O)=O.C(O)(=O)C=CC(O)=O	C28H30N2O8	522.546	Azatadine dimaleate
Selleck Chemicals	S4088	C1=C[C@@]2(C)C([C@@H](F)C[C@]3([H])[C@@]2(F)[C@H](O)C[C@@]4(C)[C@@]3([H])C[C@@H](C)[C@@]4(C(=O)CO)O)=CC1=O	C22H28F2O5	410.452	Flumethasone
Selleck Chemicals	S3202	c1cc2c([nH]c3c2CCN(C[C@@]45[H])[C@]([H])(C(CC)=C4)[C@]3(C(OC)=O)C5)cc1	C21H24N2O2	336.427	Catharanthine
Selleck Chemicals	S4098	C1C[C@@]2(C)C(CC[C@]3([H])[C@@]2(F)[C@H](O)C[C@@]4(C)[C@@]3([H])C[C@@H]5[C@]4(C(CCl)=O)OC(C)(C)O5)=CC1=O	C24H32ClFO5	454.959	Halcinonide
Selleck Chemicals	S4003	[C@]1([H])(CC[C@H]2[C@H](C)CCC(=O)O)[C@]2(C)CC[C@@]3([H])[C@@]1([H])CC[C@@]4([H])[C@]3(C)CC[C@@H](O)C4	C24H40O3	376.573	Lithocholic acid
Selleck Chemicals	S4107	N1(c2ccc(Cl)cc2)c3c(cccc3)N=C4C1=CC(=NC(C)C)C(Nc5ccc(Cl)cc5)=C4	C27H22Cl2N4	473.396	Clofazimine

Selleck Chemicals	S4014	C1(OC(=O)[C@H](CO)c2ccccc2)CC3N(C)C(CC3)C1	C17H23NO3	289.369	Hyoscyamine
Selleck Chemicals	S4118	c1(nc[nH]c1)CCN.Cl.Cl	C5H11Cl2N3	184.067	Histamine 2HCl
Selleck Chemicals	S4023	c1(ccc(N)cc1)C(=O)OCCN(CC)CC.Cl	C13H21ClN2O2	272.771	Procaine HCl
Selleck Chemicals	S4132	c1cc(OC2OCCCC2)ccc1O	C11H14O3	194.227	Deoxyarbutin
Selleck Chemicals	S4037	C1(=O)N(CC)CC(CCN2CCOCC2)C1(c3ccccc3)c4ccccc4.Cl.O	C24H33ClN2O3	432.983	Doxapram HCl
Selleck Chemicals	S4146	C([C@@H](N)C1=NCC(C(=O)N[C@@H](CC(C)C)C(N[C@@H](CCC(=O)O)C(=O)N[C@@H]([C@@H](CC)C)C(N[C@@H]2CCCCNC(=O)[C@H](CC(N)=O)NC(=O)[C@H](CC(O)=O)NC(=O)[C@H](Cc3c[nH]cn3)NC(=O)[C@H](Cc4ccccc4)NC(=O)[C@H]([C@H](C)CC)NC(=O)[C@@H](CCCN)NC2=O)=O)=O)S1)(C)C	C65H101N17O16S	1408.667	Bacitracin
Selleck Chemicals	S4161	c1cc(C(=O)O)ccc1	C7H6O2	122.121	Benzoic Acid
Selleck Chemicals	S4257	c([nH]nn1)(cc(OC)c(C(=O)NCC2CCCN2CC=C)c3)c13.Cl	C16H22ClN5O2	351.831	Alizapride HCl
Selleck Chemicals	S4170	O1c(c2C=CC1=O)cccc2	C9H6O2	146.143	Coumarin
Selleck Chemicals	S4266	c1cnc2c(c(Br)c(NC(NCC3)=N3)cc2)n1.[C@H](C(=O)O)(O)[C@@H](O)C(=O)O	C15H16BrN5O6	442.221	Brimonidine Tartrate
Selleck Chemicals	S4179	N1(S(C)(=O)=O)CCN(C(N[C@@H](c2ccccc2)C(N[C@H]3C(=O)N4[C@@]3([H])SC(C)(C)[C@@H]4C(=O)[O-])=O)=O)C1=O.[Na+]	C21H24N5NaO8S2	561.564	Mezlocillin Sodium
Selleck Chemicals	S4278	c1cc2c(CCC(=O)N2)c(OCC(O)CNC(C)(C)C)c1.Cl	C16H25ClN2O3	328.834	Carteolol HCl
Selleck Chemicals	S4189	C1C(OC(C(O)c2ccccc2)=O)CC(C)(C)CC1C	C17H24O3	276.371	Cyclandelate
Selleck Chemicals	S4290	[C@H]1(O)C[C@]([H])(O[C@@H]2[C@H](C)O[C@@]([H])(O[C@H]3CC[C@@]4(C)[C@]([H])(CC[C@]5([H])[C@@]4([H])C[C@@H](O)[C@@]6(C)[C@]5(O)CC[C@H]6C7=CC(=O)OC7)C3)C[C@@H]2O)O[C@H](C)[C@H]1O[C@@]8([H])C[C@H](O)[C@H](O)[C@@H](C)O8	C41H64O14	780.938	Digoxin
Selleck Chemicals	S4198	c1nc(N)sc1	C3H4N2S	100.142	Aminothiazole
Selleck Chemicals	S4420	Cl.c1c(C(F)(F)F)c2c(c([C@@H]([C@@]3([H])CCCCN3)O)cc(C(F)(F)F)n2)cc1	C17H17ClF6N2O	414.773	Mefloquine HCl
Selleck Chemicals	S4207	c1cc(OC(C)(C)C(O)=O)ccc1Cl	C10H11ClO3	214.646	Clofibric Acid
Selleck Chemicals	S4921	c(OCO1)(cc(C=C[N+]([O-])=O)cc2)c12	C9H7NO4	193.156	MNS (3,4-Methylenedioxy-?-nitrostyrene, MDBN)
Selleck Chemicals	S4217	c1(c(I)cc(C[C@H](N)C([O-])=O)cc1I)Oc2ccc(O)c(I)c2.[Na+]	C15H11I3NNaO4	672.955	Liothyronine Sodium
Selleck Chemicals	S7016	n1c(N)ncc(c2c3c(n(C(C)C)c(C)n3)nc(N4CCOCC4)n2)c1	C17H22N8O	354.41	VS-5584 (SB2343)
Selleck Chemicals	S4227	[C@@H]1(CC)[C@H](O[C@@]2([H])[C@H](O)[C@@H](O)[C@H](OC(=O)C(C)C)C(C)(C)O2)C(C)=CC(C)=CC[C@]([H])([C@H](O)C)OC(=O)C(CO[C@@H]3O[C@H](C)[C@@H](OC(c4c(O)c(Cl)c(O)c(Cl)c4CC)=O)[C@H](O)[C@@H]3OC)=CC=CC[C@H](O)C(C)=C1	C52H74Cl2O18	1058.039	Fidaxomicin
Selleck Chemicals	S7036	c1c(NC(=O)[C@H]2NCCC2)ccc(c3nc(Nc4ccc(N5CCOCC5)cc4)ncc3)c1	C25H28N6O2	444.529	XL019
Selleck Chemicals	S4237	c1(OC)cc2c(nccc2)c(NC(C)CCCN)c1.OP(O)(=O)O.OP(O)(=O)O	C15H27N3O9P2	455.337	Primaquine Diphosphate
Selleck Chemicals	S7051	C(Nc1ccc(C(N2CCOCC2)=O)cc1)(C(=O)N(C)C=C3c4c(C)c(NC(=O)c5ccc(C(C)(C)C)cc5)ccc4)=N3	C34H37N5O4	579.689	CGI1746
Selleck Chemicals	S4247	C(O)(=O)CCC(O)=O.O1c2c(c(N)c(Cl)cc2C(NC3CCN(CCCOC)CC3)=O)CC1	C22H32ClN3O7	485.958	Prucalopride Succinat
Selleck Chemicals	S7076	c1cc(S(=O)(N(CC(F)(F)F)c2ccc(C(C(F)(F)F)(O)C(F)(F)F)cc2)=O)ccc1	C17H12F9NO3S	481.333	T0901317
Selleck Chemicals	S3121	c1nc(C)n(CC(O)CCl)c1[N+]([O-])=O	C7H10ClN3O3	219.626	Ornidazole
Selleck Chemicals	S4046	c1cc2c(CC[C@]3([H])[C@@]2([H])CC[C@@]4(C)[C@@]3([H])CC[C@H]4OC(CCC5CCCC5)=O)cc1O	C26H36O3	396.562	Estradiol Cypionate
Selleck Chemicals	S3140	[C@H]1(CN)C[C@@]1(c2ccccc2)C(=O)N(CC)CC.Cl	C15H23ClN2O	282.809	Milnacipran HCl

Selleck Chemicals	S4056	[C@@]1([H])(C[C@H](SCC(=O)O[C@H]2[C@@]3(C)[C@@]4([H])[C@](CCC4=O)(CC[C@@H]3C)[C@@H](C)[C@H](O)[C@](C=C)(C)C2)C5)N(C)[C@]5([H])CC1	C30H47NO4S	517.763	Retapamulin
Selleck Chemicals	S3155	N1(C)CCCCC1C(Nc2c(C)cccc2C)=O.Cl	C15H23ClN2O	282.809	Mepivacaine HCl
Selleck Chemicals	S4070	NC(=N)N.Cl	CH6ClN3	95.531	Guanidine HCl
Selleck Chemicals	S3173	N1(C)C(C)=CC(=O)N1c2ccccc2	C11H12N2O	188.226	Antipyrine
Selleck Chemicals	S4079	n1([C@@H]2C[C@H](OCCO)[C@H](O)[C@H]2O)nnc3c1nc(SCCC)nc3N[C@@H]4C[C@@H]4c5ccc(F)c(F)c5	C23H28F2N6O4S	522.568	Ticagrelor
Selleck Chemicals	S3188	c1cc(C(O)CN)ccc1O.Cl	C8H12ClNO2	189.639	(+,-)-Octopamine HCl
Selleck Chemicals	S4089	C1=C[C@@]2(C)C([C@@H](F)C[C@]3([H])[C@@]2(F)[C@H](O)C[C@@]4(C)[C@@]3([H])C[C@H](C)[C@@]4(OC(CC)=O)C(=O)CCl)=CC1=O	C25H31ClF2O5	484.96	Halobetasol Propionate
Selleck Chemicals	S3204	c1(O)cccc(C2(CC)CCCCN(C)C2)c1.Cl	C15H24ClNO	269.81	Meptazinol HCl
Selleck Chemicals	S4099	c1(OCC(F)(F)F)c(C)c(C[S@@](=O)c2[nH]c3c(cccc3)n2)ncc1	C16H14F3N3O2S	369.361	Dexlansoprazole
Selleck Chemicals	S4004	[C@@H](CO)(CC)NCCN[C@H](CO)CC.Cl.Cl	C10H26Cl2N2O2	277.232	Ethambutol HCl
Selleck Chemicals	S4109	C1Cc2c(cc(Cl)cc2)[C@@H](C)CN1.Cl	C11H15Cl2N	232.15	Lorcaserin HCl
Selleck Chemicals	S4015	C1(CCCCC1)NS(=O)(=O)O	C6H13NO3S	179.237	Cyclamic acid
Selleck Chemicals	S4120	c1n(CC(SCc2ccc(Cl)cc2)c3ccc(Cl)cc3Cl)ccn1.[N+]([O-])(=O)O	C18H16Cl3N3O3S	460.762	Sulconazole Nitrate
Selleck Chemicals	S4024	[Br-].[C@@]1([H])(C[C@@H](OC(=O)C(O)c2ccccc2)C3)[N+](C)(C)[C@]3([H])CC1	C17H24BrNO3	370.281	Homatropine Methylbromide
Selleck Chemicals	S4135	c1cc(Cl)c(C(O)CNC(C)C)cc1.Cl	C11H17Cl2NO	250.165	Clorprenaline HCl
Selleck Chemicals	S4038	c1(cccc2)c2nc(OCCCC)cc1C(=O)NCCN(CC)CC.Cl	C20H30ClN3O2	379.924	Dibucaine HCl
Selleck Chemicals	S4148	c1ccc([C@@H](N)C(=O)N[C@H]2[C@@]3([H])N([C@@H](C(=O)O)C(C)(C)S3)C2=O)cc1.O.O.O	C16H25N3O7S	403.451	Ampicillin Trihydrate
Selleck Chemicals	S4162	c1cc(OCCOCC[N+](C)(C)Cc2ccccc2)ccc1C(C)(C)CC(C)(C)C.[Cl-]	C27H42ClNO2	448.081	Benzethonium Chloride
Selleck Chemicals	S4258	c1(ccc(Cl)cc1Cl)[C@@H]2CSC(=C(C#N)n3cncc3)S2	C14H9Cl2N3S2	354.277	Luliconazole
Selleck Chemicals	S4171	C(CO)[N+](C)(C)C.[Cl-]	C5H14ClNO	139.624	Choline Chloride
Selleck Chemicals	S4267	c(C(=O)c1c(cccc1OC(C)=O)C2=O)(c(OC(=O)C)cc(C(=O)O)c3)c23	C19H12O8	368.294	Diacerein
Selleck Chemicals	S4181	C(C(=O)OC)(C(c1cccc([N+]([O-])=O)c1)C(C(=O)OCCN(C)Cc2ccccc2)=C(C)N3)=C3C.Cl	C26H30ClN3O6	515.986	Nicardipine HCl
Selleck Chemicals	S4279	c1c(Cl)c2c(C(=O)C(=C3O)[C@]([H])(C[C@]4([H])[C@@]3(O)C(=O)C(C(=O)N)=C(O)[C@@H]4N(C)C)[C@H]2O)c(O)c1.Cl	C21H22Cl2N2O8	501.314	Demeclocycline HCl
Selleck Chemicals	S4190	n1c(c2c(C(O)=O)cc1c3ccccc3)cccc2	C16H11NO2	249.264	Cinchophen
Selleck Chemicals	S4291	c1(C(=O)N)cc(C(O)CNC(C)CCc2ccccc2)ccc1O.Cl	C19H25ClN2O3	364.866	Labetalol HCl
Selleck Chemicals	S4199	c1ccc(CN(c2ccccc2)CC3=NCCN3)cc1.Cl	C17H20ClN3	301.814	Antazoline HCl
Selleck Chemicals	S4627	c(C(C)(C)CCC1(C)C)(cc(C(C)=Cc2ccc(C(=O)O)cc2)cc3)c13	C24H28O2	348.478	TTNPB (Arotinoid Acid)
Selleck Chemicals	S4208	c1cc2c(OC(C(=O)O)=CC2=O)cc1	C10H6O4	190.152	Chromocarb
Selleck Chemicals	S4926	c1(F)cc2c(CC[C@@H](N3C(=S)NC=C3CN)C2)c(F)c1.Cl	C14H16ClF2N3S	331.812	(R)-Nepicastat HCl
Selleck Chemicals	S4219	N1(CCN(CCCC(=O)c2ccc(F)cc2)CC1)c3ncccc3	C19H22FN3O	327.396	Azaperone
Selleck Chemicals	S7018	c1(S(NC(C)(C)C)(=O)=O)cc(c2cn(c3c(F)c2)nc(N)n3)cnc1	C15H17FN6O2S	364.398	CZC24832
Selleck Chemicals	S4228	[C@](F)([C@]1(C)C(=CC(=O)C=C1)[C@@]([H])(C)C2)([C@](O)([H])C[C@@]3(C)[C@@]4([H])CC[C@@]3(C(=O)C)OC(=O)C)[C@@]24[H]	C24H31FO5	418.498	Fluorometholone Acetate
Selleck Chemicals	S7037	n1ccc(c2ccc(CC(=O)Nc3ccc(c4cnccc4)cc3)cc2)cc1C	C25H21N3O	379.454	Wnt-C59 (C59)

Selleck Chemicals	S4238	c1(OC)cc2c([C@@]3([H])N(C)CC2)cc1Oc(c(c45)[C@]([H])(N(C)CC4)Cc(ccc6Oc7cc(ccc7OC)C3)cc6)c8c(OCO8)c5	C37H38N2O6	606.707	Cepharanthine
Selleck Chemicals	S7059	c1(OC(F)(F)O2)c2cc(C3(CC3)C(Nc4cc5c(n(C[C@@H](O)CO)c(C(C)(C)CO)c5)cc4F)=O)cc1	C26H27F3N2O6	520.498	VX-661
Selleck Chemicals	S4248	[Na+].C([O-])(=O)Cc1cccc(C(=O)c2ccc(Br)cc2)c1N	C15H11BrNNaO3	356.147	Bromfenac Sodium
Selleck Chemicals	S7077	N1(C)[C@@H](C(C)C)C(=O)N[C@@H](CCCNC(N)=N)C(=O)NCC(=O)N[C@@H](CC(O)=O)C(=O)N[C@H](Cc2ccccc2)C1=O	C27H40N8O7	588.656	Cilengitide
Selleck Chemicals	S3129	c1(N)ncc(Cc2cc(OC)c(OC)c(OC)c2)c(N)n1	C14H18N4O3	290.318	Trimethoprim
Selleck Chemicals	S4047	c1(C(c2ccc(OC(C)=O)cc2)c3ncccc3)ccc(OC(C)=O)cc1	C22H19NO4	361.391	Bisacodyl
Selleck Chemicals	S3144	c1(CCN2C[C@H](C(c3ccccc3)(c4ccccc4)C(N)=O)CC2)cc5c(OCC5)cc1.Br	C28H31BrN2O2	507.462	Darifenacin HBr
Selleck Chemicals	S4057	c1(S(N)(=O)=O)cc2c(NC(CCl)N(C)S2(=O)=O)cc1Cl	C9H11Cl2N3O4S2	360.237	Methyclothiazide
Selleck Chemicals	S3160	C1C[C@@]2([H])C(CC[C@]3([H])[C@@]2([H])CC[C@@]4(C)[C@@]3([H])CC[C@]4(OC(C)=O)C#C)=C[C@H]1OC(C)=O	C24H32O4	384.508	Ethynodiol diacetate
Selleck Chemicals	S4071	c1c(OC)c2c(O[C@]3(C(OC)=CC(=O)C[C@H]3C)C2=O)c(Cl)c1OC	C17H17ClO6	352.766	Griseofulvin
Selleck Chemicals	S3176	c1(CCNC)ncccc1.Cl.Cl	C8H14Cl2N2	209.116	Betahistine 2HCl
Selleck Chemicals	S4080	c1(N)nc(N)c2c(nc(N)c(c3ccccc3)n2)n1	C12H11N7	253.263	Triamterene
Selleck Chemicals	S3190	OC[C@H]1O[C@@H](n2c(c3nc2)ncnc3NC)[C@H](O)[C@@H]1O	C11H15N5O4	281.268	N6-methyladenosine (m6A)
Selleck Chemicals	S4090	c1c(CCN2CCC3(OC(=O)NC3)CC2)cccc1.Cl	C15H21ClN2O2	296.792	Fenspiride HCl
Selleck Chemicals	S3208	C1(C(O)(c2ccccc2)c3ccccc3)CCN(CCCC(O)c4ccc(C(C)(C)C(=O)O)cc4)CC1.Cl	C32H40ClNO4	538.117	Fexofenadine HCl
Selleck Chemicals	S4100	c1cc(OCC(O)CNC(C)C)ccc1CCC(=O)OC.Cl	C16H26ClNO4	331.835	Esmolol HCl
Selleck Chemicals	S4005	Cl.c1cc2c(C(=O)C(=C3O)[C@]([H])([C@@H](O)[C@]4([H])[C@@]3(O)C(=O)C(C(=O)N)=C(O)[C@@H]4N(C)C)[C@@H]2C)c(O)c1	C22H25ClN2O8	480.895	Doxycycline HCl
Selleck Chemicals	S4110	c1cc2c(CC[C@]3([H])[C@@]2([H])CC[C@@]4(C)[C@@]3([H])CC[C@H]4O)cc1OC(=O)c5ccccc5	C25H28O3	376.488	Estradiol Benzoate
Selleck Chemicals	S4016	[C@@]1(CO)([C@@H](O)C[C@@H](O[C@@H]2O[C@H](C)[C@H](O)[C@@H](O)[C@@H]2O)C3)[C@]3(O)CC[C@]4([H])[C@@]1([C@H](O)C[C@@]5(C)[C@]4(O)CC[C@H]5C6=CC(=O)OC6)[H].O.O.O.O.O.O.O.O	C29H60O20	728.775	Ouabain
Selleck Chemicals	S4122	[C@@H]1(O[C@@H]2O[C@@H](C)[C@@H](O)[C@H](N(C)C)[C@H]2O)[C@@H](C)[C@H](O)CC(=O)O[C@H](CC)[C@H](CO[C@@H]3O[C@@H](C)[C@@H](O)[C@@H](OC)[C@H]3OC)C=C(C)C=CC(=O)[C@H](C)C[C@@H]1CCN4C[C@H](C)C[C@@H](C)C4	C46H80N2O13	869.133	Tilmicosin
Selleck Chemicals	S4025	[C@@H]1(OC(=O)C(O)c2ccccc2)C[C@]3([H])N(C)[C@]([H])(CC3)C1.Br	C16H22BrNO3	356.255	Homatropine Bromide
Selleck Chemicals	S4136	OC(=O)C(C)c1cc2c(c3c(ccc(Cl)c3)[nH]2)cc1	C15H12ClNO2	273.714	Carprofen
Selleck Chemicals	S4039	C1(=NC(=O)C)N(C)N=C(S(=O)(=O)N)S1	C5H8N4O3S2	236.272	Methazolamide
Selleck Chemicals	S4149	c1(cccc(CC([O-])=O)c1N)C(=O)c2ccccc2.O.[Na+]	C15H14NNaO4	295.266	Amfenac Sodium Monohydrate
Selleck Chemicals	S4163	c1c(O)c2c([C@H](C)[C@]3([H])C(=C(O)[C@@]4(O)[C@]([H])([C@H](N(C)C)C(O)=C(C(N)=O)C4=O)[C@H]3O)C2=O)cc1.c5c(O)c6c([C@H](C)[C@]7([H])C(=C(O)[C@@]8(O)[C@]([H])([C@H](N(C)C)C(O)=C(C(N)=O)C8=O)[C@H]7O)C6=O)cc5.Cl.O.CCO.Cl	C46H58Cl2N4O18	1025.875	Doxycycline Hyclate
Selleck Chemicals	S4259	c(cc(C#N)cc1)(c(CCCCN2CCN(c3ccc4c(cc(C(=O)N)o4)c3)CC2)c[nH]5)c15.Cl	C26H28ClN5O2	477.986	Vilazodone HCl
Selleck Chemicals	S4172	[n+]1(CCCCCCCCCCCCCCCC)ccccc1.[Cl-]	C21H38ClN	339.986	Cetylpyridinium Chloride
Selleck Chemicals	S4268	C(O)(=O)c1ccccc1Nc2cccc(C(F)(F)F)c2	C14H10F3NO2	281.23	Flufenamic acid
Selleck Chemicals	S4182	c1cc(C=NNC(=O)c2ccc(O)cc2)oc1[N+](=O)[O-]	C12H9N3O5	275.217	Nifuroxazide
Selleck Chemicals	S4280	c1cc(OCC(=O)OCCN(C)C)ccc1Cl.Cl	C12H17Cl2NO3	294.174	Meclofenoxate (Centrophenoxine) HCl
Selleck Chemicals	S4191	c1ccccc1C(=O)NCCC(=O)O	C10H11NO3	193.199	Betamipron

Selleck Chemicals	S4292	c1cc(C(O)(c2ccccc2)CCCN3CCCCC3)ccc1.Cl	C21H28ClNO	345.906	Diphenidol HCl
Selleck Chemicals	S4200	c1cc(C(=O)C(C)CN2CCCCC2)ccc1C.Cl	C16H24ClNO	281.821	Tolperisone HCl
Selleck Chemicals	S4900	CC(c1ccc(C(NC(Nc2ccc(NC(CCCCN(C)C)=O)cc2)=S)=O)cc1)(C)C	C25H34N4O2S	454.628	Tenovin-6
Selleck Chemicals	S4209	c1(C)cc(O)ccc1Cl	C7H7ClO	142.583	Chlorocresol
Selleck Chemicals	S5001	n1c(N([C@@H]2[C@@H](C)CCN(C(CC#N)=O)C2)C)c3c([nH]cc3)nc1.C(C(=O)O)C(O)(CC(O)=O)C(=O)O	C22H28N6O8	504.493	Tofacitinib (CP-690550) Citrate
Selleck Chemicals	S4220	n1c(NS(=O)(=O)c2ccc(C(C)(C)C)cc2)c(Oc3c(OC)cccc3)c(OCCO)nc1c4nccn4	C27H29N5O6S	551.614	Bosentan
Selleck Chemicals	S7023	C(C)(C)[C@H](NC(=O)OCc1ccccc1)C(=O)N[C@@H](C)C(N[C@H](C(CF)=O)CC(=O)OC)=O	C22H30FN3O7	467.488	Z-VAD-FMK
Selleck Chemicals	S4229	c1(C(=O)OCCN(CC)CC)ccc(N)c(OCCCC)c1.Cl	C17H29ClN2O3	344.877	Oxybuprocaine HCl
Selleck Chemicals	S7039	c1cc2c(c(Nc3cc(Br)ccc3)ncn2)cc1NC(=O)C=C	C17H13BrN4O	369.215	PD168393
Selleck Chemicals	S4239	C1=Cc2c(cc3c(cco3)c2OC)OC1=O	C12H8O4	216.19	Bergapten
Selleck Chemicals	S7060	n1c(N)c2c(n(C(C)(C)C)nc2c3ccc(C)cc3)nc1	C16H19N5	281.356	PP1
Selleck Chemicals	S4249	c1(C(=O)CC)c(O)cc(O)cc1O	C9H10O4	182.173	Flopropione
Selleck Chemicals	S7079	c1(C(C)(C)C)ccc(NC(NCCCN(C[C@H]2O[C@@H](n3cc(Br)c4c3ncnc4N)[C@H](O)[C@@H]2O)C(C)C)=O)cc1	C28H40BrN7O4	618.566	SGC 0946
Selleck Chemicals	S3130	C1(=O)N[C@@]2([H])[C@]([H])(CS[C@@H]2CCCCC(O)=O)N1	C10H16N2O3S	244.311	Biotin (Vitamin B7)
Selleck Chemicals	S4048	C1=CN(C)C(=S)N1C(OCC)=O	C7H10N2O2S	186.232	Carbimazole
Selleck Chemicals	S3146	C(N(C)C)CN(Cc1ccccc1)c2ncccc2.Cl	C16H22ClN3	291.819	Tripelennamine HCl
Selleck Chemicals	S4059	[Fe](N=O)(C#N)(C#N)(C#N)(C#N)C#N.[Na+].[Na+].[R]	C5FeN6Na2OR	261.918	Sodium Nitroprusside
Selleck Chemicals	S3161	c1n(CC(OCc2csc3c2cccc3Cl)c4ccc(Cl)cc4Cl)ccn1.[R]	C20H15Cl3N2ORS	437.77	Sertaconazole nitrate
Selleck Chemicals	S4072	[N+](C)(C)(C)CCCCCCCCCC[N+](C)(C)C.[Br-].[Br-]	C16H38Br2N2	418.294	Decamethonium Bromide
Selleck Chemicals	S3178	S1(=O)(=O)c2c(cc(S(N)(=O)=O)s2)[C@@H](NCC)CN1CCCOC	C12H21N3O5S3	383.507	Brinzolamide
Selleck Chemicals	S4081	c1cc(S(=O)(=O)[N-]C(=O)C)ccc1N.[Na+]	C8H9N2NaO3S	236.223	Sulfacetamide Sodium
Selleck Chemicals	S3193	N1(C2SC(C)(C)C1C(O)=O)C(=O)C2NC([C@H](C(O)=O)c3ccsc3)=O.[Na].[Na]	C15H16N2Na2O6S2	430.407	Ticarcillin sodium
Selleck Chemicals	S4091	c1(O)ccc(C(O)C(C)N2CCC(Cc3ccccc3)CC2)cc1.c4(O)ccc(C(O)C(C)N5CCC(Cc6ccccc6)CC5)cc4.C(=O)(O)[C@H](O)[C@@H](O)C(=O)O	C46H60N2O10	800.976	Ifenprodil Tartrate
Selleck Chemicals	S3209	N1(C)C(C)=C(N(C)C)C(=O)N1c2ccccc2	C13H17N3O	231.294	Amidopyrine
Selleck Chemicals	S4101	[C@@H]1(O)[C@@](CO)(O)C[C@H](NC(CO)CO)[C@H](O)[C@H]1O	C10H21NO7	267.276	Voglibose
Selleck Chemicals	S4007	c1(C(=N)N)ccc(OCCCCCOc2ccc(C(=N)N)cc2)cc1.Cl.Cl	C19H26Cl2N4O2	413.341	Pentamidine
Selleck Chemicals	S4111	c1(c2c(C(=O)N[C@H]3[C@H]4N([C@@H](C(O[Na])=O)C(C)(C)S4)C3=O)c(C)on2)c(Cl)cccc1Cl.O	C19H18Cl2N3NaO6S	510.323	Dicloxacillin Sodium
Selleck Chemicals	S4017	N(CC=C)C(=S)N	C4H8N2S	116.185	Allylthiourea
Selleck Chemicals	S4123	c1(N2CCOCC2)nsnc1OC[C@@H](O)CNC(C)(C)C.C(C(=O)O)=CC(=O)O	C17H28N4O7S	432.492	Timolol Maleate
Selleck Chemicals	S4026	C(c1ccccc1)(c2ccc(Cl)cc2)N3CCN(CCOCCO)CC3.Cl.Cl	C21H29Cl3N2O2	447.826	Hydroxyzine 2HCl
Selleck Chemicals	S4138	N1(CCN(CC(O)CO)CC1)c2ccccc2	C13H20N2O2	236.31	Dropropizine
Selleck Chemicals	S4040	[C@@]([H])([C@@]1([H])[C@]([H])([C@]2([H])C(=CC(=O)CC2)CC1)CC3)(CC[C@@]4(O)C#C)[C@]34C	C20H26O2	298.419	Norethindrone
Selleck Chemicals	S4151	c1(ccc(Cl)c(C(F)(F)F)c1)C2(O)CCN(CCCC(c3ccc(F)cc3)c4ccc(F)cc4)CC2	C28H27ClF5NO	523.965	Penfluridol
Selleck Chemicals	S4164	N1(C)C(=O)c2c(ncn2CC3OCCO3)N(C)C1=O	C11H14N4O4	266.253	Doxofylline

Selleck Chemicals	S4260	c(C(C)(C)CCC1(C)C)(ccc(NC(=O)c2ccc(C(=O)O)cc2)c3)c13	C22H25NO3	351.439	Tamibarotene
Selleck Chemicals	S4173	C(CCCCCCCC)CCCCCCCO	C16H34O	242.441	1-Hexadecanol
Selleck Chemicals	S4269	c1([C@]2(C(=O)OC)c3c(c4c(cccc4)[nH]3)CN(CC5C2)CC(CC)=C5)cc6c(N(C)[C@]7([H])[C@]6(CC8)[C@]([H])(N89)[C@@](CC)(C=CC9)[C@@H](OC(=O)C)[C@]7(O)C(=O)OC)cc1OC.C(=O)(O)[C@H](O)[C@@H](O)C(=O)O.C(=O)(O)[C@H](O)[C@@H](O)C(=O)O	C53H66N4O20	1079.106	Vinorelbine Tartrate
Selleck Chemicals	S4184	N(C(=O)c1c(n(CCC(CO)CO)cn1)N2)=C2N	C10H15N5O3	253.258	Penciclovir
Selleck Chemicals	S4282	C1C[C@]2([H])[C@@]([H])(CN(C[C@@H](O)[C@H](CSc3ccccc3)NC(=O)c4cccc(O)c4C)[C@H](C(=O)NC(C)(C)C)C2)CC1.OS(=O)(=O)C	C33H49N3O7S2	663.888	Nelfinavir Mesylate
Selleck Chemicals	S4192	n1c(c2ccc1C)c(O)c(Cl)cc2Cl	C10H7Cl2NO	228.075	Chlorquinaldol
Selleck Chemicals	S4293	c1cc2c(N(CC(N(C)C)C)c3c(cccc3)S2)cc1.Cl	C17H21ClN2S	320.88	Promethazine HCl
Selleck Chemicals	S4201	c1(S(=O)(=O)C)ccc([C@@H](O)[C@@H](CF)NC(=O)C(Cl)Cl)cc1	C12H14Cl2FNO4S	358.213	Florfenicol
Selleck Chemicals	S4901	C(Nc1ccc(Nc2nccc(c3cccnc3)n2)c(C)c1)(=O)c4cccc(NC(=O)C=CCN(C)C)c4	C29H29N7O2	507.586	JNK-IN-8
Selleck Chemicals	S4210	c1(N)ccc(C(OCC)=O)cc1	C9H11NO2	165.189	Benzocaine
Selleck Chemicals	S7000	c1(Nc2ccc(N3CCC(N(C)C)CC3)cc2OC)ncc(Cl)c(Nc4ccccc4P(C)(C)=O)n1	C26H34ClN6O2P	529.014	AP26113
Selleck Chemicals	S4221	c1(C(=O)c2cc(Br)c(O)c(Br)c2)c(CC)oc3c1cccc3	C17H12Br2O3	424.083	Benzbromarone
Selleck Chemicals	S7024	c1([N+]([O-])=O)cc2c(C=CS2(=O)=O)cc1	C8H5NO4S	211.195	Stattic
Selleck Chemicals	S4230	c1(c2ccccc2)oc(CCC(O)=O)nc1c3ccccc3	C18H15NO3	293.317	Oxaprozin
Selleck Chemicals	S7040	c1cc(OCCN2CCN(C(=O)C)CC2)ccc1C3CCN(C4=Nn5c(nnc5C(F)(F)F)CC4)CC3	C25H32F3N7O2	519.562	AZD3514
Selleck Chemicals	S4240	c1cccc(C(OCCN(C)C)(C)c2ncccc2)c1.C(O)(=O)CCC(O)=O	C21H28N2O5	388.457	Doxylamine Succinate
Selleck Chemicals	S7062	N([C@H]1C[C@@H](CCc2[nH]c3c(cc(C(C)(C)C)cc3)n2)C1)(C[C@H]4O[C@@H](n5cnc6c5ncnc6N)[C@H](O)[C@@H]4O)C(C)C	C30H42N8O3	562.706	EPZ5676
Selleck Chemicals	S4250	c1(S(Nc2nnc(OC)cc2)(=O)=O)ccc(N)cc1	C11H12N4O3S	280.303	Sulfamethoxypyridazine
Selleck Chemicals	S7083	c1ccc(Nc2nc(Nc3cc(C)c(C4CCNCC4)cc3OC(C)C)ncc2Cl)c(S(=O)(=O)C(C)C)c1	C28H36ClN5O3S	558.135	LDK378
Selleck Chemicals	S3132	c1(S(Nc2nccc(C)n2)(=O)=O)ccc(N)cc1	C11H12N4O2S	264.304	Sulfamerazine
Selleck Chemicals	S4049	c1cc(c2c(c3ccc(S(=O)(=O)N)cc3)c(C)on2)ccc1	C16H14N2O3S	314.359	Valdecoxib
Selleck Chemicals	S3147	C(N(CC)CC)(=O)C(C#N)=Cc1cc([N+]([O-])=O)c(O)c(O)c1	C14H15N3O5	305.286	Entacapone
Selleck Chemicals	S4062	c1([N+]([O-])=O)cnc(COC(=O)N)n1C	C6H8N4O4	200.152	Ronidazole
Selleck Chemicals	S3162	[C@H]1(CO[C@@H]2O[C@H](C)[C@@H](O)[C@@H](OC)[C@H]2OC)C=C(C)C=CC(=O)[C@H](C)C[C@H](CC=O)[C@H](O[C@@]3([H])O[C@H](C)[C@@H](O[C@@]4([H])C[C@](O)(C)[C@@H](O)[C@H](C)O4)[C@H](N(C)C)[C@H]3O)[C@@H](C)[C@H](O)CC(=O)O[C@@H]1CC.C(O)(=O)[C@H](O)[C@@H](O)C(O)=O	C50H83NO23	1066.187	Tylosin tartrate
Selleck Chemicals	S4073	[O-]C(=O)c1ccc(N)cc1O.[Na+]	C7H6NNaO3	175.117	Sodium 4-Aminosalicylate
Selleck Chemicals	S3179	N1([C@]2([H])SC(C)(C)[C@@H]1C([O-])=O)C(=O)[C@H]2NC(C(C([O-])=O)c3ccccc3)=O.[Na+].[Na+]	C17H16N2Na2O6S	422.363	Carbenicillin disodium
Selleck Chemicals	S4082	C1C=CC=C[C@H](O[C@H]2CC[C@H](N(C)C)[C@H](C)O2)[C@H](C)C[C@H](CC=O)[C@H](O[C@H]3O[C@@H](C)[C@H](O[C@H]4O[C@H](C)[C@@H](O)[C@@](C)(O)C4)[C@@H](N(C)C)[C@@H]3O)[C@H](OC)[C@H](O)CC(=O)O[C@@H]1C	C43H74N2O14	843.053	Spiramycin
Selleck Chemicals	S3195	N1(C2SC(C)(C)C1C(O)=O)C(=O)C2NC([C@H](NC(N3C(=O)NCC3)=O)c4ccccc4)=O.[Na]	C20H23N5NaO6S	484.481	Azlocillin sodium salt
Selleck Chemicals	S4092	Cl.c1cc(OCCCN2CCOCC2)ccc1OCCCC	C17H28ClNO3	329.862	Pramoxine HCl
Selleck Chemicals	S3600	c1(c(OC)c2c(OCO2)c3)c3C[C@H](C)[C@@H](C)Cc4c1c(OC)c(OC)c(OC)c4	C23H28O6	400.465	Schisandrin B (Sch B)

Selleck Chemicals	S4102	c1cc(Cn2c(CCCC)ncc2C=C(Cc3cccs3)C(=O)O)ccc1C(O)=O.CS(O)(=O)=O	C24H28N2O7S2	520.618	Eprosartan Mesylate
Selleck Chemicals	S4009	s1cc(CC(Nc2ccc(CCNC[C@H](O)c3ccccc3)cc2)=O)nc1N	C21H24N4O2S	396.506	Mirabegron
Selleck Chemicals	S4112	C1CCCCC1(O)C(c2ccc(O)cc2)CN(C)C.C(=O)(O)CCC(=O)O	C20H31NO6	381.463	Desvenlafaxine Succinate
Selleck Chemicals	S4019	C(c1cnc(N2CCC[C@H]2CO)nc1NCc3ccc(OC)c(Cl)c3)(=O)NCc4ncccn4	C23H26ClN7O3	483.951	Avanafil
Selleck Chemicals	S4124	c1(ccccc1)CC2=NCCN2.Cl	C10H13ClN2	196.677	Tolazoline HCl
Selleck Chemicals	S4027	c(c(C(=O)OCCN1CCCCC1)ccc2)(OC(c3ccccc3)=C(C)C4=O)c24.Cl	C24H26ClNO4	427.921	Flavoxate HCl
Selleck Chemicals	S4139	C1N(C)CCN(C(c2ccccc2)c3ccccc3)C1.Cl.Cl	C18H24Cl2N2	339.303	Cyclizine 2HCl
Selleck Chemicals	S4041	c1(C([O-])=O)c(O)ccc(N=Nc2cc(C(=O)[O-])c(O)cc2)c1.[Na+].[Na+]	C14H8N2Na2O6	346.203	Olsalazine Sodium
Selleck Chemicals	S4152	c1(S(=O)(=O)O)cc(O)ccc1O.N(CC)CC	C10H17NO5S	263.311	Ethamsylate
Selleck Chemicals	S4165	c1cc2c(c(OCCCN(C)C)nn2Cc3ccccc3)cc1.Cl	C19H24ClN3O	345.866	Benzydamine HCl
Selleck Chemicals	S4261	c1(c2c(OC)ccc1)C=[N]3[Mn+3]([Cl-])([N](=C4)CC3)([O-]c5c4cccc5OC)[O-]2	C18H18ClMnN2O4	416.738	EUK 134
Selleck Chemicals	S4175	c1(ccc(N)cc1)S(NC(N)=N)(=O)=O	C7H10N4O2S	214.245	Sulfaguanidine
Selleck Chemicals	S4270	N1(CC(=O)N)C(=O)CC(O)C1	C6H10N2O3	158.155	Oxiracetam
Selleck Chemicals	s4185	c1(O)c(I)cc(Oc2c(I)cc(CC(=O)O)cc2I)cc1	C14H9I3O4	621.932	Tiratricol
Selleck Chemicals	S4284	Nc1cc(Cl)c(C(=O)OCCN(CC)CC)cc1.Cl	C13H20Cl2N2O2	307.216	Chloroprocaine HCl
Selleck Chemicals	S4194	C1(N)=Nc2c(nn[nH]2)C(=O)N1	C4H4N6O	152.114	Azaguanine-8
Selleck Chemicals	S4294	c1cc(C(=O)NCCN(CC)CC)ccc1N.Cl	C13H22ClN3O	271.786	Procainamide HCl
Selleck Chemicals	S4203	C1OCCN(CC2CN(N=Cc3ccc([N+]([O-])=O)o3)C(=O)O2)C1.Cl	C13H17ClN4O6	360.75	Furaltadone HCl
Selleck Chemicals	S4902	n1c(c2c(NCCc3ccc(Oc4ccccc4)cc3)nc1)ccc(N)c2	C22H20N4O	356.42	QNZ (EVP4593)
Selleck Chemicals	S4211	n1c(C=Cc2cccc([C@@H](CCc3ccccc3C(C)(C)O)SCC4(CC([O-])=O)CC4)c2)ccc(c15)ccc(Cl)c5.[Na+]	C35H35ClNNaO3S	608.165	Montelukast Sodium
Selleck Chemicals	S7007	c(ncn1C)(c(F)c(Nc2ccc(Br)cc2F)c(C(=O)NOCCO)c3)c13	C17H15BrF2N4O3	441.227	MEK162 (ARRY-162, ARRY-438162)
Selleck Chemicals	S4222	[C@H]([C@@H](NC(=O)[C@H](NC(=O)N1CCN(CC)C(=O)C1=O)c2ccccc2)C3=O)(SC(C)(C)[C@H]4C([O-])=O)N34.[Na+]	C23H26N5NaO7S	539.537	Piperacillin Sodium
Selleck Chemicals	S7025	C1(CCCCCCCCCCC)=C(O)C(=O)C=C(O)C1=O	C17H26O4	294.386	Embelin
Selleck Chemicals	S4231	[C@H]1(Cc2cncn2C)COC(=O)[C@H]1CC.Cl	C11H17ClN2O2	244.718	Pilocarpine HCl
Selleck Chemicals	S7041	c1(C(=O)N2CCN(C)CCC2)cc(c3oc(C=C4c5c(ccc(Cl)c5)NC4=O)cc3)ccc1.Cl	C26H25Cl2N3O3	498.401	CX-6258 HCl
Selleck Chemicals	S4242	CCCCCCCCCCCCCCCC[N+](C)(C)C.[Br-]	C19H42BrN	364.447	Cetrimonium Bromide (CTAB)
Selleck Chemicals	S7065	c1(Cl)c(F)c(C(=O)N2CCN(Cc3nc(Nc4sccn4)ccc3)CC2)ccc1	C20H19ClFN5OS	431.914	MK-8745
Selleck Chemicals	S4251	c(cccc1)(Nc2c(cccc2)S3)c13	C12H9NS	199.272	Phenothiazine
Selleck Chemicals	S7085	C(CCS1)(N=C(SCC(=O)Nc2sc3c(ccc(C)c3)n2)N(c4ccccc4)C5=O)=C15	C22H18N4O2S3	466.599	IWP-2
Selleck Chemicals	S3133	c1cc(S(=O)(=O)Nc2nc(C)cc(C)n2)ccc1N	C12H14N4O2S	278.33	Sulfamethazine
Selleck Chemicals	S4050	C([C@H](N)C(=O)OCC(OCN1C2C(C(=O)NC(N)=N2)N=C1)CO)(C)C.Cl	C14H25ClN6O5	392.838	Valganciclovir HCl
Selleck Chemicals	S3149	c1cc2c(CC[C@]3([H])[C@@]2([H])CC[C@@]4(C)[C@@]3([H])CC[C@H]4OC(=O)CCCC)cc1O	C23H32O3	356.498	Estradiol valerate
Selleck Chemicals	S4063	C1C(=CC=C2CCC[C@@]3(C)[C@@]2([H])CC[C@H]3[C@H](C)CCCC(C)C)C(=C)CC[C@@H]1O	C27H44O	384.638	Vitamin D3
Selleck Chemicals	S3163	C1[C@@H](N(C)[C@@H]2C1)C[C@@H](OC(c3ccccc3)c4ccccc4)C2.CS(O)(=O)=O	C22H29NO4S	403.535	Benztropine mesylate

Selleck Chemicals	S4074	[O-]N=O.[Na+]	NNaO2	68.995	Sodium Nitrite
Selleck Chemicals	S3181	c1(cc(F)c2)c(N3C=C(C(O)=O)C1=O)c2CCC3C	C14H12FNO3	261.248	Flumequine
Selleck Chemicals	S4084	C(c1ccc(F)cc1)(c2ccc(F)cc2)N3CCN(Cc4ccc(OC)c(OC)c4OC)CC3.Cl.Cl	C27H32Cl2F2N2O3	541.457	Lomerizine HCl
Selleck Chemicals	S3196	N1CCC(C(c2ccccc2)(c3ccccc3)O)CC1	C18H21NO	267.365	Azacyclonol
Selleck Chemicals	S4095	C1(=O)C=C[C@@]2(C)C([C@@H](F)C[C@]3([H])[C@@]2(F)[C@H](O)C[C@@]4(C)[C@@]3([H])CC[C@@]4(OC(CCC)=O)C(=O)COC(=O)C)=C1	C27H34F2O7	508.551	Difluprednate
Selleck Chemicals	S3603	[C@@]([C@H](C(C)=C)CC1)([C@]2([H])[C@](C)([C@@]3(C)[C@@]([H])([C@]4(C)[C@]([H])(C(C)(C)[C@@H](O)CC4)CC3)CC2)CC5)([C@]15C(=O)O)[H]	C30H48O3	456.7	Betulinic acid
Selleck Chemicals	S4104	c1cc(N=NNc2ccc(C(N)=N)cc2)ccc1C(=N)N.OC(=O)CNC(C)=O.OC(=O)CNC(C)=O	C22H29N9O6	515.522	Diminazene Aceturate
Selleck Chemicals	S4010	c1(OCC(O)CNC(C)C)ccc(NC(=O)CCC)cc1C(=O)C.Cl	C18H29ClN2O4	372.887	Acebutolol HCl
Selleck Chemicals	S4113	C1CCCCC1(O)C(c2ccc(O)cc2)CN(C)C	C16H25NO2	263.375	Desvenlafaxine
Selleck Chemicals	S4020	C(c1ncccc1)(c2ccc(OS([O-])(=O)=O)cc2)c3ccc(OS([O-])(=O)=O)cc3.[Na+].[Na+]	C18H13NNa2O8S2	481.407	Sodium Picosulfate
Selleck Chemicals	S4125	C(O[Na])(=O)CCCc1ccccc1	C10H11NaO2	186.183	Sodium Phenylbutyrate
Selleck Chemicals	S4031	C(O)(c1cccs1)(c2cccs2)C(=O)O[C@@H]3C([H])(CC[N+]4(CCCOc5ccccc5)C3)CC4.[Br-]	C26H30BrNO4S2	564.555	Aclidinium Bromide
Selleck Chemicals	S4141	c1(C(N)=O)cc([N+](=O)[O-])cc([N+]([O-])=O)c1C	C8H7N3O5	225.158	Dinitolmide
Selleck Chemicals	S4042	c(cccc1)(c(C(=O)N[C@H]2[C@]3([H])N([C@H](C(O[Na])=O)C(C)(C)S3)C2=O)c(OCC)cc4)c14.O	C21H23N2NaO6S	454.472	Nafcillin Sodium
Selleck Chemicals	S4155	c(ccc(Cl)c1)(OC(=O)N2)c12	C7H4ClNO2	169.565	Chlorzoxazone
Selleck Chemicals	S4166	c1cc(S(=O)(=O)NC(NCCC)=O)ccc1Cl	C10H13ClN2O3S	276.74	Chlorpropamide
Selleck Chemicals	S4263	c1(C)cc(NC(=O)Cc2ccc(OC(C)(C)C(=O)[O-])cc2)cc(C)c1.[Na+]	C20H22NNaO4	363.383	Efaproxiral Sodium
Selleck Chemicals	S4176	C(N)(CO)(CO)CO	C4H11NO3	121.135	Trometamol
Selleck Chemicals	S4274	C1c2c(cccc2O)C[C@@H](N(CCC)CCc3cccs3)C1	C19H25NOS	315.473	Rotigotine
Selleck Chemicals	S4186	C(C)CCCCCCCCCC[N+](C)(C)CCOc1ccccc1.[Br-]	C22H40BrNO	414.463	Domiphen Bromide
Selleck Chemicals	S4285	c1cc(C(c2ccccc2)=C(c3ccccc3)CCCl)ccc1OCCO	C24H23ClO2	378.891	Ospemifene
Selleck Chemicals	S4195	c1c(Br)c2c(nccc2)c(O)c1Br	C9H5Br2NO	302.95	Broxyquinoline
Selleck Chemicals	S4295	c1c(Cl)c(Nc2c(C(O[Na])=O)cccc2)c(Cl)c(C)c1	C14H10Cl2NNaO2	318.13	Meclofenamate Sodium
Selleck Chemicals	S4204	[C@@H]1(O)[C@@]2([H])[C@@]([H])([C@H](O)CO2)OC1	C6H10O4	146.141	Isosorbide
Selleck Chemicals	S4904	N1(C(Oc2ccc([N+]([O-])=O)cc2)=O)CCC(C(c3ccc(c4c3)OCO4)(c5ccc(c6c5)OCO6)O)CC1	C27H24N2O9	520.487	JZL184
Selleck Chemicals	S4213	C1[C@H](C)O[C@@H](O[C@@H]2[C@H](C)[C@H](O[C@H]3C[C@@](C)(OC)[C@@H](O)[C@H](C)O3)[C@H](C)C(=O)O[C@@H](CC)[C@@](C)(O)C([H])([C@H](C)[C@]4([H])[C@H](C)C[C@@]2(O)C)O[C@H](COCOC)N4)[C@H](O)[C@H]1N(C)C	C42H78N2O14	835.074	Dirithromycin
Selleck Chemicals	S7008	n1c(N)c2c(n(C(C)(C)C)nc2c3ccc(Cl)cc3)nc1	C15H16ClN5	301.774	PP2
Selleck Chemicals	S4223	C1(=O)C[C@H](O)C[C@H](CC[C@H]2[C@@H](C)C=CC([C@@]23[H])=CCC[C@@H]3OC([C@@H](C)CC)=O)O1	C23H34O5	390.513	Mevastatin
Selleck Chemicals	S7028	c1c(Cl)c2c(C=C([C@@H](Nc3c4c([nH]cn4)ncn3)C)N(c5ccccc5)C2=O)cc1	C22H17ClN6O	416.863	IPI-145 (INK1197)
Selleck Chemicals	S4232	c1(NC(=O)C)ncc([N+]([O-])=O)s1	C5H5N3O3S	187.176	Nithiamide
Selleck Chemicals	S7048	c1c2c(C3=NNC2=O)c(N[C@H](c4ccc(F)cc4)[C@H]3c5n(C)ncn5)cc1F	C19H14F2N6O	380.351	BMN 673
Selleck Chemicals	S4244	c1(O)cc2c([nH]cc2CCN)cc1.Cl	C10H13ClN2O	212.676	Serotonin HCl

Selleck Chemicals	S7070	C1CN(c2cc(NCCC(=O)OCC)nc(c3ncccc3)n2)CCc4c1cccc4.Cl	C24H28ClN5O2	453.964	GSK J4 HCl
Selleck Chemicals	S4252	ClCCN(CCCl)C.Cl	C5H12Cl3N	192.514	Mechlorethamine HCl
Selleck Chemicals	S7086	[C@@](C)([C@H](C[C@H]12)C=C1)(C(=O)N(c3ccc(C(Nc4c5c(cccn5)ccc4)=O)cc3)C6=O)[C@@]26[H]	C26H21N3O3	423.463	IWR-1-endo
Selleck Chemicals	S3137	C(O)(=O)c1ccccc1O.[Na]	C7H6NaO3	161.111	Sodium salicylate
Selleck Chemicals	S4051	c1(OC)cc2c(cc(CCC(=O)C)cc2)cc1	C15H16O2	228.286	Nabumetone
Selleck Chemicals	S3150	c1(C(OC)=O)scc(C)c1NC(=O)C(NCCC)C.Cl	C13H21ClN2O3S	320.835	Articaine HCl
Selleck Chemicals	S4064	c1(C(c2ccc(F)cc2)(CCCN(C)C)OC3)c3cc(C#N)cc1.OC(C(O)=O)=O	C22H23FN2O5	414.427	Escitalopram Oxalate
Selleck Chemicals	S3167	C1CC(=C(C=C[C@@]2(C)[C@@]3([H])CC[C@]2(O)CC=C)[C@]34[H])C(C4)=CC1=O	C21H26O2	310.43	Altrenogest
Selleck Chemicals	S4076	c1cc2c(cccc2OCC(O)CNC(C)C)cc1.Cl	C16H22ClNO2	295.804	Propranolol HCl
Selleck Chemicals	S3183	C(N(C)C)CC=C1c2c(cccc2)CCc3c1cccc3.Cl	C20H24ClN	313.864	Amitriptyline HCl
Selleck Chemicals	S4085	c1(OC[C@@H](O)CNC(C)C)ccc(CCOCC2CC2)cc1.Cl	C18H30ClNO3	343.889	Levobetaxolol HCl
Selleck Chemicals	S3200	C(O)(=O)c1ccc(C(F)(F)F)cc1OC(C)=O	C10H7F3O4	248.155	Triflusal
Selleck Chemicals	S4096	c1cc2c(N(C3=CCN(CCCC(c4ccc(F)cc4)=O)CC3)C(=O)N2)cc1	C22H22FN3O2	379.427	Droperidol
Selleck Chemicals	S3604	[C@](O1)([C@@H]1[C@@H]2[C@@](C(C)C)(O2)[C@H]3O)([C@]4(C)[C@@]([H])(C5=C(C(=O)OC5)CC4)C6)[C@@]3(O7)[C@@H]67	C20H24O6	360.401	Triptolide (PG490)
Selleck Chemicals	S4105	c1(cc(C)c(NC(=O)c2cc(I)cc(I)c2[O-])cc1Cl)C(C#N)c3ccc(Cl)cc3.[Na+]	C22H13Cl2I2N2NaO2	685.056	Closantel Sodium
Selleck Chemicals	S4011	C1(OC(C)OC(=O)OCC)=C(C(=O)Nc2ncccc2)N(C)S(=O)(=O)c3c1cccc3	C20H21N3O7S	447.462	Ampiroxicam
Selleck Chemicals	S4114	c1ccc(Oc2c(Cl)cc3c(nc(SC)[nH]3)c2)c(Cl)c1Cl	C14H9Cl3N2OS	359.658	Triclabendazole
Selleck Chemicals	S4021	c1(cc(O)c(O)c([N+]([O-])=O)c1)C(=O)c2ccc(C)cc2	C14H11NO5	273.241	Tolcapone
Selleck Chemicals	S4128	c1(C(=O)NC2CNCCC2)cc(OC)c(OC)c(OC)c1	C15H22N2O4	294.346	Troxipide
Selleck Chemicals	S4034	C(c1ccccc1)(c2ccccc2)=C3CC[N+](C)(C)CC3.S([O-])(=O)(=O)OC	C21H27NO4S	389.508	Diphemanil Methylsulfate
Selleck Chemicals	S4143	c1cnc(C(c2ccccc2)(C(=O)N)CCN(C(C)C)C(C)C)cc1.P(O)(O)(O)=O	C21H32N3O5P	437.47	Pentoxyverine Citrate
Selleck Chemicals	S4043	N(CCN1)=C1C2CCCc(c23)cccc3.Cl	C13H17ClN2	236.74	Tetrahydrozoline HCl
Selleck Chemicals	S4159	c1cc(C(NCCc2ccc(OC(C(O)=O)(C)C)cc2)=O)ccc1Cl	C19H20ClNO4	361.819	Bezafibrate
Selleck Chemicals	S4167	n1c(NC2CC2)nc(N)nc1N	C6H10N6	166.184	Cyromazine
Selleck Chemicals	S4264	c1cc(OC(C)(C)C(=O)OCCOC(=O)c2cnccc2)ccc1Cl	C18H18ClNO5	363.792	Etofibrate
Selleck Chemicals	S4177	N1C(=O)NC=CC1=O	C4H4N2O2	112.087	Uracil
Selleck Chemicals	S4276	c(sc(CC)c1)(n2c(nnc2C)CN=C3c4c(Cl)cccc4)c13	C17H15ClN4S	342.846	Etizolam
Selleck Chemicals	S4187	C(Nc1ccccc1)(=O)c2ccccc2O	C13H11NO2	213.232	Salicylanilide
Selleck Chemicals	S4286	[C@H]1(O)NC(=O)[C@@H](N2C(=O)[C@H]([C@H](O)C)NC(=O)[C@H]([C@H](O)[C@@H](O)c3ccc(O)cc3)NC(=O)[C@@H](N4C(=O)[C@H]([C@H](O)C)NC(=O)[C@@H](NC(c5ccc(c6ccc(c7ccc(OCCCCC)cc7)cc6)cc5)=O)C[C@H]1O)C[C@@H](O)C4)[C@@H](O)[C@@H](C)C2	C58H73N7O17	1140.237	Anidulafungin (LY303366)
Selleck Chemicals	S4196	c1cc2c(nc3c(cc(OCC)cc3)c2N)cc1N.OC(C)C(=O)O.O	C18H23N3O5	361.392	Ethacridine lactate monohydrate
Selleck Chemicals	S4297	[C@H]1(O)[C@H](O)[C@H](CC(C)=CC(=O)OCCCCCCCCC(=O)O)OC[C@]1([H])C[C@H]2[C@]([C@@H](C)[C@@H](O)C)([H])O2	C26H44O9	500.622	Mupirocin
Selleck Chemicals	S4205	c1cc2c(c3c(cccc3)s2)cc1	C12H8S	184.257	Dibenzothiophene
Selleck Chemicals	S4907	c1(c2ccsc2)sc(C(N)=O)c(N)c1	C9H8N2OS2	224.303	SC-514

Selleck Chemicals	S4214	[C@H]1(Cl)[C@H](CO)O[C@H](O[C@@]2(CCl)[C@H](O)[C@H](O)[C@@H](CCl)O2)[C@H](O)[C@H]1O	C12H19Cl3O8	397.633	Sucralose
Selleck Chemicals	S7010	s1c(NC(=O)[C@@H]2CCCN2C(=O)[C@H](C3CCCCC3)NC(=O)[C@H](C)NC)c(c4ccccc4)nn1	C25H34N6O3S	498.641	GDC-0152
Selleck Chemicals	S4224	[C@@H](O)(CO)[C@@H](O)CO	C4H10O4	122.12	Erythritol
Selleck Chemicals	S7029	c1cc2c(C(Cc3cc(C(=O)N4CCC(OC)CC4)c(F)cc3)=NNC2=O)cc1	C22H22FN3O3	395.427	AZD2461
Selleck Chemicals	S4233	c(ccc(Cl)c1)(oc(N)n2)c12	C7H5ClN2O	168.58	Zoxazolamine
Selleck Chemicals	S7049	N(C(=O)c1sc(C)nc1)[C@@H](COC)C(=O)N[C@@H](COC)C(=O)N[C@@H](Cc2ccccc2)C(=O)C3(C)CO3	C25H32N4O7S	532.609	Oprozomib (ONX 0912)
Selleck Chemicals	S4245	[C@@]1([H])([C@@H](O)CO)OC(=O)C(O)C1O.[Na]	C6H10NaO6	201.13	Sodium ascorbate
Selleck Chemicals	S7071	[C@]1([H])([C@@]2([H])N(C)CCc3c2cc4c(OCO4)c3)OC(=O)c5c1ccc6c5OCO6	C20H17NO6	367.352	(+)-Bicuculline
Selleck Chemicals	S4253	N(C(N)=NC1)(c2c(cccc2)Cc3c4cccc3)C14.[H]Cl	C16H16ClN3	285.771	Epinastine HCl
Selleck Chemicals	S7087	c1(N2C[C@@H](C(NC3CCCCC3)=O)CC[C@H]2C)cc(c4ccc5c([nH]nc5N)c4)nc(NC)n1	C25H34N8O	462.59	GSK2334470
Selleck Chemicals	S3138	C1(=S)NC(C)=CC(=O)N1	C5H6N2OS	142.179	Methylthiouracil
Selleck Chemicals	S4053	c(cccc1)([C@H](NC)CC[C@H]2c3cc(Cl)c(Cl)cc3)c12.Cl	C17H18Cl3N	342.691	Sertraline HCl
Selleck Chemicals	S3151	c1(S(NC(NC2CCCCC2)=O)(=O)=O)ccc(CCN3C(=O)C(C)(C)c(c4C3=O)ccc(OC)c4)cc1	C27H33N3O6S	527.632	Gliquidone
Selleck Chemicals	S4065	c1c(Cl)c(C=NNC(=N)N)c(Cl)cc1.CC(=O)O	C10H12Cl2N4O2	291.134	Guanabenz Acetate
Selleck Chemicals	S3170	N1([C@]2([H])SC(C)(C)[C@@H]1C([O-])=O)C(=O)[C@H]2NC(=O)[C@H](N)c3ccccc3.[Na+]	C16H18N3NaO4S	371.387	Ampicillin sodium
Selleck Chemicals	S4077	c1c(O)ccc(OC)c1	C7H8O2	124.137	Mequinol
Selleck Chemicals	S3185	O=C(c1cc(O)c(O)cc1)CNC.Cl	C9H12ClNO3	217.649	Adrenalone HCl
Selleck Chemicals	S4086	C1(N2CCN(C)CC2)=Nc3c(cccc3)Oc4c1cc(Cl)cc4.C(C(=O)O)CC(=O)O	C22H24ClN3O5	445.896	Loxapine Succinate
Selleck Chemicals	S3201	c1c(c2ccc1C(F)(F)F)N(CCCN3CCN(C)CC3)c(c4S2)cccc4.Cl.Cl	C21H26Cl2F3N3S	480.417	Trifluoperazine 2HCl
Selleck Chemicals	S4097	C1C[C@]2(C)C(C=C[C@]3([H])[C@]2([H])CC[C@@]4(C)[C@@]3([H])CC[C@]4(C(C)=O)[H])=CC1=O	C21H28O2	312.446	Dydrogesterone
Selleck Chemicals	S4000	c(c1[nH]c2)(c2C[C@]3([H])[C@]4([H])C[C@H](CSC)CN3CCC)c4ccc1.CS(=O)(=O)O	C20H30N2O3S2	410.594	Pergolide mesylate
Selleck Chemicals	S4106	c1(cc(C)c(NC(=O)c2cc(I)cc(I)c2O)cc1Cl)C(C#N)c3ccc(Cl)cc3	C22H14Cl2I2N2O2	663.074	Closantel
Selleck Chemicals	S4012	C1NCCC(=C2c3c(cccn3)CCc4c2ccc(Cl)c4)C1	C19H19ClN2	310.821	Desloratadine
Selleck Chemicals	S4116	CC(C)CC(=O)N	C5H11NO	101.147	Isovaleramide
Selleck Chemicals	S4022	c1cc(C(O)=O)ccc1S(=O)(=O)N(CCC)CCC	C13H19NO4S	285.359	Probenecid
Selleck Chemicals	S4131	c1cc(N2CCN(C[C@@H](O)CO)CC2)ccc1	C13H20N2O2	236.31	Levodropropizine
Selleck Chemicals	S4035	[C@](C)([C@@H]([C@H](C)C=C[C@H](C)C(C)C)CC1)(CCCC2=CC=C3C[C@H](O)CCC3=C)[C@@]12[H]	C28H44O	396.648	Vitamin D2
Selleck Chemicals	S4144	c1c(C)[n+](Cc2c(N)nc(CCC)nc2)ccc1.Cl.[Cl-]	C14H20Cl2N4	315.241	Amprolium HCl
Selleck Chemicals	S4044	c1cc(SC(F)(F)F)ccc1Oc2ccc(N3C(=O)NC(=O)N(C)C3=O)cc2C	C18H14F3N3O4S	425.382	Toltrazuril
Selleck Chemicals	S4160	N([C@@H](C([O-])=O)C(C)(C)S1)(C(=O)[C@H]2NC(=O)Cc3ccccc3)[C@]12[H].[Na+]	C16H17N2NaO4S	356.372	Penicillin G Sodium
Selleck Chemicals	S4169	c1(NC(=O)C(C#N)=C(O)C)ccc(C(F)(F)F)cc1	C12H9F3N2O2	270.207	Teriflunomide
Selleck Chemicals	S4265	c1(cnccc1)C(=O)NCC(C)NC(=O)c2cnccc2	C15H16N4O2	284.313	Nicaraven
Selleck Chemicals	S4178	C(C(=O)C(C)(C)C)(Oc1ccc(Cl)cc1)n2cncc2	C15H17ClN2O2	292.761	Climbazole
Selleck Chemicals	S4277	c1(OC(N(C)C)=O)cc(C(O)CNC(C)(C)C)cc(OC(=O)N(C)C)c1.Cl	C18H30ClN3O5	403.901	Bambuterol HCl

Selleck Chemicals	S4188	C(Oc1ccccc1C(O)=O)(=O)c2ccccc2O	C14H10O5	258.226	Sasapyrine
Selleck Chemicals	S4288	c1(N(CCCl)CCCl)ccc(CCCC(=O)O)cc1	C14H19Cl2NO2	304.212	Chloroambucil
Selleck Chemicals	S4197	C1(=O)NC(=O)CC(C)(CC)C1	C8H13NO2	155.194	Bemegride
Selleck Chemicals	S4299	c1cc2c(C(O)=C(CC3=C(O)c4c(cccc4)OC3=O)C(=O)O2)cc1	C19H12O6	336.295	Dicoumarol
Selleck Chemicals	S4206	C(N)CS.Cl	C2H8ClNS	113.61	Cysteamine HCl
Selleck Chemicals	S4908	c1(nc2c(cc(O)cc2)c3CC)c3CN4C1=CC5=C(COC(=O)[C@]5(O)CC)C4=O	C22H20N2O5	392.405	SN-38
Selleck Chemicals	S4216	[C@]([H])(C(=O)CC1)([C@@]2(C)[C@H](OC(=O)CSC(C)(CNC(=O)[C@H](N)C(C)C)C)C[C@@](C)(C=C)[C@@H](O)[C@@H]3C)[C@@]13CC[C@H]2C.Cl	C31H53ClN2O5S	601.281	Valnemulin HCl
Selleck Chemicals	S7015	CN[C@@H](C)C(=O)N[C@H](C(=O)N1C[C@@H](O)C[C@H]1Cc2c3c(cc(F)cc3)[nH]c2c4c(C[C@H]5N(C(=O)[C@H](CC)NC(=O)[C@H](C)NC)C[C@@H](O)C5)c6c(cc(F)cc6)[nH]4)CC	C42H56F2N8O6	806.941	Birinapant
Selleck Chemicals	S4225	c1(OCC(N)C)c(C)cccc1C.Cl	C11H18ClNO	215.72	Mexiletine HCl
Selleck Chemicals	S7033	n1c(N)c(c(c2ccc(N(C(Cc3nc(C)ccc3)=O)CC4)c4c2F)cn5C)c5nc1	C23H21FN6O	416.451	GSK2656157
Selleck Chemicals	S4235	c1ccc(N=Nc2ccc(N)nc2N)cc1.Cl	C11H12ClN5	249.699	Phenazopyridine HCl
Selleck Chemicals	S7050	c1c(N2CCOC[C@H]2C)nc(c3c4c([nH]cc4)ccc3)nc1C5(CC5)S(=O)(=O)C	C21H24N4O3S	412.505	AZ20
Selleck Chemicals	S4246	Cl.c1cc([C@H]2C[C@@H]2N)ccc1	C9H12ClN	169.651	Tranylcypromine (2-PCPA) HCl
Selleck Chemicals	S7072	[C@H](NC)(C(=O)O)CC(=O)O	C5H9NO4	147.129	NMDA (N-Methyl-D-aspartic acid)
Selleck Chemicals	S4255	c1(NC(C)CCCN(CC)CC)c2c(cc(Cl)cc2)nc3c1cc(OC)cc3.Cl.Cl	C23H32Cl3N3O	472.879	Quinacrine 2HCl
Selleck Chemicals	S7088	C1CN(C2CCN(C(c3ccc(C(N4CCC(N5CCCC5)CC4)=O)c(Nc6ccccc6)c3)=O)CC2)CC1	C32H43N5O2	529.716	UNC1215
Selleck Chemicals	S7090	C(N[C@@H](Cc1ccc(c2cn(c3n2)cccc3[C@@H](O)C)cc1)CNC(=O)CN(C)C)(=O)c4ccc(OC(C)C)c(Cl)c4	C32H38ClN5O4	592.128	GSK923295
Selleck Chemicals	S7251	c1(C(F)(F)F)cc(c2nn(C=CC(=O)N3CC(F)(F)C3)cn2)cc(C(F)(F)F)c1	C16H10F8N4O	426.264	KPT-276
Selleck Chemicals	S7099	c1(N2CC[C@]3(O)C2=Nc4c(cc(C)cc4)C3=O)ccccc1	C18H16N2O2	292.332	(-)-Blebbistatin
Selleck Chemicals	S7263	c1cc2c(c(Sc3ccc(Cl)cc3)c(C)n2CC(O)=O)c(NC(=O)C)c1	C19H17ClN2O3S	388.868	AZD1981
Selleck Chemicals	S7114	C1CC(COc2c(N=O)c(N)nc(N)n2)CCC1	C11H17N5O2	251.285	NU6027
Selleck Chemicals	S7279	c1cc2c(nc(N3CCN(C(=O)c4c(n5nccn5)ccc(C)c4)[C@H](C)CC3)o2)cc1Cl	C23H23ClN6O2	450.921	Suvorexant (MK-4305)
Selleck Chemicals	S7129	N1C(=O)C(=Cc2ccc([N+]([O-])=O)o2)C(=O)N1c3ccc(C(OCC)=O)cc3	C17H13N3O7	371.301	PYR-41
Selleck Chemicals	S7293	c1c(C)nc(NS(=O)(=O)c2ccc(NC(=S)NC(=O)COc3ccc(Br)cc3Cl)cc2)nc1C	C21H19BrClN5O4S2	584.894	ZCL278
Selleck Chemicals	S7139	c1cc(C=C2SC(=S)N(c3cc(C(F)(F)F)ccc3)C2=O)ccc1C(=O)O	C18H10F3NO3S2	409.402	CFTRinh-172
Selleck Chemicals	S7310	c(cccc1)(c2c(cc(NC(=O)C(C)(C)C)cc2)C(=O)C3=O)c13	C19H17NO3	307.343	SF1670
Selleck Chemicals	S7149	c1nc(C(C)=NNC(=S)N2CCC2)ccc1	C11H14N4S	234.321	NSC 319726
Selleck Chemicals	S7327	n1(c2ccc(OC)cc2)c(c3c([N+]([O-])=O)c1C)cc(O)cc3	C16H14N2O4	298.293	ID-8
Selleck Chemicals	S7167	c1ccn2c(c(OC)c(C)c2C(c3cc(C(=O)O[Na])c(N)cc3)=O)c1	C18H15N2NaO4	346.312	SSR128129E
Selleck Chemicals	S7340	c1c(Cl)c(c2nc(N)nc(SCCCC(=O)N)n2)c3c(c4ccc3)c1COC4	C19H18ClN5O2S	415.897	CH5138303
Selleck Chemicals	S7193	C1C(=O)Nc2c(nccc2)c3c1c4c(ccc(Br)c4)[nH]3	C15H10BrN3O	328.163	1-Azakenpaullone
Selleck Chemicals	S7364	CN(c1ccc(c2cccc(NC(N(C)C)=O)c2)cc1)C	C17H21N3O	283.368	Atglistatin
Selleck Chemicals	S7209	c1(c2ccccc2)cc3c([nH]cc3c4ccc(C(O)=O)c(C5CCCC5)c4)nc1	C25H22N2O2	382.454	GSK650394

Selleck Chemicals	S7378	c1c(S(=O)(=O)F)ccc(CCN)c1.Cl	C8H11ClFNO2S	239.695	AEBSF HCl
Selleck Chemicals	S7229	c1(NC(=O)C=Cc2cn(CC=Cc3ccccc3)nc2)c(N)cc(F)cc1	C21H19FN4O	362.4	RGFP966
Selleck Chemicals	S7399	CC(NC1=C(C(=O)CC(C)(C)C1)C2c3ccc([N+]([O-])=O)cc3)=C2C(OC4CCCCC4)=O	C25H30N2O5	438.516	FLI-06
Selleck Chemicals	S7430	c1cc(Oc2ccc(S(=O)(=O)CC3CS3)cc2)ccc1	C15H14O3S2	306.4	SB-3CT
Selleck Chemicals	S8032	n1cc(C(=O)N)c(Nc2cccc(n3nccn3)c2)nc1N[C@H]4[C@H](N)CCCC4.Cl	C19H24ClN9O	429.907	PRT062607 (P505-15, BIIB057) HCl
Selleck Chemicals	S7448	[Ru]1(Cl)(OC(=O)CN1)(C=O)(C=O)C=O.[R]	C5H6ClNO5RRu	296.628	CORM-3
Selleck Chemicals	S8042	n1c(N)c(Cc2cc(OC)c(OCc3ccc(OC)cc3)cc2)cnc1N	C20H22N4O3	366.414	GW2580
Selleck Chemicals	S7461	c1ccc(Nc2ncnc(c3c(OC)cccc3)c2)cc1CS(=O)(=O)N	C18H18N4O3S	370.426	LDC000067
Selleck Chemicals	S8057	c1c2nc(Nc3cc(c(OCCN4CCCC4)cc3)COCC=CCOCc5cc2ccc5)nc1	C28H32N4O3	472.579	Pacritinib (SB1518)
Selleck Chemicals	S7489	c1cc(Cl)ccc1CN(C=O)C(c2c3c(cc(Cl)cc3)[nH]c2C(=O)OCC)C(NC(C)(C)C)=O	C25H27Cl2N3O4	504.406	YH239-EE
Selleck Chemicals	S8077	c1(Cl)c(Cl)ccc(N2C(=O)C(Cl)=C(N3CCOCC3)C2=O)c1	C14H11Cl3N2O3	361.608	RI-1
Selleck Chemicals	S7505	CC(Oc1cc(c2cnn(C3CCNCC3)c2)cnc1N)c4c(Cl)c(F)ccc4Cl	C21H22Cl2FN5O	450.337	(S)-crizotinib
Selleck Chemicals	S7520	C1(=O)C2=C(CCC2)N(CC(=O)N(CCN(CC)CC)Cc3ccc(c4ccc(C(F)(F)F)cc4)cc3)C(SCc5ccc(F)cc5)=N1	C36H38F4N4O2S	666.771	Darapladib (SB-480848)
Selleck Chemicals	S7546	c1ccc(c2ccc(CC(=O)N(C)c3nc(C)c(S(N)(=O)=O)s3)cc2)nc1	C18H18N4O3S2	402.491	Pritelivir (BAY 57-1293)
Selleck Chemicals	S7635	N1(CCCCC2(C(NCC(F)(F)F)=O)c3c(cccc3)c4c2cccc4)CCC(NC(=O)c5ccccc5c6ccc(C(F)(F)F)cc6)CC1	C39H37F6N3O2	693.72	Lomitapide
Selleck Chemicals	S8007	c1(C(Nc2ccccc2)=O)nc(c3ccc(S(C)(=O)=O)cc3)cnc1N	C18H16N4O3S	368.41	VE-821
Selleck Chemicals	S8021	c1(C)cc(C)c(Sc2c(N3CCNCC3)cccc2)cc1.Br	C18H23BrN2S	379.358	Vortioxetine (Lu AA21004) HBr
Selleck Chemicals	S7091	[C@H]1(C)[C@]2(O)O[C@H](C[C@H](OC)C(C)=CC=CC=C[C@@H](C)C[C@@H](C)C(=O)[C@H](OC)[C@H](O)C(C)=C[C@@H](C)C(=O)C[C@@H]([C@H](C)C[C@H]3C[C@@H](OC)[C@@H](n4cnnn4)CC3)OC(=O)[C@@]5([H])N(CCCC5)C(=O)C2=O)CC1	C52H79N5O12	966.21	Zotarolimus(ABT-578)
Selleck Chemicals	S7252	c1(C(F)(F)F)cc(c2nn(C=CC(NNc3nccnc3)=O)cn2)cc(C(F)(F)F)c1	C17H11F6N7O	443.306	KPT-330
Selleck Chemicals	S7102	n1c(N)c(c2onc(c3ccc(CNC)cc3)c2)nc(c4ccc(S(C(C)C)(=O)=O)cc4)c1	C24H25N5O3S	463.552	VE-822
Selleck Chemicals	S7265	C1CCC(C(NC(c2ccc(F)cc2)c3ccc(F)cc3)=O)(NC(=O)[C@H](CCCNC(=N)N)NC(C(CC)(CC)NC(C(C)C)=O)=O)C1	C35H49F2N7O4	669.805	MM-102
Selleck Chemicals	S7115	c1(C(F)(F)F)ccc(c2cc(Oc3c4c(sc(NC(=O)C)n4)ccc3)ncn2)cc1	C20H13F3N4O2S	430.403	AMG-517
Selleck Chemicals	S7280	C(=O)(C(=O)Nc1ncc(Cl)cc1)N[C@H]2CC[C@@H](C(=O)N(C)C)C[C@@H]2NC(=O)c3sc4c(CCN(C)C4)n3	C24H30ClN7O4S	548.058	Edoxaban
Selleck Chemicals	S7130	c1(SC#N)cc(SC#N)c(N)nc1N	C7H5N5S2	223.278	PR-619
Selleck Chemicals	S7295	c1(OC)cc2c(C(=O)NC(c3cc(C)c(OCCO)c(C)c3)=N2)c(OC)c1	C20H22N2O5	370.399	RVX-208
Selleck Chemicals	S7140	c1(Cl)c(Cl)c2c(C(=O)CC2=O)c(Cl)c1Cl	C9H2Cl4O2	283.923	TCID
Selleck Chemicals	S7316	c1ccc(CNC(=O)c2ccc(c3nc(CS(=O)(=O)c4ccc(C)cc4)c(C)o3)cc2)cn1	C25H23N3O4S	461.533	STF-118804
Selleck Chemicals	S7152	Cc1cc(c2oc(C=C3C(=O)N(c4ccc(C(O)=O)cc4)N=C3C)cc2)c([N+]([O-])=O)cc1C	C24H19N3O6	445.424	C646
Selleck Chemicals	S7329	c1ccc(Oc2ccc(CNC(=O)c3nc(c4cc(Br)c(O)c(Br)c4)no3)cc2)cc1	C22H15Br2N3O4	545.18	IOWH032
Selleck Chemicals	S7172	N(C(=O)CN1CCOCC1)[C@@H](C)C(=O)N[C@@H](Cc2ccc(OC)cc2)C(=O)N[C@@H](Cc3ccccc3)C(=O)[C@]4(C)CO4	C31H40N4O7	580.672	ONX-0914 (PR-957)
Selleck Chemicals	S7351	c1(NCCCc2ccccc2)ccc(C)cc1N	C16H20N2	240.343	JSH-23
Selleck Chemicals	S7194	c1c(C)c(C#Cc2cnc3c(cn[nH]3)c2)cc(C(=O)Nc4ccc(CN5CCN(C)CC5)c(C(F)(F)F)c4)c1.CS(O)(=O)=O.CS(O)(=O)=O	C31H35F3N6O7S2	724.771	GZD824
Selleck Chemicals	S7365	c1cc(C(=O)c2ccc(OCC(NC3CCN(Cc4ccccc4)CC3)=O)cc2)ccc1	C27H28N2O3	428.523	AdipoRon

Selleck Chemicals	S7213	[C@H]1(CO)O[C@@]2([H])[C@]([H])(N=C(NCC)S2)[C@@H](O)[C@@H]1O	C9H16N2O4S	248.299	Thiamet G
Selleck Chemicals	S7379	c1c(O)c2c([C@H](C)[C@]3([H])C(=C(O)[C@@]4(O)[C@]([H])([C@H](N(C)C)C(O)=C(C(N)=O)C4=O)[C@H]3O)C2=O)cc1.c5c(O)c6c([C@H](C)[C@]7([H])C(=C(O)[C@@]8(O)[C@]([H])([C@H](N(C)C)C(O)=C(C(N)=O)C8=O)[C@H]7O)C6=O)cc5.Cl.O.CCO.Cl	C46H58Cl2N4O18	1025.875	E-64
Selleck Chemicals	S7233	c1c(c2cc(NC(OCC)=O)c3n(c(C)nn3)n2)cc(NS(=O)(=O)C)c(C)c1	C17H20N6O4S	404.443	Bromosporine
Selleck Chemicals	S7414	C(OCCc1ccccc1)(=O)C=Cc2ccc(O)c(O)c2	C17H16O4	284.307	Caffeic Acid Phenethyl Ester
Selleck Chemicals	S7434	c1cc2c(cc(C[C@H](NC(=O)C(CC(=O)NO)CC(C)C)C(=O)N[C@@H](C)C(=O)NCCN)cc2)cc1	C26H37N5O5	499.602	TAPI-1
Selleck Chemicals	S8034	c1cc(NC(=O)C)c2c(C(=O)N([C@H](CS(=O)(C)=O)c3ccc(OC)c(OCC)c3)C2=O)c1	C22H24N2O7S	460.5	Apremilast (CC-10004)
Selleck Chemicals	S7449	c1cc([N+]([O-])=O)c2c(cc(C(O)=O)[nH]2)c1	C9H6N2O4	206.155	CRT0044876
Selleck Chemicals	S8043	c1c2c(c3C(=O)N(CCCCCC(=O)NO)C2=O)c(ccc3)cc1	C18H18N2O4	326.347	Scriptaid
Selleck Chemicals	S7462	c1cc(OCC(=O)N(C(C)C)Cc2onc(c3cnccc3)n2)ccc1CCC	C22H26N4O3	394.467	PI-1840
Selleck Chemicals	S8058	c1ccc(C(Oc2c(c(O)cc(O)c2[C@@H]3CCN(C)[C@H]3CO)C4=O)=C4)c(Cl)c1.Cl	C21H21Cl2NO5	438.301	P276-00
Selleck Chemicals	S7490	c1cc2c(c3C(=O)N(CCCSc4nnc(c5ccncc5)n4c6ccc(OC)cc6)C2=O)c(ccc3)c1	C29H23N5O3S	521.59	WIKI4
Selleck Chemicals	S8078	C1[C@@]([H])([C@]2(C(OC)=O)CCC1(C)C)[C@@]([H])([C@@]3(C)CC2)C(=O)C=C([C@]34C)[C@@](C)([C@@]5([H])CC4)C=C(C#N)C(=O)C5(C)	C32H43NO4	505.688	Bardoxolone Methyl
Selleck Chemicals	S7508	C1Cc2c(sc(NC(=O)c3c4c(cccc4)ccc3)c2C#N)CC1	C20H16N2OS	332.419	JNK Inhibitor IX
Selleck Chemicals	S7523	C1N(c2ccc(Nc3c4n(ccn4)cc(c5ccc6c([nH]nc6)c5)n3)cc2)CCOC1	C23H21N7O	411.459	GS-9973
Selleck Chemicals	S7565	c1(OC)c(N(c2nc(Nc3ccc(N4CCN(C)CC4)cc3)ncc2)C(Oc5c(C)cccc5C)=O)ccc(OC)c1	C32H36N6O4	568.666	WH-4-023
Selleck Chemicals	S8000	c1cc(C(=O)NC(=S)Nc2ccc(NC(=O)C)cc2)ccc1C(C)(C)C	C20H23N3O2S	369.481	Tenovin-1
Selleck Chemicals	S8009	c1(ccc(O)c(O)c1)C=C(C#N)C#N	C10H6N2O2	186.167	AG-18
Selleck Chemicals	S8022	[C@H]1(CO)O[C@@H](c2ccc(Cl)c(Cc3ccc(O[C@@H]4COCC4)cc3)c2)[C@H](O)[C@@H](O)[C@@H]1O	C23H27ClO7	450.909	Empagliflozin (BI 10773)
Selleck Chemicals	S7092	c1c(n2c(C)c(C=NN3CCN(Cc4ccccc4)CC3)c(C)n2)cccc1	C23H27N5	373.494	SANT-1
Selleck Chemicals	S7253	c1nc(N)c(C(Nc2cccnc2)=O)nc1c3ccc(S(N4CCN(C)CC4)(=O)=O)cc3	C21H23N7O3S	453.517	AZD2858
Selleck Chemicals	S7104	c1c(C=C2SC(=O)NC2=O)c(N3CCC[C@@H](N)C3)c(c4ccccc4)cc1	C21H21N3O2S	379.475	AZD1208
Selleck Chemicals	S7266	n1cc(C2[C@]3([H])[C@]([H])(C=CC3)c4c(c(F)cc(F)c4)N2)ccc1	C17H14F2N2	284.303	Golgicide A
Selleck Chemicals	S7120	c1cc2c(ncn2[C@H]3[C@@H](O)[C@H](O)C(CO)=C3)c(N)n1	C12H14N4O3	262.265	3-Deazaneplanocin A (DZNeP)
Selleck Chemicals	S7282	CN1CCC(NC(C(NOC2=C3C(Oc4ccc([N+]([O-])=O)cc4)=CC(=O)C=C3O)=C2)=O)CC1	C22H22N4O7	454.433	NMS-E973
Selleck Chemicals	S7132	c1c([N+]([O-])=O)c(Sc2c(Cl)c(Cl)ccc2)sc1C(C)=O	C12H7Cl2NO3S2	348.225	P5091 (P005091)
Selleck Chemicals	S7297	c1c(c2c3c(cccc3)n(C)c2)nc(Nc4cc(NC(=O)C=C)c(N(C)CCN(C)C)cc4OC)nc1	C28H33N7O2	499.607	AZD9291
Selleck Chemicals	S7142	Cc1ccc(S(c2oc([N+]([O-])=O)cc2)(=O)=O)cc1	C11H9NO5S	267.258	NSC697923
Selleck Chemicals	S7317	n1cc(Cl)c(Oc2cccc(NC(=O)CC)c2)nc1Nc3ccc(N4CCN(C)CC4)cc3OC	C25H29ClN6O3	496.989	WZ4003
Selleck Chemicals	S7153	c1cc(C=C2SC(=S)NC2=O)ccc1CC	C12H11NOS2	249.352	10058-F4
Selleck Chemicals	S7330	c1(Cl)ccc(SCC(=O)N2CCC(C(=O)NCCSSCCN(C)C)CC2)cc1.C(C(O)=O)(F)(F)F	C22H31ClF3N3O4S3	590.142	6H05
Selleck Chemicals	S7173	c1(F)c(Nc2cccc(NC(=O)C=C)c2)nc(Nc3ccc(OCCOC)cc3)nc1	C22H22FN5O3	423.44	AVL-292
Selleck Chemicals	S7353	C(CN(C(C)C)C[C@H]1O[C@@H](n2ccc3c2ncnc3N)[C@H](O)[C@@H]1O)CNC(=O)Nc4ccc(C(C)(C)C)cc4	C28H41N7O4	539.67	EPZ004777
Selleck Chemicals	S7195	c1cc(c2csc(NC(=O)NCc3cc(O)ccc3)n2)ccn1	C16H14N4O2S	326.373	RKI-1447

Selleck Chemicals	S7367	n1cc(C(F)(F)F)c(NC)nc1Nc2c(C)nn(C(C)(C)C#N)c2	C14H16F3N7	339.319	GNE-0877
Selleck Chemicals	S7214	c(cc(Nc1ccc(F)cc1F)cc2)(CCc3c(cc(OC[C@H](O)CO)cc3)C4=O)c24	C24H21F2NO4	425.425	Skepinone-L
Selleck Chemicals	S7380	C(C[C@@H](C(=O)N[C@H](C(=O)N[C@H](C([H])=O)CCCNC(=N)N)CC(C)C)NC(=O)C)(C)C.[R]	C20H38N6O4R	426.554	Leupeptin Hemisulfate
Selleck Chemicals	S7234	c1nc2c(c(C(=O)O)ccc2O)cc1	C10H7NO3	189.167	IOX1
Selleck Chemicals	S7417	Cl.Cl.n1c(N(C)C)c2c(n([C@@H]3O[C@@H](CO)[C@H](NC(=O)[C@@H](N)Cc4ccc(OC)cc4)[C@@H]3O)cn2)nc1	C22H31Cl2N7O5	544.431	Puromycin 2HCl
Selleck Chemicals	S7435	c1nc(NC(=O)NCc2ccc(OC)cc2)sc1[N+]([O-])=O	C12H12N4O4S	308.313	AR-A014418
Selleck Chemicals	S8035	c1nc(C(=O)Nc2ccc(S(Nc3ccccc3Cl)(=O)=O)c(Cl)c2)ccc1	C18H13Cl2N3O3S	422.285	VU 0364439
Selleck Chemicals	S7450	CC(Nc1ccc(Cc2ccc(NC(C)=O)cc2)cc1)=O	C17H18N2O2	282.337	FH1(BRD-K4477)
Selleck Chemicals	S8044	c1(C)cc2c(nc(NCCN)c3n2c(C)cn3)cc1	C14H17N5	255.318	BMS-345541
Selleck Chemicals	S7467	C1N(C(c2c(c3c(c4ccc3)cccc4)cn(Cc5n(Cc6ccc(Br)cc6)cnc5)c2)=O)CCOC1	C30H27BrN4O2	555.465	LB42708
Selleck Chemicals	S8059	N1(C(=O)N2CCNC(=O)C2)[C@@H](c3ccc(Cl)cc3)[C@H](c4ccc(Cl)cc4)N=C1c5c(OC(C)C)cc(OC)cc5	C30H30Cl2N4O4	581.49	Nutlin-3a
Selleck Chemicals	S7493	C(=O)(c1ccccc1)Nc2nc(c3ccc(C)cc3C)cs2	C18H16N2OS	308.397	INH1
Selleck Chemicals	S7509	Cc1oc(CNc2c3c(ccc(c4oc(CO)cc4)c3)ncn2)cc1	C19H17N3O3	335.357	ML167
Selleck Chemicals	S7525	C1N(c2cc(OCC)c(Nc3ncc4c(N(C)c5c(cccc5)C(=O)N4C)n3)cc2)CCC(O)C1	C26H30N6O3	474.555	XMD8-92
Selleck Chemicals	S7566	C1(=O)N(C)C(=O)C(NCCc2ccc(F)cc2)=C1c3c(C)[nH]c4c3cccc4	C22H20FN3O2	377.412	IM-12
Selleck Chemicals	S8001	c1cc(N(c2ncc(C(NCCCCCCC(=O)NO)=O)cn2)c3ccccc3)ccc1	C24H27N5O3	433.503	Rocilinostat (ACY-1215)
Selleck Chemicals	S8010	C(O)(=O)C=CC(O)=O.C(#N)c1cc(CN2CCC(Nc3ncnc4c3cc(Cl)s4)CC2)ccc1F	C23H21ClFN5O4S	517.96	PRX-08066 Maleic acid
Selleck Chemicals	S8023	c(CCCC1)(sc(NC(=O)c2ccc(OC)c(OC)c2)c3C(N)=O)c13	C18H20N2O4S	360.427	TCS 359
Selleck Chemicals	S7093	S(c1c(c2ccc1O)cccc2)Sc3c(c4ccc3O)cccc4	C20H14O2S2	350.454	IPA-3
Selleck Chemicals	S7255	c1(OC(F)(F)F)c(Nc2ncc3c(c4c(c(C(=O)N)nn4CCO)CC3)n2)cc(N5CCN(C)CC5)cc1	C24H27F3N8O3	532.518	NMS-P937 (NMS1286937)
Selleck Chemicals	S7106	c([nH]cc1c2nc(Nc3ccc(N4CCC(N)CC4)cc3OC)ncc2Cl)(cccc5)c15	C24H25ClN6O	448.948	AZD3463
Selleck Chemicals	S7269	C1(=O)N(C)C2C(C=NC(Nc3cc(SC)ccc3)=N2)C=C1c4c(Cl)cccc4Cl	C21H18Cl2N4OS	445.365	PD173955
Selleck Chemicals	S7122	c1(N(C2CC3)C3CC(NC(c4c(C)cc(C(=O)N)c(N[C@H](C)CC)c4)=O)C2)ccc(C(C5CC5)=O)cn1	C29H37N5O3	503.636	XL888
Selleck Chemicals	S7284	c1(C(F)(F)F)c(Nc2cc(NC(C=C)=O)ccc2)nc(Nc3ccc(N4CCN(C(=O)C)CC4)cc3OC)nc1	C27H28F3N7O3	555.552	CO-1686 (AVL-301)
Selleck Chemicals	S7133	c1c([N+]([O-])=O)c(Sc2c(F)cc(F)cc2)sc1C(C)=O	C12H7F2NO3S2	315.316	P22077
Selleck Chemicals	S7300	c1(cc(NC(=O)CN(C)C)cc2)c2NC(=O)c3c1cccc3.Cl	C17H18ClN3O2	331.797	PJ34 HCl
Selleck Chemicals	S7143	c1(c2cc(C)ncc2)ncc(CC(=O)Nc3ncc(c4nccnc4)cc3)cc1C	C23H20N6O	396.444	LGK-974
Selleck Chemicals	S7319	c1(NCCCN2CCOCC2)nccc(Nc3cc(c4cc3)c(c5n4CC)cccc5)n1	C25H30N6O	430.545	EHop-016
Selleck Chemicals	S7155	ONC(=O)[C@@H](CSc1cccs1)[C@H](CC(C)C)C(=O)N[C@H](Cc2ccccc2)C(NC)=O	C23H31N3O4S2	477.64	Batimastat (BB-94)
Selleck Chemicals	S7332	c1(Cl)cc(OC)c(NCC(=O)N2CCC(NS(=O)(=O)C=C)CC2)cc1I	C16H21ClIN3O4S	513.778	K-Ras(G12C) inhibitor 9
Selleck Chemicals	S7176	c1(csc(Nc2ccc(O)cc2)n1)c3ccc(Cl)cc3	C15H11ClN2OS	302.779	SKI II
Selleck Chemicals	S7355	c(ccc(Cl)c1)(C(=O)N(Nc2ccccc2)C([C@@H](CC#C)N(CCCN)C(c3c(F)c(Cl)ccc3)=O)=N4)c14	C28H24Cl2FN5O2	552.427	ARQ 621
Selleck Chemicals	S7198	c1cc2c(NC(=C3c4c(cc(Br)cc4)NC3=O)C2=NO)cc1	C16H10BrN3O2	356.173	BIO
Selleck Chemicals	S7368	n1cc(C(F)(F)F)c(NC)nc1Nc2cnn([C@@H]3[C@H](F)CN(C4COC4)CC3)c2Cl	C17H20ClF4N7O	449.834	GNE-9605

Selleck Chemicals	S7215	c1(C(=O)NC2CC2)cc(c3ncc(C(=O)NCC(C)(C)C)cc3)c(C)c(F)c1	C22H26FN3O2	383.459	Losmapimod (GW856553X)
Selleck Chemicals	S7382	c1cc2c(c(C[C@H](NC(=O)[C@H](CC(C)C)NP(=O)(O)O[C@@H]3O[C@@H](C)[C@H](O)[C@@H](O)[C@H]3O)C(O)=O)c[nH]2)cc1.[Na][Na]	C23H34N3Na2O10P	589.483	Phosphoramidon Disodium Salt
Selleck Chemicals	S7237	C1C(N)C1c2ccc(c3cccc(O)c3)cc2	C15H15NO	225.286	OG-L002
Selleck Chemicals	S7421	c1c(Nc2[nH]nc(c23)ncnc3N)ccc(F)c1	C11H9FN6	244.228	CGP 57380
Selleck Chemicals	S7436	CCCCCCCCCCCCCCCCn1c(C)[n+](Cc2ccccc2)cc1.[I-]	C27H45IN2	524.564	NH125
Selleck Chemicals	S8036	c1(C(=O)C=Cc2ccc(O)c(O)c2)ccc(O)cc1O	C15H12O5	272.253	Butein
Selleck Chemicals	S7451	Cc1ccc(Cl)cc1N(S(C)(=O)=O)CC(Nc2c(F)cccc2F)=O	C16H15ClF2N2O3S	388.817	FPH1 (BRD-6125)
Selleck Chemicals	S8047	c1(O)ccc(C=NNC(c2cc3c(cccc3)cc2O)=O)cc1O	C18H14N2O4	322.315	Dynasore
Selleck Chemicals	S7470	c1cnc(C=NNC(N)=S)c(N)c1	C7H9N5S	195.245	Triapine
Selleck Chemicals	S8061	c1(O)cc2c(cc(C)c(c3c(C)cc4c(cc(O)c(O)c4C(NC[C@@H](c5ccccc5)C)=O)c3O)c2O)c(C(NC[C@@H](c6ccccc6)C)=O)c1O	C42H40N2O8	700.776	Sabutoclax
Selleck Chemicals	S7494	c1(C)cc(C)c(c2nc(NC(=O)c3ccccc3)sc2)c(C)c1	C19H18N2OS	322.424	INH6
Selleck Chemicals	S7513	CN1C(=O)N(Cc2cc(F)ccc2C#N)C(N3CC(N)CCC3)=CC1=O	C18H20FN5O2	357.382	Trelagliptin
Selleck Chemicals	S7526	C(NCCO)(=O)c1cccc(c2cc(Nc3ccc(OC(F)(F)F)cc3)ncn2)c1	C20H17F3N4O3	418.369	GNF-5
Selleck Chemicals	S7587	c1(NC(c2nonc2N)=NO)cc(Cl)c(F)cc1	C9H7ClFN5O2	271.636	INCB024360
Selleck Chemicals	S8002	c1c(C(O)=O)c2c(n(Cc3c(C)c(C(F)(F)F)ccc3)c(C)n2)cc1N4CCOCC4	C22H22F3N3O3	433.424	GSK2636771
Selleck Chemicals	S8014	C(O)(CCc1ccc(NCc2cccc(Oc3ccccc3)c2)cc1)=O	C22H21NO3	347.407	GW9508
Selleck Chemicals	S8024	n1c(c2ncc1c3ccccc3)cc(OC)c(OC)c2	C16H14N2O2	266.295	Tyrphostin AG 1296
Selleck Chemicals	S7094	c(scc1)(c(Nc2n[nH]c3c2CN(C(N[C@@H](c4ccccc4)CN(C)C)=O)C3(C)C)nc(C)n5)c15	C25H30N8OS	490.624	PF-3758309
Selleck Chemicals	S7256	c1cc2c(nc(CCc3ccc(OC)c(Cl)c3)n2C[C@@H](N4CCOCC4)C)cc1c5c(C)onc5C	C28H33ClN4O3	509.04	SGC-CBP30
Selleck Chemicals	S7108	CC(n1nc(c2c(F)c(NS(C)(=O)=O)cc(Cl)c2)c(c3ccnc(NCC(NC(OC)=O)C)n3)c1)C	C22H27ClFN7O4S	540.011	LGX818
Selleck Chemicals	S7270	c1c(C(F)(F)F)cc(NC(=O)c2ccncc2)c(N3CCCCC3)c1	C18H18F3N3O	349.35	SRPIN340
Selleck Chemicals	S7124	c1n2c(c(NNC(c3nccnc3)=O)nc4c2ccc(F)c4)cc1	C16H11FN6O	322.297	SC144
Selleck Chemicals	S7285	c1c(n2c(COc3cc(C)c(c4ccc(S(C)(=O)=O)cc4)cc3)nnc2SC5CCCC5)cncc1	C27H28N4O3S2	520.666	NMS-873
Selleck Chemicals	S7134	c1cc(n2c(C)cc(C(=O)CN3CCCC3)c2C)ccc1F	C18H21FN2O	300.371	IU1
Selleck Chemicals	S7301	C(CCS1)(N=C(SCC(=O)Nc2ccc(c3ccccc3)cn2)N(c4ccccc4)C5=O)=C15	C25H20N4O2S2	472.582	IWP-L6
Selleck Chemicals	S7145	c1cc2c(c(c3ncc(CN4CCOCC4)cc3)c(O)[nH]2)cc1C#N	C19H18N4O2	334.372	AZD1080
Selleck Chemicals	S7320	c1(cc(OC)cc2)c2SC(=CC(=O)C)N1CC	C13H15NO2S	249.329	TG003
Selleck Chemicals	S7157	c1cc2c([nH]cc2C[C@H](NC([C@H](CC(C)C)CC(NO)=O)=O)C(NC)=O)cc1	C20H28N4O4	388.461	Ilomastat (GM6001, Galardin)
Selleck Chemicals	S7333	c1cc(OCC(=O)N2CCC(NC(=O)CCCS)CC2)c(Cl)cc1Cl	C17H22Cl2N2O3S	405.339	K-Ras(G12C) inhibitor 6
Selleck Chemicals	S7177	c1cc(S(=O)(=O)Cc2cc(C)cc(OCc3ccc(CN4CCC[C@@H]4CO)cc3)c2)ccc1	C27H31NO4S	465.604	PF-543
Selleck Chemicals	S7356	c1c(NS(c2ccccc2)(=O)=O)cc(c3ccc4n(c(C(OCC)=O)cn4)c3)cn1	C21H18N4O4S	422.457	HS-173
Selleck Chemicals	S7199	c1cc2c(c(NCc3ccccc3)nc(NCc4ccccc4)n2)cc1	C22H20N4	340.421	DBeQ
Selleck Chemicals	S7369	C1SC(NN=C(C(O)=O)Cc2c(N(O)O)cccc2)=NC1c3ccc(Cl)c(Cl)c3	C18H16Cl2N4O4S	455.315	4EGI-1
Selleck Chemicals	S7217	c1cc(C(=O)Nc2ncc3c(cc[nH]3)c2)ccc1	C14H11N3O	237.257	OAC1

Selleck Chemicals	S7383	OC(=O)C(=O)O.C1SC(=N2)N(C[C@@H]2c3ccc(Br)cc3)C1	C13H13BrN2O4S	373.222	(-)-p-Bromotetramisole Oxalate
Selleck Chemicals	S7241	C1C(NS(c2ccc(c3ccsc3)c(NC(Nc4cc(C(F)(F)F)ccc4)=O)c2)(=O)=O)C1	C21H18F3N3O3S2	481.511	AGI-6780
Selleck Chemicals	S7422	c1(ccnc2)c2cccc1S(=O)(=O)N(C)[C@@H](Cc3ccc(OS(=O)(=O)c4cccc5c4ccnc5)cc3)C(=O)N6CCN(c7ccccc7)CC6	C38H35N5O6S2	721.844	KN-62
Selleck Chemicals	S7437	c1ccc(C=CC(NC(NC(Nc2ccc(Cl)cc2)=S)C(Cl)(Cl)Cl)=O)cc1	C18H15Cl4N3OS	463.208	Sal003
Selleck Chemicals	S8037	c1cc2c([nH]cc2CC3C(=O)N(C)C(=S)N3)cc1	C13H13N3OS	259.327	Necrostatin-1
Selleck Chemicals	S7452	c1cc(OC)c(NC(=S)Nc2cn(CC)nc2C(=O)N)cc1Cl	C14H16ClN5O2S	353.827	FPH2 (BRD-9424)
Selleck Chemicals	S8048	c1cc(C2=C(CN3CCN(c4ccc(C(NS(=O)(=O)c5cc([N+]([O-])=O)c(NCC6CCOCC6)cc5)=O)c(Oc7cc8c([nH]cc8)nc7)c4)CC3)CCC(C)(C)C2)ccc1Cl	C45H50ClN7O7S	868.439	ABT-199 (GDC-0199)
Selleck Chemicals	S7473	CCCCN(C(Nc1ccccc1)=O)Cc2ccc(C(NO)=O)cc2	C19H23N3O3	341.404	Nexturastat A
Selleck Chemicals	S8065	N1(C(=O)N2CCNC(=O)C2)[C@H](c3ccc(Cl)cc3)[C@@H](c4ccc(Cl)cc4)N=C1c5c(OC(C)C)cc(OC)cc5	C30H30Cl2N4O4	581.49	Nutlin-3b
Selleck Chemicals	S7497	Cc1[nH]c(c2c1CCNC(c3sccc3)=O)cccc2	C16H16N2OS	284.376	CK-636
Selleck Chemicals	S7515	C1C(c2ccccc2)=NOC1CC(O)=O	C11H11NO3	205.21	VGX-1027
Selleck Chemicals	S7528	c1(C(F)(F)F)c(NCC)nc(Nc2c(OC)cc(C(=O)N3CCOCC3)c(F)c2)nc1	C19H21F4N5O3	443.395	GNE-7915
Selleck Chemicals	S7589	c1c(n2c(c3ccc(n4ccnc4)cc3)ccc2CCC(=O)O)c(C)cc(C(=O)N)c1	C24H22N4O3	414.456	N6022
Selleck Chemicals	S8003	N(c1cc(Cl)ccc1OC)C(Nc2c(c3nc(C)c2)cccc3)=O	C18H16ClN3O2	341.792	PQ 401
Selleck Chemicals	S8015	N(c1cccc(Oc2c(c3ncn2)cc(OC)c(OC)c3)c1)C(Nc4cc(C(C)(C)C(F)(F)F)on4)=O	C24H22F3N5O5	517.457	CEP-32496
Selleck Chemicals	S8025	c1c(C(F)(F)F)ccc(S(=O)(CCNC(=O)c2ccc(Cl)cc2)=O)n1	C15H12ClF3N2O3S	392.781	GSK3787
Selleck Chemicals	S7096	c1(CCC(Nc2nc3c(cc(Cl)cc3)s2)=O)ccc(OC)c(OC)c1	C18H17ClN2O3S	376.857	KY02111
Selleck Chemicals	S7257	c1ccc(NC(=O)C=C)cc1Nc2nc(Nc3ccc(Oc4cc(C(=O)NC)ncc4)cc3)ncc2F	C26H22FN7O3	499.496	CNX-774
Selleck Chemicals	S7110	n1nc2n(c3c(c(C)c(C)s3)C(c4ccc(Cl)cc4)=N[C@@H]2CC(=O)OC(C)(C)C)c1C	C23H25ClN4O2S	456.988	(+)-JQ1
Selleck Chemicals	S7272	c1(O)c(C=O)c2c(C(C)=CC(=O)O2)cc1	C11H8O4	204.179	4?8C
Selleck Chemicals	S7125	c1(C(F)(F)F)cc(c2nn(C=CC(OC(C)C)=O)cn2)cc(OC)c1	C16H16F3N3O3	355.312	KPT-185
Selleck Chemicals	S7289	c1cc(C(=O)C=Cc2nc3c(cccc3)cc2)ccn1	C17H12N2O	260.29	PFK15
Selleck Chemicals	S7135	c1(Cl)cc2c(N(Cc3c(Cl)ccc(Cl)c3)C(=O)C2=NOC(C)=O)cc1	C17H11Cl3N2O3	397.64	LDN-57444
Selleck Chemicals	S7303	n1c(Nc2c(C)cc(C=CC#N)cc2C)ccnc1Nc3ccc(C#N)cc3	C22H18N6	366.419	Rilpivirine
Selleck Chemicals	S7146	CC(Oc1ccc(c2cnc3n(ncc3c4c(c5ncc4)cccc5)c2)cc1)C	C24H20N4O	380.442	DMH1
Selleck Chemicals	S7324	c1(c2nc(C(F)(F)F)on2)cc(C(=O)NCC3(c4scc(c5ccccc5)n4)CCOCC3)ccc1	C25H21F3N4O3S	514.519	TMP269
Selleck Chemicals	S7158	c1(CN2CCN(CC)CC2)cnc(Nc3ncc(F)c(c4cc5c(nc(C)n5C(C)C)c(F)c4)n3)cc1.CS(O)(=O)=O	C28H36F2N8O3S	602.699	LY2835219
Selleck Chemicals	S7336	c1(I)cc(C(O)=O)c(NC(=O)[C@@H](NCc2ccccc2)Cc3ccccc3)cc1	C23H21IN2O3	500.329	CW069
Selleck Chemicals	S7185	C1CCC(NC(=O)C(c2ccccc2C)N(c3cccc(F)c3)C(=O)Cn4c(C)ncc4)CC1	C27H31FN4O2	462.559	AGI-5198
Selleck Chemicals	S7357	c1ccc(CNc2nc(Nc3cc(CC(=O)N4)c4cc3)ncc2C(F)(F)F)c(N(S(C)(=O)=O)C)n1.Cl	C21H21ClF3N7O3S	543.95	PF-562271 HCl
Selleck Chemicals	S7206	c1(C(F)(F)F)c(Nc2cc(NC(=O)C=C)ccc2)nc(Nc3ccc(NC4CN(CCF)C4)cc3OC)nc1	C26H27F4N7O2	545.532	CNX-2006
Selleck Chemicals	S7370	c1ccc(C(N(c2ccc(C(O)=O)cc2)C(=O)C3=Cc4oc(c5ccc([N+]([O-])=O)cc5)cc4)=C3)cc1	C28H18N2O6	478.452	4E1RCat
Selleck Chemicals	S7218	C(N(c1cc(C(F)(F)F)ccc1)C(=O)C(C(=O)NCc2ccc(S(=O)(=O)C)cn2)=C3)=C3c4n(C)ncc4	C24H20F3N5O4S	531.507	Alvelestat (AZD9668)

Selleck Chemicals	S7391	[C@@H](NC(=O)OCc1ccccc1)(C(=O)NC(C)C(=O)CF)Cc2ccccc2	C21H23FN2O4	386.417	Z-FA-FMK
Selleck Chemicals	S7242	c1c2c(C(=O)N(c3ccccc3OCC)C(C(N4CCN(C(=O)COc5ccc(Cl)cc5)CC4)C)=N2)ccc1	C30H31ClN4O4	547.045	Erastin
Selleck Chemicals	S7423	c1(ccccc1CN(C)CC=Cc2ccc(Cl)cc2)N(CCO)S(=O)(=O)c3ccc(OC)cc3.P(O O)(=O)=O.[H]	C26H32ClN2O8PS	599.033	KN-93 Phosphate
Selleck Chemicals	S7438	CC(NC(CCC(Nc(c1C2=O)cccc1)=N2)=O)c3ccccc3	C19H19N3O2	321.373	ME0328
Selleck Chemicals	S8038	c1cc2c(C=CNC2=O)c(OCC(=O)c3ccccc3)c1	C17H13NO3	279.29	UPF 1069
Selleck Chemicals	S7458	CCNC(C(NNC1=C2C(=O)C=C(O)C(Cl)=C2)=C1c3ccc(OC)cc3)=O	C19H18ClN3O4	387.817	VER-49009
Selleck Chemicals	S8050	c1c2c(c3c(COC(=O)N3c4ccc(C(C)(C)C#N)cc4)cn2)cc(c5cc6c(cccc6)nc5)c1	C30H22N4O2	470.521	ETP-46464
Selleck Chemicals	S7482	O1C=C(OCCCCCSc2c3c(cc(C(F)(F)F)cc3)ncc2)C(=O)C=C1CN4CCOCC4.Cl.Cl	C25H29Cl2F3N2O4S	581.475	EHT 1864
Selleck Chemicals	S8072	c1cc(C(=O)NC(C)CCC)cc(Cl)c1Cl	C12H15Cl2NO	260.16	NSC 405020
Selleck Chemicals	S7499	c1c(NN=C(C#N)C(=O)c2noc(C(C)(C)C)c2)cccc1Cl	C16H15ClN4O2	330.769	ESI-09
Selleck Chemicals	S7516	Cc1n(c2ccc(Cl)cc2)ncc1C(Nc3ccc(N4CCC(N5CCOCC5)CC4)c(C#N)c3)=O	C27H29ClN6O2	505.011	Y-320
Selleck Chemicals	S7529	c1(c2ncc(C)c(NCc3ccc(n4ccnn4)cc3)n2)ccccc1C(C)C	C23H24N6	384.477	ML323
Selleck Chemicals	S7595	c1ccccc1CCNC(=O)CCCNC(=O)OCC	C15H22N2O3	278.347	Santacruzamate A (CAY10683)
Selleck Chemicals	S8004	C(c1ccc(c2c1)cccc2)(=O)CCN(Cc3ccccc3)C(C)C.Cl	C23H26ClNO	367.912	ZM 39923 HCl
Selleck Chemicals	S8016	N(C)Cc1cc(c2ccccc2F)n(S(c3cccnc3)(=O)=O)c1.C(O)(=O)C=CC(O)=O	C21H20FN3O6S	461.463	TAK-438
Selleck Chemicals	S8028	c1cc2c(ncc(C(=O)Nc3cc(OC)c(OC)cc3C(=O)Nc4ccc(CCN5CCc6c(cc(OC)c(OC)c6)C5)cc4)c2)cc1	C38H38N4O6	646.732	Tariquidar
Selleck Chemicals	S7097	n1c(c2cc(F)ccc2[C@H]3Cc4c(c(C)nc(N)n4)C(=O)N3)cccc1OC	C20H18FN5O2	379.388	HSP990 (NVP-HSP990)
Selleck Chemicals	S7258	Cc1sc(Sc2c(c3ncn2)cc(OC)c(OC)c3)nn1	C13H12N4O2S2	320.39	SKLB1002
Selleck Chemicals	S7111	c1ccc2c(c3n(cnc3)C2CC(O)C4CCCCC4)c1	C18H22N2O	282.38	NLG919
Selleck Chemicals	S7273	n1c(N2CCC(c3nc(C(=O)Nc4[nH]c5c(ccc(C(=O)c6ccccc6)c5)n4)cs3)CC2)c7c(ccs7)nc1	C29H23N7O2S2	565.669	SC75741
Selleck Chemicals	S7127	c1cc(CN2CC3=C(N(Cc4ccccc4C)C(=N5)N(CC5)C3=O)CC2)ccc1	C24H26N4O	386.489	TIC10
Selleck Chemicals	S7291	c(nc(NC(=O)C1CC1)s2)(ccc(Oc3ccc(F)c(NC(=O)Cc4cc(C(F)(F)F)ccc4)c3)c5C#N)c25	C27H18F4N4O3S	554.515	TAK-632
Selleck Chemicals	S7136	c1cc(NC(=S)NC(NC(=O)C(c2ccccc2)c3ccccc3)C(Cl)(Cl)Cl)cc([N+]([O-])=O)c1F	C23H18Cl3FN4O3S	555.836	CGK 733
Selleck Chemicals	S7304	c1(C)sc2c(C(c3ccc(Cl)cc3)=N[C@@H](CC(N)=O)c4n2c(C)nn4)c1C	C19H18ClN5OS	399.897	CPI-203
Selleck Chemicals	S7147	C1NCCN(c2ccc(c3cnc4n(ncc4c5cccc(c56)nccc6)c3)cc2)C1	C25H22N6	406.482	LDN-212854
Selleck Chemicals	S7325	n1c(N[C@H]2CC[C@H](O)CC2)c(C(=O)NCc3ccc(n4ccnc4)cc3)cnc1NCCCC	C25H33N7O2	463.575	UNC2881
Selleck Chemicals	S7162	N(c(c1C(=O)N2c3cc(OC)c(Cl)cc3Cl)cccc1)=C2S	C15H10Cl2N2O2S	353.223	Mdivi-1
Selleck Chemicals	S7337	C1CC(c2ccc(CN(c3ccc(C(=O)O)cc3)C(CN(C)S(=O)(=O)c4c(F)c(F)c(F)c(F)c4F)=O)cc2)CCC1	C29H27F5N2O5S	610.592	SH-4-54
Selleck Chemicals	S7189	n(c(C)nn1)(c2c(cc(OC)cc2)C(c3ccc(Cl)cc3)=N[C@H]4CC(=O)NCC)c14	C22H22ClN5O2	423.895	I-BET-762
Selleck Chemicals	S7359	n1c(N)c(c2cc(OC)c(OC)c(OC)c2)cc(c3cc(O)ccc3)c1	C20H20N2O4	352.384	K02288
Selleck Chemicals	S7207	c1cccc2c1n(CCCSC(N)=N)cc2C3=C(c4c(c5n(C)c4)cccc5)C(=O)NC3=O.CS(O)(=O)=O	C26H27N5O5S2	553.653	Ro 31-8220 Mesylate
Selleck Chemicals	S7372	Cc1nc(n2c1c3nc(Nc4c(Br)cc(OC)cc4Br)sc3)nccc2	C17H13Br2N5OS	495.191	PTC-209
Selleck Chemicals	S7223	n1c(c2ccc1c3c[nH]nc3c4nc(C)ccc4)cccn2	C17H13N5	287.319	RepSox
Selleck Chemicals	S7392	CC(CCNC(C(NC(C1OC1C(O)=O)=O)CC(C)C)=O)C	C15H26N2O5	314.377	Loxistatin Acid (E-64C)

Selleck Chemicals	S7243	c1(NC2CCCCC2)c(N)cc(C(=O)OCC)cc1	C15H22N2O2	262.347	Ferrostatin-1 (Fer-1)
Selleck Chemicals	S7424	c1cc2c(c(C=C(C(=O)O)S)c[nH]2)cc1F	C11H8FNO2S	237.25	PD 151746
Selleck Chemicals	S7440	CN(C(c1n(C2CCCC2)c(c3c1)nc(Nc4ccc(N5CCNCC5)cn4)nc3)=O)C	C23H30N8O	434.537	LEE011
Selleck Chemicals	S8039	n1c(N)c2c(n(CCCNC(C)C)c(Sc3c(I)cc4c(OCO4)c3)n2)nc1	C18H21IN6O2S	512.368	PU-H71
Selleck Chemicals	S7459	n1oc(c2c(O)cc(O)c(Cl)c2)c(c3ccc(OC)cc3)c1C(=O)NCC	C19H17ClN2O5	388.802	VER-50589
Selleck Chemicals	S8051	c1(Br)cnc(OCCOc2ncnc(NS(=O)(NCCC)=O)c2c3ccc(Br)cc3)nc1	C19H20Br2N6O4S	588.273	Macitentan
Selleck Chemicals	S7484	c1c(Cl)cc(S(=O)(=O)Nc2ccc([N+]([O-])=O)cc2C)c(Cl)c1	C13H10Cl2N2O4S	361.2	FH535
Selleck Chemicals	S8073	c1ncc(n2c(C)cc(C=C3C(=O)NC(=S)S3)c2C)cc1	C15H13N3OS2	315.413	Optovin
Selleck Chemicals	S7500	c1c(C)cc(C)c(S(n2c(C)cc(C)c2)(=O)=O)c1C	C15H19NO2S	277.382	HJC0350
Selleck Chemicals	S7517	CC(O)(C(F)(F)F)C(Nc1ccc(S(c2ccc(C(N(C)C)=O)cc2)(=O)=O)cc1Cl)=O	C19H18ClF3N2O5S	478.87	AZD7545
Selleck Chemicals	S7534	c1(ccccc1OCCOc2ccccc2N(CC(=O)OCOC(=O)C)CC(=O)OCOC(=O)C)N(CC(=O)OCOC(=O)C)CC(=O)OCOC(=O)C	C34H40N2O18	764.684	BAPTA-AM
Selleck Chemicals	S7597	CN[C@@H](C)C(=O)N[C@@H](C1CCCCC1)C(=O)N2CCC[C@H]2C(N[C@@H](C(c3ccccc3)c4ccccc4)C(=O)NCCCCCCNC([C@H](C(c5ccccc5)c6ccccc6)NC(=O)[C@@H]7CCCN7C(=O)[C@H](C8CCCCC8)NC(=O)[C@H](C)NC)=O)=O	C70H96N10O8	1205.573	BV-6
Selleck Chemicals	S8005	S1C(=Cc2cccc(C(F)(F)F)c2)C(=O)NC1=O	C11H6F3NO2S	273.231	SMI-4a
Selleck Chemicals	S8019	N1(c2ncnc3c2cc[nH]3)CCC(N)(C(N[C@H](c4ccc(Cl)cc4)CCO)=O)CC1	C21H25ClN6O2	428.915	AZD5363
Selleck Chemicals	S8029	c1(cc(Cl)nc(SCC(=O)O)n1)Nc2cccc(C)c2C	C14H14ClN3O2S	323.798	WY-14643 (Pirinixic Acid)
Selleck Chemicals	S7098	N1(C(C(c2ccccc2)c3ccccc3)=O)Cc4c(n(Cc5ccc(N(C)C)c(C)c5)cn4)C[C@H]1C(=O)O	C31H32N4O3	508.611	PD123319
Selleck Chemicals	S7261	c1cc2c(C(=O)C(=O)C3=C2OC(C)(C)CC3)cc1	C15H14O3	242.27	Beta-Lapachone
Selleck Chemicals	S7113	[C@@H]1(CO)[C@H](O)[C@H](O)[C@H](N2C=CC=NC2=O)O1	C9H12N2O5	228.202	Zebularine
Selleck Chemicals	S7276	n1c2c(cccc2)c(Nc3ccc(C(=O)Nc4ccc(Nc5cc(C)nc(N)n5)cc4)cc3)cc1	C27H23N7O	461.518	SGI-1027
Selleck Chemicals	S7128	C1OCCC(N(c2cc(c3ccc(CN4CCOCC4)cc3)cc(C(=O)NCC5=C(C)C=C(C)NC5=O)c2C)CC)C1	C34H44N4O4	572.738	EPZ-6438
Selleck Chemicals	S7292	c1(C)ccc(C(NCCCCCC(=O)Nc2c(N)cccc2)=O)cc1	C20H25N3O2	339.431	RG2833 (RGFP109)
Selleck Chemicals	S7138	c1cc2c(c(c3ccccc3)nc(Nc4ccc(C(=O)Nc5cc(CNC)ccc5C)cc4)n2)cc1	C30H27N5O	473.568	BMS-833923
Selleck Chemicals	S7307	c1cc2c(CCN2C(Cc3cc(C(F)(F)F)ccc3)=O)cc1c4cn(C)c5c4c(N)ncn5	C24H20F3N5O	451.444	GSK2606414
Selleck Chemicals	S7148	COc1ccc(c2cnc3n(ncc3c4cccc(c45)nccc5)c2)cc1	C22H16N4O	352.389	ML347
Selleck Chemicals	S7326	C(NS(=O)(c1ccc(Br)s1)=O)(=O)c2ccc(Cl)cc2Cl	C11H6BrCl2NO3S2	415.11	Tasisulam
Selleck Chemicals	S7165	c1cc(c2cc(C(NCC(C(=O)NC(C)=C3)=C3CCC)=O)c4c(n(C(C)C)nc4)c2)cnc1N5CCN(C(C)C)CC5	C33H43N7O2	569.74	UNC1999
Selleck Chemicals	S7338	c1cc2c(n(C)cc2c3ccnc(Nc4c(OC)cc(N5CCN(C)CC5)cc4)n3)cn1	C24H27N7O	429.517	AZ191
Selleck Chemicals	S7192	n1c(N)c2c(OCCN(c3ccc([C@H]4CC[C@H](CC(=O)O)CC4)cc3)C2=O)nc1	C21H24N4O4	396.44	PF-04620110
Selleck Chemicals	S7360	Cc1c2c(n(c3C(CC(Nc4ccc(O)cc4)=O)N=C2c5ccc(Cl)cc5)c(C)nn3)sc1C	C25H22ClN5O2S	491.992	OTX015
Selleck Chemicals	S7208	c1cccc2c1n(CCCN(C)C)cc2C3=C(c4c(c5[nH]c4)cccc5)C(=O)NC3=O	C25H24N4O2	412.484	GF109203X
Selleck Chemicals	S7373	C1CN(C2CCN(C(c3cncc(Br)c3)=O)CC2)CC1	C15H20BrN3O	338.243	UNC669
Selleck Chemicals	S7224	c1cc2c(n([C@H](COc3ccc(c4nc5c(cccc5)n4Cc6ccccc6)cc3)C7CCNCC7)c(c8ccccc8)n2)cc1	C40H37N5O	603.755	Deltarasin
Selleck Chemicals	S7393	CCOC(C1OC1C(NC(C(NCC(C)C)=O)CC(C)C)=O)=O	C17H30N2O5	342.43	Aloxistatin

Selleck Chemicals	S7248	c1(C(=O)NC2CCN(C)CC2)ccc(Nc3ncc4c(N(C5CCCC5)CC(F)(F)C(=O)N4C)n3)c(OC)c1	C27H35F2N7O3	543.609	Ro3280
Selleck Chemicals	S7429	n1n(c2ccc(NC(CCl)=O)cc2)c(c3ccc(Cl)c(Cl)c3)nc1OCCOC	C19H17Cl3N4O3	455.722	MI-2 (MALT1 inhibitor)
Selleck Chemicals	S7447	c1cc2c([nH]c(c3ccc(C(C)(C)C)cc3)n2)cc1	C17H18N2	250.338	ZLN005
Selleck Chemicals	S8040	N(CC)C(Nc1ccc(c2nc(N3CCOC[C@@H]3C)c4c(CN(C5COC5)CC4)n2)cc1)=O	C24H32N6O3	452.549	GDC-0349
Selleck Chemicals	S7460	c1ccc(S(c2ccc([N+]([O-])=O)c(OC(c3ccc(Cl)cc3)=O)c2)(=O)=O)cc1	C19H12ClNO6S	417.82	BTB06584
Selleck Chemicals	S8056	n1c(N)nc(OCc2scc(Br)c2)c3c1nc[nH]3	C10H8BrN5OS	326.172	Lomeguatrib
Selleck Chemicals	S7488	Cc1cc(N2CCOCC2)ccc1Nc3nc(NC4CCCCC4)c5c(nc[nH]5)n3	C22H29N7O	407.512	MPI-0479605
Selleck Chemicals	S8076	c1nccc(C(NNc2ccccc2)=O)c1	C12H11N3O	213.235	PluriSIn #1 (NSC 14613)
Selleck Chemicals	S7501	C1(=Cc2ccc(F)cc2)C(=O)C(=Cc3ccc(F)cc3)CN(CC4=CC(C)(C)N(O[H])C4(C)C)C1	C28H30F2N2O2	464.547	HO-3867
Selleck Chemicals	S7518	c(N(c1nccs1)C=C(C(O)=O)C2=O)(nc(N3C[C@H](OC)[C@@H](NC)C3)cc4)c24	C18H19N5O4S	401.44	Voreloxin (SNS-595)
Selleck Chemicals	S7539	c1(OC)cc(Br)c(Nc2scc(c3c(C)nc4n3cccn4)n2)c(Br)c1.Br	C17H14Br3N5OS	576.103	PTC-209 HBr
Selleck Chemicals	S7605	c1c(c2ccc(CN3CCS(=O)(=O)CC3)cc2)n4c(nc(NC(=O)C5CC5)n4)cc1	C21H23N5O3S	425.504	Filgotinib (GLPG0634)
Selleck Chemicals	S8006	n1c(c2c(NC3CCN(Cc4ccccc4)CC3)nc1N5CCCN(C)CC5)cc(OC)c(OC)c2.Cl.Cl.Cl	C28H41Cl3N6O2	600.023	BIX 01294
Selleck Chemicals	S8020	n1c(C)c(CSc2ccc(OCC(O)=O)c(C)c2)sc1c3ccc(C(F)(F)F)c(F)c3	C21H17F4NO3S2	471.488	GW0742
Selleck Chemicals	S8031	n1c(c2c(N)cc1C)ccc(Nc3cc(C)nc(NC(C)CCCN(CC)CC)n3)c2.Cl.Cl.Cl	C24H38Cl3N7	530.964	NSC 23766
Selleck Chemicals	S1001	c1c(C(CCC(C)(C)C2)=C2CN3CCN(c4ccc(C(=O)NS(=O)(=O)c5ccc(N[C@@H](CSc6ccccc6)CCN7CCOCC7)c(S(=O)(=O)C(F)(F)F)c5)cc4)CC3)ccc(Cl)c	C47H55ClF3N5O6S3	974.613	ABT-263 (Navitoclax)
Selleck Chemicals	S1107	c1([C@@H](OC)C(=O)N2Cc3c([nH]nc3NC(c4ccc(N5CCN(C)CC5)cc4)=O)C2)ccccc1	C26H30N6O3	474.555	Danusertib (PHA-739358)
Selleck Chemicals	S1010	c1cc2c(NC(=O)C2=C(Nc3ccc(N(C(=O)CN4CCN(C)CC4)C)cc3)c5ccccc5)cc1C(=O)OC	C31H33N5O4	539.625	Nintedanib (BIBF 1120)
Selleck Chemicals	S1116	n1cc2c(N(C3CCCC3)C(=O)C(C(=O)C)=C2C)nc1Nc4ccc(N5CCNCC5)cn4.Cl	C24H30ClN7O2	483.994	Palbociclib (PD-0332991) HCl
Selleck Chemicals	S1025	c1(OC)cc2c(c(Nc3cc(Cl)c(F)cc3)ncn2)cc1OCCCN4CCOCC4	C22H24ClFN4O3	446.902	Gefitinib (ZD1839)
Selleck Chemicals	S1130	c1cc2c(C(=O)C3=C([N](Cc4cnccn4)=C(C)N3CCOC)C2=O)cc1.[Br-]	C20H19BrN4O3	443.294	YM155 (Sepantronium Bromide)
Selleck Chemicals	S1035	c1cc(Nc2nc(N(C)c3cc4c(c(C)n(C)n4)cc3)ccn2)cc(S(=O)(N)=O)c1C.Cl	C21H24ClN7O2S	473.979	Pazopanib HCl (GW786034 HCl)
Selleck Chemicals	S1142	C(NCCN(C)C)(C1=O)=C(C[C@@H](C)C[C@H](OC)[C@H](O)[C@@H](C)C=C(C)[C@H](OC(N)=O)[C@@H](OC)C=CC=C(C)C(=O)N2)C(=O)C2=C1	C32H49ClN4O8	653.206	17-DMAG (Alvespimycin) HCl
Selleck Chemicals	S1044	[C@H]1(OC)[C@@H](OC(=O)C(C)(CO)CO)CC[C@@H](C[C@@H](C)[C@@H]2OC(=O)[C@@]3([H])N(CCCC3)C(=O)C(=O)[C@](O)(O[C@@]4([H])C[C@H](OC)C(C)=CC=CC=C[C@@H](C)C[C@@H](C)C(=O)[C@H](OC)[C@H](O)C(C)=C[C@@H](C)C(=O)C2)[C@H](C)CC4)C1	C56H87NO16	1030.287	Temsirolimus (CCI-779, NSC 683864)
Selleck Chemicals	S1154	c(scc1)(c(NCCc2cnc(NC(=O)Nc3cccc(Cl)c3)s2)ncn4)c14.CS(O)(=O)=O	C19H19ClN6O4S3	527.04	SNS-314 Mesylate
Selleck Chemicals	S1055	c1cc2c(n(C3CCN(Cc4ccccn4)CC3)cc2C5=C(c6cn(C)c7c6cccc7)C(=O)NC5=O)cc1	C32H29N5O2	515.605	Enzastaurin (LY317615)
Selleck Chemicals	S1164	c1(C(=O)N)cc2c(nccc2Oc3ccc(NC(NC4CC4)=O)c(Cl)c3)cc1OC	C21H19ClN4O4	426.853	Lenvatinib (E7080)
Selleck Chemicals	S1068	c1(c2cnn(C3CCNCC3)c2)cnc(N)c(O[C@H](C)c4c(Cl)c(F)ccc4Cl)c1	C21H22Cl2FN5O	450.337	Crizotinib (PF-02341066)
Selleck Chemicals	S1172	c1cccc2c1c(CCNc3ccc(Nc4ccncc4)cc3)c[nH]2	C21H20N4	328.41	JNJ-26854165 (Serdemetan)
Selleck Chemicals	S1078	c1(c2cc3c(C=CN4C3=NNC4=O)nc2c5ccc(C6(N)CCC6)cc5)ccccc1.Cl.Cl	C25H23Cl2N5O	480.389	MK-2206 2HCl
Selleck Chemicals	S1181	c1(C=Cc2ccccc2)nc(N3CCN(C)CC3)cc(Nc4cc(C)[nH]n4)n1	C21H25N7	375.47	ENMD-2076
Selleck Chemicals	S1090	c1cc2c(c(CN(C)C)c(C(NCCOc3ccc(C(NO)=O)cc3)=O)o2)cc1	C21H23N3O5	397.424	PCI-24781 (Abexinostat)
Selleck Chemicals	S1193	C1(=O)c(c2C(=O)N1C3CCC(=O)NC3=O)cccc2	C13H10N2O4	258.23	Thalidomide

Selleck Chemicals	S1098	c1([nH]c2c(c3cc(F)c2)c1CCNC3=O)c4ccc(CNC)cc4.P(O)(O)(O)=O	C19H21FN3O5P	421.359	Rucaparib (AG-014699,PF-01367338)
Selleck Chemicals	S1204	c1c2c(c(CCNC(C)=O)c[nH]2)cc(OC)c1	C13H16N2O2	232.278	Melatonin
Selleck Chemicals	S1213	n1c(OC)c2c(n([C@@H]3O[C@H](CO)[C@@H](O)[C@@H]3O)cn2)nc1N	C11H15N5O5	297.267	Nelarabine
Selleck Chemicals	S1393	[C@@H]1(C)O[C@@H](O[C@@]2([H])C[C@](O)(C(=O)CO)Cc3c2c(O)c4c(C(=O)c5c(c(OC)ccc5)C4=O)c3O)C[C@H](N)[C@@H]1O[C@@H]6CCCCO6	C32H37NO12	627.636	Pirarubicin
Selleck Chemicals	S1226	n1c(N2CCOCC2)c3c(nc(c4ccc(OC)c(CO)c4)cc3)nc1N5C[C@H](C)O[C@H](C)C5	C25H31N5O4	465.545	KU-0063794
Selleck Chemicals	S1407	c1ccc(CCC(C=C[C@H]2[C@H](O)C[C@H](O)[C@@H]2CC=CCCCC(NCC)=O)O)cc1	C25H37NO4	415.566	Bimatoprost
Selleck Chemicals	S1243	C(NCCc1c(c2ccc1)cc(OC)cc2)(=O)C	C15H17NO2	243.301	Agomelatine
Selleck Chemicals	S1431	c1(S(=O)(=O)N2CCN(C)CC2)cc(C(NC(=O)c3c4c(CCC)nn3C)=N4)c(OCC)cc1.C(C(C(=O)O)(O)CC(=O)O)C(=O)O	C28H38N6O11S	666.7	Sildenafil Citrate
Selleck Chemicals	S1257	c1(N2C=NN([C@@H](CC)[C@@H](O)C)C2=O)ccc(N3CCN(c4ccc(OC[C@H]5C[C@](Cn6cncn6)(c7c(F)cc(F)cc7)OC5)cc4)CC3)cc1	C37H42F2N8O4	700.777	Posaconazole
Selleck Chemicals	S1453	c1c([C@@](N)(c2ccc3c(C(c4cccc(Cl)c4)=CC(=O)N3C)c2)c5n(C)cnc5)ccc(Cl)c1	C27H22Cl2N4O	489.396	Tipifarnib
Selleck Chemicals	S1271	[C@@H]1(N[C@@H]2[C@@H](O)[C@@H](O)[C@H](O)C(CO)=C2)[C@H](C)O[C@H](O[C@@H]3[C@H](CO)O[C@H](O[C@@H]4[C@H](CO)O[C@@H](O)[C@H](O)[C@H]4O)[C@H](O)[C@H]3O)[C@H](O)[C@H]1O	C25H43NO18	645.605	Acarbose
Selleck Chemicals	S1462	C(O)(=O)c1ccccc1N[C@@H](C(C(N2C=C3C)=NC(N4CCOCC4)=CC2=O)=C3)C	C22H24N4O4	408.45	AZD6482
Selleck Chemicals	S1287	c1c(C(O)=O)cc(NCCCC)c(Oc2ccccc2)c1S(N)(=O)=O	C17H20N2O5S	364.416	Bumetanide
Selleck Chemicals	S1476	c1(c2c(c3nc(C)ccc3)nc(C(C)(C)C)[nH]2)cc4c(nccn4)cc1	C21H21N5	343.425	SB525334
Selleck Chemicals	S1302	C1CN(CCCl)P(=O)(NCCCl)OC1	C7H15Cl2N2O2P	261.086	Ifosfamide
Selleck Chemicals	S1487	[nH]1c2c(CN(C(=O)C3CCN(C)CC3)C2(C)C)c(NC(=O)CC(C)C)n1	C19H31N5O2	361.482	PHA-793887
Selleck Chemicals	S1336	c1(C(F)(F)F)ccc(C(=NOCCN)CCCCOC)cc1.C(=CC(O)=O)C(O)=O	C19H25F3N2O6	434.407	Fluvoxamine maleate
Selleck Chemicals	S1502	c1ccc([C@@H](N)C(=O)N[C@H]2[C@@]3([H])N(C(C(O)=O)=C(C)CS3)C2=O)cc1	C16H17N3O4S	347.389	Cephalexin
Selleck Chemicals	S1363	c1c(Oc2cc(F)c(NC(=O)Nc3c(F)cc(F)cc3)cc2)c4c(cc(OC)c(OC)c4)nc1	C24H18F3N3O4	469.413	Ki8751
Selleck Chemicals	S1519	c1(Cl)c(N2CCN(CC(=O)Nc3nccs3)CC2)c4c([nH]c(c5ccc(N(C)C)cc5)n4)nc1	C23H25ClN8OS	497.016	CCT129202
Selleck Chemicals	S1379	C(O)(=O)C=C(C)C=CC=C(C)C=CC(C(C)(C)CCC1)=C1C	C20H28O2	300.435	Isotretinoin
Selleck Chemicals	S1531	c1cc2c(NC(=O)C2=C(c3ccccc3)Nc4cccc(CN(C)C)c4)cc1C(=O)N(C)C	C27H28N4O2	440.537	BIX 02189
Selleck Chemicals	S1002	c1c(C(NS(=O)(=O)c2cc([N+]([O-])=O)c(N[C@H](CCN(C)C)CSc3ccccc3)cc2)=O)ccc(N4CCN(Cc5ccccc5c6ccc(Cl)cc6)CC4)c1	C42H45ClN6O5S2	813.427	ABT-737
Selleck Chemicals	S1109	c1(C(=O)NC2CCN(C)CC2)cc(OC)c(Nc3nc4c(N(C)C(=O)[C@@H](CC)N4C5CCCC5)cn3)cc1	C28H39N7O3	521.654	BI 2536
Selleck Chemicals	S1012	c1(N2CCOCC2)cc3c(nc(C(C(=O)NC=C4)=C4NC[C@@H](O)c5cc(Cl)ccc5)[nH]3)c(C)c1	C25H26ClN5O3	479.959	BMS-536924
Selleck Chemicals	S1117	[C@H]1(O)[C@@H](n2cc3c(c4N(C)N=C3N)c2ncn4)O[C@H](CO)[C@H]1O	C13H16N6O4	320.304	Triciribine
Selleck Chemicals	S1026	c1(NC(c2ccc(CN3CCN(C)CC3)cc2)=O)cc(Nc4nc(c5cnccc5)ccn4)c(C)cc1.OS(=O)(=O)C	C30H35N7O4S	589.708	Imatinib Mesylate (STI571)
Selleck Chemicals	S1132	c1c(C(N)=O)cc(N)cc1	C7H8N2O	136.151	INO-1001
Selleck Chemicals	S1036	c1c(C(NOC[C@@H](CO)O)=O)c(Nc2c(F)cc(I)cc2)c(F)c(F)c1	C16H14F3IN2O4	482.193	PD0325901
Selleck Chemicals	S1143	c1(O)cc(C=C(C#N)C(=O)NCc2ccccc2)ccc1O	C17H14N2O3	294.305	AG-490 (Tyrphostin B42)
Selleck Chemicals	S1045	c1cc(C(=O)[C@H](C)C=C(C)C=CC(=O)NO)ccc1N(C)C	C17H22N2O3	302.368	Trichostatin A (TSA)
Selleck Chemicals	S1155	c1(C)ccc(S(=O)(=O)OCC(=O)Nc2cc(O)c(C(=O)O)cc2)cc1	C16H15NO7S	365.358	S3I-201

Selleck Chemicals	S1056	C1OC[C@@H](COC(=O)Nc2c(C)c3n(ncnc3Nc4ccc5c(cnn5Cc6cccc(F)c6)c4)c2)NC1.Cl	C27H28ClFN8O3	567.014	AC480 (BMS-599626)
Selleck Chemicals	S1165	c1cc(S(Nc2cccnc2Nc3ccc(O)cc3)(=O)=O)ccc1OC	C18H17N3O4S	371.41	ABT-751 (E7010)
Selleck Chemicals	S1069	c1(O)c(c2onc(C(NCC)=O)c2c3ccc(CN4CCOCC4)cc3)cc(C(C)C)c(O)c1	C26H31N3O5	465.541	AUY922 (NVP-AUY922)
Selleck Chemicals	S1173	c1(N2CCN(C)CC2)ccc(Nc3nc(Oc4cccc(NC(=O)C=C)c4)c(Cl)cn3)c(OC)c1	C25H27ClN6O3	494.973	WZ4002
Selleck Chemicals	S1080	c1(Cl)cc(N(S(=O)(=O)c2ccc3c(C(=Cc4c(C)c(C(=O)N5CCN(C)CC5)c(C)[nH]4)C(=O)N3)c2)C)ccc1	C28H30ClN5O4S	568.087	SU11274
Selleck Chemicals	S1183	[C@@H]1(OC(=O)N2Cc3c(cccc3F)C2)C[C@@H]4N(C(=O)[C@@H](NC(=O)OC(C)(C)C)CCCCCC=C[C@H]5[C@](C5)(C(=O)NS(C6CC6)(=O)=O)NC4=O)C1	C35H46FN5O9S	731.831	Danoprevir (ITMN-191)
Selleck Chemicals	S1091	c1cc2c(nc(c3ccccc3)cc2)cc1c4nc([C@@H]5C[C@@](O)(C)C5)n6c4c(N)ncc6	C26H23N5O	421.494	OSI-906 (Linsitinib)
Selleck Chemicals	S1194	c(cc(OCCCCCCC(=O)NO)c(OC)c1)(c(Nc2cccc(C#C)c2)ncn3)c13	C24H26N4O4	434.488	CUDC-101
Selleck Chemicals	S1100	c1cc2c(C(c3c(F)cccc3F)=NCc4c2nc(Nc5ccc(C(O)=O)cc5)nc4)cc1Cl	C25H15ClF2N4O2	476.862	MLN8054
Selleck Chemicals	S1205	c1cc2n(c(C=NN(C)S(=O)(=O)c3cc([N+]([O-])=O)ccc3C)cn2)cc1Br.Cl	C16H15BrClN5O4S	488.743	PIK-75
Selleck Chemicals	S1218	n1c(N)c2c(n([C@@H]3O[C@H](CO)[C@@H](O)[C@@H]3F)cn2)nc1Cl	C10H11ClFN5O3	303.677	Clofarabine
Selleck Chemicals	S1396	c1(O)ccc(C=Cc2cc(O)cc(O)c2)cc1	C14H12O3	228.243	Resveratrol
Selleck Chemicals	S1227	c1(O)cc2c(c(C(=O)c3ccc(OCCN4CCCCC4)cc3)c(c5ccc(O)cc5)s2)cc1.Cl	C28H28ClNO4S	510.044	Raloxifene HCl
Selleck Chemicals	S1408	c1cc(N2C(=O)O[C@@H](CNC(=O)C)C2)cc(F)c1N3CCOCC3	C16H20FN3O4	337.346	Linezolid
Selleck Chemicals	S1244	c1cc2c(oc3c2ncnc3N4CCN(C(NCc5cc6c(OCO6)cc5)=S)CC4)cc1	C23H21N5O3S	447.51	Amuvatinib (MP-470)
Selleck Chemicals	S1437	c1(Cl)c(NC2=NCCN2)c3c(nsn3)cc1.Cl	C9H9Cl2N5S	290.172	Tizanidine HCl
Selleck Chemicals	S1258	C(=O)(C1CC1)C(N2Cc3c(sc(OC(C)=O)c3)CC2)c4ccccc4F	C20H20FNO3S	373.441	Prasugrel
Selleck Chemicals	S1454	c1(N2CCN(C)CC2)ccc(C(Nc3n[nH]c4c3CN(C(Nc5c(CC)cccc5CC)=O)C4)=O)cc1	C28H35N7O2	501.623	PHA-680632
Selleck Chemicals	S1275	N1(C(Nc2cccc(Nc3ncc(Br)c(NCCc4[nH]cnc4)n3)c2)=O)CCCC1	C20H23BrN8O	471.354	BX-912
Selleck Chemicals	S1465	c1(F)cc2c(N(C3CC3)C=C(C(=O)O)C2=O)c(OC)c1N4C[C@@]5([H])[C@]([H])(NCCC5)C4.Cl	C21H25ClFN3O4	437.892	Moxifloxacin HCl
Selleck Chemicals	S1288	O1CC2=C(C=C3N(Cc4c3nc5c(cccc5)c4)C2=O)[C@@](O)(CC)C1=O	C20H16N2O4	348.352	Camptothecin
Selleck Chemicals	S1478	C1[C@@H](C)[C@H](C[C@H](O)C)O[C@]2(O[C@@H]3[C@@]([H])(C)[C@@H](OC(=O)C=C[C@H](C)[C@@H](O)[C@H](C)C(=O)[C@H](C)[C@](O)([H])[C@H](C)C(=O)[C@@](O)(C)[C@@H](O)[C@H](C)CC=CC=C[C@H](CC)CC3)[C@@H]2C)C1	C45H74O11	791.062	Oligomycin A
Selleck Chemicals	S1304	C1C[C@@]2(C)C(C(C)=C[C@]3([H])[C@@]2([H])CC[C@@]4(C)[C@@]3([H])CC[C@@]4(OC(C)=O)C(=O)C)=CC1=O	C24H32O4	384.508	Megestrol Acetate
Selleck Chemicals	S1488	c1(CCS(NC)(=O)=O)cc2c([nH]cc2C3CCN(C)CC3)cc1.Cl	C17H26ClN3O2S	371.925	Naratriptan
Selleck Chemicals	S1342	c1(O)cc(O)c2c(OC=C(c3ccc(O)cc3)C2=O)c1	C15H10O5	270.237	Genistein
Selleck Chemicals	S1504	N1(C)C(=O)c2c(ncn2CC(O)CO)N(C)C1=O	C10H14N4O4	254.243	Dyphylline
Selleck Chemicals	S1364	O1[C@H]([C@]12C)C[C@H](C(=Cc3csc(C)n3)C)OC(=O)C[C@@H](O)C(C)(C)C(=O)[C@H](C)[C@@H](O)[C@@H](C)CCC2	C27H41NO6S	507.683	Epothilone B (EPO906, Patupilone)
Selleck Chemicals	S1523	c1(nc(Nc2cc(OC)cc(OC)c2)c(NS(=O)(=O)c3ccc(NC(c4cc(OC)c(C)cc4)=O)cc3)n5)c5cccc1	C31H29N5O6S	599.657	SAR245409 (XL765)
Selleck Chemicals	S1380	c1c(C)c(OCC(=O)N[C@H]([C@@H](O)C[C@@H](NC([C@H](C(C)C)N2C(=O)NCCC2)=O)Cc3ccccc3)Cc4ccccc4)c(C)cc1	C37H48N4O5	628.801	Lopinavir
Selleck Chemicals	S1532	c1(F)cc(c2cc(NC(=O)N)c(C(=O)N[C@H]3CCCNC3)s2)ccc1	C17H19FN4O2S	362.422	AZD7762
Selleck Chemicals	S1003	c1([nH]nc2N)c2c(c3ccc(NC(Nc4cc(C)ccc4F)=O)cc3)ccc1	C21H18FN5O	375.399	Linifanib (ABT-869)
Selleck Chemicals	S1110	c1(CC)c(C(=O)C(=O)N)c2c(cccc2OCC(O)=O)n1Cc3ccccc3	C21H20N2O5	380.394	Varespladib (LY315920)

Selleck Chemicals	S1013	c1nc(C(=O)N[C@@H](Cc2ccccc2)C(=O)N[C@@H](CC(C)C)B(O)O)cnc1	C19H25BN4O4	384.237	Bortezomib (PS-341)
Selleck Chemicals	S1118	c1cc2c(nc(NS(c3ccc(C)cc3)(=O)=O)c(Nc4ccc5c(nsn5)c4)n2)cc1	C21H16N6O2S2	448.521	XL147
Selleck Chemicals	S1028	c1cc2c(c(Nc3cc(Cl)c(OCc4cc(F)ccc4)cc3)ncn2)cc1c5ccc(CNCCS(=O)(C)=O)o5.c6cc(S(=O)(=O)O)ccc6C.c7cc(S(=O)(=O)O)ccc7C	C43H42ClFN4O10S3	925.461	Lapatinib (GW-572016) Ditosylate
Selleck Chemicals	S1133	c1c(OC)c(C(c2c(ccc(Cl)c2)c3c(cnc(Nc4ccc(C(=O)O)c(OC)c4)n3)C5)=N5)c(F)cc1	C27H20ClFN4O4	518.924	Alisertib (MLN8237)
Selleck Chemicals	S1038	c(oc1c2cccn1)(c(N3CCOCC3)nc(c4cc(O)ccc4)n5)c25	C19H16N4O3	348.355	PI-103
Selleck Chemicals	S1144	c1c(C(C)(C)C)c(NC(=O)C2=CNc3c(cccc3)C2=O)cc(O)c1C(C)(C)C	C24H28N2O3	392.491	Ivacaftor (VX-770)
Selleck Chemicals	S1046	c(cc(OCC1CCN(C)CC1)c(OC)c2)(ncnc3Nc4ccc(Br)cc4F)c23	C22H24BrFN4O2	475.354	Vandetanib (ZD6474)
Selleck Chemicals	S1156	N(C(=O)N([C@H]1[C@@H](O)[C@H](O)[C@@H](C)O1)C=C2F)=C2NC(=O)OCCCCC	C15H22FN3O6	359.35	Capecitabine
Selleck Chemicals	S1057	c1cc2c([nH]c(C3=NC(=Cc4[nH]c(C)cc4C)C(OC)=C3)c2)cc1.CS(O)(=O)=O	C21H23N3O4S	413.49	Obatoclax Mesylate (GX15-070)
Selleck Chemicals	S1166	[Pt](N)(Cl)(Cl)N	Cl2H4N2Pt	298.029	Cisplatin
Selleck Chemicals	S1070	c1c(Cl)c(CS(=O)(=O)c2ccc3c(C(=Cc4[nH]c(C)c(C(=O)N5CCC[C@@H]5CN6CCCC6)c4C)C(=O)N3)c2)c(Cl)cc1	C32H34Cl2N4O4S	641.608	PHA-665752
Selleck Chemicals	S1174	C(Nc1ccc(C#N)c(C(F)(F)F)c1)(=O)[C@](O)(C)COc2ccc(C#N)cc2	C19H14F3N3O3	389.328	MK-2866 (GTx-024)
Selleck Chemicals	S1082	c1(Cl)ccc(NC(=O)c2c(Cl)cc(S(=O)(=O)C)cc2)cc1c3ccccn3	C19H14Cl2N2O3S	421.297	Vismodegib (GDC-0449)
Selleck Chemicals	S1185	c1cc(C[C@H](NC(=O)OCc2scnc2)[C@@H](O)C[C@@H](NC(=O)[C@H](C(C)C)NC(=O)N(C)Cc3nc(C(C)C)sc3)Cc4ccccc4)cccc1	C37H48N6O5S2	720.944	Ritonavir
Selleck Chemicals	S1092	c1cc2c(Sc3c(c(C(OC(N4CCOCC4)=CC5=O)=C5)ccc3)S2)cc1	C21H17NO3S2	395.495	KU-55933 (ATM Kinase Inhibitor)
Selleck Chemicals	S1196	C1(=O)C=C([C@]2(C)C=C1)C(=C)C[C@]([H])([C@]23[H])[C@@]([H])([C@@]4(C)CC3)CCC4=O	C20H24O2	296.403	Exemestane
Selleck Chemicals	S1101	c1cc2c(c(Cc3ccncc3)nnc2Nc4ccc(Cl)cc4)cc1.Cl.Cl	C20H17Cl3N4	419.735	Vatalanib (PTK787) 2HCl
Selleck Chemicals	S1206	c1cc(OCC(CNC(C)C)O)ccc1COCCOC(C)C.OC(=O)C=CC(=O)O	C22H35NO8	441.515	Bisoprolol fumarate
Selleck Chemicals	S1219	c1nc(N)ccc1C(Nc2cccc(c3nc(N4CCOCC4)c5c(c6c(nccc6)o5)n3)c2)=O	C25H21N7O3	467.479	YM201636
Selleck Chemicals	S1397	[C@H]1(N2CCOCC2)C[C@@]3(C)[C@@]([H])(CC[C@]4([H])[C@@]3([H])CC[C@@]5(C)[C@@]4([H])C[C@H]([N+]6(CC=C)CCCC6)[C@H]5OC(C)=O)C[C@@H]1O.[Br-]	C32H53BrN2O4	609.678	Rocuronium Bromide
Selleck Chemicals	S1229	n1c(N)c2c(n([C@@H]3O[C@H](COP(O)(O)=O)[C@@H](O)[C@@H]3O)cn2)nc1F	C10H13FN5O7P	365.212	Fludarabine Phosphate
Selleck Chemicals	S1409	c1(OC)cc2c(nc(N(C)CCCNC(=O)C3OCCC3)nc2N)cc1OC.Cl	C19H28ClN5O4	425.91	Alfuzosin HCl
Selleck Chemicals	S1247	c1(NC(=O)c2c(C)onc2)ccc(C(F)(F)F)cc1	C12H9F3N2O2	270.207	Leflunomide
Selleck Chemicals	S1439	c1(OC)cc(C=CC(=O)Nc2c(C(=O)O)cccc2)ccc1OC	C18H17NO5	327.331	Tranilast
Selleck Chemicals	S1260	c1ccc(CCCN[C@]([H])(C)c2c3c(cccc3)ccc2)cc1C(F)(F)F.Cl	C22H23ClF3N	393.873	Cinacalcet HCl
Selleck Chemicals	S1455	c1(OC2CCCC2)cc(C3(C#N)CCC([H])(C(O)=O)CC3)ccc1OC	C20H25NO4	343.417	Cilomilast
Selleck Chemicals	S1276	c1(C2(CC(CC3C2)CC4C3)C4)cc(c5ccc6cc(ccc(C(=O)O)c6)c5)ccc1OC	C28H28O3	412.52	Adapalene
Selleck Chemicals	S1466	[C@@H]1(O)CC(=CC=C2CCC[C@@]3(C)[C@@]2([H])CC[C@H]3[C@H](C)CCCC(O)(C)C)C(=C)[C@@H](O)C1	C27H44O3	416.636	Calcitriol
Selleck Chemicals	S1289	C1(F)=CN(C(=O)NCCCCCC)C(=O)NC1=O	C11H16FN3O3	257.261	Carmofur
Selleck Chemicals	S1481	c(cccn1)(c(OC[C@H](O)CN2CCN([C@H]3c4c(cccc4)[C@H]5[C@H](C5(F)F)c6c3cccc6)CC2)ccc7)c17.Cl.Cl.Cl	C32H34Cl3F2N3O2	636.987	Zosuquidar (LY335979) 3HCl
Selleck Chemicals	S1312	[C@H]1(O)O[C@H](CO)[C@@H](O)[C@H](O)[C@H]1NC(=O)N(C)N=O	C8H15N3O7	265.221	Streptozotocin (STZ)
Selleck Chemicals	S1489	c1(Cl)ccc(c2c(C)nc(NC(=O)C)s2)cc1S(NCCO)(=O)=O	C14H16ClN3O4S2	389.878	PIK-93
Selleck Chemicals	S1344	c1cc(S(=O)(=O)NC(=O)N[C@H]2CC[C@H](C)CC2)ccc1CCNC(=O)N3C(=O)C(CC)=C(C)C3	C24H34N4O5S	490.616	Glimepiride

Selleck Chemicals	S1505	[C@H]1(NC(=O)C(c2csc(N)n2)=NOC(C)(C)C(=O)O)[C@@H](C)N(S(=O)(=O)O)C1=O	C13H17N5O8S2	435.433	Aztreonam
Selleck Chemicals	S1366	c1(F)ccc(CNC(=O)C2=C(O)C(=O)N(C)C(N3CCCCS3(=O)=O)=N2)cc1	C17H19FN4O5S	410.42	BMS-707035
Selleck Chemicals	S1524	c1ccc(Cl)c(C(=O)Nc2c(C(NC3CCNCC3)=O)n[nH]c2)c1Cl	C16H17Cl2N5O2	382.244	AT7519
Selleck Chemicals	S1381	N(C(C(=O)O)=C(SC1CNC(C(N(C)C)=O)C1)C2C)(C(=O)C3C(O)C)C23	C17H25N3O5S	383.463	Meropenem
Selleck Chemicals	S1533	c1(OC)c(OC)cc(Nc2ncc(F)c(Nc3ccc4c(NC(=O)C(C)(C)O4)n3)n2)cc1OC	C22H23FN6O5	470.454	R406 (free base)
Selleck Chemicals	S1004	c1c(C(=O)N)c2c([nH]c([C@]3(C)CCCN3)n2)cc1	C13H16N4O	244.292	Veliparib (ABT-888)
Selleck Chemicals	S1111	c1(NC(=O)C2(CC2)C(=O)Nc3ccc(Oc4ccnc5c4cc(OC)c(OCCCN6CCOCC6)c5)c(F)c3)ccc(F)cc1	C34H34F2N4O6	632.654	Foretinib (GSK1363089)
Selleck Chemicals	S1017	c1(OC)cc2c(ncnc2Oc3ccc4c(cc(C)[nH]4)c3F)cc1OCCCN5CCCC5	C25H27FN4O3	450.505	Cediranib (AZD2171)
Selleck Chemicals	S1119	c(cc(OC)c(OC)c1)(nccc2Oc3ccc(NC(=O)C4(CC4)C(=O)Nc5ccc(F)cc5)cc3)c12	C28H24FN3O5	501.506	Cabozantinib (XL184, BMS-907351)
Selleck Chemicals	S1029	c1cc2c(CN(C3CCC(=O)NC3=O)C2=O)c(N)c1	C13H13N3O3	259.261	Lenalidomide (CC-5013)
Selleck Chemicals	S1134	N(C1CC1)C(Nc2c[nH]nc2c3nc4c(ccc(CN5CCOCC5)c4)[nH]3)=O	C19H23N7O2	381.432	AT9283
Selleck Chemicals	S1039	[C@@H]1(O)CC[C@@H](C[C@@H](C)[C@H]2OC(=O)[C@@H](N3C(=O)C(=O)[C@](O)(O[C@@H]4C[C@H](OC)C(C)=CC=CC=C[C@@H](C)C[C@@H](C)C(=O)[C@@H](OC)[C@H](O)C(C)=C[C@@H](C)C(=O)C2)[C@H](C)CC4)CCCC3)C[C@H]1OC	C51H79NO13	914.172	Rapamycin (Sirolimus)
Selleck Chemicals	S1145	c1(C(C)(C)C)oc(CSc2sc(NC(C3CCNCC3)=O)nc2)nc1	C17H24N4O2S2	380.528	SNS-032 (BMS-387032)
Selleck Chemicals	S1047	c1cc(NC(CCCCCCC(=O)NO)=O)ccc1	C14H20N2O3	264.32	Vorinostat (SAHA, MK0683)
Selleck Chemicals	S1157	C(NC(=O)[C@@H](NC(c1nc(c2ccccc2)ccc1)=O)[C@H](O)C)[C@H](B(O)O)C(C)C	C21H28BN3O5	413.275	CEP-18770 (Delanzomib)
Selleck Chemicals	S1060	c1c2c(C(=O)NN=C2Cc3ccc(F)c(C(=O)N4CCN(C(C5CC5)=O)CC4)c3)ccc1	C24H23FN4O3	434.463	Olaparib (AZD2281, Ku-0059436)
Selleck Chemicals	S1167	c1(C=CCNC(=O)COC)cc2c(ncnc2Nc3cc(C)c(Oc4cnc(C)cc4)cc3)cc1	C27H27N5O3	469.535	CP-724714
Selleck Chemicals	S1071	c1cc2c([C@@]([H])([C@@H](C(=O)OCC)C#N)C(C(=O)OCC)=C(N)O2)cc1Br	C17H17BrN2O5	409.231	HA14-1
Selleck Chemicals	S1175	n1c(Cl)c2c(n(Cc3ncc(C)c(OC)c3C)cn2)nc1N	C14H15ClN6O	318.761	BIIB021
Selleck Chemicals	S1084	c([nH]c(C)c1)(ccc(Oc2c3n(cc(OC[C@H](O)C)c3C)ncn2)c4F)c14	C19H19FN4O3	370.378	Brivanib (BMS-540215)
Selleck Chemicals	S1186	c1cc2c(ccc(C(C)=CC(=O)Nc3ccccc3C(O)=O)c2)cc1	C21H17NO3	331.365	BIBR 1532
Selleck Chemicals	S1093	c1ccc(F)c(NC(=O)c2cc(c3nc4n(cccc4)c3c5nc(Nc6cc(CC)c(N7CCC(N8CCN(S(=O)(=O)C)CC8)CC7)cc6OC)ncc5)ccc2OC)c1F	C44H47F2N9O5S	851.963	GSK1904529A
Selleck Chemicals	S1197	C1=C[C@@]2(C)[C@@]([H])(CC[C@]3([H])[C@@]2([H])CC[C@@]4(C)[C@@]3([H])CC[C@H]4C(=O)NC(C)(C)C)NC1=O	C23H36N2O2	372.544	Finasteride
Selleck Chemicals	S1102	c1cc(SC(=C(C#N)C(C#N)=C(N)Sc2ccccc2N)N)c(N)cc1.CCO	C20H22N6OS2	426.558	U0126-EtOH
Selleck Chemicals	S1207	c1(Oc2c3c(cc(OC)c(OC)c3)ncc2)cc(Cl)c(NC(Nc4noc(C)c4)=O)cc1	C22H19ClN4O5	454.863	Tivozanib (AV-951)
Selleck Chemicals	S1220	c1cc2c(nccc2CNc3ccsc3C(=O)Nc4ccc(OC(F)(F)F)cc4)cc1	C22H16F3N3O2S	443.442	OSI-930
Selleck Chemicals	S1398	O1C(CO)C=CC1N2C=C(C)C(=O)NC2=O	C10H12N2O4	224.213	Stavudine (d4T)
Selleck Chemicals	S1231	c1(O)c(CN(C)C)c2c(nc3c(CN4C3=CC5=C(COC(=O)[C@]5(O)CC)C4=O)c2)cc1.Cl	C23H24ClN3O5	457.907	Topotecan HCl
Selleck Chemicals	S1415	c1c(Cl)c([C@@H](C(OC)=O)N2Cc3c(scc3)CC2)ccc1.OS(=O)(=O)O	C16H18ClNO6S2	419.9	Clopidogrel
Selleck Chemicals	S1248	C(c1c(cc(OC)c([C@@]2(C(=O)OC)C[C@](CN3CCc(c24)c(c5[nH]4)cccc5)([H])C[C@](CC)(O)C3)c1)N6C)([C@]6([H])[C@](C(OC)=O)(O)[C@H]7OC(C)=O)(CC8)[C@]([H])([C@@]7(CC)C=CC9)N89	C46H58N4O9	810.974	Vinblastine
Selleck Chemicals	S1442	c1cc(F)cc(F)c1[C@](O)([C@@H](C)c2c(F)cncn2)Cn3ncnc3	C16H14F3N5O	349.31	Voriconazole
Selleck Chemicals	S1261	c1(n2nc(C(F)(F)F)cc2c3ccc(C)cc3)ccc(S(=O)(=O)N)cc1	C17H14F3N3O2S	381.372	Celecoxib

Selleck Chemicals	S1456	c1(c2ccc(c3nnco3)cc2)ncccc1S(Nc4ncc(C)nc4OC)(=O)=O	C19H16N6O4S	424.433	Zibotentan (ZD4054)
Selleck Chemicals	S1278	n1c(N(C)C)nc(N(C)C)nc1N(C)C	C9H18N6	210.279	Altretamine
Selleck Chemicals	S1467	C1[C@@H](O)CC(=CC=C2[C@@]3([H])[C@@](C)([C@]([H])([C@H](C)C=C[C@@H](C)C(C)C)CC3)CCC2)C(=C)[C@H]1O	C28H44O2	412.648	Doxercalciferol
Selleck Chemicals	S1291	c1c(C(N2CCN(CCOCC(=O)O)CC2)c3ccc(Cl)cc3)cccc1.Cl.Cl	C21H27Cl3N2O3	461.81	Cetirizine DiHCl
Selleck Chemicals	S1482	c1c(c2[nH]c([C@@H]3CCCN3C(=O)[C@@H](NC(=O)OC)C(C)C)nc2)ccc(c4ccc(c5[nH]c([C@@H]6CCCN6C(=O)[C@@H](NC(=O)OC)C(C)C)nc5)cc4)	C40H50N8O6	738.875	Daclatasvir (BMS-790052)
Selleck Chemicals	S1315	c1c(Cl)c(C(OC(Nc2c(c3ccc(CSCCC(=O)O)cc3)onc2C)=O)C)ccc1	C23H23ClN2O5S	474.957	Ki16425
Selleck Chemicals	S1490	c1cc2n(c(C#Cc3cc(C(Nc4ccc(CN5CCN(C)CC5)c(C(F)(F)F)c4)=O)ccc3C)cn2)nc1	C29H27F3N6O	532.559	Ponatinib (AP24534)
Selleck Chemicals	S1352	c1(c2cccc(O)c2)nc3c(nc(N)nc3N)nc1c4cc(O)ccc4	C18H14N6O2	346.343	TG100-115
Selleck Chemicals	S1508	[C@@H]1(CCCCCCC(=O)O)C(=O)C[C@H](O)[C@H]1C=C[C@@H](O)CCCCC	C20H34O5	354.481	Alprostadil
Selleck Chemicals	S1367	c1(ccc2)c(c23)c(C(=O)N(CCN(C)C)C3=O)cc(N)c1	C16H17N3O2	283.325	Amonafide
Selleck Chemicals	S1525	c1cc(C(C)(C)O)nc(N2c3c(cnc(Nc4ccc(N5CCN(C)CC5)cc4)n3)C(=O)N2CC=C)c1	C27H32N8O2	500.595	MK-1775
Selleck Chemicals	S1382	c1(cccc2)c2Cc3c(cccc3)C4N1CCN(C)C4.Cl	C18H21ClN2	300.826	Mianserin HCl
Selleck Chemicals	S1536	c1cc2c(nc(n3cnc(c34)cc(OCCOC)cc4)cc2)c(N5CCC(N)CC5)c1	C24H27N5O2	417.503	CP-673451
Selleck Chemicals	S1005	c1(Sc2ccc3c([nH]nc3C=Cc4ncccc4)c2)c(C(NC)=O)cccc1	C22H18N4OS	386.47	Axitinib
Selleck Chemicals	S1112	c1cc(Sc2nnc3n2nc(c4cn(C)nc4)cc3)cc5c1nccc5	C18H13N7S	359.408	SGX-523
Selleck Chemicals	S1020	c1cc(C(=O)NOCC2CC2)c(Nc3c(Cl)cc(I)cc3)c(F)c1F	C17H14ClF2IN2O2	478.66	PD184352 (CI-1040)
Selleck Chemicals	S1120	C(CO[C@@H]1CC[C@@H](C[C@H](C)[C@@H]2CC(=O)[C@H](C)C=C(C)[C@H](O)[C@@H](OC)C(=O)[C@H](C)C[C@H](C)C=CC=CC=C(C)[C@@H](OC)C[C@@H](O[C@@]3(O)C(=O)C(=O)N([C@@H]4C(=O)O2)CCCC4)CC[C@H]3C)C[C@H]1OC)O	C53H83NO14	958.224	Everolimus (RAD001)
Selleck Chemicals	S1030	c1(CNCCc2c3c(cccc3)[nH]c2C)ccc(C=CC(NO)=O)cc1	C21H23N3O2	349.426	Panobinostat (LBH589)
Selleck Chemicals	S1137	C(OC(C)C)(=O)C(=C1SC=CS1)C(OC(C)C)=O	C12H16O4S2	288.383	Malotilate
Selleck Chemicals	S1040	c1(Cl)c(C(F)(F)F)cc(NC(=O)Nc2ccc(Oc3cc(C(=O)NC)ncc3)cc2)cc1.c4c(S(=O)(=O)O)ccc(C)c4	C28H24ClF3N4O6S	637.027	Sorafenib Tosylate
Selleck Chemicals	S1147	c1(OCCCN(CC)CCO)cc2c(c(Nc3cc(CC(=O)Nc4cc(F)ccc4)n[nH]3)ncn2)cc1	C26H30FN7O3	507.56	Barasertib (AZD1152-HQPA)
Selleck Chemicals	S1048	c1(Nc2n[nH]c(C)c2)nc(Sc3ccc(NC(C4CC4)=O)cc3)nc(N5CCN(C)CC5)c1	C23H28N8OS	464.586	VX-680 (Tozasertib, MK-0457)
Selleck Chemicals	S1158	n1c(c2onc(N3CCC(Oc4cc(F)ccc4Br)CC3)c2)nnn1CC(O)=O	C17H16BrFN6O4	467.249	MK-8245
Selleck Chemicals	S1064	c1(C)ccc(NC(c2ccc(CN3CCN(C)CC3)cc2)=O)cc1Nc4scc(c5cnccc5)n4	C28H30N6OS	498.642	Masitinib (AB1010)
Selleck Chemicals	S1168	CCCC(CCC)C(=O)[O-].[Na+]	C8H15NaO2	166.193	Valproic acid sodium salt (Sodium valproate)
Selleck Chemicals	S1072	n1c(N2CCOCC2)nc(n3c(C(F)F)nc4c3cccc4)nc1N5CCOCC5	C19H21F2N7O2	417.413	ZSTK474
Selleck Chemicals	S1177	c1cc2c(OC(c3c(N)c(OC)ccc3)=CC2=O)cc1	C16H13NO3	267.279	PD98059
Selleck Chemicals	S1085	c1(S(=O)(=O)Nc2ccccc2)cc(C=CC(=O)NO)ccc1	C15H14N2O4S	318.348	Belinostat (PXD101)
Selleck Chemicals	S1188	c1c(Cn2ncnc2)cc(C(C#N)(C)C)cc1C(C#N)(C)C	C17H19N5	293.366	Anastrozole
Selleck Chemicals	S1094	c1(cc(Cn2nnc3c2nc(c4cnn(CCO)c4)cn3)cc5)c5nccc1	C19H16N8O	372.383	PF-04217903
Selleck Chemicals	S1199	n1c(Cl)nc2c(ncn2[C@@H]3O[C@H](CO)[C@@H](O)C3)c1N	C10H12ClN5O3	285.687	Cladribine
Selleck Chemicals	S1103	O1CCN(CCCOc2cc3c(c(Nc4ccc(NC(c5ccccc5)=O)cc4)ncn3)cc2OC)CC1	C29H31N5O4	513.588	ZM 447439
Selleck Chemicals	S1208	c1c(OC)c2c(C(=O)c3c(c(O)c4c(C[C@@](O)(C(=O)CO)C[C@H]4O[C@]5([H])O[C@@H](C)[C@@H](O)[C@@H](N)C5)c3O)C2=O)cc1.Cl	C27H30ClNO11	579.98	Doxorubicin (Adriamycin)

Selleck Chemicals	S1221	c1[nH]c(N=NN(C)C)c(C(N)=O)n1	C6H10N6O	182.183	Dacarbazine
Selleck Chemicals	S1400	n1c(N)c2c(n(C[C@@H](C)OCP(=O)(OCOC(OC(C)C)=O)OCOC(=O)OC(C)C)cn2)nc1.C(C(O)=O)=CC(O)=O	C23H34N5O14P	635.515	Tenofovir Disoproxil Fumarate
Selleck Chemicals	S1233	c1(OC)cc2c(CC[C@]3([H])[C@@]2([H])CC[C@@]4(C)[C@@]3([H])CC[C@H]4O)cc1O	C19H26O3	302.408	2-Methoxyestradiol (2-MeOE2)
Selleck Chemicals	S1422	c1cc(OCCCC(=O)NO)c(C)cc1Cl	C11H14ClNO3	243.687	Droxinostat
Selleck Chemicals	S1249	c1(S(=O)(=O)N)ccc(Nc2nn(C(=O)c3c(F)cccc3F)c(N)n2)cc1	C15H12F2N6O3S	394.356	JNJ-7706621
Selleck Chemicals	S1443	c1cc2c(cc(C(C)N(C(=O)N)O)s2)cc1	C11H12N2O2S	236.29	Zileuton
Selleck Chemicals	S1262	c1(CN(S(=O)(=O)c2ccc(Cl)cc2)[C@H](CCC(F)(F)F)C(=O)N)ccc(c3nocn3)c1F	C20H17ClF4N4O4S	520.885	Avagacestat (BMS-708163)
Selleck Chemicals	S1457	O(C)C(=O)N[C@@H](C(C)(C)C)C(=O)N[C@@H](Cc1ccccc1)[C@@H](O)CN(Cc2ccc(c3ccccn3)cc2)NC(=O)[C@H](C(C)(C)C)NC(=O)OC.S(O)(O)(=O)=O	C38H54N6O11S	802.934	Atazanavir Sulfate
Selleck Chemicals	S1282	[C@@](C)(OO1)(CC2)O[C@@]([H])(OC(=O)[C@H]3C)[C@]1([C@@]3([H])CC[C@@H]4C)[C@@]24[H]	C15H22O5	282.332	Artemisinin
Selleck Chemicals	S1470	c1cc2c(NC(=O)C2=Cc3c(C)c(CCC(=O)O)c(C)[nH]3)cc1	C18H18N2O3	310.347	TSU-68 (SU6668, Orantinib)
Selleck Chemicals	S1294	C1Cc2c(ccc(OCCCCc3nnnn3C4CCCCC4)c2)NC1=O	C20H27N5O2	369.461	Cilostazol
Selleck Chemicals	S1483	c1(OC)cc(C(=O)C)ccc1OCCCN2CCC(c3c4c(cc(F)cc4)on3)CC2	C24H27FN2O4	426.481	Iloperidone
Selleck Chemicals	S1324	c1(nc(N2CCN(C(=O)C3Oc4c(cccc4)OC3)CC2)nc5N)c5cc(OC)c(OC)c1.S(C)(=O)(O)=O	C24H29N5O8S	547.581	Doxazosin Mesylate
Selleck Chemicals	S1494	CC(C)(C)c1[nH]c(c2ccc3c(n(CC(C)(C)C)c(N)n3)n2)c(c4ccc(F)cc4)n1.CS(O)(=O)=O.CS(O)(=O)=O	C26H37FN6O6S2	612.737	LY2228820
Selleck Chemicals	S1354	c1cc2c([nH]c(S(=O)Cc3c(C)c(OCC(F)(F)F)ccn3)n2)cc1	C16H14F3N3O2S	369.361	Lansoprazole
Selleck Chemicals	S1512	N1(C)C(=O)[C@]2([H])N([C@H](c3cc4c(OCO4)cc3)c5c(c6c(cccc6)[nH]5)C2)C(=O)C1	C22H19N3O4	389.404	Tadalafil
Selleck Chemicals	S1368	c1c(C)c(C=CC(C)=CC=CC(C)=CC(=O)O)c(C)c(C)c1OC	C21H26O3	326.429	Acitretin
Selleck Chemicals	S1526	c1cc(c2cn3c(sc4c3ccc(OCCN5CCOCC5)c4)n2)ccc1NC(=O)Nc6noc(C(C)(C)C)c6	C29H32N6O4S	560.667	Quizartinib (AC220)
Selleck Chemicals	S1386	c1(C(Oc2ccc3c(ccc(C(N)=N)c3)c2)=O)ccc(NC(N)=N)cc1.S(=O)(=O)(C)O.S(=O)(=O)(C)O	C21H25N5O8S2	539.582	Nafamostat Mesylate
Selleck Chemicals	S1537	c(C(=O)c1c(c(C)c(C)cc1)O2)(cccc3CC(=O)O)c23	C17H14O4	282.291	DMXAA (Vadimezan)
Selleck Chemicals	S1006	c1c(OC2CCOCC2)c3c(ncnc3Nc4c5c(OCO5)ccc4Cl)cc1OCCN6CCN(C)CC6	C27H32ClN5O5	542.026	Saracatinib (AZD0530)
Selleck Chemicals	S1113	c(nc(c1nonc1N)n2CC)(c(C#CC(C)(O)C)ncc3OC[C@H]4CCCNC4)c23	C21H27N7O3	425.484	GSK690693
Selleck Chemicals	S1021	n1c(Nc2sc(C(=O)Nc3c(C)cccc3Cl)cn2)cc(N4CCN(CCO)CC4)nc1C	C22H26ClN7O2S	488.006	Dasatinib
Selleck Chemicals	S1122	C(Nc1ccccc1N)(=O)c2ccc(CNc3nccc(c4cccnc4)n3)cc2	C23H20N6O	396.444	Mocetinostat (MGCD0103)
Selleck Chemicals	S1032	c1c(CNc2ncccc2C(Nc3cc4c(C(C)(C)CN4)cc3)=O)ccnc1.P(O)(O)(=O)O.P(O)(O)(=O)O	C22H29N5O9P2	569.441	Motesanib Diphosphate (AMG-706)
Selleck Chemicals	S1139	c1c(O)c2c(OC3(CCNCC3)C=C2c4ccc(C(N(CC)CC)=O)cc4)cc1.Cl	C24H29ClN2O3	428.952	ADL5859 HCl
Selleck Chemicals	S1041	n1ccc(c2nc(Nc3cc(C)ccc3)sc2)cc1	C15H13N3S	267.349	STF-62247
Selleck Chemicals	S1150	c1cc(C(N[C@@H](c2ccccc2)[C@@H](O)C(=O)O[C@@H]3C(C)=C4C(C)(C)[C@](O)([C@@H](OC(=O)c5ccccc5)C6[C@@](C)([C@H](O)C[C@@H]7[C@@]6(OC(=O)C)CO7)C(=O)[C@@H]4OC(=O)C)C3)=O)ccc1	C47H51NO14	853.906	Paclitaxel
Selleck Chemicals	S1049	[C@@H]1(C(Nc2ccncc2)=O)CC[C@@H]([C@H](N)C)CC1.Cl.Cl	C14H23Cl2N3O	320.258	Y-27632 2HCl
Selleck Chemicals	S1159	N1=C(c2cc(C(C)C)c(O)cc2O)N(c3ccc(c4c3)n(C)cc4)C(=O)N1	C20H20N4O3	364.398	Ganetespib (STA-9090)
Selleck Chemicals	S1065	c1(c2nc3c(sc(CN4CCN(S(C)(=O)=O)CC4)c3)c(N5CCOCC5)n2)c6c([nH]nc6)ccc1	C23H27N7O3S2	513.636	GDC-0941
Selleck Chemicals	S1169	C(N1C(=NC(N2CCOCC2)=CC1=O)C(C(Nc3ccccc3)C)=C4)=C4C	C21H24N4O2	364.441	TGX-221
Selleck Chemicals	S1075	c1c2c(c(C(C(=O)NC3=O)=C3c4c(Cl)cc(Cl)cc4)cn2C)ccc1	C19H12Cl2N2O2	371.217	SB216763

Selleck Chemicals	S1178	c1cc(Oc2ccc(NC(=O)Nc3cc(C(F)(F)F)c(Cl)cc3)c(F)c2)cc(C(NC)=O)n1	C21H15ClF4N4O3	482.815	Regorafenib (BAY 73-4506)
Selleck Chemicals	S1087	c1c(C(=O)N)cc([N+]([O-])=O)c(I)c1	C7H5IN2O3	292.031	Iniparib (BSI-201)
Selleck Chemicals	S1190	C(Nc1ccc(C#N)c(C(F)(F)F)c1)(=O)C(O)(C)CS(c2ccc(F)cc2)(=O)=O	C18H14F4N2O4S	430.373	Bicalutamide
Selleck Chemicals	S1095	c1(C=CC(=O)NO)ccc(CN(CCc2c3c(cccc3)[nH]c2)CCO)cc1	C22H25N3O3	379.452	LAQ824 (Dacinostat)
Selleck Chemicals	S1200	[C@H]1(N2C(=O)N=C(N)N=C2)C[C@H](O)[C@@H](CO)O1	C8H12N4O4	228.205	Decitabine
Selleck Chemicals	S1104	C(O)Cn1cc(c2cc3c(C(=NO)CC3)cc2)c(c4ccncc4)n1	C19H18N4O2	334.372	GDC-0879
Selleck Chemicals	S1209	C1(=O)NC=C(F)C(=O)N1	C4H3FN2O2	130.077	Fluorouracil (5-Fluoracil, 5-FU)
Selleck Chemicals	S1222	N1C(=O)CN(C[C@H](C)N2CC(=O)NC(=O)C2)CC1=O.Cl	C11H17ClN4O4	304.73	Dexrazoxane HCl (ICRF-187, ADR-529)
Selleck Chemicals	S1403	c1(N(C)C)c2c(C(=O)C(=C3O)[C@]([H])(C[C@]4([H])[C@@]3(O)C(=O)C(C(N)=O)=C(O)[C@@H]4N(C)C)C2)c(O)c(NC(=O)CNC(C)(C)C)c1	C29H39N5O8	585.649	Tigecycline
Selleck Chemicals	S1234	C(#N)C(=Cc1cc(C(C)(C)C)c(O)c(Br)c1)C#N	C14H13BrN2O	305.17	AG-1024
Selleck Chemicals	S1425	c1ccc(C)c(NC(CN2CCN(CC(COc3c(OC)cccc3)O)CC2)=O)c1C.Cl.Cl	C24H35Cl2N3O4	500.458	Ranolazine 2HCl
Selleck Chemicals	S1250	c1(F)cc(N2C(C)(C)C(=O)N(c3cc(C(F)(F)F)c(C#N)cc3)C2=S)ccc1C(=O)NC	C21H16F4N4O2S	464.436	Enzalutamide (MDV3100)
Selleck Chemicals	S1450	[O-]C(=O)[C@@H](C)C[C@H](C(=O)C(C)=C[C@H](C)[C@]1([H])[C@@H](C)[C@@H](O)C[C@@]2(O[C@](C)([C@@]3([H])C[C@H](OC4CC[C@H](OC)[C@@H](C)O4)[C@@H](C)[C@@]5(O[C@@]([H])([C@]6([H])[C@H](C)C[C@@H](C)[C@](O)(CO)O6)C[C@@H]5C)O3)CC2)O1)C.[Na+]	C47H77NaO14	889.096	Nanchangmycin
Selleck Chemicals	S1264	n1cc2c(nc(NC(=O)NC(C)(C)C)c(c3cc(OC)cc(OC)c3)c2)nc1NCCCCN(CC)CC	C28H41N7O3	523.67	PD173074
Selleck Chemicals	S1458	c1c(F)c(SC2=NN3C(=C(c4c(Cl)cccc4Cl)C(=O)N=C3)C=C2)ccc1F	C19H9Cl2F2N3OS	436.262	VX-745
Selleck Chemicals	S1283	c(cccc1)(Oc2c(cc(Cl)cc2)[C@]3([H])[C@]4([H])CN(C)C3)c14.C(O)(=O)C=CC(O)=O	C21H20ClNO5	401.84	Asenapine
Selleck Chemicals	S1472	c1(F)cc(COc2ccc(CN[C@@H](C)C(=O)N)cc2)ccc1.CS(O)(=O)=O	C18H23FN2O5S	398.449	Safinamide Mesylate
Selleck Chemicals	S1297	O1[C@H]([C@H]12)C[C@H](C(=Cc3csc(C)n3)C)OC(=O)C[C@@H](O)C(C)(C)C(=O)[C@H](C)[C@@H](O)[C@@H](C)CCC2	C26H39NO6S	493.656	Epothilone A
Selleck Chemicals	S1484	c1cc(C(=O)C=Cc2cc(C=CC(=O)NO)n(C)c2)cc(F)c1	C17H15FN2O3	314.311	MC1568
Selleck Chemicals	S1326	C1(C)=NN(c2ccccc2)C(=O)C1	C10H10N2O	174.199	Edaravone
Selleck Chemicals	S1497	n1c(N)c2c(ncc(CC(CC#C)c3ccc(C(=O)N[C@H](C(O)=O)CCC(=O)O)cc3)n2)nc1N	C23H23N7O5	477.473	Pralatrexate
Selleck Chemicals	S1358	C1C(=C2c3c(cc(Cl)cc3)CCc4c2nccc4)CCN(C(OCC)=O)C1	C22H23ClN2O2	382.883	Loratadine
Selleck Chemicals	S1514	C1(CC(C)C)C(=O)N(C)C(CC(C)C)C(=O)N(C)C(C(C)C)C(=O)N(C)C([C@H](O)[C@H](C)CC=CC)C(=O)NC(CC)C(=O)N(C)CC(=O)N(C)C(CC(C)C)C(=O)NC(C(C)C)C(=O)N(C)C(CC(C)C)C(=O)NC(C)C(=O)NC(C)C(=O)N1C	C62H111N11O12	1202.611	Cyclosporine
Selleck Chemicals	S1376	C1C[C@@]2([H])C(CC[C@]3([H])[C@@]2([H])CC[C@@]4(CC)[C@@]3([H])C=C[C@@]4(C#C)O)=CC1=O	C21H26O2	310.43	Gestodene
Selleck Chemicals	S1528	c1ncc(c2cc([C@]3(C)CCSC(N)=N3)c(F)cc2F)cn1	C15H14F2N4S	320.36	LY2811376
Selleck Chemicals	S1387	c1(OCC(O)CN2CCN(c3c(OC)cccc3)CC2)cccc4c1cccc4.Cl.Cl	C24H30Cl2N2O3	465.413	Naftopidil DiHCl
Selleck Chemicals	S1538	[C@@]([H])(CCC1)([C@H](C(N[C@H](C(C(NC2CC2)=O)=O)CCC)=O)N(C([C@@H](NC([C@H](C3CCCCC3)NC(c4cnccn4)=O)=O)C(C)(C)C)=O)C5)[C@@]15[H]	C36H53N7O6	679.849	Telaprevir (VX-950)
Selleck Chemicals	S1007	c(ccc(Oc1ccccc1)c2)(c(O)c(C(=O)NCC(=O)O)nc3C)c23	C19H16N2O5	352.341	FG-4592
Selleck Chemicals	S1114	n1c2c(cc(C(F)(F)c3nnc4n3nc(c5cn(C)nc5)cc4)cc2)ccc1	C19H13F2N7	377.35	JNJ-38877605
Selleck Chemicals	S1022	[C@H]1(OC)[C@@H](OP(C)(=O)C)CC[C@@H](C[C@@H](C)[C@H]2OC(=O)[C@@]3([H])N(CCCC3)C(=O)C(=O)[C@](O)(O[C@@]4([H])C[C@H](OC)C(C)=CC=CC=C[C@@H](C)C[C@@H](C)C(=O)[C@H](OC)[C@H](O)C(C)=C[C@@H](C)C(=O)C2)[C@H](C)CC4)C1	C53H84NO14P	990.206	Ridaforolimus (Deforolimus, MK-8669)

Selleck Chemicals	S1124	c(ccc1)(c(Nc2n[nH]c(C3CC3)c2)nc(N4[C@](C(Nc5cnc(F)cc5)=O)(C)CCC4)n6)n16	C23H24FN9O	461.495	BMS-754807
Selleck Chemicals	S1033	c1(C(Nc2cc(n3cnc(C)c3)cc(C(F)(F)F)c2)=O)cc(Nc4nc(c5cccnc5)ccn4)c(C)cc1	C28H22F3N7O	529.516	Nilotinib (AMN-107)
Selleck Chemicals	S1140	C(Nc1ccc([N+]([O-])=O)c(C(F)(F)F)c1)(=O)[C@](O)(C)COc2ccc(NC(C)=O)cc2	C19H18F3N3O6	441.358	Andarine
Selleck Chemicals	S1042	c1(F)cc2c(NC(=O)C2=Cc3c(C)c(C(=O)NCCN(CC)CC)c(C)[nH]3)cc1.C(=O)(O)C[C@H](O)C(=O)O	C26H33FN4O7	532.561	Sunitinib Malate
Selleck Chemicals	S1152	c1(Cl)cc2c([nH]cc2C(c3c(F)ccc(NS(CCC)(=O)=O)c3F)=O)nc1	C17H14ClF2N3O3S	413.826	PLX-4720
Selleck Chemicals	S1052	c1ccc(C(=S)N(C)NC(=O)CC(=O)NN(C(c2ccccc2)=S)C)cc1	C19H20N4O2S2	400.518	Elesclomol (STA-4783)
Selleck Chemicals	S1162	O1C[C@@H](OCc2ccc(OC(F)(F)F)cc2)Cn3c1nc([N+]([O-])=O)c3	C14H12F3N3O5	359.257	PA-824
Selleck Chemicals	S1066	c1c(C(F)(F)F)c(C(C#N)=C(N)Sc2ccc(N)cc2)ccc1	C16H12F3N3S	335.347	SL-327
Selleck Chemicals	S1170	c1(N2CCN(C)CC2)ccc(Nc3nc(Oc4cccc(NC(=O)C=C)c4)c(Cl)cn3)cc1	C24H25ClN6O2	464.947	WZ3146
Selleck Chemicals	S1076	n1ccc(c2[nH]c(c3ccc(S(=O)C)cc3)nc2c4ccc(F)cc4)cc1	C21H16FN3OS	377.435	SB203580
Selleck Chemicals	S1179	c1(N2CCN(C)CC2)ccc(Nc3nc(Sc4cccc(NC(=O)C=C)c4)c(Cl)cn3)cc1	C24H25ClN6OS	481.013	WZ8040
Selleck Chemicals	S1088	c(c(c1cccc(OCc2ccccc2)c1)cn3[C@H]4C[C@H](CN5CCCC5)C4)(c(N)ncn6)c36	C28H31N5O	453.579	NVP-ADW742
Selleck Chemicals	S1191	c1(O)cc(c2cc1)C[C@@H](CCCCCCCCCS(CCCC(F)(F)C(F)(F)F)=O)[C@]([H])([C@]23[H])[C@@]([H])([C@@]4(C)CC3)CC[C@@H]4O	C32H47F5O3S	606.771	Fulvestrant
Selleck Chemicals	S1096	c1cc2c(n(C)cc2CNCC3CCN(c4ncc(C(NO)=O)cn4)CC3)cc1	C21H26N6O2	394.47	Quisinostat (JNJ-26481585)
Selleck Chemicals	S1201	S(=O)(=O)([O-])CCSSCCS(=O)(=O)[O-].[Na+].[Na+]	C4H8Na2O6S4	326.342	Dimesna
Selleck Chemicals	S1105	c1cc2c(OC(N3CCOCC3)=CC2=O)c(c4ccccc4)c1	C19H17NO3	307.343	LY294002
Selleck Chemicals	S1210	n1c(N)c2c(ncc(CN(C)c3ccc(C(=O)N[C@H](C(O)=O)CCC(O)=O)cc3)n2)nc1N	C20H22N8O5	454.439	Methotrexate
Selleck Chemicals	S1224	[Pt+2].[O-]C(=O)C(=O)[O-].C1C[C@@H](N)[C@H](N)CC1	C8H14N2O4Pt	397.286	Oxaliplatin
Selleck Chemicals	S1404	C(C#N)(C[C@@]1(C)[C@@]2(CC[C@]3([H])[C@@]1([H])CC[C@@]4(C)[C@@]3([H])CC[C@H]4O)[C@]5([H])O2)=C5O	C20H27NO3	329.433	Trilostane
Selleck Chemicals	S1237	N1(C)C(=O)n2c(c(C(=O)N)nc2)N=N1	C6H6N6O2	194.151	Temozolomide
Selleck Chemicals	S1426	c1(OCC)cc(CC(=O)N[C@@H](CC(C)C)c2c(N3CCCCC3)cccc2)ccc1C(=O)O	C27H36N2O4	452.586	Repaglinide
Selleck Chemicals	S1251	C1(=O)C=C(C(=C2[C@]3([H])[C@@]([H])([C@@]4(C)CC2)CC[C@]4(CC#N)O)CC1)CC3	C20H25NO2	311.418	Dienogest
Selleck Chemicals	S1451	n1c(Nc2ccc(CC(=O)N3CCN(CC)CC3)cc2)nc(Nc4ccc(C(=O)Nc5c(Cl)cccc5)cc4)c(F)c1	C31H31ClFN7O2	588.075	Aurora A Inhibitor I
Selleck Chemicals	S1266	c1cc(c2nc(N3CCOCC3)c4c(n(C5CCN(C(OC)=O)CC5)nc4)n2)ccc1NC(=O)OC	C24H29N7O5	495.531	WYE-354
Selleck Chemicals	S1459	c1(Nc2ncncc2)nc(C(=O)NCc3ccccc3)cs1	C15H13N5OS	311.362	Thiazovivin
Selleck Chemicals	S1285	c1cccc(C(O)(C2C(CC3C2)C=C3)CCN4CCCCC4)c1.Cl	C21H30ClNO	347.922	Biperiden HCl
Selleck Chemicals	S1474	C1(=O)NC(C)=C(C(=O)Nc2cc3c([nH]nc3)cc2F)C(c4ccc(C(F)(F)F)cc4)C1	C21H16F4N4O2	432.371	GSK429286A
Selleck Chemicals	S1299	O1[C@H](N2C(=O)NC(=O)C(F)=C2)C[C@H](O)[C@H]1CO	C9H11FN2O5	246.192	Floxuridine
Selleck Chemicals	S1485	c1c(N(C(=O)C)S(=O)(=O)c2ccc(OC)cc2)c(C=Cc3cc[n+]([O-])cc3)ccc1	C22H20N2O5S	424.47	HMN-214
Selleck Chemicals	S1329	n1(C(c2ccccc2)C)cncc1C(=O)OCC	C14H16N2O2	244.289	Etomidate
Selleck Chemicals	S1500	C1=C[C@@]2(C)C(CC[C@]3([H])[C@@]2(F)[C@H](O)C[C@@]4(C)[C@@]3([H])C[C@H](C)[C@@]4(O)C(=O)CO)=CC1=O	C22H29FO5	392.461	Betamethasone
Selleck Chemicals	S1360	c1(C=C2SC(=O)NC2=O)cc3c(nccc3c4ccncc4)cc1	C18H11N3O2S	333.364	GSK1059615
Selleck Chemicals	S1515	c1(C=CC(=O)NO)cc2c(n(CCN(CC)CC)c(CCCC)n2)cc1	C20H30N4O2	358.478	Pracinostat (SB939)

Selleck Chemicals	S1377	C1C[C@@]2(C)C([C@@]3([H])[C@]([H])(C3)[C@]4([H])[C@@]2([H])CC[C@@]5(C)[C@@]4([H])[C@@]6([H])[C@]([H])(C6)[C@]57CCC(=O)O7)=CC1=O	C24H30O3	366.493	Drospirenone
Selleck Chemicals	S1529	c1(NS(=O)(=O)CC)cc2c(NC(=O)C2=C(Nc3ccc(CN4CCCCC4)cc3)c5ccccc5)cc1	C29H32N4O3S	516.654	Hesperadin
Selleck Chemicals	S1390	c1cc2c(n(C)c3c2C(=O)C(Cn4ccnc4C)CC3)cc1.Cl	C18H20ClN3O	329.824	Ondansetron HCl
Selleck Chemicals	S1540	C1C(CC(CC2([C@H](N)C(=O)N3[C@@]4([H])[C@@]([H])(C4)C[C@H]3C#N)C5)(O)CC15)C2	C18H25N3O2	315.41	Saxagliptin
Selleck Chemicals	S1008	c1c(C(=O)NOCCO)c(Nc2c(Cl)cc(Br)cc2)c(F)c3c1n(C)cn3	C17H15BrClFN4O3	457.681	Selumetinib (AZD6244)
Selleck Chemicals	S1115	c1cc(c2ccc([C@@H](C(F)(F)F)N[C@@H](CC(F)(C)C)C(NC3(CC3)C#N)=O)cc2)ccc1S(C)(=O)=O	C25H27F4N3O3S	525.559	Odanacatib (MK-0822)
Selleck Chemicals	S1023	c1(N[C](C)(c2c(cc(OCCOC)c(OCCOC)c2)N=C3)=N3)cc(C#C)ccc1.Cl	C23H27ClN3O4	444.931	Erlotinib HCl (OSI-744)
Selleck Chemicals	S1129	n1c(c2ncc1C(Nc3ccccc3c4cn(c5n4)c(CN6CCNCC6)cs5)=O)cccc2.Cl	C25H24ClN7OS	506.022	SRT1720
Selleck Chemicals	S1034	c1cc(OCc2ccccc2)cc(c3c4c(ncnc4N)n([C@H]5C[C@@H](CN6CCC6)C5)c3)c1	C27H29N5O	439.552	NVP-AEW541
Selleck Chemicals	S1141	C(NCC=C)(C1=O)=C(C[C@@H](C)C[C@H](OC)[C@H](O)[C@@H](C)C=C(C)[C@H](OC(N)=O)[C@@H](OC)C=CC=C(C)C(=O)N2)C(=O)C2=C1	C31H43N3O8	585.688	17-AAG (Tanespimycin)
Selleck Chemicals	S1043	c1c2c(c(N3CCN(C(=O)Nc4ccc(OC(C)C)cc4)CC3)ncn2)cc(OC)c1OCCCN5CCCCC5	C31H42N6O4	562.703	Tandutinib (MLN518)
Selleck Chemicals	S1153	n1c(N[C@H](CC)CO)nc(NCc2ccccc2)c3c1n(C(C)C)cn3	C19H26N6O	354.449	Roscovitine (Seliciclib,CYC202)
Selleck Chemicals	S1053	c1cc(C(=O)Nc2c(N)cccc2)ccc1CNC(=O)OCc3cccnc3	C21H20N4O3	376.409	Entinostat (MS-275)
Selleck Chemicals	S1163	C(c1cc(C(C)C)c(O)cc1O)(N2Cc(c3C2)cc(CN4CCN(C)CC4)cc3)=O	C24H31N3O3	409.521	AT13387
Selleck Chemicals	S1067	c1nc(c2c(c3ccc4c(OCO4)c3)nc(c5ccc(C(N)=O)cc5)[nH]2)ccc1	C22H16N4O3	384.387	SB431542
Selleck Chemicals	S1171	c1(Nc2ccc(N3CCOCC3)cc2)nc(c4c(C)nc(N)s4)ccn1	C18H20N6OS	368.456	CYC116
Selleck Chemicals	S1077	c1cc(c2c(c3ccncc3)[nH]c(c4ccc(O)cc4)n2)ccc1F	C20H14FN3O	331.343	SB202190 (FHPI)
Selleck Chemicals	S1180	c1c(C(F)(F)F)ccc(c2nc3c(CSCC3)c(O)n2)c1	C14H11F3N2OS	312.31	XAV-939
Selleck Chemicals	S1089	c1(NS(=O)(=O)C2(CC2)C[C@H](O)CO)c(Nc3c(F)cc(I)cc3)c(F)c(F)cc1OC	C19H20F3IN2O5S	572.337	Refametinib (RDEA119, Bay 86-9766)
Selleck Chemicals	S1192	C(O)(=O)[C@@H](NC(c1sc(N(C)Cc2ccc(c3c2)N=C(C)NC3=O)cc1)=O)CCC(O)=O	C21H22N4O6S	458.488	Raltitrexed
Selleck Chemicals	S1097	c1(C(F)(F)F)cc2c(SC(N3CCC4(OC(C)CO4)CC3)=NC2=O)c([N+]([O-])=O)c1	C17H16F3N3O5S	431.386	BTZ043 Racemate
Selleck Chemicals	S1202	C1=C[C@@]2(C)[C@@]([H])(CC[C@]3([H])[C@@]2([H])CC[C@@]4(C)[C@@]3([H])CC[C@H]4C(=O)Nc5c(C(F)(F)F)ccc(C(F)(F)F)c5)NC1=O	C27H30F6N2O2	528.53	Dutasteride
Selleck Chemicals	S1106	c(cccc1)(c2c(cc(c3n(c4ccc(NC(=O)CN)cc4)nc(C(F)(F)F)c3)cc2)cc5)c15	C26H19F3N4O	460.45	OSU-03012 (AR-12)
Selleck Chemicals	S1212	c1cc2c(nc(CCCC(=O)O)n2C)cc1N(CCCl)CCCl.Cl	C16H22Cl3N3O2	394.724	Bendamustine HCl
Selleck Chemicals	S1225	[C@@]([H])(C(=O)OC1)([C@@H](c2cc(OC)c(O)c(OC)c2)c3c(cc4c(OCO4)c3)[C@H]5O[C@@]6([H])[C@@H](O)[C@@H](O)[C@@]7([H])[C@@]([H])(CO[C@@H](C)O7)O6)[C@]15[H]	C29H32O13	588.557	Etoposide
Selleck Chemicals	S1405	C1CCN([C@H]2C[C@@]3(C)[C@@]([H])(CC[C@]4([H])[C@@]3([H])CC[C@@]5(C)[C@@]4([H])C[C@H]([N+]6(C)CCCCC6)[C@H]5OC(=O)C)C[C@@H]2OC(=O)C)CC1.[Br-]	C34H57BrN2O4	637.731	Vecuronium Bromide
Selleck Chemicals	S1241	c1(OC)c([C@@]2(C(=O)OC)C[C@@]3([H])CN(C[C@@](CC)(O)C3)CCc4c2[nH]c5c4cccc5)cc6c(N(C=O)[C@]7([H])[C@]6(CC8)[C@@]([H])(N89)[C@@](CC)(C=CC9)[C@@H](OC(C)=O)[C@]7(O)C(=O)OC)c1.S(O)(O)(=O)=O	C46H58N4O14S	923.036	Vincristine
Selleck Chemicals	S1430	c1(OC2CCCC2)cc(C3CC(=O)NC3)ccc1OC	C16H21NO3	275.343	Rolipram
Selleck Chemicals	S1255	C(N)(=O)Cc1cccc(C(=O)c2ccccc2)c1N	C15H14N2O2	254.284	Nepafenac
Selleck Chemicals	S1452	c1cccc(CN2C([C@@H](C(C)C)N(C(=O)c3ccc(C)cc3)CCCN)=Nc4c(ccc(Cl)c4)C2=O)c1	C30H33ClN4O2	517.062	Ispinesib (SB-715992)
Selleck Chemicals	S1267	c1(c2ccc(Cl)cc2)cc3c([nH]cc3C(c4c(F)ccc(NS(CCC)(=O)=O)c4F)=O)nc1	C23H18ClF2N3O3S	489.922	Vemurafenib (PLX4032, RG7204)
Selleck Chemicals	S1460	c1cc2c(c3c(c4[nH]n3)c(ccc4)C2=O)cc1	C14H8N2O	220.226	SP600125

Selleck Chemicals	S1286	C1=C[C@@]2(C)C(CC[C@]3([H])[C@@]2([H])[C@H](O)C[C@@]4(C)[C@@]3([H])C[C@@H]5[C@]4(C(=O)CO)O[C@]([H])(CCC)O5)=CC1=O	C25H34O6	430.534	Budesonide
Selleck Chemicals	S1475	c1c(F)c(Nc2c(C(=O)NC[C@H](O)CO)ccnc2)ccc1I	C15H15FIN3O3	431.201	Pimasertib (AS-703026)
Selleck Chemicals	S1300	N1C(=O)C(F)=CN([C@H]2CCCO2)C1=O	C8H9FN2O3	200.167	FT-207 (NSC 148958)
Selleck Chemicals	S1486	c1nc2c(cc(c3ccc(CN4CCN(CC)CC4)cc3)[nH]2)c(N[C@H](C)c5ccccc5)n1	C27H32N6	440.583	AEE788 (NVP-AEE788)
Selleck Chemicals	S1331	c1(C(O)(Cn2cncn2)Cn3cncn3)ccc(F)cc1F	C13H12F2N6O	306.271	Fluconazole
Selleck Chemicals	S1501	c(C(=O)OC1)(c(O)c(CC=C(C)CCC(=O)OCCN2CCOCC2)c(OC)c3C)c13	C23H31NO7	433.495	Mycophenolate Mofetil
Selleck Chemicals	S1361	c1(CC(=O)NC(=S)Nc2cc(F)c(Oc3ccnc4c3sc(c5cn(C)cn5)c4)cc2)ccccc1	C26H20FN5O2S2	517.598	MGCD-265 analog
Selleck Chemicals	S1517	[C@@H]1(N)[C@@H](O)[C@]([H])(O[C@@H]2C[C@]([H])(O[C@]3(O)C[C@@H](O)C[C@]4([H])[C@H](O4)C=CC(=O)O[C@H](C)CC=CC=CC=CC=C2)[C@H](C(O)=O)[C@@H](O)C3)O[C@H](C)[C@H]1O	C33H47NO13	665.725	Natamycin
Selleck Chemicals	S1378	n1c(c2cnn([C@H](C3CCCC3)CC#N)c2)c4c([nH]cc4)nc1	C17H18N6	306.365	Ruxolitinib (INCB018424)
Selleck Chemicals	S1530	c1cc2c(NC(=O)C2=C(c3ccccc3)Nc4cccc(CN(C)C)c4)cc1C(=O)NC	C26H26N4O2	426.51	BIX 02188
Selleck Chemicals	S1392	c1(OCC)cc2c(c(Nc3ccc(F)c(Cl)c3)c(C#N)cn2)cc1NC(=O)C=CCN(C)C	C24H23ClFN5O2	467.923	Pelitinib (EKB-569)
Selleck Chemicals	S1541	[nH]1c2c(cc(Cl)cc2)c3c1C(C(N)=O)CCC3	C13H13ClN2O	248.708	EX 527 (Selisistat)
Selleck Chemicals	S1545	c1c(F)c2c(c(NC(=O)Nc3ccc(N(C)C)cc3)cc(C)n2)cc1F	C19H18F2N4O	356.369	SB408124
Selleck Chemicals	S1712	c1c(C(=O)O)ccc(n2nc(c3c(O)cccc3)nc2c4c(O)cccc4)c1	C21H15N3O4	373.362	Deferasirox
Selleck Chemicals	S1558	c1(Cl)ccc(C2(CCNCC2)c3ccc(c4cn[nH]c4)cc3)cc1	C20H20ClN3	337.846	AT7867
Selleck Chemicals	S1725	c1cc2c(cccc2CN(CC=CC#CC(C)(C)C)C)cc1	C21H25N	291.43	Terbinafine
Selleck Chemicals	S1573	c1ccc(S(=O)(=O)N2CCCNCC2)c3c1cncc3.Cl	C14H18ClN3O2S	327.83	Fasudil (HA-1077) HCl
Selleck Chemicals	S1737	C1=C[C@@]2(C)C(CC[C@]3([H])[C@@]2([H])[C@H](O)C[C@@]4(C)[C@@]3([H])CC[C@@]4(O)C(CO)=O)=CC1=O	C21H28O5	360.444	Prednisolone
Selleck Chemicals	S1594	C(C)(C)[C@H](O)C(=O)N[C@@H](C)C(=O)N[C@H]1C(=O)N(C)CCc2c1cccc2	C19H27N3O4	361.435	Semagacestat (LY450139)
Selleck Chemicals	S1762	c1nc(C(N)=O)cnc1	C5H5N3O	123.113	Pyrazinamide
Selleck Chemicals	S1618	c1(N)ccc(S(=O)(=O)Nc2ncc(OC)cn2)cc1	C11H12N4O3S	280.303	Sulfameter
Selleck Chemicals	S1784	c1nc2c(ncn2[C@@H]3O[C@H](CO)[C@@H](O)[C@@H]3O)c(N)n1	C10H13N5O4	267.241	Vidarabine
Selleck Chemicals	S1629	C1(=O)O[C@@H](C[C@@H](OC([C@@H](NC=O)CC(C)C)=O)CCCCCCCCCCC)[C@@H]1CCCCCC	C29H53NO5	495.735	Orlistat
Selleck Chemicals	S1806	c1nc(C(O)=O)c[n+]([O-])c1C	C6H6N2O3	154.123	Acipimox
Selleck Chemicals	S1644	c1([N+](=O)[O-])oc(C=NNC(=O)N)cc1	C6H6N4O4	198.136	Nitrofural
Selleck Chemicals	S1833	c1c(Cl)c(SC(CCc2ccc(Cl)cc2)Cn3ccnc3)c(Cl)cc1.[N+](O)(=O)[O-]	C19H18Cl3N3O3S	474.789	Butoconazole nitrate
Selleck Chemicals	S1658	c1cc(CCN(C)CCOc2ccc(NS(=O)(=O)C)cc2)ccc1NS(=O)(=O)C	C19H27N3O5S2	441.565	Dofetilide
Selleck Chemicals	S1849	c1cc2c(OC=C(c3ccc(O)cc3)C2=O)cc1O	C15H10O4	254.238	Daidzein
Selleck Chemicals	S1673	NCCN.N1(C)C(=O)c2c(nc[nH]2)N(C)C1=O.N3(C)C(=O)c4c(nc[nH]4)N(C)C3=O	C16H24N10O4	420.426	Aminophylline
Selleck Chemicals	S1883	C(I)(C(=O)NC(=O)N1[C@H]2C[C@H](O)[C@@H](CO)O2)=C1	C9H11IN2O5	354.099	Idoxuridine
Selleck Chemicals	S1692	CS(=O)(=O)OCCCCOS(=O)(=O)C	C6H14O6S2	246.302	Busulfan
Selleck Chemicals	S1903	c1ccc(Cl)c(Nc2ccccc2CC(=O)[O-])c1Cl.[Na+]	C14H10Cl2NNaO2	318.13	Diclofenac Sodium
Selleck Chemicals	S1916	c1cc(S(=O)(=O)Nc2onc(C)c2C)ccc1N	C11H13N3O3S	267.304	Sulfisoxazole

Selleck Chemicals	S2134	c1c(F)c(NC2=C(C(=O)NOCCO)C=C(C)C(=O)N2C)ccc1I	C16H17FIN3O4	461.227	AZD8330
Selleck Chemicals	S1952	C1=Cc2c(c(OC)c3c(cco3)c2)OC1=O	C12H8O4	216.19	Methoxsalen
Selleck Chemicals	S2161	c(nc(Nc1ccc(C(F)(F)F)cc1)n2C)(cc(Oc3cc(c4ncc(C(F)(F)F)[nH]4)ncc3)cc5)c25	C24H16F6N6O	518.414	RAF265 (CHIR-265)
Selleck Chemicals	S1974	CNC[C@H](O)[C@@H](O)[C@H](O)[C@H](O)CO	C7H17NO5	195.214	Meglumine
Selleck Chemicals	S2182	c1cccc(CC2=C([C@@H](C(C)C)N(CCCN)C(c3ccc(C)cc3)=O)Oc4c(ccc(Cl)c4)C2=O)c1.Cl	C31H34Cl2N2O3	553.519	SB743921
Selleck Chemicals	S2005	N1(C)C(=O)C(O)=C(C(=O)NCc2ccc(F)cc2)N=C1C(C)(C)NC(=O)c3oc(C)nn3	C20H21FN6O5	444.416	Raltegravir (MK-0518)
Selleck Chemicals	S2201	n1ccc(Oc2c(F)cc(NC(=O)C(C(=O)C(c3ccc(F)cc3)=CN4)=C4)cc2)c(Cl)c1N	C23H15ClF2N4O3	468.84	BMS-794833
Selleck Chemicals	S2026	O=C1O[C@]2([H])[C@@](O)([C@]3(O4)C(C([C@]([H])(O)C(=O)O5)([C@H]45)[C@]([H])(C(C)(C)C)C6)([C@]6([H])OC3=O)C2)[C@]1([H])C	C20H24O9	408.399	Ginkgolide A
Selleck Chemicals	S2218	n1c(N)c2c(n(C(C)C)nc2c3cc4c(ccc(O)c4)[nH]3)nc1	C16H16N6O	308.338	PP242
Selleck Chemicals	S2038	c1(cccc(O)c1)N(CC2=NCCN2)c3ccc(C)cc3.CS(=O)(=O)O	C18H23N3O4S	377.458	Phentolamine Mesylate
Selleck Chemicals	S2229	c1ccc(c2cc(C(=O)O)ccc2)c(O)c1NN=C3C(C)=NN(c4cc(C)c(C)cc4)C3=O	C25H22N4O4	442.467	Eltrombopag
Selleck Chemicals	S2055	C(Cl)(C(O)=CC(=O)N1)=C1	C5H4ClNO2	145.544	Gimeracil
Selleck Chemicals	S2244	c1cccc([C@H](C(C)C)C(=O)Nc2ccc(C(=O)NO)cc2)cc1	C18H20N2O3	312.363	AR-42
Selleck Chemicals	S2066	n1c(Cl)c(NC(NCC2)=N2)c(OC)nc1C	C9H12ClN5O	241.677	Moxonidine
Selleck Chemicals	S2261	[C@@H]1(O)CC[C@]2(C)[C@]([H])(CCC(=C)[C@@H]2CC=C3C(O)COC3=O)[C@]1(C)CO	C20H30O5	350.449	Andrographolide
Selleck Chemicals	S2097	[C@H](C(OC)(c1ccccc1)c2ccccc2)(C(=O)O)Oc3nc(C)cc(C)n3	C22H22N2O4	378.421	Ambrisentan
Selleck Chemicals	S2269	[C@@H]1(O)[C@H](C(O)=O)O[C@H](Oc2cc3c(C(=O)C=C(c4ccccc4)O3)c(O)c2O)[C@H](O)[C@H]1O	C21H18O11	446.361	Baicalin
Selleck Chemicals	S2120	c1(O)c(CN(C)C)c2c(n(C)c(CSc3ccccc3)c2C(=O)OCC)cc1Br.Cl	C22H26BrClN2O3S	513.875	Arbidol HCl
Selleck Chemicals	S2287	C([C@H](C[C@@H]1C2)CNC1)(=CC=CC3=O)N23	C11H14N2O	190.242	Cytisine
Selleck Chemicals	S1547	c1(C#N)cc(c2nc(C)c(C(=O)O)s2)ccc1OCC(C)C	C16H16N2O3S	316.375	Febuxostat
Selleck Chemicals	S1713	c1ccc(NC(=O)C2=C(O)c3c(cccc3)S(=O)(=O)N2C)nc1	C15H13N3O4S	331.346	Piroxicam
Selleck Chemicals	S1561	c1(Cl)c(Oc2c(F)cc(NC(C3=C(OCC)C=CN(c4ccc(F)cc4)C3=O)=O)cc2)ccnc1N	C25H19ClF2N4O4	512.893	BMS-777607
Selleck Chemicals	S1729	c1c(C)c(OCCCC(C)(C)C(O)=O)cc(C)c1	C15H22O3	250.333	Gemfibrozil
Selleck Chemicals	S1574	c1cc(OCCN2CCOCC2)c3c(cccc3)c1NC(=O)Nc4n(c5ccc(C)cc5)nc(C(C)(C)C)c4	C31H37N5O3	527.657	BIRB 796 (Doramapimod)
Selleck Chemicals	S1738	c1cccc(c2ccc(Cn3c4c(c(C)cc(c5nc6c(cccc6)n5C)c4)nc3CCC)cc2)c1C(=O)O	C33H30N4O2	514.617	Telmisartan
Selleck Chemicals	S1601	c(c1c(cc(OC)cc1)[nH]2)(CCN3[C@]4([H])C[C@@]5([H])[C@]([H])(C[C@@H](OC(c6cc(OC)c(OC)c(OC)c6)=O)[C@H](OC)[C@H]5C(=O)OC)C3)c24	C33H40N2O9	608.679	Reserpine
Selleck Chemicals	S1764	c1(C=NN2CCN(C)CC2)c(O)c(c(C(=O)[C@]3(C)O4)c45)c(c(O)c5C)c(O)c1NC(=O)C(C)=CC=C[C@H](C)[C@H](O)[C@@H](C)[C@@H](O)[C@@H](C)[C@H](OC(C)=O)[C@H](C)[C@@H](OC)C=CO3	C43H58N4O12	822.94	Rifampin
Selleck Chemicals	S1620	[C@@]([H])(OCC1)(OC[C@@]2(OC(=O)N[C@@]([C@@](O)([H])CN(S(=O)(=O)c3ccc(N)cc3)CC(C)C)([H])Cc4ccccc4)[H])[C@@]12[H].CCO	C29H43N3O8S	593.732	Darunavir Ethanolate
Selleck Chemicals	S1787	c1c2c(OCO2)cc3c1[C@@H](O[C@@H]4O[C@H]5[C@@H](O[C@H](c6sccc6)OC5)[C@H](O)[C@H]4O)[C@@H]7[C@]([H])(C(=O)OC7)[C@H]3c8cc(OC)c(O)c(OC)c8	C32H32O13S	656.654	Teniposide
Selleck Chemicals	S1631	C(NC(=O)c1c2[nH]nc1)=N2.[Na+]	C5H4N4NaO	159.101	Allopurinol Sodium
Selleck Chemicals	S1807	OCCOCn1cnc2c1N=C(N)NC2=O	C8H11N5O3	225.205	Aciclovir
Selleck Chemicals	S1645	c1cc(C(=O)c2cc(C(C)C(=O)O)ccc2)ccc1	C16H14O3	254.281	Ketoprofen

Selleck Chemicals	S1835	[C@@H]1(O)[C@@H](C)N(C)C[C@H](C)C[C@](O)(C)[C@H](O[C@H]2[C@@H](O)[C@@H](N(C)C)C[C@@H](C)O2)[C@@H](C)[C@H](O[C@@H]3O[C@@H](C)[C@H](O)[C@@](OC)(C)C3)[C@H](C)C(=O)O[C@H](CC)[C@@]1(C)O	C38H72N2O12	748.984	Azithromycin
Selleck Chemicals	S1662	c1cc2c(non2)c(C3C(C(=O)OC(C)C)=C(C)NC(C)=C3C(OC)=O)c1	C19H21N3O5	371.387	Isradipine
Selleck Chemicals	S1855	c1(F)cc2c(N(CC)C=C(C(O)=O)C2=O)cc1N3CCN(C)CC3.S(O)(=O)(=O)C	C18H24FN3O6S	429.463	Pefloxacin Mesylate
Selleck Chemicals	S1676	c1cc(CC(C)CN2C[C@@H](C)O[C@@H](C)C2)ccc1C(C)(C)CC.Cl	C21H36ClNO	353.97	Amorolfine HCl
Selleck Chemicals	S1884	[C@@H]1(C)CN(c2c(F)c(N)c3c(N(C4CC4)C=C(C(=O)O)C3=O)c2F)C[C@H](C)N1	C19H22F2N4O3	392.4	Sparfloxacin
Selleck Chemicals	S1701	C1=C[C@@]2(C)C(CC[C@]3([H])[C@@]2([H])[C@H](O)C[C@@]4(C)[C@@]3([H])C[C@]5([H])[C@]4(C(=O)CO)OC(C)(C)O5)=CC1=O	C24H32O6	416.507	Desonide
Selleck Chemicals	S1904	c1cc(C(=O)CC(=O)c2ccc(C(C)(C)C)cc2)ccc1OC	C20H22O3	310.387	Avobenzone
Selleck Chemicals	S1917	c1c(N(C)C)ccc(C(=C2C=CC(=[N+](C)C)C=C2)c3ccc(N(C)C)cc3)c1.[Cl-]	C25H30ClN3	407.979	Crystal Violet
Selleck Chemicals	S2149	c1(c2ccc(S(C)(=O)=O)cc2)ncc(OCC3CCN(c4onc(C(C)C)n4)CC3)cc1	C23H28N4O4S	456.558	GSK1292263
Selleck Chemicals	S1956	c1cc(C(Cn2cncc2)OCc3c(Cl)cc(Cl)cc3)c(Cl)cc1Cl.O[N+]([O-])=O	C18H15Cl4N3O4	479.141	Miconazole Nitrate
Selleck Chemicals	S2162	c1(Nc2cc(C)[nH]n2)c(Cl)cnc(N[C@@H](C)c3ncc(F)cn3)n1	C14H14ClFN8	348.766	AZD1480
Selleck Chemicals	S1978	[Br-].[C@@]1([H])(C[C@H](OC(=O)[C@H](CO)c2ccccc2)C3)[N+](C)(C)[C@]3([H])[C@@]4([H])[C@]1([H])O4	C18H24BrNO4	398.291	Methscopolamine
Selleck Chemicals	S2185	C(Nc1ccc(c2c1)ncnc2Nc3ccc(OCc4cccc(F)c4)c(Cl)c3)(=O)C=C.c5c(S(=O)(=O)O)ccc(C)c5	C31H26ClFN4O5S	621.078	AST-1306
Selleck Chemicals	S2006	n1c(N)c(c2ccc(Cl)cc2)c(CC)nc1N	C12H13ClN4	248.711	Pyrimethamine
Selleck Chemicals	S2202	c(cnn1C)(c(Nc2c(C)ccc(C(=O)Nc3cc(C(F)(F)F)ccc3)c2)nc(c4cnccc4)n5)c15	C26H20F3N7O	503.479	NVP-BHG712
Selleck Chemicals	S2028	c1cc(C(C#N)c2c(Cl)cc(N3C(=O)NC(=O)C=N3)cc2Cl)ccc1Cl	C17H9Cl3N4O2	407.638	Diclazuril
Selleck Chemicals	S2219	c1cc(Nc2nc(c3ccc(C(=O)NCC#N)cc3)ccn2)ccc1N4CCOCC4	C23H22N6O2	414.46	CYT387
Selleck Chemicals	S2040	c1c(NS(C)(=O)=O)c(Oc2ccccc2)cc([N+]([O-])=O)c1	C13H12N2O5S	308.31	Nimesulide
Selleck Chemicals	S2230	c1(C)nc(c2c(c3c4c(ccc(C(N)=O)c4)ncc3)c5n(CCC5)n2)ccc1	C22H19N5O	369.419	LY2157299
Selleck Chemicals	S2058	c1(cccc(C)c1)N(C)C(=S)Oc2cc3c(cccc3)cc2	C19H17NOS	307.409	Tolnaftate
Selleck Chemicals	S2247	c1c(C(F)(F)F)c(c2cc(N3CCOCC3)nc(N4CCOCC4)n2)cnc1N	C18H21F3N6O2	410.394	BKM120 (NVP-BKM120, Buparlisib)
Selleck Chemicals	S2067	c1cc(C=CC(=O)O)ccc1Cn2ccnc2.Cl	C13H13ClN2O2	264.708	Ozagrel HCl
Selleck Chemicals	S2262	c1c(O)cc2c(C(=O)C=C(c3ccc(O)cc3)O2)c1O	C15H10O5	270.237	Apigenin
Selleck Chemicals	S2098	c(C(C)(C)CCC1(C)C)(cc(C)c(C(=C)c2ccc(C(O)=O)cc2)c3)c13	C24H28O2	348.478	Bexarotene
Selleck Chemicals	S2270	[C@H]1(CO)O[C@]2([H])[C@]([H])(OC(=O)c3c2c(O)c(OC)c(O)c3)[C@@H](O)[C@@H]1O	C14H16O9	328.271	Bergenin
Selleck Chemicals	S2121	c1(c(CC(O)=O)n2c(CC(C)(C)C2)c1c3ccccc3)c4ccc(Cl)cc4	C23H22ClNO2	379.879	Licofelone
Selleck Chemicals	S2290	C1[C@H](C)[C@@]2([H])[C@](OO3)([C@]4([H])O[C@@]3(C)CC2)[C@]([H])([C@@H](C)[C@H](O)O4)C1	C15H24O5	284.348	Dihydroartemisinin (DHA)
Selleck Chemicals	S1548	c1([C@H]2[C@@H](O)[C@@H](O)[C@H](O)[C@@H](CO)O2)cc(Cc3ccc(OCC)cc3)c(Cl)cc1	C21H25ClO6	408.873	Dapagliflozin
Selleck Chemicals	S1714	N(C(=O)N([C@H]1C(F)(F)[C@H](O)[C@@H](CO)O1)C=C2)=C2N	C9H11F2N3O4	263.198	Gemcitabine
Selleck Chemicals	S1565	c(OC(F)(F)O1)(ccc(C2(CC2)C(=O)Nc3ccc(C)c(c4cc(C(=O)O)ccc4)n3)c5)c15	C24H18F2N2O5	452.407	VX-809 (Lumacaftor)
Selleck Chemicals	S1730	c1cc(Cl)c(S(=O)(=O)N)cc1C(=O)NN2C(C)Cc3c2cccc3	C16H16ClN3O3S	365.835	Indapamide
Selleck Chemicals	S1575	O=C1[C@H](NC(=O)C(C)(C)C(=O)NCC(F)(F)C(F)(F)F)c2c(cccc2)c3c(cccc3)N1	C22H20F5N3O3	469.405	RO4929097

Selleck Chemicals	S1739	n1c(c2nc3c(cccc3)[nH]2)csc1	C10H7N3S	201.248	Thiabendazole
Selleck Chemicals	S1603	c1(C(=O)O)c(NCc2occc2)cc(Cl)c(S(=O)(=O)N)c1	C12H11ClN2O5S	330.744	Furosemide
Selleck Chemicals	S1767	C1C(C)=C(C=CC(C)=CC=CC(C)=CC=CC=C(C)C=CC=C(C)C=CC(C(C)(C)CCC2)=C2C)C(C)(C)CC1	C40H56	536.873	Beta Carotene
Selleck Chemicals	S1622	C1=C[C@@]2(C)C(CC[C@]3([H])[C@@]2([H])C(=O)C[C@@]4(C)[C@@]3([H])CC[C@@]4(O)C(=O)CO)=CC1=O	C21H26O5	358.428	Prednisone
Selleck Chemicals	S1790	[C@@H]1(O)[C@@H](C)[C@@H](O)[C@H](C)C=CC=C(C)C(=O)Nc2c(O)c(c(c(C(=O)[C@]3(C)OC=C[C@H](OC)[C@@H](C)[C@@H](OC(=O)C)[C@@H]1C)c4O3)c5c2n6c(cc(C)cc6)n5)c(O)c4C	C43H51N3O11	785.879	Rifaximin
Selleck Chemicals	S1633	c(n(C)cc1Cc2c(OC)cc(C(NS(=O)(=O)c3c(C)cccc3)=O)cc2)(ccc(NC(=O)OC4CCCC4)c5)c15	C31H33N3O6S	575.675	Zafirlukast
Selleck Chemicals	S1811	c1(Cl)nc(C(=O)NC(=N)N)c(N)nc1N.Cl	C6H9Cl2N7O	266.088	Amiloride HCl
Selleck Chemicals	S1646	c1ccc(C(=O)c2n3c(C(C(O)=O)CC3)cc2)cc1.OCC(N)(CO)CO	C19H24N2O6	376.404	Ketorolac
Selleck Chemicals	S1836	c1(S(=O)CCC)cc2c(nc(NC(OC)=O)[nH]2)cc1	C12H15N3O3S	281.331	Albendazole Oxide
Selleck Chemicals	S1665	c1cc2c(CC[C@]3([H])[C@@]2([H])CC[C@@]4(C)[C@@]3([H])CCC4=O)cc1O	C18H22O2	270.366	Estrone
Selleck Chemicals	S1859	c1(O)ccc(C(CC)=C(CC)c2ccc(O)cc2)cc1	C18H20O2	268.35	Diethylstilbestrol
Selleck Chemicals	S1677	c1cc([C@@H](O)[C@@H](CO)NC(C(Cl)Cl)=O)ccc1[N+]([O-])=O	C11H12Cl2N2O5	323.129	Chloramphenicol
Selleck Chemicals	S1889	c1c(O)c2c(C(=O)c3c(c(NCCNCCO)ccc3NCCNCCO)C2=O)c(O)c1	C22H28N4O6	444.481	Mitoxantrone
Selleck Chemicals	S1702	[C@H]1(CO)O[C@@H](n2cnc3c2N=CNC3=O)CC1	C10H12N4O3	236.227	Didanosine
Selleck Chemicals	S1907	n1(c(C)ncc1[N+]([O-])=O)CCO	C6H9N3O3	171.154	Metronidazole
Selleck Chemicals	S1928	c1c(CC=C)cc(C(NCCO)=O)c(O)c1OC	C13H17NO4	251.278	Alibendol
Selleck Chemicals	S2151	c1(NC(=O)c2cccc(c3ccc(OC(F)(F)F)cc3)c2C)cnc(N4C[C@@H](C)O[C@@H](C)C4)cc1	C26H26F3N3O3	485.498	LDE225 (NVP-LDE225,Erismodegib)
Selleck Chemicals	S1957	c1cc(S(=O)(=O)Nc2nnc(C)s2)ccc1N	C9H10N4O2S2	270.331	Sulfamethizole
Selleck Chemicals	S2170	c1c(CN(CC)CC)cc2c(cc(COC(=O)Nc3ccc(C(=O)NO)cc3)cc2)c1.O.Cl	C24H30ClN3O5	475.965	Givinostat (ITF2357)
Selleck Chemicals	S1979	c1(OCCN(CC)CC)c(I)cc(C(c2c3c(cccc3)oc2CCCC)=O)cc1I.Cl	C25H30ClI2NO3	681.773	Amiodarone HCl
Selleck Chemicals	S2186	c1(C)nc(c2c(c3ccc4c(OCO4)c3)nc(C(C)(C)C)[nH]2)ccc1	C20H21N3O2	335.4	SB505124
Selleck Chemicals	S2007	c1c(C=C2C(C)=C(CC(O)=O)c3c2ccc(F)c3)ccc(S(=O)C)c1	C20H17FO3S	356.411	Sulindac
Selleck Chemicals	S2205	c1c(Nc2c3c(cc(OCCOC)c(OCCO)c3)ncn2)cccc1C#C.Cl	C21H22ClN3O4	415.87	OSI-420
Selleck Chemicals	S2029	C1=CC(=O)NC(=O)N1[C@H]2[C@@H](O)[C@H](O)[C@@H](CO)O2	C9H12N2O6	244.201	Uridine
Selleck Chemicals	S2220	n1c(c2ccc(OCCN(C)C)cc2)[nH]c(c3cc4c(C(=NO)CC4)cc3)c1c5ccncc5	C27H27N5O2	453.536	SB590885
Selleck Chemicals	S2042	[C@@H]1(C2)[C@@H]2[C@@]3(C)C(C(Cl)=C[C@]4([H])[C@@]3([H])CC[C@@]5(C)[C@@]4([H])CC[C@@]5(OC(=O)C)C(C)=O)=CC1=O	C24H29ClO4	416.938	Cyproterone Acetate
Selleck Chemicals	S2231	n1nc(OCc2ccnc(C(NC)=O)c2)c3c(cco3)c1Nc4ccc(Cl)cc4	C20H16ClN5O3	409.826	Telatinib
Selleck Chemicals	S2059	c(cc(OC)c(OC)c1)(nc(N2CCN(C(=O)C3OCCC3)CC2)nc4N)c14.Cl.O.O	C19H30ClN5O6	459.924	Terazosin HCl
Selleck Chemicals	S2248	c1cc2c(c3c(cc(C(O)=O)cc3)nc2Nc4cccc(Cl)c4)cn1	C19H12ClN3O2	349.77	CX-4945 (Silmitasertib)
Selleck Chemicals	S2074	c1cc2c(c(C(OCC)=O)c(C)n2C)cc1O	C13H15NO3	233.263	Mecarbinate
Selleck Chemicals	S2263	[C@H]1(CO)O[C@@H](Oc2ccc(O)cc2)[C@H](O)[C@@H](O)[C@@H]1O	C12H16O7	272.251	Arbutin
Selleck Chemicals	S2101	c1(OC(CCCCCNC(=N)N)=O)ccc(C(=O)OCC)cc1.O=S(C)(O)=O	C17H27N3O7S	417.477	Gabexate Mesylate
Selleck Chemicals	S2271	c1cc2c(c[n+]3c(c4c(cc5c(OCO5)c4)CC3)c2)c(OC)c1OC.[Cl-]	C20H18ClNO4	371.814	Berberine HCl

Selleck Chemicals	S2125	c1cc2c(CC[C@]3([H])[C@@]2([H])CC[C@@]4(C)[C@@]3([H])CC[C@]4(O)C#C)cc1OC	C21H26O2	310.43	Mestranol
Selleck Chemicals	S2292	[C@@H]1(O)[C@H](O)[C@H](C)O[C@@H](OC[C@H]2O[C@@H](Oc3cc(O)c4c(OC(c5ccc(OC)c(O)c5)=CC4=O)c3)[C@H](O)[C@@H](O)[C@@H]2O)[C@@H]1O	C28H32O15	608.545	Diosmin
Selleck Chemicals	S1549	c1cc2c(CC[C@]([H])([C@H](O)CNC[C@@H](O)[C@@]3([H])Oc4c(cc(F)cc4)CC3)O2)cc1F.Cl	C22H26ClF2NO4	441.896	Nebivolol
Selleck Chemicals	S1715	c1(S(=O)(=O)NC(=O)NC2CCCCC2)ccc(CCNC(=O)c3ncc(C)nc3)cc1	C21H27N5O4S	445.535	Glipizide
Selleck Chemicals	S1567	c1c(N)c2c(C(=O)N(C3CCC(=O)NC3=O)C2=O)cc1	C13H11N3O4	273.244	Pomalidomide
Selleck Chemicals	S1732	c1cc(C(C(Cl)Cl)c2ccccc2Cl)ccc1Cl	C14H10Cl4	320.041	Mitotane
Selleck Chemicals	S1576	c1(ncccc1)NS(=O)(=O)c2ccc(N=Nc3ccc(O)c(C(O)=O)c3)cc2	C18H14N4O5S	398.393	Sulfasalazine
Selleck Chemicals	S1741	[C@@H]1(C)[C@@H](OC)C=CO[C@]2(C)C(=O)c(c3c4C(=O)C(=C(NC56CCN(CC(C)C)CC5)C3=N6)NC(=O)C(C)=CC=C[C@H](C)[C@H](O)[C@@H](C)[C@@H](O)[C@@H](C)[C@@H]1OC(C)=O)c(O2)c(C)c4O	C46H62N4O11	847.005	Rifabutin
Selleck Chemicals	S1605	[C@]([H])([C@@](NC(=O)C(=NO)c1csc(N)n1)([H])C2=O)(SCC(C=C)=C3C(=O)O)N23	C14H13N5O5S2	395.414	Cefdinir
Selleck Chemicals	S1768	N(C(=O)[C@@H]1NC(C(c2nc(N)sc2)=NOC)=O)(C(C(OCOC(=O)C(C)(C)C)=O)=C(C=Cc3c(C)ncs3)CS4)[C@@]14[H]	C25H28N6O7S3	620.721	Cefditoren Pivoxil
Selleck Chemicals	S1623	C(=O)(N[C@@]([H])(CS)C(=O)O)C	C5H9NO3S	163.195	Acetylcysteine
Selleck Chemicals	S1792	C1[C@@H](OC(C(C)(C)CC)=O)[C@@]2([H])C(C=C[C@H](C)[C@H]2CC[C@]3([H])C[C@@H](O)CC(=O)O3)=C[C@@H]1C	C25H38O5	418.566	Simvastatin
Selleck Chemicals	S1635	C1[C@@](O)(C)[C@H](O[C@H]2OC(C)C[C@@H](N(C)C)[C@H]2O)[C@@H](C)[C@H](O[C@H]3O[C@H](C)[C@@H](O)[C@](C)(OC)C3)[C@@H](C)C(=O)O[C@H](CC)[C@@](C)(O)[C@H](O)[C@@H](C)C(=O)[C@@H	C37H67NO13	733.927	Erythromycin
Selleck Chemicals	S1816	c1nc(C(c2ccc(Cl)cc2)CCN(C)C)ccc1.C(=O)(O)C=CC(=O)O	C20H23ClN2O4	390.861	Chlorpheniramine Maleate
Selleck Chemicals	S1647	O1[C@H](n2cnc3c2ncnc3N)[C@H](O)[C@H](O)[C@H]1CO	C10H13N5O4	267.241	Adenosine
Selleck Chemicals	S1839	c1c(Cl)c(O)c2c(cccn2)c1Cl	C9H5Cl2NO	214.048	Chloroxine
Selleck Chemicals	S1666	C1(=O)N=C(N)C(F)=CN1	C4H4FN3O	129.092	Flucytosine
Selleck Chemicals	S1866	c1cccc(C(OCCN(C)C)c2ccccc2)c1.Cl	C17H22ClNO	291.816	Diphenhydramine HCl
Selleck Chemicals	S1679	c1(F)cc([C@H](C)C(=O)O)ccc1c2ccccc2	C15H13FO2	244.261	Flurbiprofen
Selleck Chemicals	S1891	c1(O)cc(C[C@@](NN)(C)C(=O)O)ccc1O	C10H14N2O4	226.229	Carbidopa
Selleck Chemicals	S1704	N(C(=O)N([C@@H]1CS[C@H](CO)O1)C=C2F)=C2N	C8H10FN3O3S	247.247	Emtricitabine
Selleck Chemicals	S1908	c1([N+]([O-])=O)ccc(NC(=O)C(C)C)cc1C(F)(F)F	C11H11F3N2O3	276.212	Flutamide
Selleck Chemicals	S1929	c1c(Cl)cc(c2nc(N)nc(N)n2)c(Cl)c1	C9H7Cl2N5	256.091	Irsogladine
Selleck Chemicals	S2154	c1(C(N)=NC(=O)OCCCCCC)ccc(NCc2nc3c(ccc(C(=O)N(c4ncccc4)CCC(=O)OCC)c3)n2C)cc1	C34H41N7O5	627.733	Dabigatran Etexilate
Selleck Chemicals	S1962	N(S(=O)(=O)c1ccc(N)cc1)c2cc(OC)nc(OC)n2	C12H14N4O4S	310.329	Sulphadimethoxine
Selleck Chemicals	S2177	c1(Cl)cc(S(=O)(=O)N)cc(S(=O)(=O)N)c1Cl	C6H6Cl2N2O4S2	305.159	Dichlorphenamide
Selleck Chemicals	S1987	C1=C[C@@]2(C)C(CC[C@]3([H])[C@@]2(Cl)[C@H](O)C[C@@]4(C)[C@@]3([H])C[C@@H](C)[C@@]4(OC(c5occc5)=O)C(=O)CCl)=CC1=O	C27H30Cl2O6	521.429	Mometasone furoate
Selleck Chemicals	S2187	c1c(C(C)C)c(CC(=O)NS(=O)(=O)Oc2c(C(C)C)cccc2C(C)C)c(C(C)C)cc1C(C)C	C29H43NO4S	501.721	Avasimibe
Selleck Chemicals	S2012	n1(Cc2ccc(OC)cc2)c(c3cc1)cc(C(NO)=O)cc3	C17H16N2O3	296.321	PCI-34051
Selleck Chemicals	S2207	C1(Cn2c3c(c(N)ncn3)cn2)=Nc4c(c(C)ccc4)C(=O)N1c5ccccc5C	C22H19N7O	397.433	PIK-293
Selleck Chemicals	S2030	c1(F)ccc(C(c2ccc(F)cc2)N3CCN(CC=Cc4ccccc4)CC3)cc1.Cl.Cl	C26H28Cl2F2N2	477.417	Flunarizine 2HCl
Selleck Chemicals	S2221	S(=O)(C)(O)=O.c1cc(C(=O)Nc2ccc(C3(C#N)CCCC3)cc2)c(NCc4ccncc4)nc1	C25H27N5O4S	493.578	Apatinib

Selleck Chemicals	S2043	Cl.C1(C)(CC(C)(CC2C1)CC3(N)C2)C3	C12H22ClN	215.763	Memantine HCl
Selleck Chemicals	S2232	c1cc2c(NC(=O)N(CCN3CCC(C(=O)c4ccc(F)cc4)CC3)C2=O)cc1	C22H22FN3O3	395.427	Ketanserin
Selleck Chemicals	S2060	c1(Br)cc(CN(C2CCCCC2)C)c(N)c(Br)c1.Cl	C14H21Br2ClN2	412.591	Bromhexine HCl
Selleck Chemicals	S2252	C1(C(C)=O)=C(O)[C@]2(C)C(Oc3c2c(O)c(C)c(O)c3C(=O)C)=CC1=O	C18H16O7	344.315	(+)-Usniacin
Selleck Chemicals	S2077	[Ca+2].n1(c(c2ccc(F)cc2)c(c3ccccc3)c(C(Nc4ccccc4)=O)c1C(C)C)CC[C@@H](O)C[C@@H](O)CC(=O)[O-].n5(c(c6ccc(F)cc6)c(c7ccccc7)c(C(Nc8ccccc8)=O)c5C(C)C)CC[C@@H](O)C[C@@H](O)CC(=O)[O-]	C66H68CaF2N4O10	1155.342	Atorvastatin Calcium
Selleck Chemicals	S2264	[C@]([H])(CC1)([C@@]([H])(C)CC2)[C@@]([C@]([H])(O[C@@]13C)O[C@H](OC)[C@]4(C)[H])(OO3)[C@@]24[H]	C16H26O5	298.375	Artemether
Selleck Chemicals	S2113	c1(OC)cc2c([C@@H](Cc3cc(OC)c(OC)cc3)[N@@+](C)(CCC(=O)OCCCCCOC(=O)CC[N@@+]4(C)CCc(c5[C@H]4Cc6cc(OC)c(OC)cc6)cc(OC)c(OC)c5)CC2)cc1OC.c7c(S(=O)(=O)[O-])cccc7.c8c(S(=O)(=O)[O-])cccc8	C65H82N2O18S2	1243.479	Cisatracurium Besylate
Selleck Chemicals	S2276	O1[C@]2([H])C3([C@@H](O)C(=O)O2)[C@@](CC(=O)O4)([C@]4([H])C[C@]3(O)C(C)(C)C)C1=O	C15H18O8	326.299	Bilobalide
Selleck Chemicals	S2126	c1(OCC(O)CN2CCN(c3c(OC)cccc3)CC2)cccc4c1cccc4	C24H28N2O3	392.491	Naftopidil
Selleck Chemicals	S2295	c(c(O)cc(O)c1)(C(=O)c2c(cc(C)cc2O)C3=O)c13	C15H10O5	270.237	Emodin
Selleck Chemicals	S1550	c1c(C2=NNC(=O)CC2C)ccc3c1nc(c4ccc(OC)cc4)[nH]3	C19H18N4O2	334.372	Pimobendan
Selleck Chemicals	S1718	c(ncn1CCOCP(OCOC(=O)C(C)(C)C)(=O)OCOC(=O)C(C)(C)C)(c(N)ncn2)c12	C20H32N5O8P	501.471	Adefovir Dipivoxil
Selleck Chemicals	S1568	c1c(C(=O)NOCC(O)CO)c(Nc2c(F)cc(I)cc2)c(F)c(F)c1Br	C16H13BrF3IN2O4	561.089	PD318088
Selleck Chemicals	S1733	C1=C[C@@]2(C)C([C@@H](C)C[C@]3([H])[C@@]2([H])[C@H](O)C[C@@]4(C)[C@@]3([H])CC[C@@]4(O)C(=O)CO)=CC1=O	C22H30O5	374.471	Methylprednisolone
Selleck Chemicals	S1577	c(ccc(OC)c1)(cc(c2[nH]c(c3ccc(S(=O)C)cc3)nc2c4ccncc4)cc5)c15	C26H21N3O2S	439.529	Tie2 kinase inhibitor
Selleck Chemicals	S1742	N1(c2c(cccn2)C(=O)Nc3c1nccc3C)C4CC4	C15H14N4O	266.298	Nevirapine
Selleck Chemicals	S1606	c1cc(C(n2ccnc2)(c3ccccc3)c4ccccc4Cl)ccc1	C22H17ClN2	344.837	Clotrimazole
Selleck Chemicals	S1770	c1nc(NS(=O)(=O)c2ccc(N)cc2)ncc1	C10H10N4O2S	250.277	Sulfadiazine
Selleck Chemicals	S1625	c1cc2c(CC[C@]3([H])[C@@]2([H])CC[C@@]4(C)[C@@]3([H])CC[C@@]4(C#C)O)cc1O	C20H24O2	296.403	Ethinyl Estradiol
Selleck Chemicals	S1793	c1c(CC[C@H](N[C@@H](C)C(=O)N2[C@@H]3[C@@H](CCC3)C[C@@H]2C(=O)O)C(OCC)=O)cccc1	C23H32N2O5	416.511	Ramipril
Selleck Chemicals	S1636	[C@@H]1(C)OC(=O)C[C@H](O)C[C@H](O)CC[C@@H](O)[C@H](O)C[C@H](O)C[C@](O)(O[C@H]2C[C@@H](O[C@@H]3O[C@H](C)[C@@H](O)[C@H](N)[C@@H]3O)C=CC=CC=CC=CC=CC=CC=C[C@H](C)[C@@H](O)[C@H]1C)C[C@H](O)[C@@H]2C(=O)O	C47H73NO17	924.079	Amphotericin B
Selleck Chemicals	S1825	S1CCC(NC(=O)CSCC(=O)O)C1=O	C8H11NO4S2	249.307	Erdosteine
Selleck Chemicals	S1651	N1C(=O)N([C@H]2O[C@@H](CO)[C@H](O)C2)C=C(C)C1=O	C10H14N2O5	242.229	Telbivudine
Selleck Chemicals	S1840	C1CC(NC(=O)N(N=O)CCCl)CCC1	C9H16ClN3O2	233.695	Lomustine
Selleck Chemicals	S1667	c1(Cl)cc2c(S(=O)(=O)NC(C(Cl)Cl)N2)cc1S(N)(=O)=O	C8H8Cl3N3O4S2	380.656	Trichlormethiazide
Selleck Chemicals	S1869	c1c(OCC[C@H](N(C)C)c2ccccc2)c3c(cccc3)cc1.Cl	C21H24ClNO	341.874	Dapoxetine HCl
Selleck Chemicals	S1680	CCN(CC)C(=S)SSC(=S)N(CC)CC	C10H20N2S4	296.539	Disulfiram
Selleck Chemicals	S1895	n1c(N2CCCCC2)c3c(c(N4CCCCC4)nc(N(CCO)CCO)n3)nc1N(CCO)CCO	C24H40N8O4	504.626	Dipyridamole
Selleck Chemicals	S1705	C1C[C@@]2(C)C(CC[C@]3([H])[C@@]2([H])CC[C@@]4(C)[C@@]3([H])CC[C@H]4C(=O)C)=CC1=O	C21H30O2	314.462	Progesterone
Selleck Chemicals	S1909	c(c(c1ccc(F)cc1)c(C=C[C@@H](O)C[C@@H](O)CC(=O)[O-])n2C(C)C)(cccc3)c23.[Na+]	C24H25FNNaO4	433.448	Fluvastatin Sodium

Selleck Chemicals	S1934	[C@@H]1(C)OC(=O)C[C@H](O)C[C@H](O)C[C@H](O)CC[C@H](O)[C@H](O)C[C@]2(O)O[C@H]([C@H](C(O)=O)[C@@H](O)C2)C[C@@H](O[C@H]3O[C@@H](C)[C@H](O)[C@@H](N)[C@H]3O)C=CC=CC=CC=CCC=CC=C[C@H](C)[C@@H](O)[C@H]1C	C47H75NO17	926.095	Nystatin (Fungicidin)
Selleck Chemicals	S2155	c1cc([C@]2(C(=O)Nc3sc(SCCN4CCCC4)cn3)[C@@H](C5CCCCC5)C2)ccc1S(=O)(=O)C6CC6	C28H37N3O3S3	559.807	LY2608204
Selleck Chemicals	S1964	C1C(C(C)N)(CC(CC2C3)CC13)C2	C12H21N	179.302	Rimantadine
Selleck Chemicals	S2178	c(n1c(c2ccc(CN(C)C)cc2)n3)(c3ccc4)c4C(=O)NCC1	C19H20N4O	320.388	AG-14361
Selleck Chemicals	S1988	C(C(=O)NC(=S)N1)=C1CCC	C7H10N2OS	170.232	Propylthiouracil
Selleck Chemicals	S2192	c1(OC2CCN(CC(=O)NC)CC2)cc3c(ncnc3Nc4cccc(Cl)c4F)cc1OC	C23H25ClFN5O3	473.928	AZD8931 (Sapitinib)
Selleck Chemicals	S2014	C(c1cnc2c(cn[nH]2)c1OCCCC)(c3c(F)cc(C)cc3F)=O	C18H17F2N3O2	345.343	BMS-265246
Selleck Chemicals	S2208	C1C[C@@]2(C)C(CC[C@]3([H])[C@@]2([H])CC[C@@]4(C)[C@@]3([H])CCC4=O)=C(O)C1=O	C19H26O3	302.408	Formestane
Selleck Chemicals	S2031	C(Cn1cncc1)(c2c(Cl)cc(Cl)cc2)OCc3ccc(Sc4ccccc4)cc3.[N+](O)(=O)[O-]	C24H21Cl2N3O4S	518.412	Fenticonazole Nitrate
Selleck Chemicals	S2224	c1(onc(C(=O)N)n1)[C@@H](CC(=O)NO)CCCC2CCCCC2	C15H24N4O4	324.375	UK 383367
Selleck Chemicals	S2044	C1(c2c(cccc2)C=Cc3c1cccc3)=C4CCN(C)CC4.Cl	C21H22ClN	323.859	Cyproheptadine HCl
Selleck Chemicals	S2235	[C@H]1(CC)N(C(C)C)c2c(cnc(Nc3c(OC)cc(C(=O)N[C@H]4CC[C@H](N5CCN(CC6CC6)CC5)CC4)cc3)n2)N(C)C1=O	C34H50N8O3	618.813	Volasertib (BI 6727)
Selleck Chemicals	S2061	C1[C@@H](OC([C@@H](C)CC)=O)[C@@]2([H])C(C=C[C@H](C)[C@H]2CC[C@@H]3C[C@@H](O)CC(=O)O3)=C[C@@H]1C	C24H36O5	404.54	Lovastatin
Selleck Chemicals	S2253	c1c2c(c(CCCC(=O)O)c[nH]2)ccc1	C12H13NO2	203.237	3-Indolebutyric acid (IBA)
Selleck Chemicals	S2078	NS(=O)(=O)NC(=N)CCSCc1nc(NC(=N)N)sc1	C8H15N7O2S3	337.445	Famotidine
Selleck Chemicals	S2265	[C@]([H])(CC1)([C@@]([H])(C)CC2)[C@@]([C@]([H])(O[C@@]13C)O[C@H](OC(CCC(=O)O)=O)[C@]4(C)[H])(OO3)[C@@]24[H]	C19H28O8	384.421	Artesunate
Selleck Chemicals	S2114	c1(NS(=O)(C)=O)cc2c(oc(CCCC)c2C(c3ccc(OCCCN(CCCC)CCCC)cc3)=O)cc1.Cl	C31H45ClN2O5S	593.217	Dronedarone HCl
Selleck Chemicals	S2277	c1cc(C=CC(=O)O)cc(O)c1O	C9H8O4	180.157	Caffeic Acid
Selleck Chemicals	S2127	c1(OC)ccc([C@@H]2CC(=O)NC2)cc1OC3CCCC3	C16H21NO3	275.343	S- (+)-Rolipram
Selleck Chemicals	S2296	C1C[C@@]2(C)[C@@]([H])(CC[C@]3(C)[C@@]2([H])C(=O)C=C4[C@@]3(C)CC[C@]5(C)[C@]4([H])C[C@](C(=O)O)(C)CC5)C(C)(C)[C@@]1([H])	C30H46O4	470.684	Enoxolone
Selleck Chemicals	S1555	c1(CO)cc(c2ccc3c(nc(N4CCOC[C@@H]4C)nc3N5CCOC[C@@H]5C)n2)ccc1OC	C25H31N5O4	465.545	AZD8055
Selleck Chemicals	S1719	[C@H]1(CO)O[C@@H](N2C=CC(N)=NC2=O)CC1	C9H13N3O3	211.218	Zalcitabine
Selleck Chemicals	S1569	C1Sc2c(cc(C#Cc3ccc(C(OCC)=O)cn3)cc2)C(C)(C)C1	C21H21NO2S	351.462	Tazarotene
Selleck Chemicals	S1734	C1(C(Nc2sc(C)cn2)=O)=C(O)c3c(cccc3)S(=O)(=O)N1C	C14H13N3O4S2	351.401	Meloxicam
Selleck Chemicals	S1582	c1c2c(ccnc2)c(S(=O)(=O)NCCNCC=Cc3ccc(Br)cc3)cc1.Cl.Cl	C20H22BrCl2N3O2S	519.283	H 89 2HCl
Selleck Chemicals	S1744	c1cc(C(O)=O)cnc1	C6H5NO2	123.109	Nicotinic Acid
Selleck Chemicals	S1607	c1c2c(c(CCN(C)C)c[nH]2)cc(Cn3ncnc3)c1.c4cccc(C(=O)O)c4	C22H25N5O2	391.466	Rizatriptan Benzoate
Selleck Chemicals	S1773	C(C(=O)N)(C(=O)[C@]1(O)[C@@]([H])([C@@H](O)[C@@]2([H])C(=C(O)c3c(cccc3O)[C@@]2(C)O)C1=O)[C@@H]4N(C)C)=C4O	C22H24N2O9	460.434	Oxytetracycline (Terramycin)
Selleck Chemicals	S1626	c1cc2c(ccc([C@H](C)C(=O)[O-])c2)cc1OC.[Na+]	C14H13NaO3	252.241	Naproxen
Selleck Chemicals	S1794	c1cc(C(=O)c2ccc(OC(C)(C)C(=O)OC(C)C)cc2)ccc1Cl	C20H21ClO4	360.831	Fenofibrate
Selleck Chemicals	S1639	c1cc(S(=O)(=O)N(CC(C)C)C[C@@H](O)[C@H](Cc2ccccc2)NC(=O)O[C@H]3CCOC3)ccc1N	C25H35N3O6S	505.627	Amprenavir
Selleck Chemicals	S1828	c1(N)cc(C(=O)OCCN(CC)CC)ccc1OCCC.Cl	C16H27ClN2O3	330.85	Proparacaine HCl
Selleck Chemicals	S1652	c1cc(O)ccc1OCc2ccccc2	C13H12O2	200.233	Monobenzone

Selleck Chemicals	S1843	C1C[C@@]2(C)[C@]([H])(C[C@@H](O)[C@]3([H])[C@@]2([H])CC[C@@]4(C)[C@@]3([H])CC[C@]4([C@H](C)CCC(=O)O)[H])C[C@@H]1O	C24H40O4	392.572	Chenodeoxycholic Acid
Selleck Chemicals	S1669	C1=C[C@@]2(C)C(CC[C@]3([H])[C@@]2([H])[C@H](O)C[C@@]4(C)[C@@]3([H])CC[C@@]4(OC(=O)OCC)C(=O)OCCl)=CC1=O	C24H31ClO7	466.952	Loteprednol etabonate
Selleck Chemicals	S1876	N1C(N)=Nc2c(ncn2COCCOC(=O)[C@@H](N)C(C)C)C1=O.Cl	C13H21ClN6O4	360.797	Valaciclovir HCl
Selleck Chemicals	S1685	c1(N)ccc(S(=O)(=O)N)cc1	C6H8N2O2S	172.205	Sulfanilamide
Selleck Chemicals	S1896	N(O)C(=O)N	CH4N2O2	76.055	Hydroxyurea
Selleck Chemicals	S1706	N(C(=O)N([C@@H]1CS[C@H](CO)O1)C=C2)=C2N	C8H11N3O3S	229.256	Lamivudine
Selleck Chemicals	S1910	c1c(Cl)ccc(C(Cn2cncc2)OCc3c(Cl)scc3)c1Cl	C16H13Cl3N2OS	387.711	Tioconazole
Selleck Chemicals	S1937	c1c(C(=O)NN)ccnc1	C6H7N3O	137.139	Isoniazid
Selleck Chemicals	S2156	c1nc(C(=O)Nc2cc([C@]3(COC4)[C@@]4([H])CSC(N)=N3)c(F)cc2)ccc1F	C18H16F2N4O2S	390.407	LY2886721
Selleck Chemicals	S1965	C1NC(=O)C(c2ccccc2)(CC)C(=O)N1	C12H14N2O2	218.252	Primidone
Selleck Chemicals	S2179	c1c(CN2CCOCC2)c3n(c(Cc4c(F)cc(Cl)cc4)c(C)n3)nc1Nc5cc(C)n[nH]5	C23H25ClFN7O	469.942	LY2784544
Selleck Chemicals	S1992	C1=C[C@@]2(C)C([C@@H](F)C[C@]3([H])[C@@]2(F)[C@H](O)C[C@@]4(C)[C@@]3([H])C[C@@H](C)[C@@]4(OC(=O)CC)C(=O)SCF)=CC1=O	C25H31F3O5S	500.571	Fluticasone propionate
Selleck Chemicals	S2193	C1CN(Cc2ccc3c(n(c4sc(C(N)=O)c(O[C@@H](c5c(C(F)(F)F)cccc5)C)c4)cn3)c2)CCN1C	C27H28F3N5O2S	543.604	GSK461364
Selleck Chemicals	S2015	c1c(NC(=O)CC[S+](C)C)ccc(OCC(COCC)O)c1.c2c(S([O-])(=O)=O)ccc(C)c2	C23H33NO7S2	499.641	Suplatast Tosylate
Selleck Chemicals	S2214	c1c(F)c(Nc2n[nH]c(C)c2)nc(N[C@@H](C)c3ccc(F)cc3)c1C#N	C18H16F2N6	354.357	AZ 960
Selleck Chemicals	S2032	c1(ccc(Cl)cc1)C(=O)NC(C(O)=O)CC(c2c(cccc2)NC3=O)=C3	C19H15ClN2O4	370.786	Rebamipide
Selleck Chemicals	S2225	c1cc(S(=O)(=O)N[C@@H](CCCNC(N)=N)C(=O)OC)ccc1C	C14H22N4O4S	342.414	TAME
Selleck Chemicals	S2045	N1C(=O)N([C@H]2[C@@H](O)[C@H](O)[C@@H](C)O2)C=C(F)C1=O	C9H11FN2O5	246.192	Doxifluridine
Selleck Chemicals	S2238	c1cc(COc2cc3c(c4c(cc(C(O)C)cc4)C(=O)O3)cc2OC)ccc1OC	C24H22O6	406.428	Palomid 529 (P529)
Selleck Chemicals	S2062	C(O)(=O)CNC(=O)C(S)C	C5H9NO3S	163.195	Tiopronin
Selleck Chemicals	S2256	c1c2c(C(C)=CC(=O)O2)ccc1O	C10H8O3	176.169	4-Methylumbelliferone (4-MU)
Selleck Chemicals	S2086	c1c(OC)c(OC)cc2c1C[C@H]2CN(C)CCCN3C(=O)Cc4c(cc(OC)c(OC)c4)CC3.Cl	C27H37ClN2O5	505.046	Ivabradine HCl
Selleck Chemicals	S2266	[C@@H]1(O)C[C@@]2(C)[C@@]([H])(CC[C@]3(C)[C@@]2([H])CC=C4[C@@]3(C)CC[C@]5(C(=O)O)[C@]4([H])[C@H](C)[C@H](C)CC5)[C@](C)(CO)[C@H]1O	C30H48O5	488.699	Asiatic Acid
Selleck Chemicals	S2116	C(=O)(c1ccccc1c2ccccc2)Nc3ccc(C(=O)N4CCc5c([nH]c(C)n5)c6c4cccc6)cc3.Cl	C32H27ClN4O2	535.035	Conivaptan HCl
Selleck Chemicals	S2281	c1c(O)cc2c(C(=O)C=C(c3ccccc3)O2)c1O	C15H10O4	254.238	Chrysin
Selleck Chemicals	S2128	c1(c2ccc(O)cc2)c(C)c(c3n1Cc4ccc(OCCN5CCCCCC5)cc4)cc(O)cc3.Cl	C30H35ClN2O3	507.063	Bazedoxifene HCl
Selleck Chemicals	S2298	c(C(=O)C(O)=C(c1ccc(O)c(O)c1)O2)(ccc(O)c3)c23	C15H10O6	286.236	Fisetin
Selleck Chemicals	S1556	c1cc(S(=O)(Nc2scnn2)=O)ccc1CCCCCCCCCCCC	C20H31N3O2S2	409.609	PHT-427
Selleck Chemicals	S1721	n1c(Sc2n(C)cnc2[N+]([O-])=O)c3c(nc[nH]3)nc1	C9H7N7O2S	277.263	Azathioprine
Selleck Chemicals	S1570	c1c2c(Sc3c(cccc3C(OC(N4CCOCC4)=CC5=O)=C5)C2)ccc1NC(=O)CN6C[C@@H](C)O[C@@H](C)C6	C30H33N3O5S	547.665	KU-60019
Selleck Chemicals	S1735	SCCS(=O)(=O)[O-].[Na+]	C2H5NaO3S2	164.179	Mesna
Selleck Chemicals	S1590	c1c(O)cc(Oc2c3c([nH]c(c4cc(N)ccc4)c3)ncn2)cc1	C18H14N4O2	318.329	TWS119
Selleck Chemicals	S1756	c1(F)cc2c(N(CC)C=C(C(=O)O)C2=O)nc1N3CCNCC3	C15H17FN4O3	320.319	Enoxacin

Selleck Chemicals	S1610	c1(Cl)cc2c(C(=O)N(c3c(C)cccc3)C(C)N2)cc1S(N)(=O)=O	C16H16ClN3O3S	365.835	Metolazone
Selleck Chemicals	S1777	c1c(C(N)=S)cc(CC)nc1	C8H10N2S	166.243	Ethionamide
Selleck Chemicals	S1627	c1c(OC(=O)C)c(C(=O)Nc2sc([N+]([O-])=O)cn2)ccc1	C12H9N3O5S	307.282	Nitazoxanide
Selleck Chemicals	S1799	c1c(C)c(NC(=O)CN2CCN(CC(O)COc3c(OC)cccc3)CC2)c(C)cc1	C24H33N3O4	427.537	Ranolazine
Selleck Chemicals	S1641	c1(Cl)cc2c(S(=O)(=O)NC=N2)cc1S(=O)(=O)N	C7H6ClN3O4S2	295.723	Chlorothiazide
Selleck Chemicals	S1830	S(=O)(c1ccccc1)c2ccc3c([nH]c(NC(=O)OC)n3)c2	C15H13N3O3S	315.347	Oxfendazole
Selleck Chemicals	S1653	C1C(C)(C)C(C=CC(C)=CC=CC(C)=CC(=O)O)=C(C)CC1	C20H28O2	300.435	Tretinoin
Selleck Chemicals	S1847	c1cc(Cl)ccc1[C@@](C)(c2ccccc2)OCC[C@H]3CCCN3C.OC(=O)C=CC(=O)O	C25H30ClNO5	459.962	Clemastine Fumarate
Selleck Chemicals	S1671	NCCCCCC(=O)O	C6H13NO2	131.173	?6-??-?Aminocaproic acid
Selleck Chemicals	S1878	N1C(=O)c2c(n(COC(CO)CO)cn2)N=C1N	C9H13N5O4	255.231	Ganciclovir
Selleck Chemicals	S1689	C1=C[C@@]2(C)C(CC[C@]3([H])[C@@]2([H])C(=O)C[C@@]4(C)[C@@]3([H])C[C@H](C)[C@]4(C(=O)CO)O)=CC1=O	C22H28O5	372.455	Meprednisone
Selleck Chemicals	S1899	c1cc(C(=O)N)cnc1	C6H6N2O	122.125	Nicotinamide (Vitamin B3)
Selleck Chemicals	S1708	c1(Cl)cc2c(S(=O)(=O)NCN2)cc1S(=O)(=O)N	C7H8ClN3O4S2	297.739	Hydrochlorothiazide
Selleck Chemicals	S1914	C1C[C@@]2(C)C(=CC[C@]3([H])[C@@]2([H])CC[C@@]4(C)[C@@]3([H])CC[C@H]4C(C)=O)C[C@H]1O	C21H32O2	316.478	Pregnenolone
Selleck Chemicals	S1940	c(N1[C@]([H])(C)CO2)(c2c(N3CCN(C)CC3)c(F)c4)c4C(=O)C(C(O)=O)=C1	C18H20FN3O4	361.368	Levofloxacin
Selleck Chemicals	S2158	c1c2c(c(C=Cc3ccc(C(N4CCNCC4)=O)cc3)n[nH]2)ccc1	C20H20N4O	332.399	KW-2449
Selleck Chemicals	S1969	C(C(=O)Nc1c(C)cccc1C)N2CCCC2=O	C14H18N2O2	246.305	Nefiracetam
Selleck Chemicals	S2180	c1c(Cl)c(C(=O)NCC(=O)N[C@@H](CC(C)C)B(O)O)cc(Cl)c1	C14H19BCl2N2O4	361.029	MLN2238
Selleck Chemicals	S2001	c1cc(Cl)c(F)c(Cc2c(OC)cc3c(C(=O)C(C(=O)O)=CN3[C@H](CO)C(C)C)c2)c1	C23H23ClFNO5	447.884	Elvitegravir (GS-9137, JTK-303)
Selleck Chemicals	S2195	n1c(c2ccc(NC(NCC)=O)c(OC)c2)nc(N[C@@H](CCC)c3cnccc3)c(C)c1	C24H30N6O2	434.534	CYT997 (Lexibulin)
Selleck Chemicals	S2021	c1cc(C2N(C)C(=O)CCS2(=O)=O)ccc1Cl	C11H12ClNO3S	273.736	Chlormezanone
Selleck Chemicals	S2215	c1(F)cc(CC(N[C@H](C(=O)N[C@@H](c2ccccc2)C(OC(C)(C)C)=O)C)=O)cc(F)c1	C23H26F2N2O4	432.46	DAPT (GSI-IX)
Selleck Chemicals	S2035	c1cc(C=C(C)C=C2C(=O)N(CC(=O)O)C(=S)S2)ccc1	C15H13NO3S2	319.399	Epalrestat
Selleck Chemicals	S2226	c1c(F)c2c(N=C([C@H](CC)Nc3ncnc4c3nc[nH]4)N(c5ccccc5)C2=O)cc1	C22H18FN7O	415.423	CAL-101 (Idelalisib, GS-1101)
Selleck Chemicals	S2047	N(C(=O)C1=C(O)c2c(cc(Cl)s2)S(=O)(=O)N1C)c3ccccn3	C13H10ClN3O4S2	371.819	Lornoxicam
Selleck Chemicals	S2239	[C@@H]1(CSc2nc(c3ccccc3)c(c4ccccc4)o2)O[C@H](c5ccc(NC(CCCCCCC(=O)NO)=O)cc5)O[C@H](c6ccc(CO)cc6)C1	C41H43N3O7S	721.861	Tubacin
Selleck Chemicals	S2064	N1(CCCC(NC)C1)c2c(F)cc3c(N(C4CC4)C=C(C(O)=O)C3=O)c2OC	C20H24FN3O4	389.421	Balofloxacin
Selleck Chemicals	S2259	c(C(=O)c1c(cccc1O)C2=O)(c(O)cc(CO)c3)c23	C15H10O5	270.237	Aloe-emodin
Selleck Chemicals	S2087	c1(OC(=O)N(CC)C)cc([C@H](C)N(C)C)ccc1.OC(=O)[C@H](O)[C@@H](O)C(=O)O	C18H28N2O8	400.424	Rivastigmine Tartrate
Selleck Chemicals	S2267	O=[N+]([O-])c1[nH]ccn1	C3H3N3O2	113.075	Azomycin
Selleck Chemicals	S2118	CS(Nc1ccc(C(O)CCCN(CC)CCCCCCC)cc1)(=O)=O.C(O)(=O)C=CC(O)=O.CS(Nc2ccc(C(O)CCCN(CC)CCCCCCC)cc2)(=O)=O	C44H76N4O10S2	885.225	Ibutilide Fumarate
Selleck Chemicals	S2282	c1cccc2c1c([C@@H](O)[C@]3([H])CC([C@@]([H])(C=C)CN34)CC4)ccn2	C19H22N2O	294.391	Cinchonidine
Selleck Chemicals	S2130	OS(=O)(=O)O.O.C(=O)(O[C@@H]1C[C@@]([H])(N(C)[C@@]2([H])C1)CC2)C(CO)c3ccccc3.C(=O)(O[C@@H]4C[C@@]([H])(N(C)[C@@]5([H])C4)CC5)C(CO)c6ccccc6	C34H50N2O11S	694.833	Atropine

Selleck Chemicals	S2299	c(C(=O)C(c1ccc(OC)cc1)=CO2)(ccc(O)c3)c23	C16H12O4	268.264	Formononetin
Selleck Chemicals	S1557	c1(OC)cc2c(ncnc2Oc3cc(Cl)c(NC(NCCC)=O)cc3)cc1OC	C20H21ClN4O4	416.858	KRN 633
Selleck Chemicals	S1723	c1cc2c(c(CC(=O)O)c(C)n2C(=O)c3ccc(Cl)cc3)cc1OC	C19H16ClNO4	357.788	Indomethacin
Selleck Chemicals	S1572	c1(NCc2ccccc2)n3c(c(C(C)C)cn3)nc(NCCCCCCN)c1.Cl	C22H33ClN6	416.991	BS-181 HCl
Selleck Chemicals	S1736	c1c(OC)c(OCC(COC(=O)N)O)ccc1	C11H15NO5	241.241	Methocarbamol
Selleck Chemicals	S1593	n1n(c2ccc(OC)cc2)c3c(CCN(c4ccc(N5CCCCC5=O)cc4)C3=O)c1C(=O)N	C25H25N5O4	459.497	Apixaban
Selleck Chemicals	S1760	c1(C=NN2CCN(C3CCCC3)CC2)c(O)c(c(C(=O)[C@]4(C)O5)c56)c(c(O)c6C)c(O)c1NC(=O)C(C)=CC=C[C@H](C)[C@H](O)[C@@H](C)[C@@H](O)[C@@H](C)[C@H](OC(C)=O)[C@H](C)[C@@H](OC)C=CO4	C47H64N4O12	877.031	Rifapentine
Selleck Chemicals	S1613	c1cc(OCCN[C@@H](Cc2cc(C(=O)N)c3c(CCN3CCCO)c2)C)c(OCC(F)(F)F)cc1	C25H32F3N3O4	495.534	Silodosin
Selleck Chemicals	S1778	N1C(=O)C(C(F)(F)F)=CN([C@@H]2O[C@H](CO)[C@@H](O)C2)C1=O	C10H11F3N2O5	296.2	Trifluridine
Selleck Chemicals	S1628	C1=C[C@@]2(C)C(CC[C@]3([H])[C@@]2(F)[C@H](O)C[C@@]4(C)[C@@]3([H])C[C@@H]5[C@]4(C(=O)CO)OC(C)(C)O5)=CC1=O	C24H31FO6	434.498	Triamcinolone Acetonide
Selleck Chemicals	S1802	[C@@H]1(O)[C@H](CO)O[C@@H](n2c(N)c(C(N)=O)nc2)[C@@H]1O	C9H14N4O5	258.231	Acadesine
Selleck Chemicals	S1643	C1C[C@@]2(C)[C@]([H])(C[C@H](O)[C@]3([H])[C@@]2([H])CC[C@@]4(C)[C@@]3([H])CC[C@@]4([H])[C@@H](CCC(=O)O)C)C[C@@H]1O	C24H40O4	392.572	Ursodiol
Selleck Chemicals	S1831	c1([nH]c2c3cccc2)c3c(OCC(O)CNCCOc4c(OC)cccc4)ccc1	C24H26N2O4	406.474	Carvedilol
Selleck Chemicals	S1655	c1cc([C@@H](O)CC[C@@H]2[C@H](c3ccc(O)cc3)N(c4ccc(F)cc4)C2=O)ccc1F	C24H21F2NO3	409.425	Ezetimibe
Selleck Chemicals	S1848	c1(OC)cc(C=CC(=O)C=C(O)C=Cc2cc(OC)c(O)cc2)ccc1O	C21H20O6	368.38	Curcumin
Selleck Chemicals	S1672	c1(C2(CC)C(=O)NC(=O)CC2)ccc(N)cc1	C13H16N2O2	232.278	Aminoglutethimide
Selleck Chemicals	S1881	c1(CCC)cc(C(=S)N)ccn1	C9H12N2S	180.27	Protionamide
Selleck Chemicals	S1691	c1cc2c(CCN3C2CN(C(=O)C4CCCCC4)CC3=O)cc1	C19H24N2O2	312.406	Praziquantel
Selleck Chemicals	S1902	[C@H]1(CC(=O)N)[C@]2([H])N=C(C(C)=C(N=C3C=C(N=C4C(C)=C([N-][C@@]25C)[C@@H](CCC(=O)N)[C@]5(C)CC(=O)N)[C@@H](CCC(=O)N)[C@@]4(CC(=O)N)C)[C@@H](CCC(=O)N)C3(C)C)[C@]1(C)CCC(=O)NC[C@@H](C)OP(=O)([O-])O[C@@H]6[C@H](CO)O[C@H](n7c8c(cc(C)c(C)c8)nc7)[C@@H]6O.[Co+2]C#N	C63H88CoN14O14P	1355.365	Vitamin B12
Selleck Chemicals	S1709	c1cc2c(CC[C@]3([H])[C@@]2([H])CC[C@@]4(C)[C@@]3([H])CC[C@H]4O)cc1O	C18H24O2	272.382	Estradiol
Selleck Chemicals	S1915	c1cc(S(=O)(=O)Nc2noc(C)c2)ccc1N	C10H11N3O3S	253.278	Sulfamethoxazole
Selleck Chemicals	S1949	c1cc2c(C(=O)C=C(C)C2=O)cc1	C11H8O2	172.18	Menadione
Selleck Chemicals	S2159	[C@]1([H])([C@H](O)C)[C@@]2([H])N(C(C(=O)OCOC(=O)C(C)(C)C)=C(SC3CN(C4=NCCS4)C3)[C@H]2C)C1=O	C22H31N3O6S2	497.628	Tebipenem Pivoxil
Selleck Chemicals	S1971	c1cc(C(NCCO[N+](=O)[O-])=O)cnc1	C8H9N3O4	211.175	Nicorandil
Selleck Chemicals	S2181	c1c(Cl)c(C(=O)NCC(=O)N[C@@H](CC(C)C)B2OC(=O)CC(CC(O)=O)(C(O)=O)O2)cc(Cl)c1	C20H23BCl2N2O9	517.122	MLN9708
Selleck Chemicals	S2003	c1cc([C@@H](NC(=O)C2CCC(F)(F)CC2)CCN(C3CC4)C4CC(n5c(C(C)C)nnc5C)C3)ccc1	C29H41F2N5O	513.666	Maraviroc
Selleck Chemicals	S2198	c1cc2n(c(c3cc(OC(F)(F)F)ccc3)cn2)nc1NCC4CCN(C)CC4	C20H22F3N5O	405.417	SGI-1776 free base
Selleck Chemicals	S2024	c1(scc2)c2C(=C3CCN(C)CC3)c4c(cccc4)CC1=O.C(=O)(O)C=CC(=O)O	C23H23NO5S	425.497	Ketotifen Fumarate
Selleck Chemicals	S2216	c1cc(C=Cc2nc(COc3ccc(CCCCn4ccnn4)cc3)co2)ccc1C(F)(F)F	C25H23F3N4O2	468.471	Mubritinib (TAK 165)
Selleck Chemicals	S2036	C(=O)(O)C[C@H](N)C(=O)N[C@@H](Cc1ccccc1)C(=O)OC	C14H18N2O5	294.303	Aspartame
Selleck Chemicals	S2227	n1c(N)c2c(n(CC3=Nc4c(c(C)ccc4)C(=O)N3c5ccccc5C)nc2c6cccc(O)c6)nc1	C28H23N7O2	489.528	PIK-294

Selleck Chemicals	S2053	C1(=O)N=C(N)C=CN1[C@H]2[C@@H](O)[C@H](O)[C@@H](CO)O2	C9H13N3O5	243.217	Cytidine
Selleck Chemicals	S2243	c1(Br)nc(C=C(C#N)C(=O)N[C@@H](CCC)c2ccccc2)ccc1	C19H18BrN3O	384.27	Degrasyn (WP1130)
Selleck Chemicals	S2065	c1c(CN2CCCCC2)ccnc1OCC=CCNC(=O)CS(Cc3occc3)=O	C22H29N3O4S	431.548	Lafutidine
Selleck Chemicals	S2260	[C@H]1(O)[C@@H](O)[C@@H](CO)O[C@@H](OC[C@H]2O[C@@H](O[C@@H](C#N)c3ccccc3)[C@H](O)[C@@H](O)[C@@H]2O)[C@@H]1O	C20H27NO11	457.428	Laetrile
Selleck Chemicals	S2091	c1c(CCOCC2CC2)ccc(OCC(O)CNC(C)C)c1	C18H29NO3	307.428	Betaxolol
Selleck Chemicals	S2268	c1(O)cc2c(C(=O)C=C(c3ccccc3)O2)c(O)c1O	C15H10O5	270.237	Baicalein
Selleck Chemicals	S2119	c1(C(C)(C)C)cc(SC(C)(C)Sc2cc(C(C)(C)C)c(O)c(C(C)(C)C)c2)cc(C(C)(C)C)c1O	C31H48O2S2	516.842	Probucol
Selleck Chemicals	S2285	C1Cc2c(ccc3c2C(=O)C(=O)C4=C3OC[C@H]4C)C(C)(C)C1	C19H20O3	296.36	Cryptotanshinone
Selleck Chemicals	S2131	n1cc(Cl)c(NC(=O)c2cc(OCC3CC3)c(OC(F)F)cc2)c(Cl)c1	C17H14Cl2F2N2O3	403.207	Roflumilast
Selleck Chemicals	S2300	c1cc(C=CC(=O)O)cc(OC)c1O	C10H10O4	194.184	Ferulic Acid
Selleck Chemicals	S2302	C1C[C@@]2(C)[C@@]([H])(CC[C@]3(C)[C@@]2([H])C(=O)C=C4[C@@]3(C)CC[C@]5(C)[C@]4([H])C[C@@](C)(C(=O)O)CC5)C(C)(C)[C@H]1O[C@@H]6[C@@H](O[C@H]7[C@@H](O)[C@@H](O)[C@H](O)[C@@H](C(=O)O)O7)[C@@H](O)[C@H](O)[C@@H](C(=O)O)O6	C42H62O16	822.932	Glycyrrhizic Acid
Selleck Chemicals	S2467	n1cc2c(n(CCC(COC(=O)C)COC(C)=O)cn2)nc1N	C14H19N5O4	321.332	Famciclovir
Selleck Chemicals	S2320	c1(O)cc2c(C(=O)C=C(c3cc(O)c(O)cc3)O2)c(O)c1	C15H10O6	286.236	Luteolin
Selleck Chemicals	S2480	c1cc(C2(O)CCN(CCC(c3ccccc3)(c4ccccc4)C(N(C)C)=O)CC2)ccc1Cl.Cl	C29H34Cl2N2O2	513.498	Loperamide HCl
Selleck Chemicals	S2337	c1(OC)c(CC=C(C)C)c2c(C=CC(=O)O2)cc1	C15H16O3	244.286	Osthole
Selleck Chemicals	S2492	[C@H]1(OC)C(C)(C)O[C@@H](Oc2c(C)c3c(C(O)=C(NC(c4cc(CC=C(C)C)c([O-])cc4)=O)C(=O)O3)cc2)[C@H](O)[C@@H]1OC(=O)N.[Na+]	C31H35N2NaO11	634.606	Novobiocin Sodium
Selleck Chemicals	S2347	c1(O)cc2c(C(=O)C(O)=C(c3cc(O)c(O)cc3)O2)c(O)c1.O.O	C15H14O9	338.266	Quercetin Dihydrate
Selleck Chemicals	S2502	c1c(OC)cc2c(nccc2[C@H](O)[C@]3([H])C[C@@]([H])([C@@H](C=C)CN34)CC4)c1.Cl.O.O	C20H29ClN2O4	396.908	Quinine HCl Dihydrate
Selleck Chemicals	S2364	c1c(C)c2c(c3c(c4c(c(C)co4)C(=O)C3=O)cc2)cc1	C18H12O3	276.286	Tanshinone I
Selleck Chemicals	S2515	C1CN(S(=O)(=O)c2cc(C(NC(=O)c3n4c(CCC)nc3C)=N4)c(OCC)cc2)CCN1CC.Cl.O.O.O	C23H39ClN6O7S	579.11	Vardenafil HCl Trihydrate
Selleck Chemicals	S2377	c1(O)cc2c(C(=O)C(c3ccc(OC)cc3)=CO2)c(O)c1	C16H12O5	284.263	Biochanin A
Selleck Chemicals	S2534	c1cc(C(Cn2cncc2)OCc3c(Cl)cccc3Cl)c(Cl)cc1Cl.[N+](O)(=O)[O-]	C18H15Cl4N3O4	479.141	Isoconazole nitrate
Selleck Chemicals	S2390	[C@@H]1(O)[C@H](O)[C@H](Oc2cc(C=Cc3ccc(O)cc3)cc(O)c2)O[C@H](CO)[C@H]1O	C20H22O8	390.384	Polydatin
Selleck Chemicals	S2548	Cl.[C@@]1([H])(C[C@@H](O)C2)N(C)[C@]2([H])[C@@]3([H])[C@]1([H])O3	C8H14ClNO2	191.655	Scopine HCl
Selleck Chemicals	S2403	c1c(OC)c(OC)c2c([C@H]3Cc(cc4Oc5ccc(cc5)C[C@H]6c(cc7O2)c(CCN6C)cc7OC)ccc4OC)c1CCN3C	C38H42N2O6	622.75	Tetrandrine
Selleck Chemicals	S2561	c1cc(C(Cl)=C(c2ccc(OCCN(CC)CC)cc2)c3ccccc3)ccc1.OC(=O)CC(O)(C(O)=O)CC(=O)O	C32H36ClNO8	598.083	Clomifene citrate
Selleck Chemicals	S2412	O1[C@H](O)[C@@]2([H])[C@]([H])(CC=C2CO)C(C(OC)=O)=C1	C11H14O5	226.226	Genipin
Selleck Chemicals	S2579	N1C(=O)C(C)=CN([C@H]2C[C@H](N=[N+]=[N-])[C@@H](CO)O2)C1=O	C10H13N5O4	267.241	Zidovudine
Selleck Chemicals	S2446	C(#N)C(=NNc1ccc(C2=NNC(=O)C[C@H]2C)cc1)C#N	C14H12N6O	280.285	Levosimendan
Selleck Chemicals	S2599	c1(O)c(I)cc(Oc2c(I)cc(C[C@H](N)C(=O)O)cc2I)cc1I	C15H11I4NO4	776.87	L-Thyroxine
Selleck Chemicals	S2608	C1=C[C@@]2(C)C([C@@H](F)C[C@]3([H])[C@@]2(F)[C@H](O)C[C@@]4(C)[C@@]3([H])C[C@@H]5[C@]4(C(=O)COC(=O)C)OC(C)(C)O5)=CC1=O	C26H32F2O7	494.525	Fluocinonide
Selleck Chemicals	S2752	n1c(c2c(Nc3ccc(Oc4ccn5c(ncn5)c4)c(C)c3)nc1)ccc(c6ccc(CNCCS(C)(=O)=O)o6)c2	C29H27N7O4S	569.634	HER2-Inhibitor-1

Selleck Chemicals	S2621	c1cc(Nc2nc(c3n(C(C)C)c(C)nc3)ccn2)ccc1S(=O)(C)=O	C18H21N5O2S	371.457	AZD5438
Selleck Chemicals	S2768	C1CCN(c2nc3n(ncc3CC)c(NCc4c[n+]([O-])ccc4)c2)[C@H](CCO)C1	C21H28N6O2	396.486	Dinaciclib (SCH727965)
Selleck Chemicals	S2631	c1ccc(OC(=O)NC2CCCCC2)cc1c3cc(C(=O)N)ccc3	C20H22N2O3	338.4	URB597
Selleck Chemicals	S2779	c1(N(C)C)ccc(C(=O)NCCCCCCC(=O)NO)cc1	C16H25N3O3	307.388	M344
Selleck Chemicals	S2656	n1(c2cc(N[C@H]3CC[C@H](OC(=O)CN)CC3)c(C(N)=O)cc2)nc(C(F)(F)F)c4c1CC(C)(C)CC4=O	C25H30F3N5O4	521.532	PF-04929113 (SNX-5422)
Selleck Chemicals	S2790	n1(C)c(c2nc1C=Cc3ccc(OC)c(OC)c3)C(=O)N(CC)C(=O)N2CC	C20H24N4O4	384.429	Istradefylline
Selleck Chemicals	S2666	c1(C(F)(F)F)ccc(Oc2cccc(CC3CCN(C(=O)Nc4cccnc4)CC3)c2)nc1	C24H23F3N4O2	456.46	PF-3845
Selleck Chemicals	S2803	[C@](C)(C(n1cnc2c1cccc2)=CC3)(CCC4C5CC=C6[C@]4(C)CC[C@H](O)C6)C35	C26H32N2O	388.545	Galeterone
Selleck Chemicals	S2681	c(OC(F)(F)O1)(cc(C=C2C(=O)NC(=O)S2)cc3)c13	C11H5F2NO4S	285.224	AS-604850
Selleck Chemicals	S2924	c1(NCCNc2ccc(C#N)cn2)nc(c3c(Cl)cc(Cl)cc3)c(c4nc(C)c[nH]4)cn1.Cl	C22H19Cl3N8	501.799	CHIR-99021 (CT99021) HCl
Selleck Chemicals	S2692	c1ccc(Nc2c(C)cnc(Nc3ccc(N4CCN(C)CC4)cc3)n2)cc1S(=O)(=O)NC(C)(C)C	C26H35N7O2S	509.667	TG101209
Selleck Chemicals	S3019	C(C(=O)N(O)C(C1CCCCC1)=C2)=C2C.NCCO	C14H24N2O3	268.352	Ciclopirox ethanolamine
Selleck Chemicals	S2714	c1(F)cc([C@H](O)C(=O)N[C@@H](C)C(=O)N[C@@H]2C(=O)N(C)c3c(cccc3)c4c2cccc4)cc(F)c1	C26H23F2N3O4	479.475	LY411575
Selleck Chemicals	S3061	c1(O)ccc([C@@H](O)CNC)cc1O.Cl	C9H14ClNO3	219.665	Epinephrine HCl
Selleck Chemicals	S2728	c1c(OC)c(OC)cc2c1ncnc2Nc3cccc(Cl)c3	C16H14ClN3O2	315.754	AG-1478 (Tyrphostin AG-1478)
Selleck Chemicals	S5002	c1(CCC(CO)(N)CO)ccc(CCCCCCCC)cc1.Cl	C19H34ClNO2	343.932	Fingolimod (FTY720) HCl
Selleck Chemicals	S2743	n1c(C)c2c(N([C@@H]3CC[C@@H](OCCO)CC3)C(=O)C(c4cnc(OC)cc4)=C2)nc1N	C22H27N5O4	425.481	PF-04691502
Selleck Chemicals	S2308	c1(O)cc2c(C(=O)C[C@@H](c3cc(O)c(OC)cc3)O2)c(O)c1	C16H14O6	302.279	Hesperetin
Selleck Chemicals	S2468	c1ccc(Sc2ccc3c([nH]c(NC(=O)OC)n3)c2)cc1	C15H13N3O2S	299.348	Fenbendazole
Selleck Chemicals	S2322	C1[C@@]2([H])[C@@]([H])([C@]3([H])[C@]4([H])N(C(=O)CCC4)C2)N(CCC3)CC1	C15H24N2O	248.364	(+)-Matrine
Selleck Chemicals	S2481	c1([N+](=O)[O-])cccc(C2C(C(=O)OCCN3CCN(C(c4ccccc4)c5ccccc5)CC3)=C(C)NC(C)=C2C(OC)=O)c1	C35H38N4O6	610.699	Manidipine
Selleck Chemicals	S2338	C1[C@@]2([H])[C@@]([H])([C@]3([H])[C@]4([H])N(C(=O)CCC4)C2)[N+]([O-])(CCC3)CC1	C15H24N2O2	264.363	Oxymatrine
Selleck Chemicals	S2494	C1(c2c(cccc2)COc3c1cc(CC(=O)O)cc3)=CCCN(C)C.Cl	C21H24ClNO3	373.873	Olopatadine HCl
Selleck Chemicals	S2349	N(CCc1c2[nH]c3c1cccc3)(C(=O)c4c5cccc4)C2=N5	C18H13N3O	287.315	Rutaecarpine
Selleck Chemicals	S2503	c1ccc(COC(=O)CNC(=O)C(Cc2ccccc2)CSC(C)=O)cc1	C21H23NO4S	385.477	Racecadotril
Selleck Chemicals	S2365	C1Cc2c(ccc3c2C(=O)C(=O)c4c3occ4C)C(C)(C)C1	C19H18O3	294.344	Tanshinone IIA
Selleck Chemicals	S2517	c1ccc2c(C3c4c(cccc4)C2(CCCNC)CC3)cc1.Cl	C20H24ClN	313.864	Maprotiline HCl
Selleck Chemicals	S2378	[Br-].c1ccccc1[C@H](CO)C(=O)O[C@@H]2C[C@@]([H])([N+](C)(CCCC)[C@@]3([H])C2)[C@]4([H])[C@@]3([H])O4	C21H30BrNO4	440.371	Butylscopolamine Bromide
Selleck Chemicals	S2535	c1cc(C(OCc2ccc(Cl)cc2)Cn3cncc3)c(Cl)cc1Cl.[N+](O)(=O)[O-]	C18H16Cl3N3O4	444.696	Econazole nitrate
Selleck Chemicals	S2391	c1(O)cc2c(C(=O)C(O)=C(c3cc(O)c(O)cc3)O2)c(O)c1	C15H10O7	302.236	Quercetin
Selleck Chemicals	S2549	[Cl-].[C@@]1([H])(C[C@@H](OC(=O)C(O)(c2ccccc2)c3ccccc3)C4)[N+]5(CCCC5)[C@]4([H])CC1	C25H30ClNO3	427.964	Trospium chloride
Selleck Chemicals	S2404	c1cc(C=CC(=O)c2ccc(O)cc2O)ccc1O	C15H12O4	256.253	Isoliquiritigenin

Selleck Chemicals	S2564	[C@H]1(NC(c2c(c3c(Cl)cccc3)noc2C)=O)[C@@]4([H])N([C@@H](C([O-])=O)C(C)(C)S4)C1=O.[Na+].O	C19H19ClN3NaO6S	475.878	Cloxacillin Sodium
Selleck Chemicals	S2413	C1=C(C(O)=O)[C@]2([H])[C@@]([H])(C(CO)=CC2)[C@H](O[C@H]3[C@@H](O)[C@@H](O)[C@H](O)[C@@H](CO)O3)O1	C16H22O10	374.34	Geniposidic acid
Selleck Chemicals	S2583	c1cc([C@@H](O)[C@@H](CO)NC(=O)C(Cl)Cl)ccc1S(=O)(=O)C	C12H15Cl2NO5S	356.222	Thiamphenicol
Selleck Chemicals	S2451	C1(CC(N)(CC2C1)CC3C2)C3.Cl	C10H18ClN	187.71	Amantadine HCl
Selleck Chemicals	S2601	c1cc(S(=O)(=O)NC(=O)NN2CC3C(CCC3)C2)ccc1C	C15H21N3O3S	323.411	Gliclazide
Selleck Chemicals	S2612	OC[C@@H](O)[C@@H](O)[C@@H](O)CO	C5H12O5	152.146	Ribitol
Selleck Chemicals	S2755	c(ccc(NC1=N[C@H](C)CO1)c2)(ncnc3Nc4ccc(OCc5nccs5)c(Cl)c4)c23	C22H19ClN6O2S	466.943	Varlitinib
Selleck Chemicals	S2622	c1(c2c3c(ncnc3N)n(C4CCCC4)n2)cc5c([nH]cc5)nc1	C17H17N7	319.364	PP121
Selleck Chemicals	S2770	n1c(C[C@]2(C(O)=O)CC[C@H](Oc3cccc(Cl)c3F)CC2)cccc1Nc4nccs4	C22H21ClFN3O3S	461.937	MK-5108 (VX-689)
Selleck Chemicals	S2632	c1c(OC)c2c(ncc2C(=O)C(=O)N3CCN(C(c4ccccc4)=O)C[C@H]3C)[nH]c1	C22H22N4O4	406.434	BMS-378806
Selleck Chemicals	S2780	c1(OC)cc2c(ncc3c2N([C@@H](c4ccccc4)C)C(=O)N3)cc1c5c(C)noc5C	C23H21N5O3	415.445	I-BET151 (GSK1210151A)
Selleck Chemicals	S2658	c1(S(Nc2cc(c3ccc(c4c3)nccc4c5ccnnc5)cnc2OC)(=O)=O)ccc(F)cc1F	C25H17F2N5O3S	505.496	GSK2126458 (GSK458)
Selleck Chemicals	S2791	N1C(=O)C(c2c(c3nc(N4CCN(C)CC4)n2)cccc3)=C(c5c(c6[nH]c5)cccc6)C1=O	C25H22N6O2	438.481	Sotrastaurin
Selleck Chemicals	S2667	c1(F)ccc(CNC(=O)C(C(=O)C(O)=C2N3C[C@@]4([H])N([C@@H](C)CCO4)C2=O)=C3)c(F)c1	C20H19F2N3O5	419.379	Dolutegravir (GSK1349572)
Selleck Chemicals	S2807	c1(S(Nc2cccc(c3c(c4ccnc(N)n4)sc(C(C)(C)C)n3)c2F)(=O)=O)c(F)cccc1F	C23H20F3N5O2S2	519.562	Dabrafenib (GSK2118436)
Selleck Chemicals	S2682	c1(F)ccc(c2ccc(C=C3SC(=O)NC3=O)o2)cc1	C14H8FNO3S	289.282	CAY10505
Selleck Chemicals	S3000	N1(C)C(C(=C2)CC1S([O-])(=O)=O)=CC(=O)C2=NNC(N)=O.[Na+]	C10H11N4NaO5S	322.273	Carbazochrome sodium sulfonate (AC-17)
Selleck Chemicals	S2696	c1(c2nc3c(sc(CN4CCN(C(=O)[C@H](C)O)CC4)c3C)c(N5CCOCC5)n2)cnc(N)nc1	C23H30N8O3S	498.601	GDC-0980 (RG7422)
Selleck Chemicals	S3020	S1CCC=C[C@@H]2CC(=O)N[C@@H](C(C)C)C(=O)N[C@@H](C(=O)NC(=CC)C(=O)N[C@@H](C(C)C)C(=O)O2)CS1	C24H36N4O6S2	540.696	Romidepsin (FK228, Depsipeptide)
Selleck Chemicals	S2717	c1(Cl)cc2c([nH]c(C(=O)N[C@@H](Cc3ccccc3)[C@@H](O)C(N(C)C)=O)c2)cc1	C21H22ClN3O3	399.871	CP-91149
Selleck Chemicals	S3062	c1cc(CC([O-])=O)c(Nc2c(Cl)cccc2Cl)cc1.[K+]	C14H10Cl2KNO2	334.239	Diclofenac Potassium
Selleck Chemicals	S2729	C1(=O)NC(=O)C(Nc2ccc(O)c(Cl)c2)=C1c3ccccc3[N+]([O-])=O	C16H10ClN3O5	359.721	SB415286
Selleck Chemicals	S5003	[C@H]1(OC)[C@@H](O)CC[C@@H](C=C(C)[C@H]2OC(=O)[C@@]3([H])N(CCCC3)C(=O)C(=O)[C@](O)(O[C@H]4[C@@H](OC)C[C@@H](C)CC(C)=C[C@@H](CC=C)C(=O)C[C@H](O)[C@H]2C)[C@H](C)C[C@@H]4OC)C1	C44H69NO12	804.018	Tacrolimus (FK506)
Selleck Chemicals	S2744	c1(Br)c(N2CCN(Cc3noc(C)c3)CC2)c4c([nH]c(c5ccc(N6CCN(C)CC6)cc5)n4)nc1	C26H31BrN8O	551.481	CCT137690
Selleck Chemicals	S2309	[C@H]1(CO[C@@H]2O[C@@H](C)[C@H](O)[C@H](O)[C@H]2O)O[C@@H](Oc3cc4c(C(=O)C[C@@H](c5cc(O)c(OC)cc5)O4)c(O)c3)[C@H](O)[C@@H](O)[C@@H]1O	C28H34O15	610.561	Hesperidin
Selleck Chemicals	S2470	C1=C[C@@]2(C)C([C@](F)([H])C[C@]3([H])[C@@]2(F)[C@H](O)C[C@@]4(C)[C@@]3([H])C[C@]5([H])[C@]4(C(=O)CO)OC(C)(C)O5)=CC1=O	C24H30F2O6	452.488	Fluocinolone Acetonide
Selleck Chemicals	S2323	[C@H]1(O)[C@@H](C)O[C@@H](OC[C@H]2O[C@@H](Oc3cc4c(C(=O)C[C@@H](c5cc(O)c(OC)cc5)O4)c(O)c3)[C@H](O)[C@@H](O)[C@@H]2O)[C@H](O)[C@@H]1O.CO	C29H38O16	642.602	Methyl-Hesperidin
Selleck Chemicals	S2482	c1([N+](=O)[O-])cccc(C2C(C(=O)OCCN3CCN(C(c4ccccc4)c5ccccc5)CC3)=C(C)NC(C)=C2C(OC)=O)c1.Cl.Cl	C35H40Cl2N4O6	683.621	Manidipine 2HCl
Selleck Chemicals	S2339	c1(C(C)=O)ccc(OC)cc1O	C9H10O3	166.174	Paeonol
Selleck Chemicals	S2495	c1(C(C)(C)C)cc(C)c(CC2=NCCN2)c(C)c1O.Cl	C16H25ClN2O	296.835	Oxymetazoline HCl
Selleck Chemicals	S2351	[C@H]1(CO)O[C@@H](Oc2c(CO)cccc2)[C@H](O)[C@@H](O)[C@@H]1O	C13H18O7	286.278	Salicin

Selleck Chemicals	S2504	O1[C@H](n2nc(C(=O)N)nc2)[C@H](O)[C@H](O)[C@H]1CO	C8H12N4O5	244.205	Ribavirin
Selleck Chemicals	S2366	c1(O)cc2c(C(=O)[C@H](O)[C@@H](c3ccc(O)c(O)c3)O2)c(O)c1	C15H12O7	304.252	Taxifolin (Dihydroquercetin)
Selleck Chemicals	S2522	c1(O)cc([C@H](CNC)O)ccc1O	C9H13NO3	183.204	L-Adrenaline
Selleck Chemicals	S2379	[C@H]1(O)[C@H](O)[C@]([H])(O[C@@H]2[C@H](CO)O[C@@]([H])(O[C@@H]3CC[C@@]4(C)C(=CC[C@]5([H])[C@@]4([H])CC[C@@]6(C)[C@@]5([H])C[C@@]7([H])[C@@]6([H])[C@@H](C)[C@@]8(CC[C@@H](C)CO8)O7)C3)[C@H](O[C@]9([H])O[C@@H](C)[C@H](O)[C@@H](O)[C@H]9O)[C@H]2O)O[C@@H](C)[C@@H]1O	C45H72O16	869.044	Dioscin
Selleck Chemicals	S2536	c1(Cl)cc(Cl)c(COC(Cn2cncc2)c3ccc(Cl)cc3Cl)cc1	C18H14Cl4N2O	416.129	Miconazole
Selleck Chemicals	S2392	c(OCO1)(cc([C@@H]2[C@@]3([H])[C@@]([H])([C@@H](c4ccc5c(OCO5)c4)OC3)CO2)cc6)c16	C20H18O6	354.353	Sesamin
Selleck Chemicals	S2552	c1cc2c(C(Cc3ccc(Cl)cc3)=NN(C4CCCN(C)CC4)C2=O)cc1.Cl	C22H25Cl2N3O	418.359	Azelastine HCl
Selleck Chemicals	S2405	C(C[C@@]1([H])N(C[C@@]2([H])[C@]([H])(N3CCC2)[C@@]1([H])CCC3)C4=O)=C4	C15H22N2O	246.348	Sophocarpine
Selleck Chemicals	S2565	c1(O)ccc([C@@H](N)C(=O)N[C@@H]2C(=O)N3[C@]2([H])SC(C)(C)[C@H]3C([O-])=O)cc1.[Na+]	C16H18N3NaO5S	387.386	Amoxicillin Sodium
Selleck Chemicals	S2415	[C@@H]1(O)CO[C@@H](O[C@H]2CC[C@](C3)([C@@]3(CC[C@@]4(C)[C@@]5(C)C[C@H](O)[C@@]4([H])[C@@]6(C)O[C@@H](C(O)(C)C)CC6)[C@]57[H])[C@@]([H])([C@@H](O[C@H]8[C@@H](O)[C@@H](O)[C@H](O)[C@@H](CO)O8)C7)C2(C)C)[C@H](O)[C@H]1O	C41H68O14	784.97	Astragaloside A
Selleck Chemicals	S2584	C1=C[C@@]2(C)C(CC[C@]3([H])[C@@]2(F)[C@]([H])(O)C[C@@]4(C)[C@@]3([H])C[C@]([H])(C)[C@@]4(OC(=O)CC)C(=O)CCl)=CC1=O	C25H32ClFO5	466.97	Clobetasol propionate
Selleck Chemicals	S2453	c1(O)ccc(CNNC(=O)C(CO)N)c(O)c1O.Cl	C10H16ClN3O5	293.704	Benserazide HCl
Selleck Chemicals	S2602	Cc1c(CC(=O)OCC(=O)O)c2cc(ccc(OC)c2)n1C(c3ccc(Cl)cc3)=O	C21H18ClNO6	415.824	Acemetacin
Selleck Chemicals	S2613	c1(C(=C(Cl)Cl)Cl)cc(N)c(S(=O)(=O)N)cc1S(=O)(=O)N	C8H8Cl3N3O4S2	380.656	Clorsulon
Selleck Chemicals	S2758	C1(=O)O[C@H](COC)[C@@]2(C)c(c3C(=O)C4=C2[C@@H](OC(C)=O)C[C@@](C)([C@]45[H])C(=O)CC5)c1co3	C23H24O8	428.432	Wortmannin
Selleck Chemicals	S2623	c1(C)ccc(NC(=O)Nc2cccc(CN3CCN(C(OC)=O)CC3)c2F)cn1	C20H24FN5O3	401.435	Omecamtiv mecarbil (CK-1827452)
Selleck Chemicals	S2772	c1c(NC(=O)C2(CC(CC)CC)CCCCC2)c(SC(=O)C(C)C)ccc1	C23H35NO2S	389.595	Dalcetrapib (JTT-705, RO4607381)
Selleck Chemicals	S2633	c1(Cl)c(C#N)c(OC[C@H](O)CNC(C)(C)Cc2cc3c(cccc3)cc2)ccc1	C24H25ClN2O2	408.921	NPS-2143
Selleck Chemicals	S2781	o1c(c2sc(CO)cc2)ccc1c3sc(CO)cc3	C14H12O3S2	292.373	RITA (NSC 652287)
Selleck Chemicals	S2659	c1c([C@@H](CCN(C(C)C)C(C)C)c2cc(CO)ccc2O)cccc1	C22H31NO2	341.487	5-hydroxymethyl Tolterodine (PNU 200577, 5-HMT, 5-HM)
Selleck Chemicals	S2792	c(cc(C(F)(F)F)cc1)([C@H](N(Cc2cc(C(F)(F)F)cc(C(F)(F)F)c2)C(OC)=O)C[C@@H](CC)N3C(=O)OCC)c13	C26H25F9N2O4	600.473	Torcetrapib
Selleck Chemicals	S2670	c1cc(C[C@H](N)COc2cncc(c3ccc4c(c(C)n[nH]4)c3)c2)ccc1	C22H22N4O	358.436	A-674563
Selleck Chemicals	S2808	C1N(C(=O)[C@H](CNC(C)C)c2ccc(Cl)cc2)CCN(c3ncnc4c3[C@H](C)C[C@@H]4O)C1	C24H32ClN5O2	457.996	GDC-0068
Selleck Chemicals	S2683	C1(=O)Nc2c(cc(Cl)cc2)C(N[C@@H]3CN(CCC34)CC4)=C1c5[nH]c6c(cccc6)n5	C23H22ClN5O	419.907	CHIR-124
Selleck Chemicals	S3001	N1C(=O)N([C@@H]2[C@@H](F)[C@@H](O)[C@H](CO)O2)C=C(C)C1=O	C10H13FN2O5	260.219	Clevudine
Selleck Chemicals	S2697	c1(c2c(O)cccc2)ccc(c3c4c(NC(=O)C(C#N)=C4O)sc3)cc1	C20H12N2O3S	360.386	A-769662
Selleck Chemicals	S3022	[C@@](C)(C(=O)[C@H](OC)C(C(C)(C)[C@@]1(O)[C@@H]2OC(c3ccccc3)=O)=C(C)[C@@H](OC(=O)[C@H](O)[C@H](c4ccccc4)NC(=O)OC(C)(C)C)C1)([C@H](OC)C[C@]5([H])[C@@]6(OC(C)=O)CO5)[C@]26[H]	C45H57NO14	835.932	Cabazitaxel
Selleck Chemicals	S2718	C1CC(NC(=O)c2cc(c3cccc(S(=O)(=O)CC)c3)c4c([nH]c5c4cc(C)cn5)c2C)CN1C	C28H32N4O3S	504.644	TAK-901
Selleck Chemicals	S3117	c1c(C(O)(C(=O)OCC#CCN(CC)CC)C2CCCCC2)cccc1.Cl	C22H32ClNO3	393.947	Oxybutynin chloride
Selleck Chemicals	S2730	c1cc2c(nc(n3c4c(cc(OCC5(C)COC5)cc4)nc3)cc2)c(N6CCC(N)CC6)c1	C26H29N5O2	443.541	Crenolanib (CP-868596)

Selleck Chemicals	S6002	c1(C=[N+]([O-])C(C)(C)C)c(S([O-])(=O)=O)cc(S(=O)(=O)[O-])cc1.[Na+].[Na+]	C11H13NNa2O7S2	381.333	NXY-059
Selleck Chemicals	S2746	c1(C(C)(C)C#N)cc(C(=O)Nc2cc(Nc3ccc4c(C(=O)N(C)C=N4)c3)c(C)cc2)ccc1	C27H25N5O2	451.52	AZ 628
Selleck Chemicals	S2310	c1(O)ccc(c2c(O)ccc(CC=C)c2)cc1CC=C	C18H18O2	266.334	Honokiol
Selleck Chemicals	S2471	c1(OCC[N+](CC)(CC)CC)c(OCC[N+](CC)(CC)CC)c(OCC[N+](CC)(CC)CC)ccc1.[I-].[I-].[I-]	C30H60I3N3O3	891.529	Gallamine Triethiodide
Selleck Chemicals	S2325	c1(O)cc2c(C(=O)C(O)=C(c3c(O)cc(O)cc3)O2)c(O)c1.O	C15H12O8	320.251	Morin Hydrate
Selleck Chemicals	S2484	n1ccc(C(C=C(C#N)C(=O)N2)=C2C)cc1	C12H9N3O	211.219	Milrinone
Selleck Chemicals	S2341	C1C=C(C)CC[C@@H]2[C@H](OC(=O)C2=C)[C@H]3[C@](C)(O3)C1	C15H20O3	248.318	(-)-Parthenolide
Selleck Chemicals	S2496	c1cc(C=CC(=O)O)ccc1Cn2ccnc2	C13H12N2O2	228.247	Ozagrel
Selleck Chemicals	S2355	[C@@]([H])(CC(=O)O1)([C@@]2(C)[C@]([H])(C(C)(C)CCC2)CC3)[C@]13C	C16H26O2	250.376	Sclareolide
Selleck Chemicals	S2506	[C@@]1(C)(O)[C@H](O)[C@@H](C)C(=NOCOCCOC)[C@H](C)C[C@](O)(C)[C@H](O[C@@H]2O[C@H](C)C[C@H](N(C)C)[C@H]2O)[C@@H](C)[C@@H](O[C@H]3C[C@](OC)(C)[C@@H](O)[C@H](C)O3)[C@@H](C)C(=O)O[C@@H]1CC	C41H76N2O15	837.047	Roxithromycin
Selleck Chemicals	S2367	c1(OC)cc2c(C(Cc3cc(OC)c(OC)cc3)NCC2)cc1OC.Cl	C20H26ClNO4	379.878	Tetrahydropapaverine HCl
Selleck Chemicals	S2527	c1cc2c(C(=O)C(=C3O)[C@]([H])([C@@H](O)[C@]4([H])[C@@]3(O)C(=O)C(C(=O)N)=C(O)[C@@H]4N(C)C)C2=C)c(O)c1.Cl	C22H23ClN2O8	478.88	Methacycline HCl
Selleck Chemicals	S2380	c1(O)cc2c(C(=O)C=C(c3cc(O)c(OC)cc3)O2)c(O)c1	C16H12O6	300.263	Diosmetin
Selleck Chemicals	S2537	n1(c(C)ncc1[N+](=O)[O-])CC(O)C	C7H11N3O3	185.181	Secnidazole
Selleck Chemicals	S2394	c1(O)cc2c(C(=O)C[C@@H](c3ccc(O)cc3)O2)c(O)c1	C15H12O5	272.253	Naringenin
Selleck Chemicals	S2554	c1(O)c(O)c2c(C=CC(=O)O2)cc1	C9H6O4	178.142	Daphnetin
Selleck Chemicals	S2406	c1cc2c(C(=O)c3c(cc(C)cc3O)C2=O)c(O)c1	C15H10O4	254.238	Chrysophanic Acid
Selleck Chemicals	S2566	c1cc(C(CNC(C)C)O)cc(O)c1O.Cl	C11H18ClNO3	247.719	Isoprenaline HCl
Selleck Chemicals	S2417	[C@H]1(O)C[C@@]2(C)[C@]([H])(C(=O)C=C3[C@@]2([H])CC[C@@]4(C)[C@@]3(O)CC[C@H]4[C@](O)(C)[C@H](O)CCC(C)(O)C)C[C@H]1O	C27H44O7	480.634	20-Hydroxyecdysone
Selleck Chemicals	S2586	C(OC)(=O)C=CC(OC)=O	C6H8O4	144.125	Dimethyl Fumarate
Selleck Chemicals	S2455	[N+](C)(C)(C)CC(C)OC(=O)N.[Cl-]	C7H17ClN2O2	196.675	Bethanechol chloride
Selleck Chemicals	S2603	c1(O)cc2c(SC(=O)O2)cc1	C7H4O3S	168.17	Tioxolone
Selleck Chemicals	S2614	N1(C)CC(C(=O)OC)=CCC1.Br	C8H14BrNO2	236.106	Arecoline
Selleck Chemicals	S2759	c1c(C(=O)NO)cnc(N(Cc2sc3c(nc(c4cnc(OC)cc4)nc3N5CCOCC5)c2)C)n1	C23H24N8O4S	508.553	CUDC-907
Selleck Chemicals	S2624	n1c(N)c2n(c([C@H]3CC[C@H](C(O)=O)CC3)nc2c4cc5c(c(OC)ccc5)[nH]4)nc1	C21H22N6O3	406.438	OSI-027
Selleck Chemicals	S2773	N(c1ccccc1Br)C(N[C@@H]2CCN(c3ncc(C(F)(F)F)cc3)C2)=O	C17H16BrF3N4O	429.234	SB705498
Selleck Chemicals	S2634	c1(NC(=O)Nc2cc(C(C)(C)C)nn2c3ccc4c(cccn4)c3)ccc(Oc5ccnc(C(=O)NC)c5)cc1F	C30H28FN7O3	553.587	DCC-2036 (Rebastinib)
Selleck Chemicals	S2783	c1cc2c(nc(N3CCOC[C@@H]3C)nc2N4CCOC[C@@H]4C)nc1c5cccc(C(=O)NC)c5	C25H30N6O3	462.544	AZD2014
Selleck Chemicals	S2660	c1c(Cl)ccc(S(=O)(=O)[C@]2(c3cc(F)ccc3F)CC[C@H](CCC(O)=O)CC2)c1	C21H21ClF2O4S	442.904	MK-0752
Selleck Chemicals	S2794	[C@H]1(CO[P@](=O)(Oc2ccccc2)N[C@@H](C)C(=O)OC(C)C)O[C@@H](N3C=CC(=O)NC3=O)[C@@](F)(C)[C@@H]1O	C22H29FN3O9P	529.453	Sofosbuvir (PSI-7977, GS-7977)
Selleck Chemicals	S2671	c1(F)ccc(c2ccc(C=C3C(=O)NC(=O)S3)o2)c(O)c1	C14H8FNO4S	305.281	AS-252424
Selleck Chemicals	S2811	n1c(N)c2c(n(C(C)C)nc2c3ccc4c(nc(N)o4)c3)nc1	C15H15N7O	309.326	INK 128 (MLN0128)
Selleck Chemicals	S2685	c1c(O)c(C(=O)c2cc(OC)c(OCCN3CCOCC3)cc2)c(CC(=O)N(CCOC)CCOC)c(CC)c1O	C30H42N2O9	574.662	KW-2478

Selleck Chemicals	S3007	[C@@H]1(NC(N)=N)[C@H](NC(=O)C)[C@]([H])([C@@H]([C@@H](CO)O)O)OC(C(O)=O)=C1	C12H20N4O7	332.31	Zanamivir
Selleck Chemicals	S2699	n1cc(c2nc(N3CCOCC3)nc4c2CCN4S(=O)(=O)C)cnc1N	C15H19N7O3S	377.421	CH5132799
Selleck Chemicals	S3023	c1(CC(NO)=O)ccc(OCCCC)cc1	C12H17NO3	223.268	Bufexamac
Selleck Chemicals	S2719	c1cnc(Oc2ccc(Nc3nnc(c4cc(C)cs4)c5c3cccc5)cc2)c(c6ccnc(N)n6)c1	C28H21N7OS	503.578	AMG-900
Selleck Chemicals	S4001	c(cc(OC)c(OC)c1)(nccc2Oc3ccc(NC(=O)C4(CC4)C(=O)Nc5ccc(F)cc5)cc3)c12.C(O)(=O)C[C@@H](C(O)=O)O	C32H30FN3O10	635.593	Cabozantinib malate (XL184)
Selleck Chemicals	S2731	c1(OC2CCN(C)CC2)ccc(Nc3ncc4c(N(C5CCCC5)C(=O)N4C)n3)c(OC)c1	C24H32N6O3	452.549	AZ 3146
Selleck Chemicals	S6003	c1c(C(O)=O)cccc1c2nc(c3c(F)cccc3)on2	C15H9FN2O3	284.242	Ataluren (PTC124)
Selleck Chemicals	S2747	N1(CC(O)(C)C)C(C)=C(C(Nc2ncc(Oc3c(c4ncc3)ccc(OC)c4)cc2)=O)C(=O)N1c5ccccc5	C30H29N5O5	539.582	AMG-458
Selleck Chemicals	S2311	C1C[C@@]2(C)[C@]([H])([C@@H](O)C[C@]3([H])[C@@]2([H])CC[C@@]4(C)[C@@]3([H])CC[C@H]4[C@@H](CCC(=O)O)C)C[C@@H]1O	C24H40O4	392.572	Hyodeoxycholic acid (HDCA)
Selleck Chemicals	S2473	c1c(O)ccc([C@H](CC)[C@@H](c2ccc(O)cc2)CC)c1	C18H22O2	270.366	Hexestrol
Selleck Chemicals	S2326	c1(O)cc2c(C(=O)C(O)=C(c3cc(O)c(O)c(O)c3)O2)c(O)c1	C15H10O8	318.235	Myricetin
Selleck Chemicals	S2485	c1c(O)c2c(C(=O)c3c(c(NCCNCCO)ccc3NCCNCCO)C2=O)c(O)c1.Cl.Cl	C22H30Cl2N4O6	517.403	Mitoxantrone HCl
Selleck Chemicals	S2342	c1(O)cc(O)c(C(=O)CCc2ccc(O)cc2)c(O)c1	C15H14O5	274.269	Phloretin
Selleck Chemicals	S2497	[C@H]1([N+]2(C)CCCCC2)C[C@@]3(C)[C@@]([H])(CC[C@]4([H])[C@@]3([H])CC[C@@]5(C)[C@@]4([H])C[C@H]([N+]6(C)CCCCC6)[C@H]5OC(=O)C)C[C@@H]1OC(=O)C.[Br-].[Br-]	C35H60Br2N2O4	732.67	Pancuronium dibromide
Selleck Chemicals	S2356	C1=C(C(O)=O)C[C@@H](O)C(O)C1O	C7H10O5	174.151	Shikimic Acid
Selleck Chemicals	S2508	O([C@@H]1C[C@@]([H])(N(C)[C@@]2([H])C1)[C@]3([H])[C@@]2([H])O3)C(=O)[C@H](CO)c4ccccc4.Br	C17H22BrNO4	384.265	Scopolamine HBr
Selleck Chemicals	S2370	C1C[C@@]2(C)[C@@]([H])(CC[C@]3(C)[C@@]2([H])CC=C4[C@@]3(C)CCC5[C@]4([H])[C@H](C)[C@H](C)C[C@H]5C(O)=O)C(C)(C)[C@H]1O	C30H48O3	456.7	Ursolic Acid
Selleck Chemicals	S2528	C1CC(C2=CC(C)=CC(=O)N2O)CCC1	C12H17NO2	207.269	Ciclopirox
Selleck Chemicals	S2381	OC[C@@H](O)[C@@H](O)[C@H](O)[C@H](O)CO	C6H14O6	182.172	D-Mannitol
Selleck Chemicals	S2542	c1ccc(CCNC(=N)NC(N)=N)cc1.Cl	C10H16ClN5	241.721	Phenformin HCl
Selleck Chemicals	S2395	c1(C)cc2c(C(=O)c3c(cc(OC)cc3O)C2=O)c(O)c1	C16H12O5	284.263	Rheochrysidin
Selleck Chemicals	S2555	[C@@]1(C)(O)[C@H](O)[C@@H](C)C(=O)[C@H](C)C[C@@](C)(OC)[C@H](O[C@H]2[C@@H](O)[C@@H](N(C)C)C[C@@H](C)O2)[C@@H](C)[C@@H](O[C@H]3O[C@@H](C)[C@H](O)[C@@](OC)(C)C3)[C@@H](C)C(=O)O[C@@H]1CC	C38H69NO13	747.953	Clarithromycin
Selleck Chemicals	S2407	C1(=C)C[C@](O[C@]23[C@@]1([H])CC[C@H]2C)(O)[C@H](C(C)C)C3	C15H24O2	236.35	Curcumol
Selleck Chemicals	S2567	C1C[C@@]2(C)C([C@@H](C)C[C@]3([H])[C@@]2([H])CC[C@@]4(C)[C@@]3([H])CC[C@@]4(OC(=O)C)C(C)=O)=CC1=O	C24H34O4	386.524	Medroxyprogesterone acetate
Selleck Chemicals	S2420	C1N2[C@@]([H])([C@]3([H])C[C@@]([H])([C@@]4([H])C(CCCN4)=C3)C2)CCC1	C15H24N2	232.364	Aloperine
Selleck Chemicals	S2589	[C@@H]1(O)[C@H](O)[C@H](O)[C@@H](CO)N(CCO)C1	C8H17NO5	207.224	Miglitol
Selleck Chemicals	S2457	[C@H]1(O)[C@H](SC)O[C@H]([C@@H](NC([C@@H]2C[C@@H](CCC)CN2C)=O)C(Cl)C)[C@H](O)[C@@H]1O.Cl	C18H34Cl2N2O5S	461.444	Clindamycin HCl
Selleck Chemicals	S2604	C1C[C@@]2(C)C(=CC[C@]3([H])[C@@]2([H])CC[C@@]4(C)[C@@]3([H])CCC4=O)C[C@H]1O	C19H28O2	288.424	Dehydroepiandrosterone (DHEA)
Selleck Chemicals	S2615	c1(O)cc([C@@]([H])(O)CN)ccc1O.OC(=O)[C@]([H])(O)[C@@]([H])(O)C(=O)O.O	C12H19NO10	337.28	Noradrenaline bitartrate monohydrate
Selleck Chemicals	S2760	[C@H]1(CO)O[C@@H](c2ccc(C)c(Cc3ccc(c4ccc(F)cc4)s3)c2)[C@H](O)[C@@H](O)[C@@H]1O	C24H25FO5S	444.516	Canagliflozin
Selleck Chemicals	S2626	c1c(OC[C@H]2OCCNC2)c(NC(=O)Nc3ncc(C)nc3)cc(Br)c1C	C18H22BrN5O3	436.303	LY2603618
Selleck Chemicals	S2775	C(OC)(=O)Nc1[nH]c2c(ccc(C(c3sccc3)=O)c2)n1	C14H11N3O3S	301.32	Nocodazole

Selleck Chemicals	S2635	n1c(N2CCC(Cc3ccc(Cl)cc3)(N)CC2)c4c([nH]cc4)nc1	C18H20ClN5	341.838	CCT128930
Selleck Chemicals	S2784	c(ccn1CCNC(CC(C)(C)O)=O)(ncnc2Nc3cc(Cl)c(Oc4cc(C(F)(F)F)ccc4)cc3)c12	C26H25ClF3N5O3	547.957	TAK-285
Selleck Chemicals	S2661	c1(N2CC(OC3C2)CC3)c4c(n(C5CCC6(OCCO6)CC5)nc4)nc(c7ccc(NC(NC)=O)cc7)n1	C27H33N7O4	519.595	WYE-125132 (WYE-132)
Selleck Chemicals	S2795	c1cc(C(=O)Nc2ccc(F)cc2F)ccc1OCCCC	C17H17F2NO2	305.319	VU 0357121
Selleck Chemicals	S2673	C1(=O)C(C)=C2C(C(=O)N(C3CC3)C(=O)N2c4cccc(NC(=O)C)c4)=C(Nc5c(F)cc(I)cc5)N1C	C26H23FIN5O4	615.395	Trametinib (GSK1120212)
Selleck Chemicals	S2814	N1(C(Nc2nc(C)c(c3ccnc(C(C)(C)C(F)(F)F)c3)s2)=O)CCC[C@H]1C(N)=O	C19H22F3N5O2S	441.47	BYL719
Selleck Chemicals	S2686	c1c(c2cc(F)c(CN3CCOCC3)c(F)c2)c4c(ncc(c5cnn(C6CCNCC6)c5)n4)cc1.Cl.Cl	C27H30Cl2F2N6O	563.47	NVP-BSK805 2HCl
Selleck Chemicals	S3008	c1cc2c(Sc3c(cc(C(C(O)=O)C)cc3)CC2=O)cc1	C17H14O3S	298.356	Zaltoprofen
Selleck Chemicals	S2700	c1cc(c2cnc(CC(=O)NCc3ccccc3)cc2)ccc1OCCN4CCOCC4	C26H29N3O3	431.527	KX2-391
Selleck Chemicals	S3025	c1(CS(F)(=O)=O)ccccc1	C7H7FO2S	174.193	PMSF
Selleck Chemicals	S2720	c1(N(C)C)cc(C(=O)Nc2ccc(C)c(NC(=O)c3ccc(O)cc3)c2)ccc1	C23H23N3O3	389.447	ZM 336372
Selleck Chemicals	S4002	c1c(F)c(C[C@@H](N)CC(=O)N2CCn3c(nnc3C(F)(F)F)C2)cc(F)c1F.P(OO)(=O)=O.[H].O	C16H20F6N5O6P	523.324	Sitagliptin phosphate monohydrate
Selleck Chemicals	S2735	c1(c(c2cnn(C)c2)cn3)n3c(N)c(Br)c([C@@H]4CCCNC4)n1	C15H18BrN7	376.254	MK-8776 (SCH 900776)
Selleck Chemicals	S6005	c1cc(F)c(N(C(N)=O)c2ccc(C(=O)N)c(c3ccc(F)cc3F)n2)c(F)c1	C19H12F4N4O2	404.318	VX-702
Selleck Chemicals	S2748	c1cc(c2c(OC)cc(F)c(C(C)C)c2)c(CN3[C@H](C)[C@@H](c4cc(C(F)(F)F)cc(C(F)(F)F)c4)OC3=O)cc1C(F)(F)F	C30H25F10NO3	637.508	Anacetrapib (MK-0859)
Selleck Chemicals	S2312	[C@H]1(CO)OC(Oc2c(CC=C(C)C)c3c(C(=O)C(OC4[C@@H](O)[C@H](O)[C@@H](O)[C@H](C)O4)=C(c5ccc(OC)cc5)O3)c(O)c2)[C@H](O)[C@@H](O)[C@@H]1O	C33H40O15	676.662	Icariin
Selleck Chemicals	S2475	n1ccc(c2cnccc2)nc1Nc3c(C)ccc(NC(c4ccc(CN5CCN(C)CC5)cc4)=O)c3	C29H31N7O	493.603	Imatinib (STI571)
Selleck Chemicals	S2328	C(N(CC)c1c(ccc(C)n1)C2=O)=C2C(=O)O	C12H12N2O3	232.235	Nalidixic acid
Selleck Chemicals	S2486	C1CN(C(=N)NC(=N)N)CCO1.Cl	C6H14ClN5O	207.661	Moroxydine HCl
Selleck Chemicals	S2343	c1(O)c(C(=O)CCc2ccc(O)cc2)c(O[C@H]3[C@@H](O)[C@@H](O)[C@H](O)[C@@H](CO)O3)cc(O)c1	C21H24O10	436.409	Phlorizin
Selleck Chemicals	S2498	OCC(C)(C)[C@@]([H])(O)C(=O)NCCC(=O)O	C9H17NO5	219.235	D-Pantothenic acid
Selleck Chemicals	S2357	[C@H]1(CO)Oc2c(cc([C@@H]3Oc4c(c(O)cc(O)c4)C(=O)[C@@H]3O)cc2)O[C@@H]1c5cc(OC)c(O)cc5	C25H22O10	482.436	Silibinin
Selleck Chemicals	S2509	c1(C(O)CNC(C)C)ccc(NS(=O)(=O)C)cc1.Cl	C12H21ClN2O3S	308.825	Sotalol
Selleck Chemicals	S2373	[nH]1c2c(cccc2)c3c1[C@@]4([H])N(C[C@@]5([H])[C@@]([H])([C@@H](C(=O)OC)[C@@H](O)CC5)C4)CC3.Cl	C21H27ClN2O3	390.904	Yohimbine HCl
Selleck Chemicals	S2529	c1(O)cc(CCN)ccc1O.Cl	C8H12ClNO2	189.639	Dopamine HCl
Selleck Chemicals	S2384	[C@](O)(Cc1c2cc(O)c(O)c1)(COc3c4ccc(O)c3O)[C@]24[H]	C16H14O6	302.279	Hematoxylin
Selleck Chemicals	S2543	N(C(=O)[C@@]1(NC(=O)C(c2csc(N)n2)=NOC)[H])(C(C(=O)O)=C(CSC(=O)c3ccco3)CS4)[C@@]14[H].Cl	C19H18ClN5O7S3	560.024	Ceftiofur HCl
Selleck Chemicals	S2396	[C@H]1(CO)O[C@@H](OCCc2ccc(O)cc2)[C@H](O)[C@@H](O)[C@@H]1O	C14H20O7	300.304	Salidroside
Selleck Chemicals	S2556	c1cc(N(C)CCOc2ccc(CC3C(=O)NC(=O)S3)cc2)ncc1	C18H19N3O3S	357.427	Rosiglitazone
Selleck Chemicals	S2408	c1cc([C@H](NC(=O)C(C)=CC)[C@@H](O)C(=O)O[C@@H]2C(C)=C3C(C)(C)[C@](O)([C@@H](OC(=O)c4ccccc4)[C@@]5([H])[C@@](C)([C@H](O)C[C@@H]6[C@@]5(OC(C)=O)CO6)C(=O)[C@H]3OC(C)=O)C2)ccc1	C45H53NO14	831.901	Cephalomannine
Selleck Chemicals	S2569	c1(O)cc([C@@]([H])(O)CNC)ccc1.Cl	C9H14ClNO2	203.666	Phenylephrine HCl
Selleck Chemicals	S2422	c1cc2c(OC=C(c3ccccc3)C2=O)cc1OC(C)C	C18H16O3	280.318	Ipriflavone (Osteofix)
Selleck Chemicals	S2593	C(N1c2c(cc(Cl)cc2)C(O)CCC1)(=O)c3ccc(NC(c4c(C)cccc4)=O)cc3C	C26H25ClN2O3	448.941	Tolvaptan

Selleck Chemicals	S2458	c1c(Cl)c(NC2=NCCN2)c(Cl)cc1.Cl	C9H10Cl3N3	266.555	Clonidine HCl
Selleck Chemicals	S2605	C1(=O)C(C)=C(CCCCCCCCCCO)C(=O)C(OC)=C1OC	C19H30O5	338.439	Idebenone
Selleck Chemicals	S2617	N1(C[C@H](CO)O)C(=O)C2=C(N(C)C(=O)C(F)=C2Nc3c(F)cc(I)cc3)N=C1	C17H15F2IN4O4	504.227	TAK-733
Selleck Chemicals	S2761	c1cc2n(c(Cc3cc4c(nccc4)cc3)cn2)nc1c5cn(C)nc5	C20H16N6	340.381	NVP-BVU972
Selleck Chemicals	S2627	c1cc(Cn2c3c(cccc3)c4c2CCN(C)C4)ccc1C(=O)NO.Cl	C20H22ClN3O2	371.861	Tubastatin A HCl
Selleck Chemicals	S2776	S(Cc1ccccc1)CCC(CCCCC(=O)O)SCc2ccccc2	C22H28O2S2	388.587	CPI-613
Selleck Chemicals	S2636	c1c(c2c(C)nc(NC(=O)N3CCC[C@H]3C(N)=O)s2)nc(C(C)(C)C)s1	C17H23N5O2S2	393.527	A66
Selleck Chemicals	S2785	o1c(c2ccc(Cl)cc2)ccc1C(Nc3cc(OC)cc(OC)c3)=O	C19H16ClNO4	357.788	A-803467
Selleck Chemicals	S2663	c1(ccccc1OC)N2CCN(CCN(C(=O)C3CCCCC3)c4ncccc4)CC2.Cl.Cl.Cl	C25H37Cl3N4O2	531.946	WAY-100635 Maleate
Selleck Chemicals	S2796	C(N[C@H](c1ccccc1)C)(=O)C(C#N)=Cc2nc(Br)ccc2	C17H14BrN3O	356.217	WP1066
Selleck Chemicals	S2674	c1ccc(NC(=O)Nc2ccc(c3ccc(C([C@H]4[C@@H](C(=O)O)CCC4)=O)cc3)cc2)cc1	C26H24N2O4	428.48	A922500
Selleck Chemicals	S2820	c1(N2CCOCC2)ccc(Nc3ncc(Cl)c(Nc4ccccc4C(=O)NC)n3)c(OC)c1	C23H25ClN6O3	468.936	TAE226 (NVP-TAE226)
Selleck Chemicals	S2688	n1c(N)c(C(=O)c2c(OC)ccc(F)c2F)cnc1NC3CCN(S(=O)(C)=O)CC3	C18H21F2N5O4S	441.452	R547
Selleck Chemicals	S3012	c1(S(N)(=O)=O)cc(Nc2nccc(N(c3cc(c4cc3)nn(C)c4C)C)n2)ccc1C	C21H23N7O2S	437.518	Pazopanib
Selleck Chemicals	S2703	n1c(Nc2c(C(NC)=O)c(F)ccc2)c3c([nH]cc3)nc1Nc4cc5c(CCN5C(CN(C)C)=O)cc4OC	C27H29FN8O3	532.569	GSK1838705A
Selleck Chemicals	S3026	c1(O)ccc(C=Cc2cc(O)cc(O)c2)cc1O	C14H12O4	244.243	Piceatannol
Selleck Chemicals	S2722	c1cc(C(Nc2ccc3c(c(N)cc(C)n3)c2)=O)c(COc4ccc(CC)cc4)cc1.Cl	C26H26ClN3O2	447.957	JTC-801
Selleck Chemicals	S4075	c1c[n+]2c([S-][Zn+2]3([S-]c4[n+](cccc4)[O-]3)[O-]2)cc1	C10H8N2O2S2Zn	317.722	Zinc Pyrithione
Selleck Chemicals	S2736	c1(OCCN2CCCC2)ccc(Nc3ncc(C)c(Nc4cc(S(=O)(=O)NC(C)(C)C)ccc4)n3)cc1	C27H36N6O3S	524.678	TG101348 (SAR302503)
Selleck Chemicals	S7046	C1CC=C[C@@]2([H])[C@@]([H])(C[C@@H](O)C2)[C@H](O)C=CC(=O)O[C@@H](C)C1	C16H24O4	280.359	Brefeldin A
Selleck Chemicals	S2749	C(O)(=O)C=CC(O)=O.n1c(c2c3c(N(C)C(=O)N3c4ccc(N5CCNCC5)c(C(F)(F)F)c4)c1)ccc(c6ccc(OC)nc6)c2	C32H29F3N6O6	650.604	BGT226 (NVP-BGT226)
Selleck Chemicals	S2314	c1(O)cc2c(C(=O)C(O)=C(c3ccc(O)cc3)O2)c(O)c1	C15H10O6	286.236	Kaempferol
Selleck Chemicals	S2476	c1cc(N2CCN(c3ccc(N4C=NN(C(C)CC)C4=O)cc3)CC2)ccc(OC[C@H]5CO[C@@](c6c(Cl)cc(Cl)cc6)(Cn7ncnc7)O5)c1	C35H38Cl2N8O4	705.633	Itraconazole
Selleck Chemicals	S2334	C1C[C@@]2(C)[C@@]([H])(CC[C@]3(C)[C@@]2([H])CC=C4[C@@]3(C)CCC5[C@]4([H])CC(C)(C)C[C@H]5C(O)=O)C(C)(C)[C@H]1O	C30H48O3	456.7	Oleanolic Acid
Selleck Chemicals	S2487	c(C(=O)OC1)(c(O)c(CC=C(CCC(O)=O)C)c(OC)c2C)c12	C17H20O6	320.337	Mycophenolic acid
Selleck Chemicals	S2344	C1CCCN(C(=O)C=CC=Cc2ccc3c(OCO3)c2)C1	C17H19NO3	285.338	Piperine
Selleck Chemicals	S2499	c1ccc(OCC(C)N(Cc2ccccc2)CCCl)cc1.Cl	C18H23Cl2NO	340.287	Phenoxybenzamine HCl
Selleck Chemicals	S2359	c1(OC)ccc2c([C@@]34[C@]([H])(C=C(OC)C(=O)C3)[C@@H](N(C)CC4)C2)c1O	C19H23NO4	329.39	Sinomenine
Selleck Chemicals	S2510	[C@H]1(NC)[C@@H](O)[C@]2([H])[C@]([H])(O[C@]3(O)[C@@]([H])(O[C@H](C)CC3=O)O2)[C@@H](NC)[C@H]1O.Cl.Cl	C14H26Cl2N2O7	405.271	Spectinomycin HCl
Selleck Chemicals	S2374	c1(O)cc2c([nH]cc2CC(N)C(=O)O)cc1	C11H12N2O3	220.225	5-hydroxytryptophan (5-HTP)
Selleck Chemicals	S2531	c1(OC)cc(C=O)c(OC)cc1OC	C10H12O4	196.2	Asaraldehyde
Selleck Chemicals	S2385	c1cc(CCN(C)C)ccc1O	C10H15NO	165.232	Hordenine
Selleck Chemicals	S2545	[C@@]1([H])(C[C@@H](O)C2)N(C)[C@]2([H])[C@@]3([H])[C@]1([H])O3	C8H13NO2	155.194	Scopine
Selleck Chemicals	S2399	c1(O)cc2c(C(=O)[C@H](O)[C@@H](c3cc(O)c(O)c(O)c3)O2)c(O)c1	C15H12O8	320.251	Dihydromyricetin

Selleck Chemicals	S2557	c1ccc2c(cccc2CN(C)CC=CC#CC(C)(C)C)c1.Cl	C21H26ClN	327.891	Terbinafine HCl
Selleck Chemicals	S2409	[C@@](C)(C(=O)[C@H](O)C(=C1C)C(C)(C)[C@@](O)(C[C@@H]1O)[C@H]2OC(c3ccccc3)=O)([C@H](O)C[C@]4([H])[C@@]5(OC(=O)C)CO4)[C@]25[H]	C29H36O10	544.59	10-Deacetylbaccatin-III
Selleck Chemicals	S2574	c1c(O)c2c([C@](O)(C)[C@@](C(=C3O)C2=O)([H])C[C@]4([H])[C@@]3(O)C(=O)C(C(=O)N)=C(O)[C@@]4([H])N(C)C)cc1.Cl	C22H25ClN2O8	480.895	Tetracycline HCl
Selleck Chemicals	S2437	c1cc2c(CN3[C@]([H])(c4c(cc(OC)c(OC)c4)CC3)C2)c(OC)c1OC	C21H25NO4	355.427	Rotundine
Selleck Chemicals	S2594	C(N1CCCC1=O)C(NCCN(C(C)C)C(C)C)=O	C14H27N3O2	269.383	Pramiracetam
Selleck Chemicals	S2461	C(N1c2c(cccc2)NC1=O)CCN3CCC(N4C(=O)Nc5c4ccc(Cl)c5)CC3	C22H24ClN5O2	425.911	Domperidone
Selleck Chemicals	S2606	C1CC(=C([C@H](c2ccc(N(C)C)cc2)C[C@@]3(C)[C@@]4([H])CC[C@@]3(C#CC)O)[C@]45[H])C(CC5)=CC1=O	C29H35NO2	429.594	Mifepristone
Selleck Chemicals	S2619	c1ccc(COC(=O)N[C@@H](CC(C)C)C(=O)N[C@@H](CC(C)C)C(=O)N[C@H](C=O)CC(C)C)cc1	C26H41N3O5	475.621	MG-132
Selleck Chemicals	S2765	N1=C(C(NC)=O)c2c(c(O)c3n2[C@H](C)CN(CC)C3=O)C(=O)N1Cc4cc(Cl)c(F)cc4	C21H21ClFN5O4	461.874	MK-2048
Selleck Chemicals	S2629	c1(OC)cc(OC)c(Cl)cc1NC(=O)Nc2cc(C)on2	C13H14ClN3O4	311.721	PNU-120596
Selleck Chemicals	S2777	c1(C)cnc(c2c(Cl)cnc(N3CCN(C(CCS(=O)(=O)C)=O)CC3)c2)c(C)c1	C20H25ClN4O3S	436.955	PF-5274857
Selleck Chemicals	S2638	C(C(=O)c1c(c(c2c3c(c4c(cccc4)s3)ccc2)ccc1)O5)=C5N6CCOCC6	C25H19NO3S	413.488	NU7441 (KU-57788)
Selleck Chemicals	S2787	N1(C)c(c2C(O)=C(C(N(CC)c3ccccc3)=O)C1=O)cccc2Cl	C19H17ClN2O3	356.803	Laquinimod
Selleck Chemicals	S2664	C1CCCC(c2ccc(OC(C(O)=O)(C)CC)cc2)(c3ccc(OC(C(=O)O)(C)CC)cc3)C1	C28H36O6	468.582	Clinofibrate
Selleck Chemicals	S2797	C1c2c(ncc(Br)c2)[C@H](C3CCN(C(=O)CC4CCN(C(=O)N)CC4)CC3)c5c(cc(Cl)cc5Br)C1	C27H31Br2ClN4O2	638.822	Lonafarnib
Selleck Chemicals	S2677	c1ccc(C(c2ccccc2)C(O)CN3CCN(c4cccc(Cl)c4)CC3)cc1.Cl.Cl	C25H29Cl3N2O	479.87	BRL-15572
Selleck Chemicals	S2840	S=C1N(c2cc(F)c(C(=O)NC)cc2)C3(CCC3)C(=O)N1c4cc(C(F)(F)F)c(C#N)nc4	C21H15F4N5O2S	477.435	ARN-509
Selleck Chemicals	S2689	c(cc[nH]1)(cc(c2nc(N3CCOCC3)c4c(n(C5CCN(Cc6cnccc6)CC5)nc4)n2)cc7)c17	C28H30N8O	494.591	WAY-600
Selleck Chemicals	S3017	c1c(C(O)=O)c(OC(=O)C)ccc1	C9H8O4	180.157	Aspirin
Selleck Chemicals	S2711	c1(F)cc(CC(=O)N[C@@H](C)C(=O)N[C@@H]2C(=O)N(C)c3c(cccc3)c4c2cccc4)cc(F)c1	C26H23F2N3O3	463.476	YO-01027
Selleck Chemicals	S3031	c1cc2c(c(C)nc(CN3C(=O)c4c(nc(N5CCC[C@@H](N)C5)n4CC#CC)N(C)C3=O)n2)cc1	C25H28N8O2	472.542	Linagliptin
Selleck Chemicals	S2726	c1(N2C(=O)C(Br)=C(OCc3c(F)cc(F)cc3)C=C2C)cc(C(NC)=O)ccc1C	C22H19BrF2N2O3	477.299	PH-797804
Selleck Chemicals	S4119	c(cc(N1CCN(C)CC1)c(F)c2)(N(CC)C=C(C(=O)O)C3=O)c23.S(=O)(=O)(O)C.O.O	C18H28FN3O8S	465.494	Pefloxacin Mesylate Dihydrate
Selleck Chemicals	S2738	c1(ccccc1)CN2CCN(CC(=O)NN=Cc3c(O)c(CC=C)ccc3)CC2	C23H28N4O2	392.494	PAC-1
Selleck Chemicals	S7397	c1(cc(NC(=O)Nc2ccc(Oc3ccnc(C(NC)=O)c3)cc2)ccc1Cl)C(F)(F)F	C21H16ClF3N4O3	464.825	Sorafenib
Selleck Chemicals	S2750	C(NC1CCOCC1)(=O)c2ccc(c3nccc(c4c[nH]nc4c5ncccc5)c3)cc2	C25H23N5O2	425.482	GW788388
Selleck Chemicals	S2319	C1(=O)[C@H]2[C@@]3(O2)[C@@](C)(CC[C@@]4([H])[C@]3(C)C(=O)C[C@]5([H])[C@]4(COC(=O)C6)[C@@]6([H])OC5(C)C)[C@H](c7ccoc7)O1	C26H30O8	470.512	Limonin
Selleck Chemicals	S2479	[C@@H]1(O)[C@]([H])([C@H](NC([C@H]2N(C)C[C@H](CCC)C2)=O)[C@H](O)C)O[C@H](SC)[C@H](O)[C@H]1O.Cl	C18H35ClN2O6S	442.998	Lincomycin HCl
Selleck Chemicals	S2335	[C@@]1([H])([C@H]2O)[C@@](CO3)(C(CC4)[C@]5([C@@]23O)[C@H](O)[C@]4([H])C(=C)C5=O)[C@@H](O)CCC1(C)C	C20H28O6	364.433	Oridonin
Selleck Chemicals	S2489	C1C[C@@H](C(=O)N[C@@H](C(O)=O)Cc2ccccc2)CC[C@@H]1C(C)C	C19H27NO3	317.423	Nateglinide
Selleck Chemicals	S2346	c(C(=O)C(c1ccc(O)cc1)=CO2)(ccc(O)c3O[C@@H]4O[C@H](CO)[C@@H](O)[C@H](O)[C@H]4O)c23	C21H20O10	432.377	Puerarin
Selleck Chemicals	S2500	c1cc(CCC(=O)c2c(OCC(CNCCC)O)cccc2)ccc1	C21H27NO3	341.444	Propafenone HCl
Selleck Chemicals	S2362	c1cc(C(CNC)O)ccc1O	C9H13NO2	167.205	Synephrine

Selleck Chemicals	S2512	c1cnc(NC(C2=C(O)c3c(ccs3)S(=O)(=O)N2C)=O)cc1	C13H11N3O4S2	337.374	Tenoxicam
Selleck Chemicals	S2375	[C@@H]1(CO)[C@H](O)[C@H](O)[C@@H](O)[C@]([H])([C@]2([H])c3c(c(O)cc(CO)c3)C(=O)c4c2cccc4O)O1	C21H22O9	418.394	Aloin
Selleck Chemicals	S2533	c1cc([C@@](O)([H])[C@](C)([H])NCCc2ccc(O)cc2)ccc1O.Cl	C17H22ClNO3	323.814	Ritodrine HCl
Selleck Chemicals	S2386	c1cc2c(C(=C3C(=O)c4c(cccc4)N3)C(=O)N2)cc1	C16H10N2O2	262.263	Indirubin
Selleck Chemicals	S2547	[Br-].O.[C@@]1([H])(C[C@@H](OC(=O)C(O)(c2sccc2)c3sccc3)C4)[N+](C)(C)[C@]4([H])[C@H]5[C@@H]1O5	C19H24BrNO5S2	490.432	Tiotropium Bromide hydrate
Selleck Chemicals	S2401	c1(O)cc(CC(O)C([O-])=O)ccc1O.[Na+]	C9H9NaO5	220.155	Sodium Danshensu
Selleck Chemicals	S2559	C1C[C@@]2(C)C(CC[C@]3([H])[C@@]2([H])C(=O)C[C@@]4(C)[C@@]3([H])CC[C@@]4(O)C(=O)COC(=O)C)=CC1=O	C23H30O6	402.481	Cortisone acetate
Selleck Chemicals	S2410	[C@H]1(CO)O[C@@H](O[C@]23C[C@]([H])([C@]24COC(=O)c5ccccc5)[C@](O)(O[C@]46[H])C[C@@]3(C)O6)[C@H](O)[C@@H](O)[C@@H]1O	C23H28O11	480.462	Paeoniflorin
Selleck Chemicals	S2576	c1c(C)c(CC2=NCCN2)c(C)cc1C(C)(C)C.Cl	C16H25ClN2	280.836	Xylometazoline HCl
Selleck Chemicals	S2438	c1cc(C(O)CNC)ccc1O.Cl	C9H14ClNO2	203.666	Synephrine HCl
Selleck Chemicals	S2596	C1(O)C(O)C(OC(=O)CCCCCCCCCCCCCCC)C(SC)OC1C(NC(C2CC(CCC)CN2C)=O)C(Cl)C.Cl	C34H64Cl2N2O6S	699.853	Clindamycin palmitate HCl
Selleck Chemicals	S2466	c1cc2c(CC[C@]3([H])[C@@]2([H])CC[C@@]4(C)[C@@]3([H])C[C@@H](O)[C@H]4O)cc1O	C18H24O3	288.381	Estriol
Selleck Chemicals	S2607	c1(OC)cc(OC)c(C(CCCN2CCCC2)=O)c(OC)c1.Cl	C17H26ClNO4	343.846	Buflomedil HCl
Selleck Chemicals	S2620	c1cc(S(=O)(=O)c2cc3c(ncc(C(N)=O)c3Nc4cccc(OC)c4)c(C)c2)cc(C(=O)N(C)C)c1	C27H26N4O5S	518.584	GSK256066
Selleck Chemicals	S2767	n1c(c2nc1)c(N)ncn2C	C6H7N5	149.153	3-Methyladenine
Selleck Chemicals	S2630	c1ccc(OCCCN(CC(c2ccccc2)c3ccccc3)Cc4c(Cl)c(C(F)(F)F)ccc4)cc1CC(O)=O.Cl	C33H32Cl2F3NO3	618.513	GW3965 HCl
Selleck Chemicals	S2778	c1(Nc2ccc(Cl)cc2Cl)ncc(C(NCC3CCOCC3)=O)c(C(F)(F)F)n1	C18H17Cl2F3N4O2	449.254	GW842166X
Selleck Chemicals	S2639	c1c(C(=O)N)c(N[C@H]2CC[C@H](O)CC2)cc(n3c4c(C(=O)CC(C)(C)C4)c(C(F)(F)F)n3)c1	C23H27F3N4O3	464.481	SNX-2112 (PF-04928473)
Selleck Chemicals	S2789	n1c(N([C@@H]2[C@@H](C)CCN(C(CC#N)=O)C2)C)c3c([nH]cc3)nc1	C16H20N6O	312.37	Tofacitinib (CP-690550,Tasocitinib)
Selleck Chemicals	S2665	c1cc(OC(C)(C)C(=O)O)ccc1C2CC2(Cl)Cl	C13H14Cl2O3	289.154	Ciprofibrate
Selleck Chemicals	S2801	c1(OC)cc(CCc2[nH]nc(NC(c3ccc(N4C[C@@H](C)N[C@@H](C)C4)cc3)=O)c2)cc(OC)c1	C26H33N5O3	463.572	AZD4547
Selleck Chemicals	S2680	c1nc2c(c(c3ccc(Oc4ccccc4)cc3)nn2[C@@H]5CCCN(C(=O)C=C)C5)c(N)n1	C25H24N6O2	440.497	Ibrutinib (PCI-32765)
Selleck Chemicals	S2853	C1OCCN(CC(=O)N[C@@H](CCc2ccccc2)C(=O)N[C@@H](CC(C)C)C(=O)N[C@@H](Cc3ccccc3)C(=O)N[C@@H](CC(C)C)C(=O)[C@@]4(C)CO4)C	C40H57N5O7	719.91	Carfilzomib (PR-171)
Selleck Chemicals	S2690	c1(F)ccc(C(=O)N2C[C@@H](c3onc(c4ccc(F)cc4)n3)CCC2)cc1	C20H17F2N3O2	369.365	ADX-47273
Selleck Chemicals	S3018	c1(Nc2cccc(C(F)(F)F)c2)ncccc1C(O)=O	C13H9F3N2O2	282.218	Niflumic acid
Selleck Chemicals	S2713	C1(OC)=C(C(=O)C2=CC1=O)C[C@H](C)C[C@@H](OC)[C@H](O)[C@H](C)C=C(C)[C@H](OC(N)=O)[C@@H](OC)C=CC=C(C)C(=O)N2	C29H40N2O9	560.636	Geldanamycin
Selleck Chemicals	S3032	C(O)(=O)C(C)(OCc1c(c2n(Cc3ccccc3)n1)cccc2)C	C19H20N2O3	324.374	Bindarit
Selleck Chemicals	S2727	C(N1CCCCC1)C=CC(=O)Nc2cc3c(ncnc3Nc4ccc(F)c(Cl)c4)cc2OC	C24H25ClFN5O2	469.939	Dacomitinib (PF299804, PF299)
Selleck Chemicals	S4273	C(N)(=O)c1cccc(N)c1	C7H8N2O	136.151	3-Aminobenzamide
Selleck Chemicals	S2742	c1cc(c2cc3c(CCNC3=O)[nH]2)ccn1	C12H11N3O	213.235	PHA-767491
Selleck Chemicals	S8049	c1cc(Cn2c3c(cccc3)c4c2CCN(C)C4)ccc1C(=O)NO	C20H21N3O2	335.4	Tubastatin A
Selleck Chemicals	S2751	c1(c2c3c(c(C(=O)NC)nn3C)C(C)(C)C1)cnc(Nc4ccc(N5CCN(C)CC5)cc4)n2	C25H32N8O	460.575	Milciclib (PHA-848125)
Selleck Chemicals	S1333	c1(C(F)(F)F)ccc(OC(c2ccccc2)CCNC)cc1.Cl	C17H19ClF3NO	345.787	Fluoxetine HCl

Selleck Chemicals	S3070	N1(CC(N)=O)CCCC1=O	C6H10N2O2	142.156	Piracetam
Selleck Chemicals	S4256	c1nc(N2CCN(CCCCN3C(=O)CC4(CCCC4)CC3=O)CC2)ncc1.Cl	C21H32ClN5O2	421.964	Buspirone HCl
Selleck Chemicals	S1189	N1=C(CN2CCO[C@H](O[C@H](c3cc(C(F)(F)F)cc(C(F)(F)F)c3)C)[C@@H]2c4ccc(F)cc4)NC(=O)N1	C23H21F7N4O3	534.427	Aprepitant
Selleck Chemicals	S1441	C1C(O)(C(c2ccc(OC)cc2)CN(C)C)CCCC1.Cl	C17H28ClNO2	313.863	Venlafaxine
Selleck Chemicals	S2103	c1cc2c([C@@]3([C@H]45)[C@@](O)(CCC4=O)[C@H](N(CC6CC6)CC3)C2)c(O5)c1O.Cl	C20H24ClNO4	377.862	Naltrexone HCl
Selleck Chemicals	S3024	n1c(c2cccc(Cl)c2Cl)c(N)nc(N)n1	C9H7Cl2N5	256.091	Lamotrigine
Selleck Chemicals	S1438	[C@@H](OC(C)(C)O1)(CO[C@]2(COS(=O)(=O)N)[C@H]3OC(C)(C)O2)[C@@H]13	C12H21NO8S	339.362	Topiramate
Selleck Chemicals	S3175	C(NC)C[C@@H](Oc1ccccc1C)c2ccccc2.Cl	C17H22ClNO	291.816	Atomoxetine HCl
Selleck Chemicals	S4283	c(cccc1)(C=Cc2c(cccc2)C3=CCCN(C)C)c13.Cl	C20H22ClN	311.848	Cyclobenzaprine HCl
Selleck Chemicals	S1259	c1cc2c([C@H](CCNC(=O)CC)CC2)c3c1OCC3	C16H21NO2	259.343	Ramelteon
Selleck Chemicals	S1444	c1c2c(snc2N3CCN(CCc4cc5c(NC(=O)C5)cc4Cl)CC3)ccc1.Cl	C21H22Cl2N4OS	449.397	Ziprasidone HCl
Selleck Chemicals	S2452	c1(C(C(NC(C)(C)C)C)=O)cc(Cl)ccc1.Cl	C13H19Cl2NO	276.202	Amfebutamone HCl
Selleck Chemicals	S1445	c1(c(CS(=O)(=O)N)no2)c2cccc1	C8H8N2O3S	212.226	Zonisamide
Selleck Chemicals	S3180	[nH]1c(c2c(C[C@H]3CCCN3C)c1)ccc(CCS(c4ccccc4)(=O)=O)c2.Br	C22H27BrN2O2S	463.431	Eletriptan HBr
Selleck Chemicals	S4289	c1(Cl)cc(C(=O)NCCN(CC)CC)c(OC)cc1N.Cl	C14H23Cl2N3O2	336.257	MetoclopraMide HCl
Selleck Chemicals	S1280	C1CCN(CC)C1CNC(=O)c2c(OC)cc(N)c(S(=O)(=O)CC)c2	C17H27N3O4S	369.479	Amisulpride
Selleck Chemicals	S1614	c(sc(N)n1)(cc(OC(F)(F)F)cc2)c12	C8H5F3N2OS	234.198	Riluzole
Selleck Chemicals	S2459	N1c2c(cc(Cl)cc2)N=C(N3CCN(C)CC3)c4c1cccc4	C18H19ClN4	326.823	Clozapine
Selleck Chemicals	S1693	N1(c2c(cccc2)C=Cc3c1cccc3)C(N)=O	C15H12N2O	236.269	Carbamazepine
Selleck Chemicals	S3189	N1c(c2CC1=O)cccc2CCN(CCC)CCC.Cl	C16H25ClN2O	296.836	Ropinirole HCl
Selleck Chemicals	S1281	c1(OC)ccc(C(=O)N2CCCC2=O)cc1	C12H13NO3	219.237	Aniracetam
Selleck Chemicals	S1615	C(C)(N=C1N(CCCC1)C2=O)=C2CCN3CCC(c4noc5c4ccc(F)c5)CC3	C23H27FN4O2	410.484	Risperidone
Selleck Chemicals	S2460	[C@@H]1(NCCC)Cc2c(nc(N)s2)CC1	C10H17N3S	211.327	Pramipexole
Selleck Chemicals	S1747	C(C)(NC(C)=C(C(=O)OCCOC)C1c2cc([N+]([O-])=O)ccc2)=C1C(=O)OC(C)C	C21H26N2O7	418.44	Nimodipine
Selleck Chemicals	S3199	c1ccc(O[C@@H]([C@@]2([H])CNCCO2)c3ccccc3)c(OCC)c1.CS(=O)(O)=O	C20H27NO6S	409.496	Reboxetine mesylate
Selleck Chemicals	S1330	c1c(C(COC(=O)N)COC(=O)N)cccc1	C11H14N2O4	238.24	Felbamate
Selleck Chemicals	S1920	c1(F)ccc(C(CCCN2CCC(O)(c3ccc(Cl)cc3)CC2)=O)cc1	C21H23ClFNO2	375.864	Haloperidol
Selleck Chemicals	S2493	N1c2c(cccc2)N=C(N3CCN(C)CC3)c4c1sc(C)c4	C17H20N4S	312.433	Olanzapine
Selleck Chemicals	S1898	c1cc2c(N=CC2C(=O)OC3C[C@]([H])(N(C)[C@]4([H])C3)CC4)cc1.Cl	C17H21ClN2O2	320.814	Tropisetron
Selleck Chemicals	S3212	C(NCCN1CCOCC1)(=O)c2ccc(Cl)cc2	C13H17ClN2O2	268.739	Moclobemide (Ro 111163)
Selleck Chemicals	S1332	c1cc2c(C(=O)N(C)Cc3n2cnc3C(OCC)=O)cc1F	C15H14FN3O3	303.288	Flumazenil
Selleck Chemicals	S1975	c1cc2c(NC(=O)CC2)cc1OCCCCN3CCN(c4cccc(Cl)c4Cl)CC3	C23H27Cl2N3O2	448.385	Aripiprazole
Selleck Chemicals	S2541	C(CCN(C)C)N1c2c(cccc2)CCc3c1cc(Cl)cc3.Cl	C19H24Cl2N2	351.313	Clomipramine HCl
Selleck Chemicals	S2456	c1ccc2c(Sc3c(cc(Cl)cc3)N2CCCN(C)C)c1.Cl	C17H20Cl2N2S	355.325	Chlorpromazine HCl

Selleck Chemicals	S4058	C1CN(CCC)[C@H](C(Nc2c(C)cccc2C)=O)CC1.Cl	C17H27ClN2O	310.862	Ropivacaine HCl
Selleck Chemicals	S1356	C1(=O)CCCN1[C@@H](CC)C(=O)N	C8H14N2O2	170.209	Levetiracetam
Selleck Chemicals	S2016	C1c2c(cccc2)C3N(CCN(C)C3)c4c1cccn4	C17H19N3	265.353	Mirtazapine
Selleck Chemicals	S3005	[C@@H]1(c2ccc(F)cc2)CCNC[C@H]1COc3ccc4c(OCO4)c3.Cl	C19H21ClFNO3	365.826	Paroxetine HCl
Selleck Chemicals	S2525	C1(c2ccccc2)(c3ccccc3)C(=O)NC(=O)N1	C15H12N2O2	252.268	Phenytoin
Selleck Chemicals	S4202	Cl.c1cc(OC)c(OC)cc1C(C(C)C)(C#N)CCCN(CCc2cc(OC)c(OC)cc2)C	C27H39ClN2O4	491.063	Verapamil HCl
Selleck Chemicals	S1391	N1(C(=O)N)c2c(cccc2)CC(=O)c3c1cccc3	C15H12N2O2	252.268	Oxcarbazepine
Selleck Chemicals	S2084	c1cc2c(cccc2)c(O[C@H](c3cccs3)CCNC)c1.Cl	C18H20ClNOS	333.876	Duloxetine HCl
Selleck Chemicals	S3021	n1(c2ccc(Cl)cc2Cl)c(c3ccc(Cl)cc3)c(C)c(C(NN4CCCCC4)=O)n1	C22H21Cl3N4O	463.787	Rimonabant
Axon Medchem	1255	COc1cccc(c1c1cc(nn1c1ccc(cc1C(C)C)C(=O)N(CCCN(C)C)C)C(=O)NC1(C(=O)O)C2CC3CC1CC(C2)C3)OC	C39H51N5O6	685.85	SR 142948
Axon Medchem	2111	ONC(=O)C[C@H](C(=O)N[C@@H](C(C)(C)C)C(=O)N[C@@H](c1ccccc1)C)CCCc1ccc(c(c1)C)c1ccccc1	C34H43N3O4	557.72	UK 356618
Cayman Chemical	10879	O=C(Oc1ccccc1C(C)C)NCCc1ccc2c(c1)cccc2	C22H23NO2	333.42	JW 480
Cayman Chemical	13942	Nc1cccc(c1)Cn1ncc2c(c1=O)n(C)c1c2sc(c1)S(=O)C	C17H16N4O2S2	372.46	ML-265
Cayman Chemical	15016	COc1ccc2c(c1)ccc(c2)OCC1(CN(C1)C(=O)c1ccc(cc1)F)C(=O)O	C23H20FNO5	409.41	PF-04418948
Cayman Chemical	16248	CCOC(=O)[C@H](CCC(=O)N[C@H](C(=O)N[C@H](c1ccccc1)C(=O)OCC)CSCc1ccccc1)N	C27H35N3O6S	529.65	Ezatiostat
Cayman Chemical	17924	O=C1CCCC(=O)C1C(=O)c1ccc(cc1[N+](=O)[O-])C(F)(F)F	C14H10F3NO5	329.23	Nitisinone
Chem-Impex	22427	O=C(C(=O)c1ccc(cc1)Cl)c1ccc(cc1)Cl	C14H8Cl2O2	279.12	4,4'-Dichlorobenzil
Cayman Chemical	9001740	CCCCCCCCCCCCCCC(=O)NCCO	C17H35NO2	285.47	Pentadecanoyl Ethanolamide
Cayman Chemical	11309	CC[C@@H]1/C=C(\C)/C[C@H](C)C[C@H](OC)[C@H]2O[C@](O)([C@@H](C[C@@H]2OC)C)C(=O)C(=O)N2[C@H](C(=O)O[C@@H]([C@@H]([C@H](CC1=O)O)C)/C(=C/[C@@H]1CC[C@H]([C@@H](C1)OC)O)/C)CCC	C43H69NO12	792	Ascomycin
Axon Medchem	1269	COc1cc(C(C)C)c2c(c1)S(=O)(=O)N(C2=O)COc1cc(=O)n2c(n1)c(OCCN1CCCCC1)ccc2	C27H32N4O7S	556.63	SSR 69071
Axon Medchem	2118	OC(=O)[C@H]1C[C@@H]1C(=O)c1ccc(c(c1)Cl)Cl	C11H8Cl2O3	259.09	UPF 648
Cayman Chemical	11332	CO[C@@H]1[C@@H](CC[C@@]2([C@H]1[C@@]1(C)O[C@@H]1CC=C(C)C)CO2)OC(=O)/C=C/C=C/C=C/C=C/C(=O)O	C26H34O7	458.54	Fumagillin
Cayman Chemical	14094	CCCc1cc(C)[nH]c(=O)c1CNC(=O)c1cc(cc2c1cnn2C(C)C)c1ccnc(c1)N1CCN(CC1)C	C31H39N7O2	541.69	GSK343
Cayman Chemical	15028	CCOC(=O)N1[C@H](CC)C[C@@H](c2c1ccc(c2)C(F)(F)F)N(C(=O)OC)Cc1cc(cc(c1)C(F)(F)F)C(F)(F)F	C26H25F9N2O4	600.47	Torcetrapib
Cayman Chemical	16271	COc1ccc(cc1)C[C@@H](C(=O)N[C@H](C(=O)[C@@]1(C)CO1)Cc1ccccc1)NC(=O)[C@@H](NC(=O)CN1CCOCC1)C	C31H40N4O7	580.67	ONX 0914
Cayman Chemical	17995	CCCN1CCN(CC1)c1ccc(cc1)C(=O)NC1(CCCCC1)C(=O)NCC#N	C23H33N5O2	411.54	Balicatib
AstaTech	41123	OC(=O)CNC(=O)[C@@H](Cc1ccccc1)CN1CC[C@@]([C@H](C1)C)(C)c1cccc(c1)O	C25H32N2O4	424.53	alvimopan
Cayman Chemical	9002002	Clc1ccc2c(c1)cc(o2)C(=O)NNS(=O)(=O)c1ccccc1C(F)(F)F	C16H10ClF3N2O4S	418.77	BCATc Inhibitor 2
Axon Medchem	1125	O[C@@H]([C@@H](NC(=O)c1cc(cc(c1)N(S(=O)(=O)C)C)C(=O)N[C@@H](c1ccccc1)C)Cc1ccccc1)CNC1CC1	C31H38N4O5S	578.72	BACE-1inhibitor
Axon Medchem	1383	OC(=O)C(=O)c1cn(c2c1cc(cc2)c1ccc(cc1)OC(F)(F)F)Cc1ccccc1	C24H16F3NO4	439.38	Tiplaxtinin
CHESS Fine Organic	2132	Oc1ccc(cc1)c1coc2c(c1=O)ccc(c2O)O	C15H10O5	270.24	7,8-Dihydroxy-3-(4-hydroxy-phenyl)-chromen-4-one
Cayman Chemical	11931	O=C(OC(C)(C)C)CN(Cc1ccc(s1)[N+](=O)[O-])Cc1ccc(cc1)Cl	C18H21ClN2O4S	396.89	GSK4112?
Cayman Chemical	14406	CCN(c1nc(N)c(nc1Cl)C(=O)NC(=N)N)C(C)C	C11H18ClN7O	299.76	5-(N-ethyl-N-isopropyl)-Amiloride

Cayman Chemical	15217	O[C@H]1C[C@@H](C[C@H]1COS(=O)(=O)N)n1ccc2c1ncnc2N[C@H]1CCc2c1cccc2	C21H25N5O4S	443.52	MLN4924
Cayman Chemical	16480	CCN1CCN(CC1)c1nc2CCCCCCc2c(c1)c1ccc(cc1)F	C23H30FN3	367.5	Blonanserin
Cayman Chemical	17999	CCN(c1ccc(cc1)/C=N/NC(=O)c1ccc(cc1)O)CC	C18H21N3O2	311.38	DY 131
AstaTech	41281	Brc1csc(c1)COc1nc(N)nc2c1nc[nH]2	C10H8BrN5OS	326.17	LOMEGUATRIB
Cayman Chemical	10007713	CCOC(=O)/C=C/[C@@H](NC(=O)[C@@H](NC(=O)[C@H](C(C)C)NC(=O)c1cccc(c1C)O)CO)CC(C)C	C26H39N3O7	505.6	HMB-Val-Ser-Leu-VE
Key Organics	HS-2008	OC[C@@H](C(=O)N[C@H](C(=O)N[C@@H](C(=O)N[C@H](C(=O)N[C@H](C(=O)N1CCC[C@H]1C(=O)N[C@@H](C(=O)N)C)CCCNC(=N)N)CC(C)C)CCCNC(=O)N)Cc1ccc(cc1)O)NC(=O)[C@@H](Cc1cccnc1)NC(=O)[C@@H](Cc1ccc(cc1)Cl)NC(=O)[C@@H](Cc1ccc2c(c1)cccc2)NC(=O)C	C70H92ClN17O14	1431.04	Cetrorelix
Axon Medchem	1755	CCOC(=O)N[C@@H]1CC[C@@H]2[C@@H](C1)C[C@@H]1[C@H]([C@H]2/C=C/c2ccc(cn2)c2cccc(c2)F)[C@H](OC1=O)C	C29H33FN2O4	492.58	SCH 530348
Axon Medchem	2148	C[C@@H]1CN(C[C@H]1c1[nH]c(=O)c2c(n1)n(nc2)C1CCOCC1)Cc1ncccn1	C20H25N7O2	395.46	PF 04447943
Cayman Chemical	12018	Clc1cc(COC(=O)N2CCN(CC2)CCC(=O)c2ccc3c(c2)oc(=O)[nH]3)cc(c1)Cl	C22H21Cl2N3O5	478.33	PF-8380
Cayman Chemical	14531	CCCCCc1ccc(cc1)/C=C/C(=O)Nc1ccccc1C(=O)O	C21H23NO3	337.41	N-(p-amylcinnamoyl) Anthranilic Acid
Cayman Chemical	15362	OC(=O)/C(=N/Nc1scc(n1)c1ccc(c(c1)Cl)Cl)/Cc1ccccc1[N+](=O)[O-]	C18H12Cl2N4O4S	451.28	4EGI-1
Acros Organics (US)	17397	CCCCCCCCCCC(=O)O	C11H22O2	186.29	Undecanoic acid, 99%
Cayman Chemical	18002	Oc1cc2c3SCCNC(=O)c3sc2cc1	C11H9NO2S2	251.32	kb NB 142-70
AstaTech	42077	C[C@H](Nc1ncnc2c1scc2C)CN1CCN(CC1)S(=O)(=O)c1ccc(c(c1)Cl)Cl	C20H23Cl2N5O2S2	500.46	LPA2 ANTAGONIST 1
Cayman Chemical	10008671	CCCc1nn(c2c1[nH]c(nc2=O)c1cc(ccc1OCC)S(=O)(=O)N1CCN(CC1)C)C	C22H30N6O4S	474.58	Sildenafil
Axon Medchem	2097	COc1cccc(c1)c1cn(c2c1c(N)ncn2)c1ccc(cc1)CCN1CCC(CC1)O	C26H29N5O2	443.54	CGP77675
Axon Medchem	1792	Clc1cccc(c1)OCc1nn(c2c1c(N)ncn2)C1CCOCC1	C17H18ClN5O2	359.81	PF 4800567
Axon Medchem	2270	Brc1cccc(c1)NC(=O)c1cccc(c1)S(=O)(=O)N1CCCCCC1	C19H21BrN2O3S	437.35	AK 7
Cayman Chemical	12046	COC(=O)c1cnn(c1/C=N/NC(=S)NC(C)(C)C)c1ccc(cc1F)F	C17H19F2N5O2S	395.43	CID-2745687
Cayman Chemical	14603	Fc1nccc(c1)c1ccc(c(c1)C(=O)Nc1cccnc1)OCc1ccccc1	C24H18FN3O2	399.42	GSK2578215
Cayman Chemical	15425	CCCSc1nc(N[C@@H]2C[C@H]2c2ccc(c(c2)F)F)c2c(n1)n(nn2)[C@@H]1C[C@@H]([C@H]([C@H]1O)O)OCCO	C23H28F2N6O4S	522.57	Ticagrelor
Cayman Chemical	17501	CCCCCCCC(=O)c1c(O)cc(cc1CC(=O)OCC)O	C18H26O5	322.4	Cytosporone B
Cayman Chemical	18377	COc1cccc(c1)c1ccc(c(c1)F)NC(=O)C1=C(CCC1)C(=O)O	C20H18FNO4	355.36	Vidofludimus
AstaTech	55318	OCCN(CCCOc1ccc2c(c1)ncnc2Nc1n[nH]c(c1)CC(=O)Nc1cccc(c1)F)CC	C26H30FN7O3	507.56	AZD1152-HQPA
Cayman Chemical	10010206	CCc1c(c2ccccc2)c(nn1c1ccccc1c1cccc(c1)OCC(=O)O)c1ccccc1	C31H26N2O3	474.55	BMS309403
Axon Medchem	1279	O=C(/C=C/c1cccnc1)NCCCCC1CCN(CC1)C(=O)c1ccccc1	C24H29N3O2	391.5	FK866
Axon Medchem	1907	Clc1cc2nc(c(nc2cc1Cl)NC(C)(C)C)S(=O)(=O)C	C13H15Cl2N3O2S	348.25	GLP-1R agonist DMB
Axon Medchem	2337	N#C/C(=C\c1ccc(c(c1)OC)OCc1ccc(cc1C(F)(F)F)C(F)(F)F)/C(=O)Nc1nnc(s1)C(F)(F)F	C23H13F9N4O3S	596.42	XCT 790
Cayman Chemical	12072	FC(C(C(F)(F)F)(c1ccc(cc1)NS(=O)(=O)c1cccs1)O)(F)F	C13H9F6NO3S2	405.34	SR 3335
Cayman Chemical	14745	CCCCCCCCn1cc(c2c1cccc(c2)c1cccc(c1)C)CC(=O)N	C25H32N2O	376.53	Cysmethynil
Cayman Chemical	15487	Clc1ccc(c(c1)/C(=N/NC(=O)c1cccc(c1)S(=O)(=O)N1CCOCC1)/C)O	C19H20ClN3O5S	437.9	SP2509
Cayman Chemical	17505	Fc1ccc(cn1)NC(=O)[C@]1(C)CCCN1c1nc(Nc2n[nH]c(c2)C2CC2)c2n(n1)ccc2	C23H24FN9O	461.49	BMS 754807
Cayman Chemical	18503	NC(=O)Nc1cc(Cl)ccc1OCC(=O)N1CCN(C[C@H]1C)Cc1ccc(cc1)F	C21H24ClFN4O3	434.89	BX 471

AstaTech	70704	CCCCC([C@@]1(O)CC[C@H]2[C@H](O1)CC(=O)[C@@H]2CCCCCCC(=O)O)(F)F	C20H32F2O5	390.46	LUBIPROSTONE
Cayman Chemical	10010740	O=C1CC(=NN1c1ccccc1)c1ccc(cc1)[N+](=O)[O-]	C15H11N3O3	281.27	CAY10550
Cayman Chemical	16257	Oc1ccc(cc1)C[C@H]1C(=O)N(C[C@H]2N1C(=O)CCN2C(=O)NCc1ccccc1)Cc1cccc2c1cccc2	C33H32N4O4	548.6	ICG-001
Axon Medchem	1985	CN[C@H](C(=O)N[C@H]1CN(CC[C@@H]2N(C1=O)[C@@H](CC2)C(=O)NC(c1ccccc1)c1ccccc1)C(=O)CC(C)C)C	C32H43N5O4	561.71	AT 406
Advanced ChemBlocks	10259	OC(=O)c1ccc(nc1)C#Cc1ccc2c(c1)C(C)(C)CCS2	C19H17NO2S	323.41	AGN 190299
Cayman Chemical	13217	N#Cc1cccc(c1c1[nH]c2c(n1)c1ccc(cc1c1c2cccc1)Cl)C#N	C23H11ClN4	378.81	MF63
Acros Organics (US)	14772	Clc1ccc(c(c1)Cl)O	C6H4Cl2O	163	2,4-Dichlorophenol, 99%
Cayman Chemical	16207	C/C=C(\C(=O)O[C@H]1C(=C[C@]23[C@]1(O)[C@H](O)C(=C[C@H](C3=O)[C@H]1[C@@H](C[C@H]2C)C1(C)C)CO)C)/C	C25H34O6	430.53	Ingenol-3-angelate
Cayman Chemical	17703	CCCCNc1ncc(c(n1)N[C@@H]1CC[C@H](CC1)O)c1ccc(cn1)CN1CCOCC1	C24H36N6O2	440.58	UNC2250
Chem-Impex	21985	O=c1c(O)c(oc2c1cccc2)c1ccccc1	C15H10O3	238.24	3-Hydroxyflavone
AstaTech	78259	Fc1cccc(c1)c1ccc(cn1)C(=O)NC1CCN(CC1)CC(F)(F)F	C19H19F4N3O	381.37	HPGDS-INHIBITOR-1
Cayman Chemical	10016	O=C[C@@H](NC(=O)[C@@H](NC(=O)[C@H](C(C)C)NC(=O)[C@H](Cc1ccc(cc1)O)NC(=O)C)C)CC(=O)O	C23H32N4O8	492.5	Ac-YVAD-CHO
Cayman Chemical	15215	COc1ccc2c(c1)[nH]c1c2C[C@@H]2N([C@H]1CC(C)C)C(=O)[C@@H](NC2=O)CCC(=O)OC(C)(C)C	C26H35N3O5	469.6	ko143
Axon Medchem	1992	NC(=O)c1cnc(nc1NCc1ccccc1)NCCc1ccc(c(c1)Cl)O	C20H20ClN5O2	397.86	AS 1517499
Advanced ChemBlocks	10265	O=C(N1CCC[C@](C1)(Cc1ccccc1)C(=O)N(N(C)C)C)[C@H](NC(=O)C(N)(C)C)Cc1c[nH]c2c1cccc2	C31H42N6O3	546.7	Anamorelin
Cayman Chemical	13243	Brc1ccc(c(c1)/C=N/NC(=O)C(Sc1nc2c([nH]1)cccc2)C)O	C17H15BrN4O2S	419.3	KH7
Cayman Chemical	14806	CCOCc1nc2c(n1CC(O)(C)C)c1ccccc1nc2N	C17H22N4O2	314.38	R-848
Cayman Chemical	16239	COc1ccc(cc1NS(=O)(=O)c1cc(Cl)cc(c1O)Cl)c1ccccc1	C19H15Cl2NO4S	424.3	BMS 303141
Cayman Chemical	17737	CNC(=O)c1ccc(cc1)Nc1ncc(c(n1)NCc1nccnc1N(S(=O)(=O)C)C)C(F)(F)F	C20H21F3N8O3S	510.49	Defactinib
Chem-Impex	22028	O=c1cc(oc2c1ccc1c2cccc1)c1ccccc1	C19H12O2	272.3	a-Naphthoflavone
ChemBridge	4403033	OC(=O)CCn1nnc2c1cccc2	C9H9N3O2	191.19	3-(1H-1,2,3-benzotriazol-1-yl)propanoic acid
Cayman Chemical	17642	CC(OC(=O)N1CCC(CC1)Oc1ncnc2c1cnn2c1ccc(cc1F)S(=O)(=O)C)C	C21H24FN5O5S	477.5	APD668
Selleck Chemicals	S7488	Cc1cc(ccc1Nc1nc(NC2CCCCC2)c2c(n1)[nH]cn2)N1CCOCC1	C22H29N7O	407.91	MPI-0479605-HCL
Tocris	3411	CC1(C)CCC(C)(C)C2=CC(=CC=C12)C3(OCCO3)C4=CC=C(C=C4)C(O)=O	C24H28O4	380.477	SR 11237; SR-11237
Tocris	3706	NC1=N[NH]C2=C1C=C(N=N2)C3=C4C=CC=C[N]4N=C3C5=CC=CC=C5	C18H13N7	327.343	FR 180204; 5-(2-PHENYLPYRAZOLO[1,5-A]PYRIDIN-3-YL)-1H-PYRAZOLO[3,4-C]PYRIDAZIN-3-AMINE; FR-180204
Tocris	4088	CC1=CC(=C(C)[N]1C2=CC=C(F)C=C2)C(=O)CN3CCCC3	C18H21FN2O	300.371	IU1; IU-1

Tocris	4349	CN1CCN(CC1)C2=NC3=CC=CC=C3NC4=C2C=C(C)S4	C17H20N4S	312.432	olanzapine ODT; Zyprexa 15mg; Lanzapin; Zyprexa; LY-170053; Ran-olanzapine Ir; Zyprexa - 7.5mg; Deprex; Lapozan; Olanzapine-odt; Zyprexa Velotab; Zyprexa - 10mg; Ipg-olanzapine ODT; Olanzapine and Fluoxetine; Act Olanzapine ODT; Apisco; Sandoz Olanzapine ODT; Act Olanzapine; Egolanza; Mar-olanzapine ODT; Teva-olanzapine; Van-olanzapine; CO Olanzapine; Jolyon-MD; Zyprexa Zydis; Ran-olanzapine ODT; Apo-olanzapine ODT; Nu-olanzapine; Caprilon; Olanzapine; Ipg-olanzapine; Olansek; 2-methyl-4-(4-methyl-1-piperazinyl)-10H-thieno[2,3-b][1,5]benzodiazepine; Jamp Olanzapine Fc; C07322; Olanzapinum; Accel-olanzapine; Ran-olanzapine; Abbott-olanzapine ODT; Elynza; PMS-olanzapine; Mar-olanzapine; OLANZAPINE; Zyprexa Intramuscular; Sandoz Olanzapine; PMS-olanzapine ODT; Mylan-
Tocris	3676	CC(F)(F)CN1CCOC2=C([N](N=C2C1=O)C3=CC=CC=C3Cl)C4=CC=C(Cl)C=C4	C21H17Cl2F2N3O2	452.281	PF-514273
J & W PharmLab LLC	75R0522S	NC(=O)c1cccc2c1nc([nH]2)[C@]1(C)CCCN1	C13H16N4O	317.21	(S)-2-(2-Methylpyrrolidin-2-yl)-1H-benimidazole-4-carboxamide
Cayman Chemical	18010	Cc1cnc(nc1NCc1ccc(cc1)n1nncc1)c1ccccc1C(C)C	C23H24N6	384.48	ML-323
Cayman Chemical	17034	OC[C@H]1CCCN1Cc1ccc(cc1)COc1cc(C)cc(c1)CS(=O)(=O)c1ccccc1	C27H31NO4S	465.6	PF-543
Medchem Express	HY-15846	NC(=O)C[C@@H]1N=C(c2ccc(cc2)Cl)c2c(n3c1nnc3C)sc(c2C)C	C19H18ClN5OS	399.9	CPI-203
Medchem Express	HY-10299	CN(CC(=O)NC[C@@H](NC(=O)c1ccc(c(c1)Cl)OC(C)C)Cc1ccc(cc1)c1nc2n(c1)cccc2[C@@H](O)C)C	C32H38ClN5O4	592.13	GSK-923295
Tocris	3507	CC1(C)CCC(C)(C)C2=C1C=CC(=C2)NC(=O)C3=CC=C(C=C3)C(O)=O	C22H25NO3	351.439	AM 80; Tamibaro; Tamibarotene; OMS-0728; tamibarotene; TM-411; TOS-80; TOS-80T; TAMIBAROTENE; retinobenzoic acid; Amnoid; Amnoleuk; INNO-507; NSC-608000; D01418; Amnolake; Am 80; AM-80; Z-208; C12864

Tocris	3743	ClC1=CC=C(CCCOCCCN2CCCCC2)C=C1	C17H26ClNO	295.847	BF 2649 hydrochloride; pitolisant; TIPROLISANT; Pitolisant hydrochloride; Tiprolisant; Pitolisant; pitolisantum; BF-2649; Tiprolisant hydrochloride; FUB-649; Wakix; BF2.649
Tocris	4100	CC1=C(C=C([N]1CCCN2CCOCC2)C3=CC=CC=C3)C(=O)NC4=CC=CC(=C4)C(F)(F)F	C26H28F3N3O2	471.515	HC 067047; HC-067047
Tocris	4357	CC1=CC=C(C=C1)C23CNCC2C3	C12H15N	173.254	Bicifadine hydrochloride; Bicifadine; CL-220075; BICIFADINE HYDROCHLORIDE; BICIFADINE; DOV-220075; NIH-9542; D03110; Bicifadine HCl; MCV-4147
Tocris	965	NC1=C2CCCCC2=NC3=CC=CC=C13	C13H14N2	198.264	Tacrine; Cognex; C01453; 1,2,3,4-tetrahydroacridin-9-amine; TACRINE HYDROCHLORIDE; Tetrahydroaminoacridine; Tacrine hydrochloride; Tacrine HCl; CI-970; THA; tacrina; THA.HCl; Tacrin hydrochloride; Tacrinum; Talem; TACRINE; Tetrahydroaminacrine; OROS-tacrine; tacrine; Tacrin
Bide Pharmatech	BD289516	Oc1cccc(c1)c1ccc(s1)c1ccc(c(c1)C)O	C17H14O2S	282.36	4-(5-(3-Hydroxyphenyl)thiophen-2-yl)-2-methylphenol
Cayman Chemical	19186	CN(C[C@H](c1ccccc1)NC(=O)N1Cc2c(C1(C)C)[nH]nc2Nc1nc(C)nc2c1scc2)C	C25H30N8OS	490.62	PF-3758309
Cayman Chemical	19932	OC[C@H](c1cccc(c1)Cl)NC(=O)c1[nH]cc(c1)c1nc(ncc1C)Nc1ccc(cc1Cl)F	C24H20Cl2FN5O2	500.35	TCS ERK 11e
Medchem Express	HY-18072	O=C(N1CCc2c1ccc(c2)c1cn(c2c1c(N)ncn2)C)Cc1cccc(c1)C(F)(F)F	C24H20F3N5O	451.44	GSK2606414
AbovChem	HY-18630	C=CCN(CCCCCCOc1ccc(c(c1)F)C(=O)c1ccc(cc1)Br)C	C23H27BrFNO2	448.37	Ro 48-8071
Tocris	3544	O=C1C=C(OC(=C1)C3=C2SC4=C(SC2=CC=C3)C=CC=C4)N5CCOCC5	C21H17NO3S2	395.495	KU 55933; KU-55933; KU-0064
Tocris	3748	OC1=C2CSCCC2=NC(=N1)C3=CC=C(C=C3)C(F)(F)F	C14H11F3N2OS	312.31	XAV 939; XAV939; NVP-XAV-939; XAV-939

Tocris	4107	CCCN(CCC)[S](=O)(=O)C1=CC=C(C=C1)C(O)=O	C13H19NO4S	285.359	Probenecid; Probenecide; Probalan; Probampicin; Benemid Tab 500mg; Probenecida; Probenecid; 4-(N,N-Dipropylsulfamoyl)benzoesaeure; Pro Biosan Kit; Benemid; Probenecid acid; Benuryl; Proben; Probenecidum; probenecid; Probecid; 4-((Dipropylamino)sulfonyl)benzoic acid; C07372; Benecid; D00475; Probenecid and Colchicine; P-(Dipropylsulfamoyl)benzoic acid; 4-(Di-N-propylsulfamoyl)benzoesaeure; probenecidum; PROBENECID
Tocris	4361	COC1=C(Cl)C=C(Cl)C(=C1)NC2=C3C=C(OC)C(=CC3=NC=C2C#N)OCCCN4CCN(C)CC4	C26H29Cl2N5O3	530.446	Bosutinib; SKI-606; Bosulif; Bosulif; Bosutinib; D03252; BOSUTINIB; bosutinib.H2O; bosutinib hydrate; bosutinibum; SKI 606; bosutinib; 4-((2,4-Dichloro-5-methoxyphenyl)amino)-6-methoxy-7-(3-(4-methyl-1-piperazinyl)propoxy)-3-quinolinecarbonitrile; Bosutinib Monohydrate; PF-5208763; Bosutinib monohydrate
Apollo Scientific	BITP1545	OC(=O)C[C@@H]1NC(=O)CNC(=O)[C@H](CCCCNC(=N)N)NC(=O)CCSSC[C@H](NC(=O)[C@H]2N(C(=O)[C@@H](NC1=O)Cc1c[nH]c3c1cccc3)CCC2)C(=O)N	C35H49N11O9S2	831.96	Human Eptifibatide
AstaTech	F16272	O[C@H]1CCC2(C(=CC[C@H]3[C@@H]2CC2=C(C)[C@]4(CC[C@@H]32)O[C@@H]2[C@H]([C@H]4C)NC[C@H](C2)C)C1)C	C27H41NO2	411.62	CYCLOPAMINE
Cayman Chemical	10170	OB(CCSC[C@@H](C(=O)O)N)O	C5H12BNO4S	193.03	BEC
Axon Medchem	2003	COc1cccc(c1C1=NCc2c(c3c1cc(Cl)cc3)nc(nc2)Nc1ccc(c(c1)OC)C(=O)O)F	C27H20ClFN4O4	518.92	MLN 8237
Medchem Express	HY-16768	OC(=O)Cc1c(C)n(c2c1cccn2)Cc1ccc(cc1C(F)(F)F)S(=O)(=O)C	C19H17F3N2O4S	426.41	Fevipiprant
ChemShuttle	109785	N1CCN(CC1)c1ccc(cc1)c1cnc2n(c1)ncc2c1ccnc2c1cccc2	C25H22N6	406.48	4-(6-(4-(piperazin-1-yl)phenyl)pyrazolo[1,5-a]pyrimidin-3-yl)quinoline

Tocris	3545	NCCC1=C[NH]C=N1	C5H9N3	111.145	zirconium imido; Histamine; Histamine Dihydrochloride; Histamine diphosphate monohydrate; imido vanadium; Alo Therapeutic Massage Pain Relieving; Histamine dihydrochloride; titanium imido dichloride; histamine; histaminium; HISTAMINE HYDROCHLORIDE; HD-O; Arthritis Relief; niobium imido; rhodium imido; Imido; Histamine Phosphate; Histamine Phosphate Injection USP, 1mg/ml; Histamine phosphate; Histamine biphosphate; MX-8899; Histamine diphosphate; Bichlorhydrate d'histamine; Histamine Positive skin test control; HISTAMINE DIHYDROGEN PHOSPHATE; Imido titanium; HISTAMINE; b-Aminoethylglyoxaline; titanium imido; histamine amine; Dr. Freds Miracle Rub; IMIDO DIPHOSPHATE; Histatrol; Histamine Phosphate Injection USP Liq Sc; Histamium; HISTAMINE
Tocris	3793	OC(=O)C1=C(NC(=O)N2CCC(CC2)C3=C(C=CC=C3)C(F)(F)F)C=CC=C1	C20H19F3N2O3	392.372	A 1120; A-1120; 2-[({4-[2-(trifluoromethyl)phenyl]piperidin-1-yl}carbonyl)amino]benzoic acid
Tocris	4152	4	C21H14ClFN2O2	380.799	TMN 355
Tocris	4370	C1=CN=C(C=C1)NC4=NC2=C(CCCC3=N[NH]C=C23)S4	C14H13N5S	283.352	TC-N 22A
Oxchem	AX8288312	OC(=O)c1cccc(c1)Nc1ccc(cc1)C(F)(F)F	C14H10F3NO2	281.23	3-((4-(Trifluoromethyl)phenyl)amino)benzoic acid
Axon Medchem	1130	O=C(N1CCCC1)Nc1cccc(c1)Nc1ncc(c(n1)NCCc1c[nH]cn1)Br	C20H23BrN8O	471.35	BX 912
Cayman Chemical	18095	CNc1nc(cc(n1)c1ccc2c(c1)[nH]nc2N)N1C[C@H](CC[C@H]1C)C(=O)NC1CCCCC1	C25H34N8O	462.59	GSK2334470
Bide Pharmatech	BD237663	COc1cccc(c1c1ccc(cc1)C[C@@H](C(=O)O)NC(=O)c1c(Cl)cccc1Cl)OC	C24H21Cl2NO5	474.33	TR-14035
Medchem Express	HY-11004	N#CC(c1cccc(c1)C(=O)Nc1ccc(c(c1)Nc1ccc2c(c1)c(=O)n(cn2)C)C)(C)C	C27H25N5O2	451.52	AZ 628
ChemShuttle	105147	O=C(Nc1noc(c1)C(C)(C)C)Nc1ccc(cc1)c1cn2c(n1)sc1c2ccc(c1)OCCN1CCOCC1	C29H32N6O4S	560.67	1-(5-(tert-butyl)isoxazol-3-yl)-3-(4-(7-(2-morpholinoethoxy)benzo[d]imidazo[2,1-b]thiazol-2-yl)phenyl)urea

Tocris	3548	NCCC1=CC(=C(O)C=C1)O	C8H11NO2	153.178	[illegible] Dopamine Hydrochloride; dopaminium cation; Hydroxytyramine; Dopamine hydrochloride; Intropin; DA; Dopamina; C03758; Dopamine HCl; 4-(2-Aminoethyl)pyrocatechol; Dynatra; dopamine; Revimine; Dopamine Hcl Inj 4%; DOPAMINE HYDROCHLORIDE; 2-(3,4-Dihydroxyphenyl)ethylamine; 4-(2-Aminoethyl)catechol; Tensamin; Dopaminum; Dopamine; Hydroxytyramin; Intropin Injection 40mg/ml; Dopamine Hydrochloride and Dextrose; DOPAMINE; dopaminium(1+); dopaminum; Rascordin; Sabax Dopamin; Dopamin-Natterman; Deoxyepinephrine; Dopamine methiodide; Oxytyramine; 3-Hydroxytyramine; Dophamine; Dopamine Hcl; 3,4-Dihydroxyphenethylamine; 4-(2-Aminoethyl)-1,2-benzenediol; 4-(2-Aminoethyl)benzene-1,2-diol; dopamina; Dopamin
Tocris	3826	OC1=C(C2=C(C=C1)C(=O)C=C(O2)C3=CC=CC=C3)O	C15H10O4	254.238	7,8-Dihydroxyflavone; 7,8-DIHYDROXYFLAVONE; 7,8-Dihydroxyflavone
Tocris	4171	FC1=CC(=CC=C1)N2CNC(=O)C23CCN(CCNC(=O)C4=CC5=CC=CC=C5C=C4)CC3	C26H27FN4O2	446.517	VU 0364739 hydrochloride; VU-0364739
Tocris	4427	CC(C)COC1=CC=C(C=C1C#N)C2=NC(=C(S2)C(O)=O)C	C16H16N2O3S	316.375	Febuxostat; febuxostat; Febuxostat potassium; Adenuric; Febric; Febuxostat; Feburic; Uloric; TEI-6720; Febuxostat lysine salt; Febuxostat triethanolamine salt; Febuxostat sodium; FEBUXOSTAT; TMX-67; Febuxostat choline salt; Febuxostat tromethamine; febuxostatum; Febuxostat triethylamine salt
Key Organics/BIONET - BioChemicals Stock	SS-4313	CCCCCCCCCCCCCCOc1ccc(o1)C(=O)O	C19H32O4	324.45	TOFA, RMI 14514

Cayman Chemical	15403	CCOC(=O)NCCCC(=O)NCCc1ccccc1	C15H22N2O3	278.35	CAY10683
Cayman Chemical	10008933	CC(=O)c1ccc2c(c1)[C@@H]1C=CC[C@@H]1[C@@H](N2)c1cc2OCOc2cc1Br	C21H18BrNO3	412.28	G-1
AbovChem	HY-15452A	NC(=O)[C@H]1CCCc2c1[nH]c1c2cc(cc1)Cl	C13H13ClN2O	248.71	Selisistat S-enantiomer(EX-527 S-enantiomer)
Medchem Express	HY-18305	O=C(C(C)C)Nc1ncc(s1)c1cc(nn1c1c(Cl)cccc1Cl)C(F)F	C17H14Cl2F2N4OS	431.29	BMS-5
ChemShuttle	134398	Cn1cc(nc1C1CCN(CC1)c1ncnc2c1cn[nH]2)c1ccc(c(c1)C(F)(F)F)F	C21H19F4N7	445.42	4-[4-[4-[4-fluoro-3-(trifluoromethyl)phenyl]-1-methylimidazol-2-yl]piperidin-1-yl]-1H-pyrazolo[3,4-d]pyrimidine
Tocris	3572	OC(=O)C1=C(C=C(C=C1)C2=C[NH]C3=NC=C(C=C23)C4=CC=CC=C4)C5CCCC5	C25H22N2O2	382.454	GSK 650394; GSK-650394
Tocris	3896	CCCN(CCC1=CC=CS1)C2CCC3=C(C2)C=CC=C3O	C19H25NOS	315.473	Rotigotine hydrochloride; Rotigotine; Neupro; N-0437; D05768
Tocris	4227	CC1=C(C=N[N]1C2=NC(=CS2)C3=CC=CC(=C3)C(F)(F)F)C(=O)NCCCO	C18H17F3N4O2S	410.413	RWJ 50271; RWJ-50271
Tocris	4479	CC1=CC(=O)OC2=C1C=CC(=C2C=O)O	C11H8O4	204.179	4μ 8C
Shanghai AQBioPharma Co. Ltd. - Stock	ABF12752	O=C(c1ccccc1c1ccc(cc1)C(F)(F)F)NC1CCN(CC1)CCCCC1(C(=O)NCC(F)(F)F)c2ccccc2c2c1cccc2	C39H37F6N3O2	693.72	N-(2,2,2-trifluoroethyl)-9-(4-(4-(4'-(trifluoromethyl)biphenyl-2-ylcarboxamido)piperidin-1-yl)butyl)-9H-fluorene-9-carboxamide
Cayman Chemical	10007927	OC(=O)CCCCCCCCCCCNC(=O)NC12CC3CC(C2)CC(C1)C3	C23H40N2O3	392.58	AUDA
Cayman Chemical	17408	[O-][N+](=O)c1ccc(c2c1non2)Nc1ccccc1c1ccccc1	C18H12N4O3	332.31	10074-G5
AbovChem	HY-10442	Nc1nccc2c1ncn2[C@@H]1C=C([C@H]([C@H]1O)O)CO	C12H14N4O3	262.26	3-Deazaneplanocin A
Medchem Express	HY-13259	O=C[C@@H](NC(=O)[C@@H](NC(=O)[C@@H](NC(=O)OCc1ccccc1)CC(C)C)CC(C)C)CC(C)C	C26H41N3O5	475.62	MG-132
Selleck Chemicals	S7252	O=C(/C=C\n1cnc(n1)c1cc(cc(c1)C(F)(F)F)C(F)(F)F)NNc1cnccn1	C17H11F6N7O	443.31	KPT-330

Tocris	3609	ClC1=C(NC2=NCCN2)C3=NSN=C3C=C1	C9H8ClN5S	253.711	Tizanidine hydrochloride; Cimbrar; Sizolan; Sirdalid; Tizanidine; Ternelin; Telzanine; Tizan; DS-103282; Sirdalud; Tizanidine hydrochloride; TIZANIDINE; Tizanidine succinate; Tizalud; Musant; Tizanidina; Tizaflex; DS-103282 ch; Zanaflex; ZANAFLEX; 5-Chloro-4-(2-imidazolin-4-on-2-ylamino)-2,1,3-benzothiazdiazole; 5-Chloro-4-(2-imidazolin-2-ylamino)-2,1,3-benzothiadiazole; Zanpeak; Tizadin; Pal-tizanidine; Tizanidin; tizanidine; Sirdalud MR; Tizanidinum; Navizan; Mylan-tizanidine; Tizanidine HCl; TIZANIDINE HYDROCHLORIDE; Tizanidine Hydrochloride; Spaslax; Relaxkov; NVD-422; Myores; AN-021A; Tizanidine sulfate; Zitanid; C07452
Tocris	3912	CN1C2=CC=C(C=C2N=C(C3=CC=C(C=C3)C(O)=O)C4=CC5=C(C=C14)C(C)(C)CCC5(C)C)[N+]([O-])=O	C29H29N3O4	483.558	HX 531; HX-531
Tocris	4255	COC1=CC(=CC(=C1)OC)C(=O)NNC(=O)NC2=CC=CC3=C2C=CC=C3	C20H19N3O4	365.383	TC-O 9311
Tocris	4493	CC5=C(CCN1CCC(CC1)C2=NOC3=CC(=CC=C23)F)C(=O)N4CCCC(O)C4=N5	C23H27FN4O3	426.484	Paliperidone; PALIPERIDONE PALMITATE; R-76477; Ro-76477; PALIPERIDONE; 9-Hydroxyrisperidone; Paliperidone; Invega; D05339; SID90341688; 3-{2-[4-(6-fluoro-1,2-benzoxazol-3-yl)piperidin-1-yl]ethyl}-9-hydroxy-2-methyl-6,7,8,9-tetrahydropyrido[1,2-a]pyrimidin-4-one; paliperidona; Invega Sustenna; paliperidonum; Paliperidone Palmitate; JNS-007ER; RO-76477
Pharmablock - US Stock	PBLJ6619	OC[C@H]1N[C@H]([C@@H]([C@@H]1O)O)c1c[nH]c2c1nc[nH]c2=O	C11H14N4O4	266.25	Forodesine
Cayman Chemical	16162	Fc1cc(F)cc(c1)[C@@H](C(=O)N[C@H](C(=O)N[C@H]1c2ccccc2c2c(N(C1=O)C)cccc2)C)O	C26H23F2N3O4	479.48	LY411575
ChemPacific	33433	COC(=O)CCc1ccc(cc1)OCC(CNC(C)C)O	C16H25NO4	295.37	Esmolol
Medchem Express	HY-10446	C#CCC(c1ccc(cc1)C(=O)N[C@H](C(=O)O)CCC(=O)O)Cc1cnc2c(n1)c(N)nc(n2)N	C23H23N7O5	477.47	Pralatrexate
Medchem Express	HY-15667	[O-][N+](=O)c1cc(sc1Sc1cccc(c1Cl)Cl)C(=O)C	C12H7Cl2NO3S2	348.22	P005091
Selleck Chemicals	S1290	O=C1C=C2C(=CC=C3[C@@]2(C)CC[C@@]2([C@]3(C)CC[C@@]3([C@H]2C[C@@](C)(CC3)C(=O)O)C)C)C(=C1O)C	C29H38O4	450.61	Celastrol

Tocris	3618	CC(=O)NCCC[S](=O)(=O)O	C5H11NO4S	181.21	Acamprosate calcium; ACAMPROSATE; Campral; Acamprosate calcium; Regtect; Acamprosate Calcium; Zulex; N-Acetylhomotaurine; Sobriol; Acamprosate; CaAOTA; Acamprosato; acamprosate calcium; ACAMPROSATE CALCIUM; N-acetyl homotaurine; calcium acetylhomotaurinate; Acamprosat; Calcium Bisacetyl Homotaurine; Aotal; Acamprosatum; Alcomed; D02780; CA-Aota; 3-Acetamido-1-propanesulfonic acid; calcium acetyl homotaurinate; NS-11; SNC-102; acamprosate(1-); acamprosate; acamprosatum
Tocris	3918	CCC1=C(C(=NC(=N1)N)N)C2=CC=C(Cl)C=C2	C12H13ClN4	248.711	Pyrimethamine; Chloridine; PYRIMETHAMINE; Primethamine; Pyrimethaminum; pirimecidan; pyrimethamine; EXR-101; Pyrimethamine; Daraprim; Diaminopyritamin; Pirimetamina; 2,4-Diamino-5-chlorophenyl-6-ethylpyrimidine; 2,4-Diamino-5-(4-chlorophenyl)-6-ethylpyrimidine; CD; chloridine; Malacide; Pyrimethamin; Fansidar Tablets; 5-(4-Chlorophenyl)-6-ethyl-2,4-pyrimidinediamine; Chloridyn; Ethylpyrimidine; 5-(4-Chlorophenyl)-6-ethyl-2,4-diaminopyrimidine; 5-(4'-Chlorophenyl)-2,4-diamino-6-ethylpyrimidine; pirimetamin; Chloridin; 2,4-Diamino-5-(P-chlorophenyl)-6-ethylpyrimidine; D00488; darachlor; Malocide; TRP-004; C07391; Pyremethamine; Pyrimethamine Hcl
Tocris	4343	COC1=CC2=C(NC3CCN(CC3)C(C)C)N=C(N=C2C=C1OCCCN4CCCC4)C5CCCC5	C30H47N5O2	509.726	UNC 0638; UNC-0638

Tocris	4620	CCCC1=NC(=C([N]1CC2=CC=C(C=C2)C3=C(C=CC=C3)C4=NN=N[NH]4)C(=O)OCC5=C(C)OC(=O)O5)C(C)(C)O	C29H30N6O6	558.585	Olmesartan medoxomil; Olvance; Olmetec; CS-866RN; OLMESARTAN MEDOXOMIL; Benevas; Openvas; Olmesartan medoxomil; Benicar; Olmesartan Medoxomil; olmesartanum; olmesartanum medoxomilum; CS-866DM
Pharmablock - US Stock	PBLJ6434	COC(=O)N[C@H](CNc1nccc(n1)c1cn(nc1c1cc(Cl)cc(c1F)NS(=O)(=O)C)C(C)C)C	C22H27ClFN7O4S	540.01	encorafenib
Cayman Chemical	11975	CCCCCCCCc1ccc(cc1)CCC(CO)(CO)N	C19H33NO2	307.47	Fingolimod
Cayman Chemical	16605	CCCCC[C@@H](C(=O)N[C@H](C(=O)N1CCC[C@H]1CO)C(C)C)CC(=O)NO	C19H35N3O5	385.5	Actinonin
Medchem Express	HY-15306	OC(=O)c1cccc(c1)c1cccc(c1O)N/N=C\1/C(=NN(C1=O)c1ccc(c(c1)C)C)C	C25H22N4O4	442.47	Eltrombopag
Medchem Express	HY-13788	CN([C@@H]1CCc2c(C1)c1cc(ccc1[nH]2)NC(=O)c1ccc(cc1)F)C	C21H22FN3O	351.42	LY 344864
Selleck Chemicals	S2224	ONC(=O)C[C@H](c1onc(n1)C(=O)N)CCCC1CCCCC1	C15H24N4O4	324.38	UK 383367
Selleck Chemicals	S2801	COc1cc(CCc2[nH]nc(c2)NC(=O)c2ccc(cc2)N2C[C@H](C)N[C@@H](C2)C)cc(c1)OC	C26H33N5O3	463.57	AZD4547
Target Molecule	T1875	CN(C(=O)Nc1cccc(c1)c1ccc(cc1)N(C)C)C	C17H21N3O	283.37	Atglistatin
Target Molecule	T2457	O=C(c1cccc(c1)[N+](=O)[O-])Nc1nc2c(n1CCN1CCOCC1)cccc2	C20H21N5O4	395.41	IRAK-1-4 Inhibitor I
Key Organics/BIONET - BioChemicals Stock	KS-1315	Clc1ccc2c(c1)C(O)CCCN2C(=O)c1ccc(cc1C)NC(=O)c1ccccc1C	C26H25ClN2O3	448.94	Tolvaptan
Combi-Blocks	QC-0834	OC[C@H]1O[C@H]([C@@H]([C@@H]1O)O)n1cccnc1=O	C9H12N2O5	228.2	Zebularine
Bide Pharmatech	BD17153	O=C1CCc2c(N1)cc(cc2)OCCCCN1CCN(CC1)c1cccc(c1Cl)Cl	C23H27Cl2N3O2	448.39	Aripiprazole
Key Organics/BIONET - BioChemicals Stock	KS-1398	O=C(Cc1csc(n1)N)Nc1ccc(cc1)CCNC[C@@H](c1ccccc1)O	C21H24N4O2S	396.51	Mirabegron
Cayman Chemical	16131	CO[C@H]1/C=C/O[C@@]2(C)Oc3c(C2=O)c2c(c(c3C)O)c(O)c(c3c2nc2n3ccc(c2)C)NC(=O)/C(=C\C=C\[C@@H]([C@@H]([C@H]([C@H]([C@H]([C@@H]([C@@H]1C)OC(=O)C)C)O)C)O)C)/C	C43H51N3O11	785.88	Rifaximin
Pharmablock - US Stock	PBN2011293	CS(=O)(=O)N1CCN(CC1)Cc1cc2c(s1)c(nc(n2)c1cccc2c1cn[nH]2)N1CCOCC1	C23H27N7O3S2	513.64	Pictilisib
Enamine BB - US Stock	EN300-119493	CC(=O)Nc1nnc(s1)S(=O)(=O)N	C4H6N4O3S2	222.25	N-(5-sulfamoyl-1,3,4-thiadiazol-2-yl)acetamide
Target Molecule	T1279	N#Cc1ccc2c(c1)c(CCCCN1CCN(CC1)c1ccc3c(c1)cc(o3)C(=O)N)c[nH]2	C26H27N5O2	441.52	Vilazodone
Target Molecule	T2379	O=C(c1ccc(c(c1)Nc1ccccc1)C(=O)N1CCC(CC1)N1CCCC1)N1CCC(CC1)N1CCCC1	C32H43N5O2	529.72	UNC1215
Target Molecule	T1853	Cc1cc(OCc2nnc(n2c2cccnc2)SC2CCCC2)ccc1c1ccc(cc1)S(=O)(=O)C	C27H28N4O3S2	520.67	NMS-873
Bide Pharmatech	BD224801	CC[C@@H](c1nc2cccc(c2c(=O)n1c1ccccc1)F)Nc1ncnc2c1nc[nH]2	C22H18FN7O	415.42	CAL-101
Combi-Blocks	ST-7591	OCCN1CCN(CC1)c1cc(nc(n1)C)Nc1ncc(s1)C(=O)Nc1c(C)cccc1Cl	C22H26ClN7O2S	488.01	Dasatinib

AbovChem	HY-N0127	COC(=O)[C@H]1[C@@H](O)CC[C@@H]2[C@@H]1C[C@@H]1N(C2)CCc2c1[nH]c1c2cccc1	C21H26N2O3	354.44	Yohimbine
Pharmablock - US Stock	PBLJ6152	N#CCNC(=O)c1ccc(cc1)c1ccnc(n1)Nc1ccc(cc1)N1CCOCC1	C23H22N6O2	414.46	benzamide, n-(cyanomethyl)-4-[2-[[4-(4-morpholinyl)phenyl]amino]-4-pyrimidinyl]-
Combi-Blocks	QE-9393	Oc1ccc2c(c1)Oc1c(C32OC(=O)c2c3cccc2)ccc(c1)O	C20H12O5	332.31	Fluorescein
Advanced ChemBlocks - In Stock	G-7963	CNC(=O)c1ccc(cc1F)N1C(=S)N(C(=O)C1(C)C)c1ccc(c(c1)C(F)(F)F)C#N	C21H16F4N4O2S	464.44	
Target Molecule	T1803	OCCNC(=O)c1cccc(c1)c1ncnc(c1)Nc1ccc(cc1)OC(F)(F)F	C20H17F3N4O3	418.37	GNF-5
Target Molecule	T2504	O=C(N(c1nc(c(s1)S(=O)(=O)N)C)C)Cc1ccc(cc1)c1ccccn1	C18H18N4O3S2	402.49	BAY 57-1293
Target Molecule	T3067	O=c1sn(c(=O)n1Cc1ccccc1)c1cccc2c1cccc2	C19H14N2O2S	334.39	Tideglusib
Target Molecule	T1947	NCCCCCCNc1cc(NCc2ccccc2)n2c(n1)c(cn2)C(C)C	C22H32N6	380.53	BS-181
AstaTech	41167	CN(Cc1cccc2c1cccc2)C/C=C/C#CC(C)(C)C	C21H25N	291.43	TERBINAFINE HCL
Bide Pharmatech	BD145505	CCOc1cc(ccc1C(=O)O)CC(=O)N[C@H](c1ccccc1N1CCCCC1)CC(C)C	C27H36N2O4	452.59	Repaglinide
Pharmablock - US Stock	PBN2011567	CCN(CCN(C(=O)Cn1c(SCc2ccc(cc2)F)nc(=O)c2c1CCC2)Cc1ccc(cc1)c1ccc(cc1)C(F)(F)F)CC	C36H38F4N4O2S	666.77	Darapladib
Cayman Chemical	15785	ONC(=O)c1ccc(cc1)Cn1c2ccccc2c2c1CCN(C2)C	C20H21N3O2	335.4	Tubastatin A
Vitas M Labs - BB	BBL023172	COc1ccc(cc1)C(=O)C(=O)c1ccccc1	C15H12O3	240.25	BBL023172
Selleck Chemicals	S7500	Cc1cc(C)c(c(c1)C)S(=O)(=O)n1cc(cc1C)C	C15H19NO2S	277.38	HJC0350
Target Molecule	T0162	OCCOCCN1CCN(CC1)C1=Nc2ccccc2Sc2c1cccc2	C21H25N3O2S	383.51	Quetiapine
Target Molecule	T6278	CN1CCN(CC1)c1nc2ccccc2c(n1)C1=C(C(=O)NC1=O)c1c[nH]c2c1cccc2	C25H22N6O2	438.48	Sotrastaurin
Target Molecule	T3032	O=C(c1nc(cnc1N)c1ccc(cc1)S(=O)(=O)C)Nc1ccccc1	C18H16N4O3S	368.41	VE-821
Bepharm	B17734	NC[C@@H]1CC[C@H](CC1)C(=O)O	C8H15NO2	157.21	Tranexamic acid
Advanced ChemBlocks - In Stock	M15325	Cc1cc(C(Nc2ccccc2)C)c2n(c1)c(=O)cc(n2)N1CCOCC1	C21H24N4O2	364.44	663619-89-4
Bide Pharmatech	BD113240	N#Cc1ccccc1Cn1c(cc(=O)n(c1=O)C)N1CCC[C@H](C1)N	C18H21N5O2	339.39	Alogliptin
Pharmablock - US Stock	THR0011	CC(=O)c1c(C)c2cnc(nc2n(c1=O)C1CCCC1)Nc1ccc(cn1)N1CCNCC1	C24H29N7O2	447.53	palbociclib
Cayman Chemical	19768	CCc1oc2c(c1C(=O)c1cc(Br)c(c(c1)Br)O)cccc2	C17H12Br2O3	424.08	Benzbromarone
Advanced ChemBlocks - In Stock	M15125	Cc1cnc(nc1Nc1cccc(c1)S(=O)(=O)NC(C)(C)C)Nc1ccc(cc1)OCCN1CCCC1	C27H36N6O3S	524.68	
Combi-Blocks	QA-4191	CC(NC(=O)NS(=O)(=O)c1cnccc1Nc1cccc(c1)C)C	C16H20N4O3S	348.42	Torasemide
Target Molecule	T1611	CC(=CC=CC(=CC(=O)O)C)C=CC1=C(C)CCCC1(C)C	C20H28O2	300.44	Isotretinoin
Target Molecule	T2627	Cc1ccc2c(c1Sc1ccncc1)c(=O)nc([nH]2)N	C14H12N4OS	357.26	AG-337; Nolatrexed 2hydrochloride
Target Molecule	T1943	COc1ccc(cc1)c1cnc2n(c1)ncc2c1cccc2c1cccn2	C22H16N4O	352.39	mL347
Bide Pharmatech	BD3002	N=C(N[N+](=O)[O-])NCCC[C@@H](C(=O)O)N	C6H13N5O4	219.2	H-Arg(NO2)-OH

Combi-Blocks	QA-9111	CSc1nc2c([nH]1)cc(c(c2)Cl)Oc1cccc(c1Cl)Cl	C14H9Cl3N2OS	359.66	Triclabendazole
Medchem Express	HY-13501	FC(c1ccc(cc1)/C=C/c1occ(n1)COc1ccc(cc1)CCCCn1nncc1)(F)F	C25H23F3N4O2	468.47	Mubritinib
Pharmablock - US Stock	PBLJ6189	C=CCn1c(=O)c2c(n1c1cccc(n1)C(O)(C)C)nc(nc2)Nc1ccc(cc1)N1CCN(CC1)C	C27H32N8O2	500.6	MK-1775
Cayman Chemical	20390	O=C(CNC(=O)C(Cc1ccccc1)CSC(=O)C)OCc1ccccc1	C21H23NO4S	385.48	Racecadotril
Advanced ChemBlocks - In Stock	I-9209	CCc1cc2C(=O)c3c4ccc(cc4[nH]c3C(c2cc1N1CCC(CC1)N1CCOCC1)(C)C)C#N	C30H34N4O2	482.62	
Cayman Chemical	15987	CC1Nc2cc(Cl)c(cc2C(=O)N1c1ccccc1C)S(=O)(=O)N	C16H16ClN3O3S	365.83	Metolazone
Target Molecule	T1330	COc1cc(C)c(c(c1C)C)C=CC(=CC=CC(=CC(=O)O)C)C	C21H26O3	326.43	Acitretin
Target Molecule	T2670	OCc1ccc(o1)c1ccc2c(c1)c(ncn2)NCc1ccc(o1)C	C19H17N3O3	335.36	mL167
Target Molecule	T2588	O=C(c1c[nH]c2c(c1=O)cccc2)Nc1cc(O)c(cc1C(C)(C)C)C(C)(C)C	C24H28N2O3	392.49	Ivacaftor
Bide Pharmatech	BD235626	O=C1CCC(C(=O)N1)N1C(=O)c2c(C1=O)c(N)ccc2	C13H11N3O4	273.24	Pomalidomide
Combi-Blocks	QA-6056	Cc1cnc(s1)NC(=O)C1=C(O)c2ccccc2S(=O)(=O)N1C	C14H13N3O4S2	351.4	Meloxicam
Medchem Express	HY-50898	Fc1cccc(c1)COc1ccc(cc1Cl)Nc1ncnc2c1cc(cc2)c1ccc(o1)CNCCS(=O)(=O)C	C29H26ClFN4O4S	581.06	Lapatinib
J&K Scientific	985371	OC[C@H]1O[C@H](O)[C@@H]([C@H]([C@@H]1O)O)O	C6H12O6	180.16	D-(+)-Glucose, ACS reagent
Cayman Chemical	14337	OC(=O)[C@H](Cn1ccc(=O)c(c1)O)N	C8H10N2O4	198.18	L-Mimosine
Advanced ChemBlocks - In Stock	10258	O=C(Nc1cc(ccc1c1ccsc1)S(=O)(=O)NC1CC1)Nc1cccc(c1)C(F)(F)F	C21H18F3N3O3S2	481.51	
Combi-Blocks	QE-1174	C[C@@H]1CC[C@H]([C@@H](C1)O)C(C)C	C10H20O	156.27	L-Menthol
Enzo Life Sciences	ALX-430-146	C=CCc1cccc(c1O)/C=N/NC(=O)CN1CCN(CC1)Cc1ccccc1	C23H28N4O2	392.49	PAC-1
Target Molecule	T6053	CC(OC(=O)C1=CN(CC(c2c1[nH]c1c2cccc1)(C)C)C(=O)c1ccc(c(c1)F)F)C	C25H24F2N2O3	438.47	Turofexorate Isopropyl (XL335)
Target Molecule	T1857	O=C(c1cccc(c1)c1noc(n1)C(F)(F)F)NCC1(CCOCC1)c1scc(n1)c1ccccc1	C25H21F3N4O3S	514.52	N-((4-(4-phenylthiazol-2-yl)tetrahydro-2H-pyran-4-yl)methyl)-3-(5-(trifluoromethyl)-1,2,4-oxadiazol-3-yl)benzamide
Chem-Impex	126	CC[C@H]1OC(=O)[C@H](C)[C@@H](O[C@@H]2O[C@@H](C)[C@@H]([C@](C2)(C)OC)O)[C@H](C)[C@@H](O[C@@H]2O[C@H](C)C[C@@H]([C@H]2O)N(C)C)[C@](C[C@H](C(=O)[C@@H]([C@H]([C@]1(C)O)O)C)C)(C)O	C37H67NO13	733.93	Erythromycin
Bide Pharmatech	BD229143	OCCOc1c(C)cc(cc1C)c1nc2cc(OC)cc(c2c(=O)[nH]1)OC	C20H22N2O5	370.4	RVX-208
Medchem Express	HY-17386	O=C1NC(=O)C(S1)Cc1ccc(cc1)OCCN(c1ccccn1)C	C18H19N3O3S	357.43	Rosiglitazone
Combi-Blocks	AN-2948	Nc1ccc(cc1)S(=O)(=O)N	C6H8N2O2S	172.2	Sulfanilamide
Cayman Chemical	16128	N#CC(=NNc1ccc(cc1)C1=NNC(=O)C[C@H]1C)C#N	C14H12N6O	280.28	Levosimendan
Advanced ChemBlocks - In Stock	R16056	O=C(Nc1ccc(cc1N)F)/C=C/c1cnn(c1)C/C=C/c1ccccc1	C21H19FN4O	362.4	
Enamine BB - US Stock	EN300-123537	O=c1oc2ccccc2c(c1Cc1c(=O)oc2c(c1O)cccc2)O	C19H12O6	336.29	107753-78-6

Key Organics/BIONET - BioChemicals Stock	SS-4787	OCc1cc(ccc1OC)c1ccc2c(n1)nc(nc2N1CCOC[C@@H]1C)N1CCOC[C@@H]1C	C25H31N5O4	465.54	AZD8055
Target Molecule	T2044	CCc1c(C(=O)C(=O)N)c2c(n1Cc1ccccc1)cccc2OCC(=O)O	C21H20N2O5	380.39	Varespladib
Target Molecule	T0389	CCCCCCOC(=O)N=C(c1ccc(cc1)NCc1nc2c(n1C)ccc(c2)C(=O)N(c1ccccn1)CCC(=O)OCC)N	C34H41N7O5	627.73	Dabigatran etexilate
Combi-Blocks	QA-4624	COc1c(C/C=C(/CCC(=O)O)\C)c(O)c2c(c1C)COC2=O	C17H20O6	320.34	Mycophenolic acid
Bide Pharmatech	BD218703	OC(=O)COc1ccc(cc1C)SCc1sc(nc1C)c1ccc(cc1)C(F)(F)F	C21H18F3NO3S2	453.5	GW 501516
Bide Pharmatech	BD55672	OC(=O)C(c1ccc2c(c1)CC(=O)c1c(S2)cccc1)C	C17H14O3S	298.36	Zaltoprofen
Combi-Blocks	OR-1175	O=C(NS(=O)(=O)c1ccc(cc1)C)NN1CC2C(C1)CCC2	C15H21N3O3S	323.41	Gliclazide
Combi-Blocks	QA-2612	Clc1ccc(cc1)OC(C(=O)C(C)(C)C)n1cncc1	C15H17ClN2O2	292.76	Climbazole
Pharmablock - US Stock	PBLJ6191	O=C(OCc1cccnc1)NCc1ccc(cc1)C(=O)Nc1ccccc1N	C21H20N4O3	376.41	Entinostat
Enamine BB - US Stock	EN300-119517	COC(=O)C1=C(C)NC(=C(C1c1ccccc1[N+](=O)[O-])C(=O)OCC(C)C)C	C20H24N2O6	388.41	3-methyl 5-(2-methylpropyl) 2,6-dimethyl-4-(2-nitrophenyl)-1,4-dihydropyridine-3,5-dicarboxylate
Key Organics/BIONET - BioChemicals Stock	ES-0052	COc1cc2c(ncnc2cc1OCCCN1CCCC1)Oc1ccc2c(c1F)cc([nH]2)C	C25H27FN4O3	450.51	AZD2171, Cediranib
Tocris	2385	Cl.Cl.C(CNC(C1=CC=CC=C1)C1=CC=CC=C1)NC(C1=CC=CC=C1)C1=CC=CC=C1	C28H30Cl2N2	465.45	AMN 082 dihydrochloride
Medchem Express	HY-A0063A	O=c1[nH]c(CN2CCCC2=N)c(c(=O)[nH]1)Cl	C9H11ClN4O2	242.66	Tipiracil
Medchem Express	HY-13946	Clc1ccc(cc1)Oc1ccc(cc1)c1nc2c([nH]1)ccc(c2)C(=O)N	C20H14ClN3O2	363.8	BML-277
Selleck Chemicals	S1043	COc1cc2c(cc1OCCCN1CCCCC1)ncnc2N1CCN(CC1)C(=O)Nc1ccc(cc1)OC(C)C	C31H42N6O4	562.7	Tandutinib (MLN518)
Maybridge	GK01981	COC(=O)c1sccc1NC(=O)Cc1cccs1	C12H11NO3S2	281.35	GK01981
Vitas M Labs	STK832240	Oc1ccc(cc1)/C=N/N(Cc1ccccc1)Cc1ccccc1	C21H20N2O	316.4	STK832240
Vitas M Labs	STL321288	C(NC(c1ccccc1)c1ccccc1)CNC(c1ccccc1)c1ccccc1	C28H28N2	392.54	STL321288
Vitas M Labs	STK772817	OCCCCn1c(cn2c1nc1c2c(=O)[nH]c(=O)n1C)c1ccccc1	C18H19N5O3	353.38	STK772817
Maybridge	KM03430	CSc1sc(c2c1C(=O)CC(C2)(C)C)c1ccn(n1)C(=O)C	C16H18N2O2S2	334.46	KM03430
Enamine BB - UA Backorder	EN300-122638	O=C1CN(C(=O)N1)/N=C/c1ccc(o1)c1ccc(cc1)[N+](=O)[O-]	C14H10N4O5	314.25	1-({[5-(4-nitrophenyl)furan-2-yl]methylidene}amino)imidazolidine-2,4-dione
Key Organics/BIONET - Screening and Fragments - Stock	MS-0333	CCCOc1ccc(cc1)/C=C/1\SC(=O)NC1=O	C13H13NO3S	263.31	5-[(Z)-(4-propoxyphenyl)methylidene]-1,3-thiazolane-2,4-dione
Target Molecule	T2456	COc1cc2c(ccnc2cc1OC)Oc1ccc(c(c1)Cl)NC(=O)Nc1noc(c1)C	C22H19ClN4O5	454.86	Tivozanib
AbovChem	HY-15370A	CC1CCN(CC1)CC[C@H]1CCCN1S(=O)(=O)c1cccc(c1)O	C18H28N2O3S	352.49	SB-269970

Enamine Screening Compounds	Z274554748	O=C(N1CCOCC1)Oc1nsnc1N1CCCCC1	C12H18N4O3S	298.36	Z274554748
Maybridge	SPB06189	NC(=N)N/N=C/c1ccc(c(c1)Cl)Cl	C8H8Cl2N4	231.08	SPB06189
Vitas M Labs	STK869906	Fc1ccccc1c1oc(=O)c2c(n1)sc1c2CCCC1	C16H12FNO2S	301.34	STK869906
Vitas M Labs	STK235555	O=C(N1CCCc2c1cccc2)CSc1nc2c(s1)cccc2	C18H16N2OS2	340.46	STK235555
Vitas M Labs	STK746869	N#Cc1c(SCC(=O)Nc2nnc(s2)CC)nc(nc1SC)N	C12H13N7OS3	367.47	STK746869
ChemDiv	D399-0002	Clc1ccc(cc1)S(=O)(=O)N1CCCC1c1cccs1	C14H14ClNO2S2	327.85	D399-0002
Advanced ChemBlocks - In Stock	R16001	Cc1[nH]c2c(n1)CCN(c1c2cccc1)C(=O)c1ccc(cc1)NC(=O)c1ccccc1c1ccccc1	C32H26N4O2	498.57	conivaptan
Key Organics/BIONET - Screening and Fragments - Stock	12P-030	OC(=O)C(CC(=O)c1ccc(cc1)c1ccc(cc1)Cl)CSc1ccccc1	C23H19ClO3S	410.91	4-[4-(4-chlorophenyl)phenyl]-4-oxo-2-[(phenylsulfanyl)methyl]butanoic acid
AbovChem	HY-15979A	Brc1ccc(cc1)/C=C/CNCCNS(=O)(=O)c1cccc2c1ccnc2	C20H20BrN3O2S	446.36	H 89
Bide Pharmatech	BD262844	Clc1ccc(cc1)C1=C(CCC(C1)(C)C)CN1CCN(CC1)c1ccc(c(c1)Oc1cnc2c(c1)cc[nH]2)C(=O)NS(=O)(=O)c1ccc(c(c1)[N+](=O)[O-])NCC1CCOCC1	C45H50ClN7O7S	868.44	ABT-199
ChemDiv	6603-0104	CC(OC(=O)CN1C(=O)C(=O)c2c1c(C)ccc2)C	C14H15NO4	261.27	6603-0104
Vitas M Labs	STK155799	CCCCCC(=O)Nc1c(C)cc(cc1C)Br	C14H20BrNO	298.22	STK155799
Specs	AG-690/40699390	[illegible]]	C24H23N3O4	417.46	AG-690/40699390
ChemDiv	0075-0068	CN1CCN(CC1)c1ccc(cc1)Nc1c2ccccc2nc2c1cccc2	C24H24N4	368.47	0075-0068
Vitas M Labs	STL336112	COc1ccccc1N1CCN(CC1)c1ncnc2c1cnn2c1ccccc1	C22H22N6O	386.45	STL336112
MolMall Sarl	18650	CN1CCN(CC1)C(=S)SSC(=S)N1CCN(CC1)C	C12H22N4S4	350.59	18650
Bide Pharmatech	BD21816	Fc1ccc(cc1)[C@@H]1CCNC[C@H]1COc1ccc2c(c1)OCO2	C19H20FNO3	329.37	(3S,4R)-3-((Benzo[d][1,3]dioxol-5-yloxy)methyl)-4-(4-fluorophenyl)piperidine
Key Organics/BIONET - Screening and Fragments - Stock	AS-2259	OB(c1ccccc1)O	C6H7BO2	121.93	Phenylboronic acid
Cayman Chemical	12097	CN(c1ccc2c(c1)nn(c2C)C)c1ccnc(n1)Nc1ccc(c(c1)S(=O)(=O)N)C	C21H23N7O2S	437.52	Pazopanib
AbovChem	HY-10203	Clc1ccc(cc1)Nc1nnc(c2c1cccc2)Cc1ccncc1	C20H15ClN4	346.81	Vatalanib base
Vitas M Labs	STK416816	Fc1ccc(cc1)S(=O)(=O)n1c(C)nc2c1cccc2	C14H11FN2O2S	290.31	STK416816
ChemDiv	6030-2123	COc1ccc(cc1OC)CCNC(=O)CC(c1cccc(c1O)C)c1ccccc1	C26H29NO4	419.51	6030-2123
Vitas M Labs	STL019982	COC(=O)c1c(NC(=O)CC2SC(=N)NC2=O)sc(c1C)C	C13H15N3O4S2	341.41	STL019982
Vitas M Labs	STK066844	Clc1ccc(c(c1)C(=O)Nc1ccc(cc1)Cl)NS(=O)(=O)c1ccc(c(c1)[N+](=O)[O-])Cl	C19H12Cl3N3O5S	500.74	STK066844
Vitas M Labs	STL304557	CC1(C)OCc2c(C1)c1c(nc2N2CCOCC2)sc2c1ncnc2NCc1cccnc1	C24H26N6O2S	462.57	STL304557
ChemDiv	0988-0215	OC(CN1CCOCC1)COc1c(Br)cc(cc1Br)C(c1cc(Br)c(c(c1)Br)OCC(CN1CCOCC1)O)(C)C	C29H38Br4N2O6	830.24	0988-0215
Medchem Express	HY-10052	O=C1NC(CN2[C@H]([C@@H](O[C@@H](C3=CC(C(F)(F)F)=CC(C(F)(F)F)=C3)C)OCC2)C4=CC=C(F)C=C4)=NN1	C23H21F7N4O3	534.427	Aprepitant

TCI America	E1096	C#Cc1c[nH]c(=O)[nH]c1=O	C6H4N2O2	136.11	5-Ethynyluracil
Medchem Express	HY-A0033	NC(=O)C(c1ccccc1)(c1ccccc1)[C@@H]1CCN(C1)CCc1ccc2c(c1)CCO2	C28H30N2O2	426.55	Darifenacin
AbovChem	HY-B0109A	CCN[C@H]1C[C@H](C)S(=O)(=O)c2c1cc(s2)S(=O)(=O)N	C10H16N2O4S3	324.44	Dorzolamide
Enamine Screening Compounds	Z26397758	O=C(c1ccc2c(c1)OCO2)NC1CCCc2c1cccc2	C18H17NO3	295.33	Z26397758
Enamine Screening Compounds	Z32036776	O=C(c1n[nH]c2c1cccc2)Nc1ccc(cc1)N1CCOCC1	C18H18N4O2	322.36	Z32036776
Vitas M Labs	STK410281	COc1ccc(cc1)C(=O)Nc1sc(nc1C(=O)N)Nc1ccccc1	C18H16N4O3S	368.41	STK410281
ChemDiv	4577-1576	CCOc1ccc(cc1)S(=O)(=O)N(c1ccccc1)CC(=O)N/N=C/1\C(=O)Nc2c1cccc2	C24H22N4O5S	478.52	4577-1576
InterBioScreen	STOCK1N-65052	O=C(Nc1cccc(c1)C(=O)C)CN1CCc2c(C1)cc1c(c2)OCCCO1	C22H24N2O4	380.44	STOCK1N-65052
Vitas M Labs	STK810090	Clc1ccc(cc1)COc1ccc(cc1)c1nn(cc1/C=C/1\SC(=S)N(C1=O)CCS(=O)(=O)O)c1ccccc1	C28H22ClN3O5S3	612.14	STK810090
Medchem Express	HY-15319	O=C(N([C@@H](C1=NC2=NC=CC=C2C(N1C3=CC=C(OCC)C=C3)=O)C)CC4=CC=CN=C4)CC5=CC=C(OC(F)(F)F)C=C5	C32H28F3N5O4	603.591	AMG 487
Cayman Chemical	10008282	COc1cc(ccc1Cc1cn(c2c1cc(cc2)NC(=O)OC1CCCC1)C)C(=O)NS(=O)(=O)c1ccccc1C	C31H33N3O6S	575.68	Zafirlukast
AbovChem	HY-13009	OC(=O)C[C@@H]1CC[C@H](CC1)c1ccc(cc1)N1CCOc2c(C1=O)c(N)ncn2	C21H24N4O4	396.44	PF-04620110
AbovChem	HY-70050C	O=C1N(CCc2c1c1ccccc1n2C)Cc1nc[nH]c1C	C17H18N4O	294.35	Alosetron
Enamine Screening Compounds	Z55729757	C1CCn2c(CC1)nnc2C12CC3CC(C2)CC(C1)C3	C17H25N3	271.4	Z55729757
Vitas M Labs	STK841699	Oc1cccc(c1)NC(=O)c1cc2n(n1)c(C)cc(n2)c1ccccc1	C20H16N4O2	344.37	STK841699
ChemDiv	G748-0054	CC(=O)Nc1ccc(cc1)C(=O)Nc1sc(nc1C(=O)N)Nc1ccccc1	C19H17N5O3S	395.43	G748-0054
ChemDiv	D419-2236	CCCC(=O)Nc1nc(cc(n1)c1ccccc1)c1ccccc1	C20H19N3O	317.38	D419-2236
Life Chemicals	F0748-0306	COc1ccccc1c1nnc(s1)NC(=O)C(SC1=NCCS1)C	C15H16N4O2S3	380.51	F0748-0306
Medchem Express	HY-B0397	Clc1cc(cc(c1Cl)S(=O)(=O)N)S(=O)(=O)N	C6H6Cl2N2O4S2	305.16	Dichlorphenamide
Medchem Express	HY-12137	O=C(C1=CC=C(C(OC)=C1)NC2=NC=C(N(C3=O)C)C(N([C@@H]3CC)C(C)C)=N2)N[C@H]4CC[C@@H](CC4)N5CCN(CC5)CC6CC6	C34H50N8O3	618.813	Volasertib
Target Molecule	T1583	ONC(=O)CCCCCCC(=O)Nc1ccccc1	C14H20N2O3	264.32	Vorinostat
Target Molecule	T0373	COCCOc1cc2c(ncnc2cc1OCCOC)Nc1cccc(c1)C#C	C22H23N3O4	393.44	Erlotinib
Apollo Scientific - Biochemicals - Stock	BIMI2205	CC(C[C@@H]([C@H](CC(=O)N[C@H](C(=O)N[C@H]([C@H](CC(=O)O)O)CC(C)C)C)O)NC(=O)[C@H](C(C)C)NC(=O)[C@H](C(C)C)NC(=O)CC(C)C)C	C34H63N5O9	685.89	Pepstatin A
Maybridge	RDR01836	Cc1csc(n1)Nc1cccc2c1cccc2	C14H12N2S	240.32	RDR01836
Vitas M Labs	STK368746	CC(COC(=O)N(c1ccc(cc1)C(C(F)(F)F)(C(F)(F)F)O)C)C	C15H17F6NO3	373.29	STK368746
ChemDiv	K407-0490	COc1cc2c(cc1OC)CCN(C2COc1ccc(cc1)OC)C(=O)c1ccc(cc1)Cl	C26H26ClNO5	467.94	K407-0490
Enamine Screening Compounds	Z166688522	O=C(Nc1cc(C)cc(c1)C)Nc1ccc(cc1)S(=O)(=O)N	C15H17N3O3S	319.38	Z166688522
Maybridge	S06924	COc1ccc(c(c1)c1nc2ccccc2c(=O)o1)Br	C15H10BrNO3	332.15	S06924
AbovChem	HY-15599	COc1c2ccccn2c(c1C)C(=O)c1ccc(c(c1)C(=O)O)N	C18H16N2O4	324.33	SSR128129E (free acid)
Medchem Express	HY-15226A	O=C(N(C1CCCCC1)CCNCCC2=C(OCC(N3)=O)C3=C(O)C=C2)CCNCCC4=CC=C(Cl)C(Cl)=C4.O=C(O)C(F)(F)F.O=C(O)C(F)(F)F	C33H40Cl2F6N4O8	805.589	AZ505 (ditrifluoroacetate)

Cayman Chemical	20389	CCC(=O)NCC[C@@H]1CCc2c1c1CCOc1cc2	C16H21NO2	259.34	Ramelteon
Cayman Chemical	11313	O[C@@H]1[C@@H](O)CN2[C@@H]([C@H]1O)[C@@H](O)CC2	C8H15NO4	189.21	Castanospermine
Bide Pharmatech	BD123889	O=C1NC(=O)CN(C1)C[C@@H](N1CC(=O)NC(=O)C1)C	C11H16N4O4	268.27	Dexrazoxane
Maybridge	RH00511	CCCCc1ccc(c(c1)C)N/C=N/O	C12H18N2O	206.28	RH00511
Specs	AQ-390/43364023	CCn1c2ccc(c3c2c(c1=O)ccc3)S(=O)(=O)Nc1cccc(c1)C(=O)C	C21H18N2O4S	394.44	AQ-390/43364023
ChemDiv	K788-2368	Fc1ccc(cc1)S(=O)(=O)NCc1ccc(cc1)C(=O)NCc1cccnc1	C20H18FN3O3S	399.44	K788-2368
Enamine Screening Compounds	Z26426477	O=C(C1CC(=O)N(C1)Cc1ccccc1)NCc1ccc(cc1Cl)Cl	C19H18Cl2N2O2	377.26	Z26426477
Maybridge	JFD01049	N#CCOC(=O)c1ccc(cc1)c1cc(=O)c2c(o1)cccc2	C18H11NO4	305.28	JFD01049
Medchem Express	HY-10122	OCCCN1CCc2c1c(cc(c2)C[C@H](NCCOc1ccccc1OCC(F)(F)F)C)C(=O)N	C25H32F3N3O4	495.53	Silodosin
Medchem Express	HY-12828	O=C(C(N1C)=C(/C(C#N)=C\N)C2=C1C(Cl)=C(Cl)C=C2)OCC	C15H13Cl2N3O2	338.189	KH-CB19
Medchem Express	HY-12216	N#CC1=NN(C2=CC(Cl)=C(OC(C=C3C(C)C)=NNC3=O)C(Cl)=C2)C(NC1=O)=O	C17H12Cl2N6O4	435.221	MGL-3196
Medchem Express	HY-13635	O=C([C@H]1CC[C@]2([H])[C@]1(C)CC[C@]3([H])[C@@]4(C)C=CC(N[C@]4([H])CC[C@]32[H])=O)NC(C)(C)C	C23H36N2O2	372.544	Finasteride
Medchem Express	HY-16106	O=C1NN=C2C3=C1C=C(F)C=C3N[C@H](C4=CC=C(F)C=C4)[C@H]2C5=NC=NN5C	C19H14F2N6O	380.351	BMN-673
Medchem Express	HY-50671	FC1([C@H]2[C@@H]1C3=C([C@@H](C4=CC=CC=C42)N5CCN(CC5)C[C@H](COC6=C7C=CC=NC7=CC=C6)O)C=CC=C3)F.Cl.Cl.Cl	C32H34Cl3F2N3O2	636.987	Zosuquidar (trihydrochloride)
Medchem Express	HY-10095	O=C1N(CC2=C(N1)C=CC=C2)C3CCN(C(N[C@@H](C(N[C@H](C(N4CCN(C5=CC=NC=C5)CC4)=O)CCCCN)=O)CC6=CC(Br)=C(C(Br)=C6)O)=O)CC	C38H47Br2N9O5	869.645	Olcegepant
Medchem Express	HY-75766	OC1=C(O[C@H]2C3=O)C([C@]2(CC[N@@+]4(C)CC5CC5)[C@](CC3)(O)[C@H]4C6)=C6C=C1.[Br-]	C21H26BrNO4	436.339	Methylnaltrexone (Bromide)
Medchem Express	HY-50175	O=S(C1=CC(F)=CC2=C1N(C3=C2CC[C@@H]3CC(O)=O)CC4=CC=C(Cl)C=C4)(C)=O	C21H19ClFNO4S	435.896	Laropiprant
Medchem Express	HY-107382	NC([C@H](CC1=CC=CC=C1)NC([C@H](CCCNC(N)=N)NC(C23C[C@H](C4)C[C@H](C[C@H]4C3)C2)=O)=O)=O	C26H38N6O3	482.618	RF9
Medchem Express	HY-108316	O=S(C1=CC=C(N(CCCC2)S2(=O)=O)C=C1)(N)=O	C10H14N2O4S2	290.359	Sultiame
Medchem Express	HY-14937	CC(C)(C)C1=CC(SC(C)(C)SC2=CC(C(C)(C)C)=C(OC(CCC(O)=O)=O)C(C(C)(C)C)=C2)=CC(C(C)(C)C)=C1O	C35H52O5S2	616.914	Succinobucol
Medchem Express	HY-16276	N#CC1=CC=C([C@H]2CCC3=CN=CN32)C(F)=C1	C13H10FN3	227.237	Osilodrostat
Medchem Express	HY-B0154	O=C(CC)O[C@@]([C@]([C@@]1([H])C2)(C[C@H](O)[C@](F)([C@]3(C=C4)C)[C@@]1([H])C[C@H](F)C3=CC4=O)C)([C@@H]2C)C(SCF)=O	C25H31F3O5S	500.571	Fluticasone (propionate)
Medchem Express	HY-15720A	CC1=CN=CC2=C1C(S(=O)(N3[C@@H](C)CNCCC3)=O)=CC=C2.[H]Cl.[H]Cl	C16H23Cl2N3O2S	392.344	H-1152 (dihydrochloride)
Medchem Express	HY-B0355	O=C1[C@@H](C)[C@]2(O)[C@@]3(C4=O)C(C5([C@@H](O)C(O6)=O)C6O3)([C@H](O4)C[C@H]5C(C)(C)C)C[C@]2([H])O1	C20H24O9	408.399	Ginkgolide A
Medchem Express	HY-14183	COC1=C(OC)C=C(CCO[C@H]2[C@H](N3CC[C@@H](O)C3)CCCC2)C=C1.Cl	C20H32ClNO4	385.925	Vernakalant (Hydrochloride)
Medchem Express	HY-12050	NC1CCN(C2=C3N=C(C=CC3=CC=C2)N4C=NC5=CC(OCCOC)=CC=C54)CC1	C24H27N5O2	417.504	CP-673451
Medchem Express	HY-A0010	O=S(CCC1=CC2=C(NC=C2C[C@@H]3N(CCC3)C)C=C1)(C4=CC=CC=C4)=O.Br	C22H27BrN2O2S	463.431	Eletriptan (hydrobromide)
Medchem Express	HY-107377	C12=C(C=C3)C=C(C=CC=C4)C4=C1C=CC5=CC=CC3=C25	C20H12	252.309	Benzo[a]pyrene
Medchem Express	HY-103318	O=C(NC(C)(C)C)[C@@H](NC([C@H](CC(C)C)N(C)CC1=CC=C(C(C)(C)C)C=C1)=O)CC2=CC=C(OCC3=CC=CC=C3)C=C2	C38H53N3O3	599.846	PD173212
Medchem Express	HY-B1383	C[C@@]12[C@](C(COC(C)=O)=O)(O)CC[C@@]1([H])[C@]3([H])CCC4=CC(C=C[C@]4(C)[C@@]3(Cl)[C@@H](Cl)C2)=O	C23H28Cl2O5	455.371	Dichlorisone acetate
Medchem Express	HY-14929A	O[C@H]1[C@@H](CO)NC[C@H](O)[C@H]1O.Cl	C6H14ClNO4	199.633	Migalastat (hydrochloride)
Medchem Express	HY-16141	O=C(NCC(N[C@H](C(N[C@H](CC1=CC=CC=C1)C(N([C@H]2C(C)C)C)=O)=O)CC(O)=O)=O)[C@H](CCCNC(N)=N)NC2=O	C27H40N8O7	588.656	Cilengitide
Medchem Express	HY-15650	NC1=NC=NC2=C1C(Br)=CN2[C@H]3[C@H](O)[C@H](O)[C@@H](CN(CCCNC(NC4=CC=C(C(C)(C)C)C=C4)=O)C(C)C)O3	C28H40BrN7O4	618.566	SGC0946
Medchem Express	HY-15449	O=C1C(O)=C(C2=CC=C(OC)C=C2)OC3=CC(O)=CC(O)=C13	C16H12O6	300.263	Kaempferide

Medchem Express	HY-15512A	ClC1=CC(C2=CC=C(N=CC(C(C)=O)=C3N[C@H]4CC[C@H](CN(C)C)CC4)C3=N2)=CC(Cl)=C1O.[H]Cl	C25H29Cl3N4O2	523.882	OTSSP167 (hydrochloride)
Medchem Express	HY-50855	O=C(C1=CC=C2C3=C(C(NC4=CC=CC(Cl)=C4)=NC2=C1)C=CN=C3)O	C19H12ClN3O2	349.771	CX-4945
Medchem Express	HY-10005	O=C1C2=C(C=C(C([C@]3([H])[C@H](O)CN(C)CC3)=C2OC(C4=CC=CC=C4Cl)=C1)O)O	C21H20ClNO5	401.84	Flavopiridol
Medchem Express	HY-12116	N[C@@H](CCCCNC(C)=N)C(O)=O	C8H17N3O2	187.24	L-NIL
Medchem Express	HY-107374	NCCC1=C(C=CC=C1Cl)Cl	C8H9Cl2N	190.07	Phenethylamine, 2,6-dichloro-
Medchem Express	HY-107378	O=C([C@@H](N)C(C)C)N[C@H](C(O)=O)CC1=CC=CC=C1	C14H20N2O3	264.32	L-Valyl-L-phenylalanine
Medchem Express	HY-13683	C[C@@]12[C@@](C#CC)(O)CC[C@@]1([H])[C@]3([H])CCC4=CC(CCC4=C3[C@@H](C5=CC=C(N(C)C)C=C5)C2)=O	C29H35NO2	429.594	Mifepristone
Medchem Express	HY-13022	C1(C2=CC=CC(OCCN3CCCCC3)=C2)=NNC4=C1C=C(C=C4)C5=NC=NN5.Cl	C22H25ClN6O	424.927	CC-401 (hydrochloride)
Medchem Express	HY-100452	CC(C)(C)[C@H](NCC1=CC=NC=C1)C(N2CCC3=C(C=C(OC)C(OC)=C3)CC2)=O	C23H31N3O3	397.511	TCS-OX2-29
Medchem Express	HY-14598	OC1=CC=C(/C(CC)=C(CC)/C2=CC=C(O)C=C2)C=C1	C18H20O2	268.35	Diethylstilbestrol
Medchem Express	HY-A0142A	CC1=CC=CC=C1N2CCN(CCC3=NN=C4CCCCN43)CC2.[H]Cl	C19H28ClN5	361.912	Dapiprazole (hydrochloride)
Medchem Express	HY-N0176	O[C@@H]1[C@H](C)[C@]2([H])CC[C@@H](C)[C@]3([H])CC[C@@](O4)(C)OO[C@]32[C@]4([H])O1	C15H24O5	284.348	Dihydroartemisinin
Medchem Express	HY-B0305A	O=C(NCCCOC1=CC=CC(CN2CCCCC2)=C1)COC(C)=O.Cl	C19H29ClN2O4	384.898	Roxatidine (Acetate Hydrochloride)
Medchem Express	HY-108323	O=C(NCC(N[C@H]1CN(CC2=CC=C(Cl)C=C2)CC1)=O)C3=CC=CC(C(F)(F)F)=C3	C21H21ClF3N3O2	439.859	Teijin compound 1
Medchem Express	HY-108322	OC[C@@H]1[C@H]([C@H]([C@H](N2C=NC3=C2N=C(N)N=C3SCC4=CC=C([N+]([O-])=O)C=C4)O1)O)O	C17H18N6O6S	434.426	NBTGR
Medchem Express	HY-12359	O=C(NC1=CC=C(N(C)[C@@H](C(C)C)C(N[C@H](CO)C2)=O)C2=C1)/C=C/C=C/C3=CC=C(C(F)(F)F)C=C3	C27H30F3N3O3	501.541	TPPB
Medchem Express	HY-13447A	O=C(N(CCC1=CC=CC=C1OC)[C@H]2CNCC2)C3=CC=C(CN(CC)CC)C=C3.[H]Cl.[H]Cl	C25H37Cl2N3O2	482.486	PF429242 (dihydrochloride)
Medchem Express	HY-B0741A	O=C(N1CCN(C)CC1)O[C@@H]2N(C3=NC=C(Cl)C=C3)C(C4=NC=CN=C42)=O	C17H17ClN6O3	388.808	Zopiclone (S-enantiomer)
Medchem Express	HY-14722A	FC1=C(CN2CCOCC2)C(F)=CC(C3=C4N=C(C5=CN(C6CCNCC6)N=C5)C=NC4=CC=C3)=C1.Cl.Cl	C27H30Cl2F2N6O	563.47	NVP-BSK805 (dihydrochloride)
Medchem Express	HY-B0426A	O=C(O)CC1=CC=C(C/2=C1)OCC3=CC=CC=C3C2=C\CCN(C)C.Cl	C21H24ClNO3	373.873	Olopatadine (hydrochloride)
Medchem Express	HY-15699	N[C@@H](C1)CCCN1[C@@H]2CC3=CC=CC=C3[C@H]2OC4=C(Cl)C=C(C#N)C=C4	C21H22ClN3O	367.872	SAR7334
Medchem Express	HY-14985	O=C(N)C1=CC=CN=C1NC2=NC(C3=CC=C(C(OC)=C3)OC)=CC4=NC=CN24.[H]Cl.[H]Cl	C20H20Cl2N6O3	463.317	BAY 61-3606 (dihydrochloride)
Medchem Express	HY-50101A	[H]Cl.[H]Cl.NCCCCN(CC1=NC2=CC=CC=C2N1)[C@@H]3C4=NC=CC=C4CCC3.[H]Cl	C21H30Cl3N5	458.855	AMD-070 (hydrochloride)
Medchem Express	HY-P1033	O=C([C@@H](N1)CCC1=O)C[C@@H](CCCNC(N)=N)C(N2[C@@H](CCC2)C(N[C@@H](CCCNC(N)=N)C(N[C@@H](CC(C)C)C(N[C@@H](CO)C(N[C@H](C(N[C@@H](CCCCN)C(NCC(N3[C@@H](CCC3)C(N[C@@H](CCSC)C(N4[C@@H](CCC4)C(N[C@H](C(O)=O)CC5=CC=CC=C5)=O)=O)=O)=O)=O)=O)CC6=CNC=N6)=O)=O)=O)=O)=O	C70H109N21O16S	1532.811	[Pyr1]-Apelin-13
Medchem Express	HY-P0195	O=C(N[C@@H](C)C(N[C@@H](C(C)C)C(NCC(N[C@H](C(N[C@@H](CC(C)C)C(N[C@H](C(N)=O)CCSC)=O)=O)CC1=CN=CN1)=O)=O)=O)[C@@H](NC([C@H](CCC(N)=O)NC([C@H](CC(N)=O)NC(CNC([C@H](CC(C)C)NC([C@H](CCCNC(N)=N)NC([C@H](CCC(N)=O)NC([C@@H](N2)CCC2=O)=O)=O)=O)=O)=O)=O)=O)CC3=CNC4=CC=CC=C34	C71H110N24O18S	1619.848	Bombesin
Medchem Express	HY-N1394	O=C(O)C1=CC=C(OC)C=C1	C8H8O3	152.147	4-Methoxybenzoic acid
Medchem Express	HY-13771	C[C@H](CCC(O)=O)[C@H]1CC[C@@]2([H])[C@]3([H])[C@@H](O)C[C@]4([H])C[C@H](O)CC[C@]4(C)[C@@]3([H])CC[C@]12C	C24H40O4	392.572	Ursodiol
Medchem Express	HY-101769	O=C(N1CCCCC1)[C@@H](N)CCNCC2=CC=C(Cl)C=C2.[H]Cl.[H]Cl	C16H26Cl3N3O	382.756	UAMC00039 (dihydrochloride)
Medchem Express	HY-10162	O=C(C1=CC(CC(C2=C3C=CC=C2)=NNC3=O)=CC=C1F)N4CCN(C(C5CC5)=O)CC4	C24H23FN4O3	434.463	Olaparib
Medchem Express	HY-14984	OC(CCC1=CC=C(N1C2=CC=C(C(N)=O)C=C2C)C(C=C3)=CC=C3N4C=CN=C4)=O	C24H22N4O3	414.457	N6022
Medchem Express	HY-14301	O=C1NC2=CC(O)=CC([C@@H](O)CNC(C)(C)CC3=CC=C(OC)C=C3)=C2OC1	C21H26N2O5	386.442	Olodaterol

Medchem Express	HY-B0251	O=C(CC1)O[C@@]21CC[C@@]3([H])[C@]4([H])[C@H](C(OC)=O)CC5=CC(CC[C@]5(C)[C@@]46[C@H](O6)C[C@@]32C)=O	C24H30O6	414.491	Eplerenone
Medchem Express	HY-50919	C[C@]([C@]([C@H](C)/C=C/[C@H](C)C(C)(C)O)([H])CC1)(CCC/2)[C@]1([H])C2=C\C=C3C[C@@H](O)C[C@H](O)C/3	C27H44O3	416.637	Paricalcitol
Medchem Express	HY-100441	OC(COC1=CC=CC2=C1C[C@](C[C@@H](O)[C@@H]3CC[C@@H](O)CCCCC)([H])C[C@]3([H])C2)=O	C23H34O5	390.513	Treprostinil
Medchem Express	HY-108313	CC1=NC2=C(C3=CC=C(OC)C(OC)=C3)C(C)=NN2C(NCCN4CCOCC4)=C1	C22H29N5O3	411.497	T-00127_HEV1
Medchem Express	HY-A0070A	OC1=C(I)C=C(OC2=C(I)C=C(C[C@H](N)C(O)=O)C=C2I)C=C1	C15H12I3NO4	650.974	3,3',5-Triiodo-L-thyronine
Medchem Express	HY-10195B	O=C1NC(C(C2=CN(C3=CC=CC=C32)CCO[C@H](CN(C)C)CC4)=C1C5=CN4C6=CC=CC=C56)=O.[H]Cl	C28H29ClN4O3	505.008	Ruboxistaurin (hydrochloride)
Medchem Express	HY-17446	3N(C([C@H](CO)NC([C@H](CC4=CC=CS4)NC(CNC([C@@H]5C[C@@H](O)CN5C([C@H]6N(C([C@H](CCCNC(N)=N)NC([C@H](N)CCCNC(N)=N)=O)=O)CCC6)=O)=O)=O)=O)=O)CC7=C(C=CC=C7)C3)=O)[C@]2([H])CCCC1	C59H89N19O13S	1304.522	Icatibant
Medchem Express	HY-12588	O[C@H]1[C@H](O)[C@@]2([H])N=C(NCC)S[C@@]2([H])O[C@@H]1CO	C9H16N2O4S	248.299	Thiamet G
Medchem Express	HY-50759	O=C1N(CC2=CC=CC=C2)C([C@@H](C(C)C)N(CCCN)C(C3=CC=C(C)C=C3)=O)=NC4=CC(Cl)=CC=C14	C30H33ClN4O2	517.062	Ispinesib
Medchem Express	HY-10969	COC1=CC(C2=CC3=C(C=CC=C3)N2)=N/C1=C\C4=C(C=C(N4)C)C.CS(=O)(O)=O	C21H23N3O4S	413.49	Obatoclax
Medchem Express	HY-B0600	O=C(OC(C)C)CCC/C=C\C[C@@H]1[C@@H](/C=C/C(F)(F)COC2=CC=CC=C2)[C@H](O)C[C@@H]1O	C25H34F2O5	452.531	Tafluprost
Medchem Express	HY-B1480	O=S(C1=NC2=CC=C(OCC)C=C2S1)(N)=O	C9H10N2O3S2	258.317	Ethoxzolamide
Medchem Express	HY-13755	O=S(CCCCN=C=S)C	C6H11NOS2	177.288	Sulforaphane
Medchem Express	HY-P0197	O=C(N[C@@H](C(C)C)C(NCC(N[C@@H](CC(C)C)C(N[C@H](C(N)=O)CCSC)=O)=O)=O)[C@@H](NC([C@H](CO)NC([C@H](CC(O)=O)NC([C@@]([C@H](O)C)([H])NC([C@H](CCCCN)NC([C@@H](N)CC1=CN=CN1)=O)=O)=O)=O)=O)CC2=CC=CC=C2	C50H80N14O14S	1133.321	Neurokinin A
Medchem Express	HY-18077	COC1=CC2=C(C=C1OC)C=CN=C2CC3=CC=C(OC)C(OC)=C3	C20H21NO4	339.385	Papaverine
Medchem Express	HY-13004	O=C(C1CCC(F)(F)CC1)N[C@H](C2=CC=CC=C2)CCN3[C@H]4C[C@@H](N5C(C)=NN=C5C(C)C)C[C@@H]3CC4	C29H41F2N5O	513.666	Maraviroc
Medchem Express	HY-15872A	CSCC[C@@H](C(OC)=O)NC(C1=CC=C(NC[C@@H](N)CS)C=C1C2=CC=CC=C2)=O.[H]Cl	C22H30ClN3O3S2	484.075	FTI-277 (hydrochloride)
Medchem Express	HY-14127	CCCN(C1=CC(C)=NC2=C(C3=CN=C(N(C)C)C=C3C)C(C)=NN21)CCC	C22H32N6	380.53	R121919
Medchem Express	HY-15292	COC1=CC=C2C(CN(C)CCS2)=C1	C11H15NOS	209.308	S107
Medchem Express	HY-17412	OC1=C(C(N)=O)C([C@@]2(O)C(O)=C3C(C4=C(O)C=CC(N(C)C)=C4C[C@@]3([H])C[C@@]2([H])[C@@H]1N(C)C)=O)=O.Cl	C23H28ClN3O7	493.937	Minocycline (hydrochloride)
Medchem Express	HY-14992	O=C1N=C(NN2C1=C(N=C2[C@@H](CCCC3=CC=CC=C3)[C@@H](C)O)C)CC4=CC=C(C(OC)=C4)OC	C27H32N4O4	476.567	Bay 60-7550
Medchem Express	HY-P0224	O=C(O)[C@H](CC1=CC=CC=C1)NC([C@H](CC(C)C)NC([C@H](CCSC)NC=O)=O)=O	C21H31N3O5S	437.553	N-Formyl-Met-Leu-Phe
Medchem Express	HY-B1334A	O=C(O)/C=C\C(O)=O.C1(CC(C2CCCCC2)C3CCCCC3)NCCCC1	C23H39NO4	393.56	Perhexiline maleate
Medchem Express	HY-108321	O=C(O)[C@H]1N(C([C@H](C)CSC(C2=CC=CC=C2)=O)=O)C[C@@H](SC3=CC=CC=C3)C1	C22H23NO4S2	429.552	Zofenopril
Medchem Express	HY-107372	O[C@H]1[C@H](N2C(NC(C=C2)=O)=O)O[C@H](COP(OP(OP(O)(O)=O)(O)=O)(O)=O)[C@H]1O	C9H15N2O15P3	484.141	Uridine triphosphate
Medchem Express	HY-B0374	CC1=NC(OC)=C(NC2=NCCN2)C(Cl)=N1	C9H12ClN5O	241.678	Moxonidine
Medchem Express	HY-10234	ClC1=CC=C2C(OCO2)=C1NC3=C4C(OC5CCOCC5)=CC(OCCN6CCN(C)CC6)=CC4=NC=N3	C27H32ClN5O5	542.027	Saracatinib
Medchem Express	HY-17368	O=C(N(C)CC)OC1=CC=CC([C@H](C)N(C)C)=C1	C14H22N2O2	250.337	Rivastigmine
Medchem Express	HY-13803	O=C(C1=CC(C2=CC=C(CN3CCOCC3)C=C2)=CC(N(CC)C4CCOCC4)=C1C)NCC5=C(C)C=C(C)NC5=O	C34H44N4O4	572.738	EPZ-6438
Medchem Express	HY-15656	CC(C)OC1=CC(C2CCNCC2)=C(C)C=C1NC3=NC=C(Cl)C(NC4=CC=CC=C4S(=O)(C(C)C)=O)=N3	C28H36ClN5O3S	558.135	LDK378
Medchem Express	HY-B0180	NC1=NC2=CC=CC=C2C3=C1N=CN3CC(C)C	C14H16N4	240.304	Imiquimod
Medchem Express	HY-17040	O=C(O[C@@H]1[C@@]2([H])[C@@](OCC2)([H])OC1)N[C@@H](CC3=CC=CC=C3)[C@H](O)CN(S(=O)(C4=CC=C(N)C=C4)=O)CC(C)C	C27H37N3O7S	547.664	Darunavir

Medchem Express	HY-13677	S=C1NC=NC2=C1NC=N2	C5H4N4S	152.177	6-Mercaptopurine
Medchem Express	HY-14185	O=C(C1=C(Cl)C=C(NC(C2=CC(F)=CC=C2C)=O)C=C1)N(C3)C4=CC=CC=C4CN5C3=CC=C5	C27H21ClFN3O2	473.926	Lixivaptan
Medchem Express	HY-50698	O=C1[C@H](N(C2=C(N1C)C=NC(NC3=CC=C(C(NC4CCN(C)CC4)=O)C=C3OC)=N2)C5CCCC5)CC	C28H39N7O3	521.654	BI 2536
Medchem Express	HY-17355	NC(S1)=NC2=C1C[C@@H](NCCC)CC2.[H]Cl.[H]Cl	C10H19Cl2N3S	284.249	Pramipexole (dihydrochloride)
Medchem Express	HY-12787	OC1=CC(C2=C(C3=CC=NC=C3)N=C(C4=CC=CC=C4)N2)=CC=C1Cl	C20H14ClN3O	347.798	L-779450
Medchem Express	HY-15270	O=C(C1=CC2=C(N(C)C=N3)C3=C(NC4=NN(C)C(C)=C4)N=C2N1CC)N(C5CC5)C6CC6	C23H28N8O	432.521	BMS-911543
Medchem Express	HY-13203	O=C(C1=C(C=CC=C1)NC2=NC(NC3=C(C=C(C=C3)N4CCOCC4)OC)=NC=C2Cl)NC	C23H25ClN6O3	468.936	NVP-TAE 226
Medchem Express	HY-15593	NC1=NC=NC2=C1N=CN2[C@H]3[C@H](O)[C@H](O)[C@@H](CN([C@@H]4C[C@H](CCC5=NC6=C(C=CC(C(C)(C)C)=C6)N5)C4)C(C)C)O3	C30H42N8O3	562.706	EPZ-5676
Medchem Express	HY-12090	O=C1O[C@@H]([C@@H](N1CC2=CC(C(F)(F)F)=CC=C2C3=CC(C(C)C)=C(C=C3OC)F)C)C4=CC(C(F)(F)F)=CC(C(F)(F)F)=C4	C30H25F10NO3	637.508	Anacetrapib
Medchem Express	HY-15722	O=C(C1=C(C)C(C2=CC=CC=C2)=NC3=CC=CC=C13)N[C@H](C4=CC=CC=C4)CC	C26H24N2O	380.482	SB-222200
Medchem Express	HY-13287	CN1CCC(CC1)CNC2=NN3C(C=C2)=NC=C3C4=CC=CC(OC(F)(F)F)=C4	C20H22F3N5O	405.417	SGI-1776
Medchem Express	HY-15388	O=C(C1=CC=C(C#CC2=CC=C3C(C(C)(C)CCS3)=C2)N=C1)OCC	C21H21NO2S	351.462	Tazarotene
Medchem Express	HY-14171	CC1(C2=C(C(C)(CC1)C)C=C(C(C(C3=CC=C(C=C3)C(O)=O)=C)=C2)C)C	C24H28O2	348.478	Bexarotene
Medchem Express	HY-B0246	O=C(N1C2=CC=CC=C2C=CC3=CC=CC=C31)N	C15H12N2O	236.269	Carbamazepine
Medchem Express	HY-10127	O=C(NC1=CC=CC(F)=C1)CC2=CC(NC3=C4C=CC(OCCCN(CC)CCOP(O)(O)=O)=CC4=NC=N3)=NN2	C26H31FN7O6P	587.54	AZD1152
Medchem Express	HY-16294	O=C(C(C1=CN2CCN(C(N3CCCCC3)=O)CC4=CC(F)=CC1=C42)=C5C6=CN=C7C=CC=CN76)NC5=O	C28H25FN6O3	512.535	LY2090314
Medchem Express	HY-14184	BrC1=CN=C(OCCOC2=C(C3=CC=C(Br)C=C3)C(NS(NCCC)(=O)=O)=NC=N2)N=C1	C19H20Br2N6O4S	588.273	Macitentan
Medchem Express	HY-16692	O=C(OC)CC1ON=C(C(C=C2)=CC=C2O)C1	C12H13NO4	235.236	ISO-1
Medchem Express	HY-12246	O=C(O)C1=CC(C(F)(F)F)=CC=C1N2C[C@@H](OCC3=NC=CC=C3)CC2	C18H17F3N2O3	366.334	XEN445
Medchem Express	HY-17416	O=C(NC(N)=N)CC1=C(Cl)C=CC=C1Cl.[H]Cl	C9H10Cl3N3O	282.554	Guanfacine (hydrochloride)
Medchem Express	HY-13734	CCC(SSC1=NC=CN1)C	C7H12N2S2	188.314	PX-12
Medchem Express	HY-15388	O=C(C1=CC=C(C#CC2=CC=C3C(C(C)(C)CCS3)=C2)N=C1)OCC	C21H21NO2S	351.462	Tazarotene
Medchem Express	HY-15433	O=C(C1=CN=C(N2CCC(CNCC3=CN(C)C4=C3C=CC=C4)CC2)N=C1)NO	C21H26N6O2	394.47	Quisinostat
Medchem Express	HY-13227	FC1=CC=C(Cl)C=C1C2=NC3=NC=CN=C3C(NC4=CC=NC=C4)=N2	C17H10ClFN6	352.753	SD-208
Medchem Express	HY-13688	O=C(NC(C=C1C2=C3C=CC=C2)=CC=C1NC3=O)CN(C)C.Cl	C17H18ClN3O2	331.797	PJ34 (hydrochloride)
Medchem Express	HY-13749	O=C(N1CC2=NN=C(C(F)(F)F)N2CC1)C[C@H](N)CC3=CC(F)=C(F)C=C3F	C16H15F6N5O	407.314	Sitagliptin
Medchem Express	HY-15202	BrC1=CC=C(C(F)=C1)NC2=C(C3=C(C=C2C(NOCCO)=O)N(C=N3)C)F	C17H15BrF2N4O3	441.227	MEK162
Medchem Express	HY-10320	O=C(NC1=CC(C(C)(C)C)=NN1C2=CC=C(C)C=C2)NC3=C4C=CC=CC4=C(OCCN5CCOCC5)C=C3	C31H37N5O3	527.657	Doramapimod
Medchem Express	HY-13104	S=C(NCCCCNC(NC1=CC(N=C=S)=CC=C1)=S)NC2=CC(N=C=S)=CC=C2	C20H20N6S4	472.673	MRS 2578
Medchem Express	HY-B0602A	OC1=CC=C(C(C2(O)CCCCC2)CN(C)C)C=C1.O=C(O)CCC(O)=O.O	C20H33NO7	399.479	Desvenlafaxine (succinate hydrate)
Medchem Express	HY-14440	O=C(C1=CC=CN(CC2=CC=C(F)C(F)=C2)C1=O)N[C@H](C3=CC=CC=C3)COC4=CC=C(N5)C(NC5=O)=C4	C28H22F2N4O4	516.496	PDK1 inhibitor
Medchem Express	HY-10038	O=C(NC1=CC=C(C2=CC=C(C([C@@H]3CCC[C@H]3C(O)=O)=O)C=C2)C=C1)NC4=CC=CC=C4	C26H24N2O4	428.48	A 922500
Medchem Express	HY-15315	N#CCC1(N2N=CC(C3=C4C(NC=C4)=NC=N3)=C2)CN(S(=O)(CC)=O)C1	C16H17N7O2S	371.417	Baricitinib
Medchem Express	HY-13258	CC1=CC=C(C=C1NC2=NC(C3=CC=CN=C3)=NC4=C2C=NN4C)C(NC5=CC=CC(C(F)(F)F)=C5)=O	C26H20F3N7O	503.479	NVP-BHG712

Medchem Express	HY-14715B	[H]Cl.COC1=C(OC)C=C(C(N[C@H]2[C@H](C(C)(C)O)CNC2)=NC(C3=C(O)C=CC(F)=C3)=N4)C4=C1	C23H28ClFN4O4	478.944	CCT241533 (hydrochloride)
Medchem Express	HY-15798	CCCCNC1=NC(N[C@H]2CC[C@@H](O)CC2)=C(C(NCC3=CC=C(N4C=NC=C4)C=C3)=O)C=N1	C25H33N7O2	463.575	UNC2881
Medchem Express	HY-14914	O=C(C1=C2C(N=C(OCC)N2CC3=CC=C(C4=CC=CC=C4C(N5)=NOC5=O)C=C3)=CC=C1)O	C25H20N4O5	456.45	Azilsartan
Medchem Express	HY-13902	NC1=NC=C(C2=CC=C(S(=O)(C(C)C)=O)C=C2)N=C1C3=CC(C4=CC=C(CNC)C=C4)=NO3	C24H25N5O3S	463.552	VE-822
Medchem Express	HY-16975	O=C(O)C1=CC=C(N(CC2=CC=C(C3CCCCC3)C=C2)C(CN(C)S(=O)(C4=C(F)C(F)=C(F)C(F)=C4F)=O)=O)C=C1	C29H27F5N2O5S	610.592	SH-4-54
Medchem Express	HY-15320	O=C(N([C@@H](C1=NC2=NC=CC=C2C(N1C3=CC=C(OCC)C=C3)=O)C)CC4=CC=CN=C4)CC5=CC=C(F)C(C(F)(F)F)=C5	C32H27F4N5O3	605.582	NBI-74330
Medchem Express	HY-13075	CN(CC1)CCN1C2=CC=C(NC(C3=CC(C#N)=CN3)=O)C(N4CCC(C)CC4)=C2	C23H30N6O	406.524	c-FMS inhibitor
Medchem Express	HY-17572	O=C([C@H](CSSCCC(N[C@@H](C1=O)CC2=CC=C(OCC)C=C2)=O)NC([C@@H](NC(C(NC([C@](N1)([H])[C@@H](C)CC)=O)[C@H](O)C)=O)CC(N)=O)=O)N(CCC3)[C@@H]3C(N[C@@H](CCCN)C(NCC(N)=O)=O)=O	C43H67N11O12S2	994.189	Atosiban
Medchem Express	HY-50907	CN(CC[C@@H](NC1=CC=C(C=C1[N+]([O-])=O)S(NC(C2=CC=C(C=C2)N3CCN(CC3)CC4=CC=CC=C4C5=CC=C(C=C5)Cl)=O)(=O)=O)CSC6=CC=CC=C6)C	C42H45ClN6O5S2	813.427	ABT-737
Medchem Express	HY-10109	O=C(NC/1=O)SC1=C/C2=CC=C3N=CC=NC3=C2	C12H7N3O2S	257.268	AS-605240
Medchem Express	HY-12282	FC(C1=CN=C(NC2=C(Cl)N([C@@H]3[C@@H](F)CN(C4COC4)CC3)N=C2)N=C1NC)(F)F	C17H20ClF4N7O	449.834	GNE-9605
Medchem Express	HY-15291	CCC1=CN=C(N2CCC(C3=NC(COC4=CC=C(N5N=NN=C5)C=C4)=CS3)CC2)N=C1	C22H24N8OS	448.544	MBX-2982
Medchem Express	HY-19351	O=C1C=C(C2=CC=C(C(C)C)C=C2)OC3=CC=CC=C13	C18H16O2	264.319	MN-64
Medchem Express	HY-10262	O=C1C(C2=NC3=CC(N4CCOCC4)=CC(C)=C3N2)=C(C=CN1)NC[C@@H](O)C5=CC=CC(Cl)=C5	C25H26ClN5O3	479.959	BMS-536924
Medchem Express	HY-13315	ClC1=CC2=C(C=C1)C=CC(/C=C/C3=CC([C@H](SCC4(CC([O-])=O)CC4)CCC5=CC=CC=C5C(C)(O)C)=CC=C3)=N2.[Na+]	C35H35ClNNaO3S	608.165	Montelukast (sodium)
Medchem Express	HY-15224	COC(C=C1)=CC=C1CN2C3=CC(C(NO)=O)=CC=C3C=C2	C17H16N2O3	296.321	PCI-34051
Medchem Express	HY-13340	CC1=C2C(SC(C(NCC3=CC=C(OC)C=C3)=O)=C2N)=NC(C)=C1	C18H19N3O2S	341.427	VU0152100
Medchem Express	HY-A0021	O=C1N(C[C@@]([H])(CCC2)C3=C2C=CC=C13)[C@@H]4CN5CCC4CC5.[H]Cl	C19H25ClN2O	332.868	Palonosetron (Hydrochloride)
Medchem Express	HY-14397	COC1=CC=C(N(C(C2=CC=C(Cl)C=C2)=O)C(C)=C3CC(O)=O)C3=C1	C19H16ClNO4	357.788	Indomethacin
Medchem Express	HY-18314	O=C1/C(C2=NC=CC=C2N1)=C\C3=CN(C)C4=C3C=CC=C4	C17H13N3O	275.305	GW 441756
Medchem Express	HY-15452	O=C(C1C(NC2=C3C=C(Cl)C=C2)=C3CCC1)N	C13H13ClN2O	248.708	EX-527
Medchem Express	HY-10230	O=C(C1=CC=CC=C1)N(C)[C@H]2[C@@H](OC)[C@@]3(C)N(C4=C5C=CC=C4)C6=C5C7=C(C(NC7)=O)C8=C6N(C9=CC=CC=C98)[C@@](O3)([H])C2	C35H30N4O4	570.637	PKC412
Medchem Express	HY-13820	CC1=CC=CC(CC(N2CCC3=C2C=CC(C4=CN(C)C5=NC=NC(N)=C54)=C3F)=O)=N1	C23H21FN6O	416.451	GSK2656157
Medchem Express	HY-13805	NC1=C2C(N(C(C)(C)C)N=C2C3=CC=C(Cl)C=C3)=NC=N1	C15H16ClN5	301.774	PP2
Medchem Express	HY-10069	O=C(C1=CC=C([C@@H](C)N)C=C1)NC2=C3C(NC=C3)=NC=C2.[H]Cl.[H]Cl	C16H18Cl2N4O	353.246	Y-33075 (dihydrochloride)
Medchem Express	HY-13030	O=C(C[C@H]1C2=NN=C(N2C3=C(C(C4=CC=C(C=C4)Cl)=N1)C(C)=C(S3)C)C)OC(C)(C)C	C23H25ClN4O2S	456.988	(+)-JQ-1
Medchem Express	HY-16425	O=C(NCCCCCC(NC1=CC=CC=C1N)=O)C2=CC=C(C)C=C2	C20H25N3O2	339.431	RG2833
Medchem Express	HY-12041	O=C1C2=C3C(NN=C3C4=C1C=CC=C4)=CC=C2	C14H8N2O	220.226	SP600125
Medchem Express	HY-14711	C1(NC2=CC=C(N3CCOCC3)C=C2)=NC(NC4CCCCC4)=C5C(NC=N5)=N1	C21H27N7O	393.485	Reversine
Medchem Express	HY-B0272	OC(C(/C=N/N1CCN(C)CC1)=C(NC(/C(C)=C\C=C\[C@@H]([C@@H]([C@@H](C)[C@H]2O)O)C)=O)C(O)=C3C(O)=C4C)=C3C5=C4O[C@](C)(O/C=C/[C@@H]([C@H]([C@@]([C@@H]2C)([H])OC(C)=O)C)OC)C5=O	C43H58N4O12	822.94	Rifampicin
Medchem Express	HY-12529	O=C1N=C(NCC2=CC=CS2)S/C1=C\C3=CC=C4N=CC=CC4=C3	C18H13N3OS2	351.445	Ro-3306
Medchem Express	HY-15323	NC(C1=CN=C(N[C@H]2[C@@H](N)CCCC2)N=C1NC3=CC=CC(N4N=CC=N4)=C3)=O.[H]Cl	C19H24ClN9O	429.907	PRT062607 (Hydrochloride)

Medchem Express	HY-B0553	CC(/N=C1SC(S(=O)(N)=O)=NN/1C)=O	C5H8N4O3S2	236.272	Methazolamide
Medchem Express	HY-10013	ClC1=CC=C(C[C@@H](C2=CC=CC(C#N)=C2)[C@@H](NC(C(C)(OC3=CC=C(C=N3)C(F)(F)F)C)=O)C)C=C1	C27H25ClF3N3O2	515.955	Taranabant
Medchem Express	HY-10042	N#CC1(NC([C@@H](N[C@H](C(F)(F)F)C2=CC=C(C3=CC=C(S(=O)(C)=O)C=C3)C=C2)CC(C)(F)C)=O)CC1	C25H27F4N3O3S	525.559	Odanacatib
Medchem Express	HY-13756	O=C([C@@](CCCC1)([H])N1C(C([C@@]2(O)[C@H](C)C[C@H](OC)[C@@](O2)([H])[C@@H](OC)C[C@@H](C)C/C(C)=C/[C@H]3CC=C)=O)=O)O[C@H](/C(C)=C/[C@H]4C[C@@H](OC)[C@H](O)CC4)[C@H](C)[C@@H](O)CC3=O	C44H69NO12	804.018	Tacrolimus
Medchem Express	HY-B0121	O=C(O)CCC(O)=O.O=S(CC1=CC2=C(NC=C2CCN(C)C)C=C1)(NC)=O	C18H27N3O6S	413.489	Sumatriptan (succinate)
Medchem Express	HY-10037	O=C(O)C(C)(C)CC1=C(SC(C)(C)C)C2=C(C=CC(OCC3=NC4=CC=CC=C4C=C3)=C2)N1CC5=CC=C(Cl)C=C5	C34H35ClN2O3S	587.171	MK-0591
Medchem Express	HY-10302	O=C1C2(NC[C@@H](C3=CC(F)=CC(F)=C3)N1CC(NC4=CC5=C(C[C@@]6(C(NC7=C6C=CC=N7)=O)C5)C=C4)=O)CCCC2.Cl	C31H30ClF2N5O3	594.051	MK-3207 (Hydrochloride)
Medchem Express	HY-18978	OC[C@@H]1[C@H]([C@H]([C@H](N2C=NC3=C2N=CN=C3N[C@@H]4[C@@H](O)CCC4)O1)O)O	C15H21N5O5	351.358	GR79236
Medchem Express	HY-16162A	[H]Cl.O=C1N(CCN)C2=CC(OC)=CC=C2N1C([C@H]3[C@H](C(C)C)CC[C@@H](C)C3)=O	C21H32ClN3O3	409.95	D-3263 (hydrochloride)
Medchem Express	HY-B0697	C[C@]1([C@](CC2)([H])[C@]3([H])CCC4=C(O)C(CC[C@]4(C)[C@@]3([H])CC1)=O)C2=O	C19H26O3	302.408	Formestane
Medchem Express	HY-13808	COC1=CC2=C(NC3CCN(CC4CCCCC4)CC3)N=C(N5CCN(C(C)C)CCC5)N=C2C=C1OCCCN6CCCCC6	C37H61N7O2	635.926	UNC 0631
Medchem Express	HY-13812	NC1=CC2=C(NCCC3=CC=C(OC4=CC=CC=C4)C=C3)N=CN=C2C=C1	C22H20N4O	356.42	QNZ
Medchem Express	HY-13570	C[C@@]12[C@](C[C@H](C)[C@]2(O)C(CO)=O)([H])[C@]3([H])CCC4=CC(C=C[C@]4(C)[C@@]3(F)[C@@H](O)C1)=O	C22H29FO5	392.461	Betamethasone
Medchem Express	HY-50865	O=C1NC([C@H]2[C@@H](CN(C2)CC3=CC=CC=C3)C)=NC4=C1C=NN4C5CCOCC5	C22H27N5O2	393.482	PDE-9 inhibitor
Medchem Express	HY-11006	O=C1C=C(N2CCOCC2)OC3=C1C=CC=C3C4=CC=CC5=C4SC6=C5C=CC=C6	C25H19NO3S	413.488	KU-57788
Medchem Express	HY-10330	O=C(NCCN1CCCC1)C2=C(NC(/C=C3C(NC4=C\3C=C(C=C4)F)=O)=C2C)C	C22H25FN4O2	396.458	Toceranib
Medchem Express	HY-13962	O=C(NC1=CC=C(NC2=NC(N)=NC(C)=C2)C=C1)C3=CC=C(NC4=CC=NC5=CC=CC=C45)C=C3	C27H23N7O	461.518	SGI-1027
Medchem Express	HY-12365	O[C@H]([C@@H]1O)[C@@H](O[C@@H]1C(NC)=O)N2C3=NC(Cl)=NC(NCC4=CC(I)=CC=C4)=C3N=C2	C18H18ClIN6O4	544.731	2-Cl-IB-MECA
Medchem Express	HY-17365	NC(C(NC(C(NC(C(NC(CC1=CNC2=C1C=CC=C2)C3=O)=O)CC4=CC=CC=C4)=O)CSSCC(C(NC(CO)C(O)C)=O)NC(C(NC(C(CCCCN)N3)=O)C(O)C)=O)=O)CC5=CC=CC=C5.CC(O)=O	C51H70N10O12S2	1079.291	Octreotide (acetate)
Medchem Express	HY-B1227	O=C(O)C(C)C1=CC(NC2=C3C=C(Cl)C=C2)=C3C=C1	C15H12ClNO2	273.714	Carprofen
Medchem Express	HY-13248	O=C(C1=C2SC(CC3=C(NN=C3C)C)=C1C(N4C[C@@H](CO4)O)=O)N(C(N2CC(C)C)=O)C	C21H27N5O5S	461.535	AR-C155858
Medchem Express	HY-15249	OC(C1=CC=C(OCO2)C2=C1)(C3CCN(C(OC4=CC=C([N+]([O-])=O)C=C4)=O)CC3)C5=CC(OCO6)=C6C=C5	C27H24N2O9	520.487	JZL 184
Medchem Express	HY-16343C	O=C(O)/C=C\C(O)=O.CC(C)(C)C#C/C=C/CN(CC)CC1=CC(OCC2=CC(C3=CSC=C3)=CS2)=CC=C1	C31H35NO5S2	565.743	NB-598 (Maleate)
Medchem Express	HY-14111	O=C(NC)C(C=N1)=CC=C1OC2=CC(CC3)=C(C=C2)CCN3C4CCC4	C21H25N3O2	351.442	GSK189254A
Medchem Express	HY-B0122	NS(OC[C@]1(OC(C)(C)O2)[C@@H]2[C@H](OC(C)(C)O3)[C@H]3CO1)(=O)=O	C12H21NO8S	339.362	Topiramate
Medchem Express	HY-15455	O=C(NC1=C(Cl)C=NC=C1Cl)C2=CC=C(OC(F)F)C(OCC3CC3)=C2	C17H14Cl2F2N2O3	403.208	Roflumilast
Medchem Express	HY-B0134	CC(C)C[C@@H](C(O)=O)NC([C@@H](O)[C@H](N)CC1=CC=CC=C1)=O	C16H24N2O4	308.373	Bestatin
Medchem Express	HY-10342	O=C(C(C1=CN(C)C2=C1C=CC=C2)=C3C4=CN(C5CCN(CC6=NC=CC=C6)CC5)C7=C4C=CC=C7)NC3=O	C32H29N5O2	515.605	Enzastaurin
Medchem Express	HY-13551	CS(=O)(NC1=CC=C(NC2=C(C=CC=C3)C3=NC4=CC=CC=C42)C(OC)=C1)=O	C21H19N3O3S	393.459	Amsacrine
Medchem Express	HY-15450C	O=C(NCC(N[C@H]1CN([C@@H]2CC[C@](C3=CC=C(OC)N=C3)(O)CC2)CC1)=O)C4=CC=CC(C(F)(F)F)=C4	C26H31F3N4O4	520.544	INCB 3284 enantiomer 1
Medchem Express	HY-B0188A	CN(CC1)CC2N1C3=CC=CC=C3CC4=CC=CC=C24.Cl	C18H21ClN2	300.826	Mianserin (hydrochloride)
Medchem Express	HY-10159	O=C(NC1=CC(C(F)(F)F)=CC(N2C=NC(C)=C2)=C1)C3=CC=C(C)C(NC4=NC=CC(C5=CC=CN=C5)=N4)=C3	C28H22F3N7O	529.516	Nilotinib

Medchem Express	HY-16114	COC1=CC(C(C2=C(C)OC3=C(O)C(OC)=CC=C23)=O)=CC(OC)=C1OC	C20H20O7	372.369	BNC105
Medchem Express	HY-100416A	O=C(O)CN(CCOC1=CC=C(C2=CC=C(OCO3)C3=C2)C=C1C(C)(C)C)C.Cl[H]	C22H28ClNO5	421.914	LY2365109 (hydrochloride)
Medchem Express	HY-50895	ClC1=C(C=CC(NC2=NC=NC3=C2C=C(C(OC)=C3)OCCCN4CCOCC4)=C1)F	C22H24ClFN4O3	446.902	Gefitinib
Medchem Express	HY-B0561	C[C@@]12[C@](OC3=O)(CC3)CC[C@@]1([H])[C@@]([C@@H](CC4=CC5=O)SC(C)=O)([H])[C@]([C@]4(CC5)C)([H])CC2	C24H32O4S	416.574	Spironolactone
Medchem Express	HY-10502	O=C1N(C2=C(C(C3=CC=CC(Cl)=C3)=C1)C=C(C=C2)[C@@](N)(C4=CN=CN4C)C5=CC=C(C=C5)Cl)C	C27H22Cl2N4O	489.396	Tipifarnib
Medchem Express	HY-13821	O=C([C@@H](NC([C@H]([C@@H](C)O)NC([C@@H](NC([C@H]([C@@H](C)CC)N(C)C(C)=O)=O)[C@@H](C)CC)=O)=O)CC(C)C)[C@@]1(C)CO	C28H50N4O7	554.719	Epoxomicin
Medchem Express	HY-10629	ClC1=C(CN2N=C(C(C(F)(F)F)=CC=C3)C3=C2C4=CC=C(F)C=C4)C=CC(F)=C1	C21H12ClF5N2	422.778	LXR-623
Medchem Express	HY-15640	S=C(N1CCCC2=CC(O)=C(O)C=C2C1)NCCC3=CC=C(Cl)C=C3	C19H21ClN2O2S	376.9	Capsazepine
Medchem Express	HY-50769	O=C(N1CCC[C@@H]([C@@H](OCCNC(OC)=O)C2=CC=CC(Cl)=C2)C1)NC[C@@H](NC)C[C@@H]3COCCC3.O=C(C(F)(F)F)O	C28H42ClF3N4O7	639.104	VTP-27999 (2,2,2-trifluoroacetate)
Medchem Express	HY-16040	O=C(O)CC1=CC=C(C2=CC=C(C3=C(NC(O[C@@H](C4=CC=CC=C4)C)=O)C(C)=NO3)C=C2)C=C1	C27H24N2O5	456.49	AM095 (free acid)
Medchem Express	HY-B0422	O=C(O)[C@@H](CC1=CC=CC=C1)NC([C@H]2CC[C@H](C(C)C)CC2)=O	C19H27NO3	317.423	Nateglinide
Medchem Express	HY-12057	FC1=CC=C(C(F)=C1C(C2=CNC3=NC=C(C=C32)C4=CC=C(C=C4)Cl)=O)NS(CCC)(=O)=O	C23H18ClF2N3O3S	489.922	Vemurafenib
Medchem Express	HY-70013	C[C@@]12C(C3=CN=CC=C3)=CC[C@]1([C@@]4(CC=C5[C@@](C)([C@]4(CC2)[H])CC[C@@H](C5)O)[H])[H]	C24H31NO	349.509	Abiraterone
Medchem Express	HY-B0101	FC1=CC=C(C(O)(CN2C=NC=N2)CN3C=NC=N3)C(F)=C1	C13H12F2N6O	306.271	Fluconazole
Medchem Express	HY-18008	O=C(C1=CC=CN=C1)NC2=CC(Cl)=CC3=C2NC4=C3C=CN=C4	C17H11ClN4O	322.748	PS-1145
Medchem Express	HY-15416	O=C1NC2=C(C=CC(Cl)=C2Cl)/C1=N/O	C8H4Cl2N2O2	231.036	NS309
Medchem Express	HY-13822	OC1=CC=C(NC2=NC(C3=CC=C(Cl)C=C3)=CS2)C=C1	C15H11ClN2OS	302.779	SKI II
Medchem Express	HY-19533	NC1=NC(N(CCC2=CC=CC=C2)N=C3)=C3C4=NC(C5=CC=CO5)=NN14	C18H15N7O	345.358	SCH 58261
Medchem Express	HY-15471	O=C(OCC)NC1=CC=C(NCC2=CC=C(F)C=C2)C=C1N	C16H18FN3O2	303.331	Retigabine
Medchem Express	HY-10423	O=C([C@H]1CC[C@H](C2=NC(C(N3)=CC4=C3C(OC)=CC=C4)=C5C(N)=NC=NN52)CC1)O	C21H22N6O3	406.438	OSI-027
Medchem Express	HY-B0774	CC(C1=O)=C(C(C(C)=C1C)=O)C(C2=CC=CC=C2)CCCCCC(O)=O	C22H26O4	354.44	Seratrodast
Medchem Express	HY-13406	C[N+](C)(CC1=CC=C(NC(C2=CC3=CC(C4=CC=C(C)C=C4)=CC=C3CCC2)=O)C=C1)C5CCOCC5.[Cl-]	C33H39ClN2O2	531.128	TAK-779
Medchem Express	HY-15476	O=C(C1=NC=CC=C1)NC2=CC=C(S(=O)(NC3=CC=CC=C3Cl)=O)C(Cl)=C2	C18H13Cl2N3O3S	422.285	VU 0364439
Medchem Express	HY-15472	N#CC1=CC(CN2CCC(NC3=C(C=C(Cl)S4)C4=NC=N3)CC2)=CC=C1F	C19H17ClFN5S	401.888	PRX-08066
Medchem Express	HY-76859	O=S(C1=CC(F)=CC2=C1N(CC(C=C3)=CC=C3Cl)C4=C2CCC4CC(O)=O)(C)=O	C21H19ClFNO4S	435.896	4-[(4-chlorophenyl)methyl]-7-fluoro-1,2,3,4-tetrahydro-5-(methylsulfonyl)-Cyclopent[b]indole-3-acetic acid
Medchem Express	HY-16508	O=C1CCC2=C3[C@@H](C4=CC=C(N(C)C)C=C4)C[C@@]5(C)[C@](CC[C@]5(OC(C)=O)C(C)=O)([H])[C@]3([H])CCC2=C1	C30H37NO4	475.619	Ulipristal (acetate)
Medchem Express	HY-B0259	O=C(NN1C(C)CC2=C1C=CC=C2)C3=CC=C(Cl)C(S(=O)(N)=O)=C3	C16H16ClN3O3S	365.835	Indapamide
Medchem Express	HY-N0156	C[C@]([C@@]1([H])[C@]2(CC[C@@H]3O)C)(CC[C@@]2([H])C3(C)C)[C@@](C4=CC1)(CC[C@]5(C(O)=O)[C@@]4([H])CC(C)(C)CC5)C	C30H48O3	456.7	Oleanolic Acid
Medchem Express	HY-15685	FC1=CN=CC2=C1C(S(N3CCCNC[C@@H]3C)(=O)=O)=CC=C2.[H]Cl.O.O	C15H23ClFN3O4S	395.877	K-115
Medchem Express	HY-15676	O=C(O)C1=CC(OC)=C(NC([C@@H]2[C@@H](C3=C(F)C(Cl)=CC=C3)[C@](C4=CC=C(Cl)C=C4F)(C#N)[C@H](CC(C)(C)C)N2)=O)C=C1	C31H29Cl2F2N3O4	616.482	RG7388
Medchem Express	HY-19957	O=C(C1=C(N)N(C2=CC=C3N=C(C)NC3=C2)N=C1)C(N4)=CC5=C4C=CC=C5	C20H16N6O	356.381	CH5183284
Medchem Express	HY-70068	O=C(NC1=CC(C)=NC2=C(C=C(C=C21)F)F)NC3=CC=C(C=C3)N(C)C	C19H18F2N4O	356.369	SB-408124

Medchem Express	HY-B0129	CC(C)(O/N=C(C1=CSC(N)=N1)\C(N[C@H]2[C@H](C)N(S(=O)(O)=O)C2=O)=O)C(O)=O	C13H17N5O8S2	435.433	Aztreonam
Medchem Express	HY-17502	CCC(C)(C)C(O[C@H]1C[C@@H](C)C=C2C=C[C@H](C)[C@H](CC[C@@H]3C[C@@H](O)CC(O3)=O)[C@@]12[H])=O	C25H38O5	418.566	Simvastatin
Medchem Express	HY-17044	O=C1N(C2=CC=CC=C2)C([C@@H](NC3=C4N=CNC4=NC=N3)C)=CC5=C1C(Cl)=CC=C5	C22H17ClN6O	416.863	Duvelisib
Medchem Express	HY-B0723	ClCC/C(C1=CC=CC=C1)=C(C2=CC=C(OCCO)C=C2)\C3=CC=CC=C3	C24H23ClO2	378.891	Ospemifene
Medchem Express	HY-15777	O=C(N(C)C)C(N1C2CCCC2)=CC(C1=N3)=CN=C3NC(N=C4)=CC=C4N5CCNCC5	C23H30N8O	434.537	LEE011
Medchem Express	HY-10259A	FC(F)(C(O)=O)F.FC(F)(C(O)=O)F.O=C([C@@H]1CC2=C(N=CN2CC3=CC=C(N(C)C)C(C)=C3)CN1C(C(C4=CC=CC=C4)C5=CC=CC=C5)=O)O	C35H34F6N4O7	736.657	PD 123319 (ditrifluoroacetate)
Medchem Express	HY-14151	ClC1=C(N)C(CCO2)=C2C(C(NC3CCN(CCCOC)CC3)=O)=C1	C18H26ClN3O3	367.87	Prucalopride
Medchem Express	HY-10681	CN(C1CCN(C(C2=CC=C(NC(NC3=CC=C(C4=NC(N5CCOCC5)=NC(N6CCOCC6)=N4)C=C3)=O)C=C2)=O)CC1)C	C32H41N9O4	615.726	PKI-587
Medchem Express	HY-18628	O=C(O)CSC1=CC(NS(=O)(C2=CC=C(Br)C=C2)=O)=C3C=CC=CC3=C1O	C18H14BrNO5S2	468.342	UMI-77
Medchem Express	HY-15891	O=C1[C@@H](C(C)C)[C@@](N(C(/C=C/CN2CCCCC2)=O)CC3)([H])[C@]3([H])N1S(=O)(C)=O	C19H31N3O4S	397.532	GW311616
Medchem Express	HY-10595	O=C(NC1=NC=CS1)[C@H](CC2CCCC2)C3=CC=C(S(=O)(C)=O)C=C3	C18H22N2O3S2	378.509	Ro 28-1675
Medchem Express	HY-50875	N=C(C1=CC2=CC=C(OC(C3=CC=CS3)=O)C=C2S1)N.O=S(O)(C)=O	C15H14N2O5S3	398.477	BCX 1470 (methanesulfonate)
Medchem Express	HY-13519	ClC1=CC=CC=C1C(N2N=CC=C2)(C3=CC=CC=C3)C4=CC=CC=C4	C22H17ClN2	344.837	TRAM-34
Medchem Express	HY-15589	O=C(O)CCC1=CC=C(NCC2=CC=CC(OC3=CC=CC=C3)=C2)C=C1	C22H21NO3	347.407	GW9508
Medchem Express	HY-15448	O=C(C1(C2=CC=C(OC(F)(F)O3)C3=C2)CC1)NC4=CC5=C(N(C[C@@H](O)CO)C(C(C)(C)CO)=C5)C=C4F	C26H27F3N2O6	520.498	VX-661
Medchem Express	HY-15488	ClC1=C(Cl)C(C2=NN=NN2CC3=CC=CN=C3)=CC=C1	C13H9Cl2N5	306.15	A 438079
Medchem Express	HY-12061	C[C@@H]1O[C@@H](CN(CC(NC2=CC3=C(C=C2)SC4=C(C3)C=CC=C4C5=CC(C=C(O5)N6CCOCC6)=O)=O)C1)C	C30H33N3O5S	547.665	KU-60019
Medchem Express	HY-15342	O=C(O)CN1C(C)=C(CC2=NC3=CC=CC=C3C=C2)C4=C1C=CC(F)=C4	C21H17FN2O2	348.37	OC000459
Medchem Express	HY-79593	OC(COCCCCN(C(C)C)C1=NC(C2=CC=CC=C2)=C(C3=CC=CC=C3)N=C1)=O	C25H29N3O3	419.516	MRE-269
Medchem Express	HY-B0162A	O=C1N(CCCN(C[C@@H]2C3=CC(OC)=C(OC)C=C3C2)C)CCC4=CC(OC)=C(OC)C=C4C1.Cl	C27H37ClN2O5	505.046	Ivabradine (hydrochloride)
Medchem Express	HY-14462	O=C(CC(C1=CC=CC=C1)C2=CC=CC=C2)N3CCN(C(C4=CC=CC=C4)C5=CC=CC=C5)CC3	C32H32N2O	460.609	NP118809
Medchem Express	HY-12238	O=C(NC1=C2N=CC=CC2=CC=C1)C3=CC=C(N(C([C@]4([H])[C@](C5)([H])C=C[C@]5([H])[C@]64[H])=O)C6=O)C=C3	C25H19N3O3	409.437	IWR-1
Medchem Express	HY-B0284	O=C(C1=C(C)NC(C)=C(C(OC)=O)C1C2=CC=CC=C2[N+]([O-])=O)OC	C17H18N2O6	346.335	Nifedipine
Medchem Express	HY-107383	O=C1C2=C(NC(N)=N1)NCC(C(O)C(O)C)N2	C9H15N5O3	241.247	Tetrahydrobiopterin
Medchem Express	HY-B0130A	O=C([C@H]1N(C([C@@H](N[C@H](C(OCC)=O)CCC)C)=O)[C@@]2([H])CCCC[C@@]2([H])C1)O.CC(C)(C)N	C23H43N3O5	441.605	Perindopril (erbumine)
Medchem Express	HY-W011846	C1(C2=NC3=CC=CC=C3C=C2)=NC4=CC=CC=C4C=C1	C18H12N2	256.308	2,2'-Biquinoline
Medchem Express	HY-103350	O=C(N1[C@@H](CCC1)C(O)=O)[C@@]([C@@H](C)CC)([H])NC([C@@H]2[C@@H](C(NCCC)=O)O2)=O	C18H29N3O6	383.439	CA-074
Medchem Express	HY-B0012	OC(P(O)(O)=O)(P(O)(O)=O)CCN	C3H11NO7P2	235.07	Pamidronic acid
Medchem Express	HY-107381	OC(CCC/C=C\C[C@@H]1[C@H]([C@H](O)C[C@@H]1O)/C=C/[C@@H](O)COC2=CC(Cl)=CC=C2)=O	C22H29ClO6	424.915	(+)-Cloprostenol
Medchem Express	HY-107373	OC([C@@H](N)CCl)=O	C3H6ClNO2	123.538	β-Chloro-L-alanine
Medchem Express	HY-103309	O=C(NC[C@@H]1[C@@]2([H])CN(CCC(C)(C)C)C[C@@]12[H])C3=CC(Cl)=CC(Cl)=C3	C19H26Cl2N2O	369.329	ML218
Medchem Express	HY-18935A	CC(NCCN1C2=C(C3=C1C=CC(C(C)=O)=C3)C=C(C(C)=O)C=C2)C.[H]Cl	C21H25ClN2O2	372.89	CBL0137 HCl
Medchem Express	HY-101479	NC1=NC=C(CC2=C3C=CC(C4CC4)OC3=C(OC)C(OC)=C2)C(N)=N1	C19H22N4O3	354.4	Iclaprim
Medchem Express	HY-100309	O=C1[C@](O)(CC)C2=C(CO1)C(N3CC4=CC5=C(N)C=CC=C5N=C4C3=C2)=O	C20H17N3O4	363.367	9-Aminocamptothecin

Medchem Express	HY-11103	O=S([N-]C1=C(C(C)=NO1)Cl)(C2=C(SC=C2)C(CC3=C(C=C4OCOC4=C3)C)=O)=O.[Na+]	C18H14ClN2NaO6S2	476.886	Sitaxsentan (sodium)
Medchem Express	HY-101105A	O=C(C1=CC=C(C2=CC=C(C3=NOC(C)=N3)C=C2C)C=C1)N4CCC5=C4C=C6C(OCC67CCN(C)CC7)=C5.Cl	C32H33ClN4O3	557.082	SB-224289 hydrochloride
Medchem Express	HY-19334	O=S(C1=CC=C(NC(NCCCCCC)=O)C=C1)(NC2=CC=C(CCNC[C@H](O)COC3=CC=C(O)C=C3)C=C2)=O	C30H40N4O6S	584.727	L755507
Medchem Express	HY-107371	O=CC1=C(C(OC)=CC=C1Br)O	C8H7BrO3	231.043	NSC95682
Medchem Express	HY-22024	O=C1C=C(C2=CC=CC=C2)OC3=CC=CC(O)=C13	C15H10O3	238.238	5-Hydroxyflavone
Medchem Express	HY-13455	NCCCSC1=C(C=C(OC)C=C2)C2=NC3=CC=C(OC)C=C31	C18H20N2O2S	328.429	LDN-192960
Medchem Express	HY-107384	O[C@H]1CCN(C1)C[C@H](C2=CC=CC=C2)N(C)C(C(C3=CC=CC=C3)C4=CC=CC=C4)=O	C27H30N2O2	414.539	Asimadoline
Medchem Express	HY-108314A	NC(NCCCCCCCN)=N.O=S(O)(O)=O	C8H22N4O4S	270.348	GC7 Sulfate
Medchem Express	HY-101502A	N=C(N)NCCC[C@@H](C(O)=O)NC(COCC(C1=CC=CC=C1)C2=CC=CC=C2)=O.OC(C(F)(F)F)=O	C24H29F3N4O6	526.51	SB290157 (trifluoroacetate)
Medchem Express	HY-12336	O=C(N1CCN(C(C2=CC=C(OC)C=C2)=O)CC1)/C=C/C3=CC=C(Br)C=C3	C21H21BrN2O3	429.307	NIBR189
Medchem Express	HY-10992	O=C(N[C@@H]1CNCCC1)C2=C(C=C(S2)C3=CC=CC(F)=C3)NC(N)=O	C17H19FN4O2S	362.422	AZD-7762
Medchem Express	HY-N0170	OCC1=CNC2=C1C=CC=C2	C9H9NO	147.174	Indole-3-carbinol
Medchem Express	HY-100546A	C1(N[C@H]2[C@H](C3=CC=CC=C3)C2)CCNCC1.Cl.Cl	C14H22Cl2N2	289.244	GSK-LSD1 Dihydrochloride
Medchem Express	HY-18260	CC(C1=CC=C(O)C=C1)(C2=CC=C(O)C=C2)C	C15H16O2	228.286	Bisphenol A
Medchem Express	HY-14167	ClC1=CC(Cl)=C(NC2=NC=C(C(NCC3CCOCC3)=O)C(C(F)(F)F)=N2)C=C1	C18H17Cl2F3N4O2	449.254	GW842166X
Medchem Express	HY-107385	C[C@@]12[C@](CC[C@@H]2C(NC(C)(C)C)=O)([H])[C@@]3([H])[C@]([C@@]4(C(C=C(C(O)=O)CC4)=CC3)C)([H])CC1	C25H37NO3	399.566	Epristeride
Medchem Express	HY-22026	CC(C)(C)OC(N(CC1)CCC1OC2CCNCC2)=O	C15H28N2O3	284.394	4-[(Piperidin-4-yl)oxy]piperidine-1-carboxylic acid tert-butyl ester
Medchem Express	HY-18299	O=C(O)C1=CC=C(NC2=C3N=CN(C(C)C)C3=NC(N[C@@H](CO)C(C)C)=N2)C=C1Cl	C20H25ClN6O3	432.9	Purvalanol
Medchem Express	HY-N2143	O=C([C@]1([H])CC2=C([C@H](/C=C(C)\C)N13)NC4=C2C=CC(OC)=C4)N5[C@](CCC5)([H])C3=O	C22H25N3O3	379.45	Fumitremorgin C
Medchem Express	HY-10858	O=S(C1=CC(S(=O)(C2=CC=CC=C2)=O)=CC=C1C(F)(F)F)(NC3CCNCC3)=O	C18H19F3N2O4S2	448.48	WAY 316606
Medchem Express	HY-50878	ClC1=C(F)C=CC(Cl)=C1[C@H](OC2=CC(C3=CN(N=C3)C4CCNCC4)=CN=C2N)C	C21H22Cl2FN5O	450.337	Crizotinib
Medchem Express	HY-107379	O=S(N1CC2=CC=CC=C2CC1)(C3=CC(C(O)=O)=CC=C3)=O	C16H15NO4S	317.36	AKR1C3-IN-1
Medchem Express	HY-N0017	OC1=C(OC)C(O)=C([C@@]2([H])[C@@](OC3=O)([H])[C@@H](O)[C@H](O)[C@@H](CO)O2)C3=C1	C14H16O9	328.272	Bergenin
Medchem Express	HY-N0085	O=C1C=CC2=CC(OC)=C(OC)C(OC)=C2O1	C12H12O5	236.221	Dimethylfraxetin
Medchem Express	HY-15849	N[C@@H](CC1=CC=C(C2=NC(N)=NC(OC(C3=CC=C(C4=CC=CC(F)=C4)C=C3)C(F)(F)F)=C2)C=C1)C(O)=O	C27H22F4N4O3	526.482	LP-533401
Medchem Express	HY-100558	C[C@H]([C@](O[C@@H]1C(C)C)(C[C@@H](O)[C@@H]1C)O)[C@H](O)[C@@H]([C@](OC(/C(OC)=C/C(C)=C\[C@@H](C)[C@@H](O)[C@@H](C)C2)=O)([H])[C@H](/C=C/C=C2\C)OC)C	C35H58O9	622.83	Bafilomycin A1
Medchem Express	HY-108318	NC1=C2C(N(C3CCCC3)C=C2C4=CC=C(OC5=CC=CC=C5)C=C4)=NC=N1	C23H22N4O	370.447	RK-24466
Medchem Express	HY-A0016	CCCCC1=C(C2=C(C=CC(NS(C)(=O)=O)=C2)O1)C(C3=CC=C(C=C3)OCCCN(CCCC)CCCC)=O	C31H44N2O5S	556.76	Dronedarone
Medchem Express	HY-103254	O=C1C=C(CSC2=NC=CC=N2)OC=C1OC(C3=CC=C([N+]([O-])=O)C=C3)=O	C17H11N3O6S	385.35	ML221
Medchem Express	HY-17453	O=C(C1=CC=C2C=CC=CC2=C1O)O.OC3=C(CO)C=C(C(CNCCCCCCOCCCCC4=CC=CC=C4)O)C=C3	C36H45NO7	603.745	Salmeterol (xinafoate)
Medchem Express	HY-B0192	O=C(C1OCCC1)NCCCN(C2=NC(N)=C3C=C(OC)C(OC)=CC3=N2)C	C19H27N5O4	389.449	Alfuzosin
Medchem Express	HY-B2159	C[C@@]12[C@@](C)(O)CC[C@@]1([H])[C@]3([H])CCC4=CC(CC[C@]4(C)[C@@]3(F)[C@@H](O)C2)=O	C20H29FO3	336.441	Fluoxymesterone

Medchem Express	HY-103392	CC(C)(C)C(O)/C=C/C1=CC=C(OCO2)C2=C1	C14H18O3	234.291	Stiripentol
Medchem Express	HY-103609	C12=CC=C3C=CC=C4C=CC(C2=C34)=CC=C1	C16H10	202.251	Pyrene
Medchem Express	HY-W008613	O=C(O)C1=C(O)C(CC2=C3C=CC=CC3=CC(C(O)=O)=C2O)=C4C=CC=CC4=C1	C23H16O6	388.375	4,4'-Methylenebis(3-hydroxy-2-naphthoic acid)
Medchem Express	HY-59047	O=C1N=CC(OC2=CC=CC(C)=C2)=CN1	C11H10N2O2	202.209	Tolimidone
Medchem Express	HY-100737	COC1=CC=C(N2CCN(C(C3=CC4=C(C(C=CC=C5)=C5S(C4)(=O)=O)S3)=O)CC2)C=C1	C23H22N2O4S2	454.562	ML349
Medchem Express	HY-12956A	CCCCC[C@H](O)/C=C/[C@H]1[C@H](O)C[C@H](O)[C@@H]1C/C=C\CCCC(O)=O.OCC(CO)(N)CO	C24H45NO8	475.62	Dinoprost (tromethamine salt)
Medchem Express	HY-108319	O=C(OCC1=CC=C([N+]([O-])=O)C=C1)NCCCC[C@H](N)C(N2CCCC2)=O	C18H26N4O5	378.42	DPP-IV-IN-2
Medchem Express	HY-10797	ClC1=CC=C(OC2=CC=C(F)C=C2)C(C(N[C@H](C3=CC=C(C(O)=O)C=C3)C)=O)=C1	C22H17ClFNO4	413.826	CJ-42794
Medchem Express	HY-10997A	C=CC(N1CC(N2N=C(C3=CC=C(OC4=CC=CC=C4)C=C3)C5=C(N)N=CN=C52)CCC1)=O	C25H24N6O2	440.497	PCI-32765 (Racemate)
Medchem Express	HY-22023	C1(CC2=CC=CC=C2)=CC=NC=C1	C12H11N	169.222	4-Benzylpyridine
Medchem Express	HY-107369	CCCCC1=C(C=C(O)C=C1)O	C10H14O2	166.217	4-Butylresorcinol
Medchem Express	HY-107368	O=C(C1C(CCCC1=O)=O)C2=C(C=C(S(C)(=O)=O)C=C2)Cl	C14H13ClO5S	328.768	Sulcotrione
Medchem Express	HY-100578	O=C(NC1=C2C=CC=NC2=CC=C1)/C(C#N)=C/C3=CC=C(C4=CC(Cl)=CC=C4Cl)O3	C23H13Cl2N3O2	434.274	AGK2
Medchem Express	HY-W016403	O=C1C(C2=C3C1=CC=CC3=CC=C2)=O	C12H6O2	182.178	Acenaphthylene-1,2-dione
Medchem Express	HY-12170	O=C([C@@H]1N(S(=O)(C2=CC=C(OC3=CC=NC=C3)C=C2)=O)CCSC1(C)C)NO	C18H21N3O5S2	423.506	Prinomastat
Medchem Express	HY-14861A	O=C(NC1=NC(C(C2CCN(C)CC2)=O)=CC=C1)C3=C(F)C=C(F)C=C3F.[H]Cl	C19H19ClF3N3O2	413.82	Lasmiditan (hydrochloride)
Medchem Express	HY-13861	CC(C)(SC1=CC=C(CCN(C(NC2CCCCC2)=O)CCCCC3CCCCC3)C=C1)C(O)=O	C29H46N2O3S	502.75	GW7647
Medchem Express	HY-90009A	O=C([C@@]1([H])CC2=C([C@@H](C3=CC=C(OCO4)C4=C3)N15)NC6=C2C=CC=C6)N(C)CC5=O	C22H19N3O4	389.404	Tadalafil
Medchem Express	HY-12222	C[C@@]([C@]1([H])[C@@H](CC)[C@H]2O)(CC[C@@H](O)C1)[C@]3([H])[C@]2([H])[C@@](CC[C@]4([H])[C@H](C)CCC(O)=O)([H])[C@]4(C)CC	C26H44O4	420.625	INT-747
Medchem Express	HY-108325	O=C(C1=C(C)C(C2=CC=C(C3=CC=CC=C3F)C=C2)=NC4=CC=C(F)C=C14)O	C23H15F2NO2	375.368	Brequinar
Medchem Express	HY-106441A	O=C(N1C[C@@H](F)[C@@H](CNC2=NC=CC=N2)CC1)OCC3=CC=C(C)C=C3	C19H23FN4O2	358.41	Rislenemdaz
Medchem Express	HY-B1751	C=C[C@H]1C[N@](CC[C@H]1C2)[C@@]2([H])[C@@H](O)C3=CC=NC4=CC=C(OC)C=C34	C20H24N2O2	324.417	Quinidine
Medchem Express	HY-100788	O=C(O)C(CP(O)(O)=O)CCC(O)=O	C6H11O7P	226.121	2-PMPA
Medchem Express	HY-108324	O=S(C1=CC(/C2=C\CCN3CCN(C)CC3)=C(SC4=C2C=CC=C4)C=C1)(N(C)C)=O	C23H29N3O2S2	443.625	(Z)-Thiothixene
Medchem Express	HY-Y0355	ClC1=NC(Cl)=NC(Cl)=N1	C3Cl3N3	184.411	Cyanuric chloride
Medchem Express	HY-50108	O=C(C1=CC=CC(/C=C/C2=CC=C(C(C=C2Cl)OCC3=C(ON=C3C4=C(Cl)C=CC=C4Cl)C(C)C)=C1)O	C28H22Cl3NO4	542.84	GW 4064
Medchem Express	HY-103107	O=C(C1=CC=C(F)C=C1)NC2=CC3=C(C=C2)NC=C3C4CCN(C)CC4	C21H22FN3O	351.42	LY334370
Medchem Express	HY-15245	CC1=NC2=C(C(O)=O)C=C(C=C2N1CC3=CC=CC(C(F)(F)F)=C3C)N4CCOCC4	C22H22F3N3O3	433.424	GSK2636771
Medchem Express	HY-103610	O=C(C1=CC=C(OC)C=C1)C(C2=CC=C(OC)C=C2)=O	C16H14O4	270.28	4,4'-Dimethoxybenzil
Medchem Express	HY-W012484	NCCC1=CC=CC=C1Br	C8H10BrN	200.079	2-Bromophenethylamine
Medchem Express	HY-20891	CCC(/C(C)=C/C1=CC=C(F)C=C1)=N\O	C12H14FNO	207.244	1-Penten-3-one, 1-(4-fluorophenyl)-2-methyl-, oxime
Medchem Express	HY-17385	CC1=CC=CC=C1O[C@@H](C2=CC=CC=C2)CCNC.[H]Cl	C17H22ClNO	291.816	Atomoxetine (hydrochloride)
Medchem Express	HY-17042A	O=C(O)COCCN1CCN(C(C2=CC=C(Cl)C=C2)C3=CC=CC=C3)CC1.Cl.Cl	C21H27Cl3N2O3	461.81	Cetirizine (dihydrochloride)

Medchem Express	HY-108315	NC1=C2C=CC=CC2=C3C(C4=CC=CC=C4C=C3)=C1	C18H13N	243.303	6-Aminochrysene
Medchem Express	HY-108317	O=C(N1N(C(OC)=O)[C@@](C2=CC=CC=C2)(C3CCCC3)C1=O)OC	C17H20N2O5	332.351	ABL127
Medchem Express	HY-B0800	NC(NCCN1CCCCCCC1)=N.O=S(O)(O)=O	C10H24N4O4S	296.39	Guanethidine (sulfate)
Medchem Express	HY-101932	C[C@]1([C@@H](C/C=C(C)/C)O1)[C@]([C@@H]2OC)([H])[C@]3(CC[C@H]2OC(NC(CCl)=O)=O)CO3	C19H28ClNO6	401.88	TNP-470
Medchem Express	HY-18234A	O=S(O)(O)=O.O=C(N[C@H](C(N[C@H](C([H])=O)CCCNC(N)=N)=O)CC(C)C)[C@@H](NC(C)=O)CC(C)C.O=C(N[C@H](C(N[C@H](C([H])=O)CCCNC(N)=N)=O)CC(C)C)[C@@H](NC(C)=O)CC(C)C	C40H78N12O12S	475.59	Leupeptin (hemisulfate)
Medchem Express	HY-10358	O=C1N2C(C3=CC(C4=CC=CC=C4)=C(N=C3C=C2)C5=CC=C(C6(N)CCC6)C=C5)=NN1.Cl.Cl	C25H23Cl2N5O	480.39	MK 2206 (dihydrochloride)
Medchem Express	HY-N0700	C/C=C/C1=C(OC)C=C(OC)C(OC)=C1	C12H16O3	208.25	alpha-Asarone
Medchem Express	HY-12355	O=C(C1CN(CC2=CC=C(/C(C)=N/OCC3=CC=C(C4CCCCC4)C(C(F)(F)F)=C3)C=C2CC)C1)O	C29H35F3N2O3	516.6	Siponimod
Medchem Express	HY-10029	O=C(N1C(C2=C(C=C(C=C2)OC)OC(C)C)=N[C@H]([C@H]1C3=CC=C(C=C3)Cl)C4=CC=C(C=C4)Cl)N5CC(NCC5)=O	C30H30Cl2N4O4	581.49	Nutlin (3a)
Medchem Express	HY-A0013	O=S(NC1=NC(C2=NC=CC=N2)=NC(OCCO)=C1OC3=CC=CC=C3OC)(C4=CC=C(C(C)(C)C)C=C4)=O	C27H29N5O6S	551.61	Bosentan
Medchem Express	HY-15187	O=C(N1[C@@](C2=CC=CC=C2)(CCCN)SC(C3=CC(F)=CC=C3F)=N1)N(OC)C	C20H22F2N4O2S	420.48	ARRY-520
Medchem Express	HY-76948	O=C(O[C@@H]1CNC(C2=CC=C(Cl)S2)=O)N(C1)C(C=C3)=CC=C3N4C(COCC4)=O	C19H18ClN3O5S	435.88	5-R-Rivaroxaban
Medchem Express	HY-13327	O=C([C@H]1CC[C@H](CN2CCC[C@H](N(CC3=CC(C(F)(F)F)=CC(C(F)(F)F)=C3)C4=NN(C)N=N4)C5=CC(C)=CC(C)=C52)CC1)O	C31H36F6N6O2	638.65	Evacetrapib
Medchem Express	HY-10001	C=C1[C@H](C[C@@H](C/C1=C/C=C2[C@]3([C@@](C)([C@H](CC3)[C@@H](/C=C/[C@H](C4CC4)O)C)CCC/2)[H])O)O	C27H40O3	412.6	Calcipotriol
Medchem Express	HY-B1017	O=C1C2=C(NC(C)=C2CC)CCC1CN3CCOCC3.[H]Cl	C16H25ClN2O2	312.83	Molindone (hydrochloride)
Medchem Express	HY-12424	CO[C@H]1[C@@H](N2C=NN=N2)CC[C@@H](C[C@H]([C@@](CC([C@@H](/C=C([C@H]([C@H](C([C@@H](C[C@@H]3C)C)=O)OC)O)\C)C)=O)([H])OC([C@@](CCCC4)([H])N4C(C([C@](O[C@]5([H])C[C@@H](/C(C)=C/C=C/C=C/3)OC)([C@@H](CC5)C)O)=O)=O)=O)C)C1	C52H79N5O12	966.21	Zotarolimus
Medchem Express	HY-17377	CC1(N2C[C@H](C)N([C@H](C3=CC=C(C(F)(F)F)C=C3)COC)CC2)CCN(C(C4=C(C)N=CN=C4C)=O)CC1.O=C(O)/C=C\C(O)=O	C32H42F3N5O6	649.7	Vicriviroc (maleate)
Medchem Express	HY-10801	O=C(CN1C(C=CC(C(O)=O)=C2)=C2C(C(C(C)C)=O)=C1)COC(C=C3)=CC=C3OC4=CC=CC=C4	C28H25NO6	471.5	CAY10650
Medchem Express	HY-11093	C[C@H]1CNCCC2=CC=C(Cl)C=C12	C11H14ClN	195.69	Lorcaserin
Medchem Express	HY-13511A	CC1=CN=CC(CN2CC/C(CC2)=C3C4=CC=C(Cl)C=C4CCC5=CC=CN=C5\3)=C1.O=C(O)/C=C/C(O)=O	C30H30ClN3O4	532.03	Rupatadine (Fumarate)
Medchem Express	HY-15478	C1(CNC2=NC=CC=C2)=CC=C(CNC3=NC=CC=C3)C=C1	C18H18N4	290.36	WZ811
Medchem Express	HY-50844	CC(N)(C(N[C@@H](C(N1CCC2(CN(C3=C2C=CC=C3)S(=O)(C)=O)CC1)=O)COCC4=CC=CC=C4)=O)C.CS(=O)(O)=O	C28H40N4O8S2	624.77	Ibutamoren (Mesylate)
Medchem Express	HY-10997	C=CC(N1C[C@H](N2N=C(C3=CC=C(OC4=CC=CC=C4)C=C3)C5=C(N)N=CN=C52)CCC1)=O	C25H24N6O2	440.5	PCI-32765
Medchem Express	HY-11000	O=C(C(C(C1=CC=C(C(F)(F)F)C=C1)C2)=C(C)NC2=O)NC3=CC4=C(NN=C4)C=C3F	C21H16F4N4O2	432.37	GSK429286A
Medchem Express	HY-A0096	O=C(O)CCC/C=C1C[C@@]2([H])C[C@@H](O)[C@H](/C=C/[C@@H](O)C(C)CC#CC)[C@@]2([H])C\1	C22H32O4	360.49	Iloprost
Medchem Express	HY-19329	O=C1N(CC2=CC=C(F)C=C2)C(S/C1=C\C3=CC=C(OCC4=CC(B(O)O)=CC=C4)C=C3)=O	C24H19BFNO5S	463.29	HA130
Medchem Express	HY-12684	O=C(C1=CC=NN1C)NC2=CC=C(/N=N/C3=CC=CC=C3C)C=C2C	C19H19N5O	333.39	CH-223191
Medchem Express	HY-12299	O=C(OC1=C(C)C=CC=C1C)N(C2=CC=C(OC)C=C2OC)C3=NC(NC4=CC=C(N5CCN(C)CC5)C=C4)=NC=C3	C32H36N6O4	568.67	WH-4-023
Medchem Express	HY-10460	CS(=O)(N(C)C1=CC=CC=C1CNC2=NC(NC3=CC4=C(C=C3)NC(C4)=O)=NC=C2C(F)(F)F)=O	C22H21F3N6O3S	506.5	PF-431396
Medchem Express	HY-15968	C12=NC=CN1C=C(C3=CC4=C(C=C3)C=NN4)N=C2NC5=CC=C(N6CCOCC6)C=C5	C23H21N7O	411.46	GS-9973
Medchem Express	HY-13343	O=C(NC1=CC=C(C)C(NC(C2=CC=C(O)C=C2)=O)=C1)C3=CC=CC(N(C)C)=C3	C23H23N3O3	389.45	ZM 336372
Medchem Express	HY-15196	ClC(C=C(NC1=NC=NC2=C1N(CCNC(CC(C)(C)O)=O)C=C2)C=C3)=C3OC4=CC=CC(C(F)(F)F)=C4	C26H25ClF3N5O3	547.96	TAK-285

Medchem Express	HY-13955	O=C(C1=CC=CC=C1C2=CC=C(CN3C4=CC(C5=NC6=CC=CC=C6N5C)=CC(C)=C4N=C3CCC)C=C2)O	C33H30N4O2	514.62	Telmisartan
Medchem Express	HY-13470	O=C(C1=CC(C2=CC=C(N3CCNCC3)N=C2)=CC4=C1C(C)=CN4[C@@H](C)CC)NCC5=C(C)C=C(C)NC5=O	C31H38N6O2	526.67	GSK126
Medchem Express	HY-18728	O=C(NC1=CC=CN=C1)C2=CC=C(CNS(=O)(C3=CC=C(C(C)(C)C)C=C3)=O)C=C2	C23H25N3O3S	423.53	STF-31
Medchem Express	HY-50868	O=C(C1=CC=C(C(C(F)(F)F)=C1)CN2C[C@@H](N(C)C)CC2)NC3=CC=C(C)C(NC4=NC=CC(C5=CN=CN=C5)=N4)=C3	C30H31F3N8O	576.62	Bafetinib
Medchem Express	HY-10284	O=C1N(C2=C(C(N1CC3=NC(C)=C4C=CC=CC4=N3)=O)N(CC#CC)C(N5CCC[C@@H](N)C5)=N2)C	C25H28N8O2	472.54	Linagliptin
Medchem Express	HY-15800A	CS(=O)(NC1=CC=CC=C1NC2=NC(NC3=CC=C(N4CCOCC4)C=C3OC)=NC=C2F)=O	C22H25FN6O4S	488.54	CZC-25146
Medchem Express	HY-10344	O=C1N2C(C([C@H](NC3=CC=CC=C3C(O)=O)C)=CC(C)=C2)=NC(N4CCOCC4)=C1	C22H24N4O4	408.45	AZD 6482
Medchem Express	HY-10198	O=C1C(C(NC2=CC=CC(C(N(C)C)=O)=C2O)=C1N[C@H](CC)C3=CC=C(O3)C)=O	C21H23N3O5	397.42	SCH 527123
Medchem Express	HY-10547	O=C(CN)NC1=CC=C(C=C1)N2N=C(C=C2C3=CC=C4C5=CC=CC=C5C=CC4=C3)C(F)(F)F	C26H19F3N4O	460.45	OSU-03012
Medchem Express	HY-20019	O=C(O)COC(C=C1)=CC=C1OCCCOC2=CC=C(C(C)=O)C(O)=C2CCC	C22H26O7	402.44	L-165041
Medchem Express	HY-10110	O=C1N(C(CN2C3=C(C(N)=NC=N3)N=C2)=NC4=CC=CC(C)=C14)C5=C(C)C=CC=C5	C22H19N7O	397.43	IC-87114
Medchem Express	HY-106150B	CS(=O)(C1=C(N2C=CC=C2)C=C(C)C(C(NC(N)=N)=O)=C1)=O.[H]Cl	C14H17ClN4O3S	356.83	Eniporide hydrochloride
Medchem Express	HY-15601	O=C1NC2=C(N)N=C(OCCCC)N=C2N(CC3=CC=CC(CN4CCCC4)=C3)C1	C22H30N6O2	410.51	GS-9620
Medchem Express	HY-14289	CC1=C(CSCC/N=C(NC)/NC#N)NC=N1	C10H16N6S	252.34	Cimetidine
Medchem Express	HY-12043	CC(C)(C1=NC(C2=CC=CC(C)=N2)=C(C3=CC=C4N=CC=NC4=C3)N1)C	C21H21N5	343.42	SB 525334
Medchem Express	HY-13034	CC1=NNC(NC2=NN3C(C(CN4CCOCC4)=C2)=NC(C)=C3CC5=CC=C(C=C5F)Cl)=C1	C23H25ClFN7O	469.94	LY2784544
Medchem Express	HY-50683	FC(F)(C1=NN=C2C=CC(C3=CN(C)N=C3)=NN21)C4=CC5=C(N=CC=C5)C=C4	C19H13F2N7	377.35	JNJ-38877605
Medchem Express	HY-10505	O=C(C1=CC=C2C=C([C@@]3(O)CCN4C=NC=C43)C=CC2=C1)NC	C18H17N3O2	307.35	Orteronel
Medchem Express	HY-B0006	OC(CNCCOC1=CC=CC=C1OC)COC2=CC=CC(N3)=C2C4=C3C=CC=C4	C24H26N2O4	406.47	Carvedilol
Medchem Express	HY-17372	O=C1OCC(C2=CC=C(S(=O)(C)=O)C=C2)=C1C3=CC=CC=C3	C17H14O4S	314.36	Rofecoxib
Medchem Express	HY-15466	NC1=NC=C(C2=C3C(N(S(=O)(C)=O)CC3)=NC(N4CCOCC4)=N2)C=N1	C15H19N7O3S	377.42	CH5132799
Medchem Express	HY-14751	C[C@@](C1=CC(C(F)(F)F)=CC(C(F)(F)F)=C1)([H])OC[C@@]2(C3=CC=CC=C3)CC[C@@](CCC4=O)(N4)CN2	C25H26F6N2O2	500.48	Rolapitant
Medchem Express	HY-15894	CCCC[C@H](C([O-])=O)NC([C@@H](CC1=CN(C(OC)=O)C2=CC=CC=C12)NC([C@H](CC(C)(C)C)NC(N3[C@H](C)CCC[C@@H]3C)=O)=O)=O.[Na+]	C34H50N5NaO7	663.78	BQ-788 (sodium salt)
Medchem Express	HY-70044	O=C(NC1=CC=C(C2=NN(C=C2C3=C4C(NC(C5=CC=CC(CN(C)C)=C5)=C4)=NC=C3)CC)C=C1)N(C)C	C30H33N7O	507.63	GSK-1070916
Medchem Express	HY-15227	NC1=C2C(N([C@H]3[C@H](O)[C@H](O)[C@@H](CN(C(C)C)CCCNC(NC4=CC=C(C(C)(C)C)C=C4)=O)O3)C=C2)=NC=N1	C28H41N7O4	539.67	EPZ004777
Medchem Express	HY-50098	CN(N=C1C2=CC=C(OCC3=NC4=CC=CC=C4C=C3)C=C2)C=C1C5=CC=NC=C5	C25H20N4O	392.45	PF-2545920
Medchem Express	HY-51424	O=S(NC1=CC=C(C(C(C2=CNC3=NC=C(C=C23)Cl)=O)=C1F)F)(CCC)=O	C17H14ClF2N3O3S	413.83	PLX-4720
Medchem Express	HY-15743	O=C(NC1=CC=C(O)C=C1)C[C@H]2C3=NN=C(C)N3C4=C(C(C)=C(C)S4)C(C5=CC=C(Cl)C=C5)=N2	C25H22ClN5O2S	491.99	OTX-015
Medchem Express	HY-10012	O=S(C1=CC=C(NC2=NC(C3=CN=C(N3C(C)C)C)=CC=N2)C=C1)(C)=O	C18H21N5O2S	371.46	AZD-5438
Medchem Express	HY-15557	O=S(C1(C2=CC(N3[C@H](C)COCC3)=NC(C4=CC=CC5=C4C=CN5)=N2)CC1)(C)=O	C21H24N4O3S	412.51	AZ20
Medchem Express	HY-12152	O=C(NC1=CC(Cl)=C(C=C1OC)OC)NC2=NOC(C)=C2	C13H14ClN3O4	311.72	PNU-120596
Medchem Express	HY-14197A	C#CCN(CCCOC1=CC=C(Cl)C=C1Cl)C.[H]Cl	C13H16Cl3NO	308.63	Clorgiline (hydrochloride)
Medchem Express	HY-19975	O=S(C1=CC=C(Cl)C=C1Cl)(N(C(C)C)CCNC(C)C)=O	C14H22Cl2N2O2S	353.31	RN-1734

Medchem Express	HY-N0437	CC([C@H]1CC[C@@]2([H])[C@]3([H])CCC4=CC(CC[C@]4(C)[C@@]3([H])CC[C@]12C)=O)=O	C21H30O2	314.46	Progesterone
Medchem Express	HY-B0216	OC1=CC=C2C(CC[C@]3([H])[C@]2([H])CC[C@@]4(C)[C@@]3([H])CC[C@]4(C#C)O)=C1	C20H24O2	296.4	Ethynyl Estradiol
Medchem Express	HY-B0696A	O=C([C@H]1CN(CC/C=C(C2=C(C)C=CS2)/C3=C(C)C=CS3)CCC1)O.[H]Cl	C20H26ClNO2S2	412.01	Tiagabine (hydrochloride)
Medchem Express	HY-16699	O=C(NO)C1=CC=C(CN(CCCC)C(NC2=CC=CC=C2)=O)C=C1	C19H23N3O3	341.4	Nexturastat A
Medchem Express	HY-B0428	O=C(O)/C=C/C1=CC=C(CN2C=CN=C2)C=C1	C13H12N2O2	228.25	Ozagrel
Medchem Express	HY-14137	ClC1=CC=C(C2=C(C)C(C(NN3CCCCC3)=O)=NN2C4=CC=C(Cl)C=C4Cl)C=C1.Cl	C22H22Cl4N4O	500.25	Rimonabant (Hydrochloride)
Medchem Express	HY-10900	O=C([C@H]1N(C(CSC2=NC3=CC=CC=C3N2C)=O)CCC1)NC4=CC=CC=C4C5=CC=CC=C5	C27H26N4O2S	470.59	TCS 1102
Medchem Express	HY-16297	CC(N1C2=CC(C3=NC(NC4=NC=C(CN5CCN(CC)CC5)C=C4)=NC=C3F)=CC(F)=C2N=C1C)C.CS(=O)(O)=O	C28H36F2N8O3S	602.7	LY2835219
Medchem Express	HY-Y0172	OC1=C(C(C)(C)C)C=C(C)C=C1C(C)(C)C	C15H24O	220.35	2,6-Di-tert-butyl-4-methylphenol
Medchem Express	HY-B1115	O=C(N(CCCCN1CCN(C2=NC=CC=N2)CC1)C(C3)=O)CC43CCCC4.[H]Cl	C21H32ClN5O2	421.96	Buspirone (hydrochloride)
Medchem Express	HY-12990	FC1=CC2=C(N=CN=C2C=C1)NCC3=CC=C(F)C=C3	C15H11F2N3	271.26	Spautin-1
Medchem Express	HY-13763	O=C1CC[C@]2([H])[C@@](CC[C@]3([H])[C@@]4(C)C=CC(C=C4CC[C@]32[H])=O)(C)O1	C19H24O3	300.39	Testolactone
Medchem Express	HY-15164	C#CC1=CC(NC2=C(C=C(OCCOCCOCCO3)C3=C4)C4=NC=N2)=CC=C1.[H]Cl	C22H22ClN3O4	427.88	Icotinib (Hydrochloride)
Medchem Express	HY-10898	FC1=C(C2=C(N3[C@H](CC4=NN=C(C5=CC=CC=C5)O4)CCC3)=O)N=C(C)S2)C=CC=C1	C24H21FN4O2S	448.51	SB-674042
Medchem Express	HY-13260	ClC1=CC=C(C=C1)CC2(CCN(CC2)C3=NC=NC4=C3C=CN4)N	C18H20ClN5	341.84	CCT128930
Medchem Express	HY-10680	OC(C=C1)=CN=C1C2=NOC(CC(C)(C)C(NC3=C(C(O)=O)CCCC3)=O)=N2	C19H22N4O5	386.4	MK-6892
Medchem Express	HY-15494	O=C1OC[C@]2([H])[C@@H](O)C3=C(C=C4OCOC4=C3)[C@@H](C5=CC(OC)=C(OC)C(OC)=C5)[C@@]21[H]	C22H22O8	414.41	AXL1717
Medchem Express	HY-10161	CC1=CC(NC2=NC(SC3=CC=C(C=C3)NC(C4CC4)=O)=NC(N5CCN(CC5)C)=C2)=NN1	C23H28N8OS	464.59	Tozasertib
Medchem Express	HY-102003	FC1=CC2=C3C(CCNC2=O)=C(C4=CC=C(CNC)C=C4)NC3=C1.O=S(C[C@@]5(C6(C)C)C(C[C@@]6([H])CC5)=O)(O)=O	C29H34FN3O5S	555.66	Rucaparib (Camsylate)
